# SEMA4A signaling in macrophage subpopulations and its implication in osteoarthritis

**DOI:** 10.3389/fimmu.2026.1847788

**Published:** 2026-06-05

**Authors:** Yue Qiu, Shuzhong Huang, Bo Yu, Baochen Wei, Tianyu Ren, Xiaofan Yang, Zhanying Shi, Zhaolan Wei

**Affiliations:** Sports Medicine Department, Liuzhou People’s Hospital, Liuzhou, China

**Keywords:** macrophages, osteoarthritis, PLXNB2, Sema4A, single-cell RNA sequencing

## Abstract

**Background:**

Osteoarthritis (OA) is a common degenerative disease characterized by the deterioration of articular cartilage, affecting approximately 240 million people worldwide. Low-grade inflammation—particularly the imbalance in macrophage polarization—is a critical factor in osteoarthritis progression. M1-type macrophages exacerbate cartilage destruction by secreting pro-inflammatory factors and matrix-degrading enzymes, while M2-type macrophages promote repair through anti-inflammatory factors. While macrophage polarization changes in OA have been reported, the macrophage subpopulation communication architecture and dominant ligand–receptor axes across dynamic state transitions remain unclear—and that this is what our integrated framework aims to address.

**Materials and methods:**

This study leverages single-cell transcriptomic data, including 6 normal samples and 12 OA samples, to systematically analyze the interaction patterns and key ligand-receptor pairs of M1/M2 macrophages during OA progression. Methods include cell subset annotation, GSVA functional enrichment, pseudotemporal trajectory analysis, and hdWGCNA network construction. This study provides single-cell-level evidence for the inflammatory mechanisms of OA.

**Results:**

Cell-cell communication analysis revealed strong bidirectional interactions between M1 and M2 macrophages. Integrative analysis of macrophage subpopulations, pseudotime, hdWGCNA, and cell-cell communication analysis identified SEMA4A as the only overlapping key gene. The SEMA4 signaling pathway exhibited active communication among macrophage subpopulations, with M1 macrophages acting as dominant signal senders. Ligand–receptor analysis showed that SEMA4A–PLXNB2 was the predominant interaction pair with the highest communication probability.

**Discussion:**

This study identified a dynamic “increase-then-decrease” expression pattern of SEMA4A along the macrophage pseudotime trajectory, suggesting its involvement in macrophage differentiation and state transitions. Ligand–receptor analysis revealed SEMA4A–PLXNB2 as the dominant interaction signaling pathway among macrophage subpopulations, with M1 macrophages acting as central hubs in sending and receiving signals. Together with previous evidence that SEMA4A forms a positive feedback loop with NF-κB and amplifies IL-6/TNF-α production, thereby promoting cartilage catabolism and tissue remodeling, these findings indicate that, in osteoarthritic joints, SEMA4A–PLXNB2 signaling pathway sustains and amplifies the inflammatory microenvironment through macrophage–stromal crosstalk and represents a potential therapeutic candidate that warrants further validation.

**Conclusion:**

The SEMA4A–PLXNB2 signaling pathway plays an important role in the macrophage-associated inflammatory network and may contribute to the progression of OA, representing a potential therapeutic candidate warranting further validation.

## 1 Introduction

Osteoarthritis (OA) is one of the most common degenerative joint diseases. According to statistics, approximately 240 million people worldwide currently suffer from OA and are deeply affected by it (1). OA is a prevalent age-related disease characterized clinically by joint pain and gradual loss of articular cartilage, ultimately leading to impaired mobility. It can be triggered by various risk factors, such as excessive activity, obesity, increased joint load due to fatigue, and aging (2). With the application of genomics, extensive research has been conducted on the onset, progression, diagnosis, and treatment of OA, leading to the identification of multiple OA-associated genetic risk loci (3).

Increasing evidence suggests that low-grade inflammation also contributes to OA. Among the inflammatory cells involved, macrophages play a pivotal role (4, 5). Generally, macrophages are divided into classically activated (M1) and alternatively activated (M2) (6). Studies have analyzed the detailed changes in macrophages within the synovium and circulation, revealing that during the progression of OAs, M1 macrophages increase in both the synovium and bloodstream, while the population of M2 macrophages decrease (7).

M1 macrophages drive synovitis, oxidative stress, and cartilage catabolism by secreting cytokines (IL-1β, TNF-α) and matrix-degrading enzymes (MMPs, ADAMTS-5), whereas M2 macrophages promote tissue repair through TGF-β and IL-10. Under physiological conditions, tissue-resident macrophages (TRMs) maintain joint homeostasis, but under various stimuli, they may polarize into pro-inflammatory M1 or anti-inflammatory M2 phenotypes. An imbalance in macrophage polarization skewed toward the M1 phenotype leads to persistent inflammation, cartilage degradation, and osteophyte formation, further exacerbating OA symptoms and structural damage. This provides an emerging therapeutic direction, such as macrophage depletion, mTOR/SIRT1 modulation, and M2 polarization, demonstrating the potential to rebalance the M1/M2 ratio and slow OA progression (8–10).

While macrophage polarization changes in OA have been reported (11), the macrophage subpopulation communication architecture and dominant ligand–receptor axes across dynamic state transitions remain unclear—and that this is what our integrated framework aims to address. To clarify these points, we analyzed single-cell transcriptomic data from human knee synovial tissue specimens, including 6 normal samples and 12 OA samples, to investigate signaling pathways involved in OA pathogenesis. Through cell subtype annotation, GSVA, pseudotime trajectory analysis, hdWGCNA and cell-cell communication analysis, we explored the intercellular communication patterns between M1 macrophages and M2 macrophages and identify key signaling pathways and genes. This study deepens our understanding of the role of macrophages in OA, aids in the discovery of potential therapeutic targets, and provides novel insights for the development of new treatments.

## 2 Materials and methods

### 2.1 Sample source and model establishment

This study used human knee joint synovial tissue. A total of 18 synovial samples were collected, including 6 normal samples (non-osteoarthritis control synovial tissues) and 12 OA samples. All participants provided written informed consent, and the study protocol was approved by the Ethics Committee of Liuzhou People’s Hospital. OA samples were obtained intraoperatively from patients with clinically and radiographically confirmed knee osteoarthritis during total knee arthroplasty, arthroscopic surgery, or both. Normal samples were collected only as surgical discard tissues from individuals without evidence of OA (no OA history and no or minimal degenerative changes on imaging, e.g., Kellgren–Lawrence grade 0–1). These individuals underwent arthroscopic procedures for non-OA indications such as traumatic injuries (e.g., meniscal or ligament injury). No additional synovial tissue was removed solely for research purposes. Only small synovial fragments routinely excised during the procedure (e.g., for visualization, debridement, or management of synovial impingement) and otherwise discarded were retrieved. Synovial tissues were harvested under sterile conditions, gently rinsed with pre-chilled phosphate-buffered saline (PBS) to remove blood and debris, immediately placed in cold tissue or cell preservation solution, and transported on ice to the laboratory to preserve RNA quality. Samples were then processed promptly to generate single-cell suspensions for downstream analysis; when temporary storage was required, tissues were cryopreserved in an appropriate freezing medium to ensure RNA integrity. The 18 collected synovial tissue samples were subsequently used for single-cell RNA sequencing (scRNA-seq).

### 2.2 Single cell RNA-seq library construction and sequencing

The fresh knee joint synovial tissue should be rinsed three times with sterile PBS after harvesting. Mince the fresh knee joint synovial tissue and digest it with 0.2% collagenase II (#2195526, Gibco, USA) diluted in DMEM solution at 37 °C for 4 hours. Post-digestion, filter the mixture through a 70-μm cell strainer to remove tissue debris and cell clumps, obtaining a single-cell suspension. Subsequently, count the cells and assess viability; qualified samples proceed to library preparation. Following the manufacturer’s protocol, construct scRNA-seq libraries using the Chromium Single Cell 3’ Library, Gel Bead Kit, and the Chromium Single Cell A Chip Kit (10× Genomics V2, USA). Briefly, cells are loaded into each channel of the 10× chip and partitioned into gel bead-in-emulsion via the Chromium instrument. RNA released from lysed cells is then barcoded for reverse transcription, followed by amplification, fragmentation, and attachment of 5’ adaptors and sample indices. Library sequencing is performed on the Illumina HiSeq Xten platform.

### 2.3 Preprocessing of scRNA-seq data

After obtaining the FASTQ files, the data were processed and expression-quantified using the 10× Genomics analysis pipeline. Specifically, FASTQ files were analyzed with Cell Ranger (version 10.0.0). First, reads were demultiplexed and cells were identified using cell barcode and unique molecular identifier (UMI) information. Subsequently, transcript reads were aligned to the reference genome (GRCh38) and annotated with gene information. Finally, UMI deduplication and counting were performed for each cell to generate a gene expression matrix. Build an expression matrix using the Seurat (version 5.4.0) package. The “Harmony” algorithm is employed to integrate sample data. Quality control and filtering were performed by initially screening for genes expressed in at least three cells and cells expressing a minimum of 200 genes and a maximum of 6,000 genes. Further screening of the matrix was conducted, mitochondrial percentage<10%, ribosomal percentage<20%, hemoglobin percentage<0.1%. Cells with extremely low or high UMI counts were removed. The standard was that the total number of RNA molecules per cell (nCount_RNA) was greater than 300 and less than the 97th percentile, in order to exclude low-quality cells and potential multiplets. The top 1,500 highly variable genes (HVGs) were calculated and returned.

### 2.4 Dimensionality reduction, clustering and annotation

In principal component analysis (PCA), the number of principal components was set to 30. T-distributed random neighborhood embedding (t-SNE) clustering (dimension = 1:40) were done to achieve dimensionality reduction and clustering. Cell type annotation was done using the SingleR (version 2.12.0) and celldex package (version 1.20.0), utilizing the reference marker gene database. We identified marker genes in each cluster using the FindAllMarkers function with the following thresholds: |logFC| > 1, adjusted p-value < 0.05, and minimum percentage of expressing cells (min.pct) > 0.25. We visualized the proportions of cell types within samples and the proportions of samples across cell types using bar charts.

### 2.5 Macrophage subpopulations

To identify clusters within each major cell type, a second round of clustering was performed specifically on macrophages. In PCA, the number of principal components was set to 20. Uniform manifold approximate projection (UMAP) clustering (dimension = 1:15) were done to achieve subdivision of macrophage subpopulations. We identified significantly marker genes in macrophages subpopulations using the FindAllMarkers function with the following thresholds: min.pct > 0.25, logfc.threshold > 0.3. These major cell types were subsequently subdivided into multiple clusters, representing distinct cell subpopulations within the primary lineages.

### 2.6 Gene set variation analysis

The potential biological functional changes of distinct cell types were assessed using the Gene Set Variation Analysis (GSVA) algorithm through the GSVA package (version 2.4.6). We used the Kyoto Encyclopedia of Genes and Genomes (KEGG) and Hallmark to perform pathway enrichment analysis. The reference gene sets used in the analysis were h.all.v2024.1.Hs.symbols.gmt and c2.cp.kegg.Hs.symbols.gmt.

### 2.7 High-dimensional weighted gene co-expression network analysis

A hdWGCNA object created by selecting genes expressed in at least 5% of cells. We construct metacells with KNN parameter set to 25 and a maximum shared cell number of 10, then build the co-expression network using an optimal soft threshold power of 5. We identified gene modules highly correlated with cell types showing significant differences in M1 macrophages and M2 macrophages, calculated module eigengene-based connectivity (kME) and hub gene eigengene (hMEs) values, extracted genes within these modules for downstream analysis.

### 2.8 Pseudotime analysis

To interpret cell differentiation fate decisions, we used Monocle (version 2.38.0) to perform pseudotime ordering on single-cell data. We construct a Monocle object using the newCellDataSet function for macrophages from OA samples (OA macrophages). Pseudotime analysis was performed exclusively on the OA group to investigate state transitions within OA macrophages. To order cells along pseudotime, we utilized variable genes identified by Seurat as ordering genes, thereby building cell differentiation trajectory. Dimensionality reduction was performed using the DDRTree algorithm with the parameter max_components=2. Subsequently, cell trajectories were captured via the orderCells function, and the inferred trajectories were visualized using plot_cell_trajectory. To identify genes critical in distinct cellular states, a branched expression analysis model was employed, detecting significantly DEGs with branch-dependent patterns (thresholds: |logFC| > 1, adjusted p value < 0.05).

### 2.9 Identify overlapping genes and perform cell-cell communication analysis

The CellChat package (version 1.6.1) was employed to identify and visualize the intercellular communication network between OA M1 macrophages and OA M2 macrophages based on scRNA-seq data. Interaction probabilities and strengths between OA M1 and OA M2 macrophages were calculated, and the overall information flow of each signaling pathway was compared. The minimum cell count required for intercellular communication analysis per group was set to 10. Significant ligand-receptor (L-R) pairs with P < 0.05 were extracted, along with pathways specific to OA M1 and OA M2 macrophages. Intersecting gene is obtained from the intersection of the following four gene sets: marker genes from macrophages subpopulations; genes with kME > 0.7 derived from hdWGCNA; shared significantly DEGs across pseudotime state branches; and genes derived from L-R pairs in cell-cell communication. A kME threshold of > 0.7 was applied to identify genes strongly correlated with the module eigengene in hdWGCNA analysis, which is a widely adopted cutoff for defining hub genes or highly module-representative genes. For differential expression analysis, genes with |logFC| > 1 were retained to focus on biologically meaningful expression changes, while filtering out marginal changes that, although potentially statistically significant, are less likely to have functional relevance. Subsequently, we investigated the roles of SEMA4A, the L-R pair involving SEMA4A, and its associated signaling pathways in OA M1 macrophages and OA M2 macrophages.

### 2.10 Construction of co-culture system of inflammatory fibroblasts and macrophages

Fibroblasts and macrophages were co-cultured using a transwell chamber system. Fibroblasts were seeded in the upper chamber, and macrophages were plated in the lower chamber. After cell adhesion, 10 ng/ml IL-1β was added to the upper chamber to establish an inflammatory microenvironment. The co-culture system was then incubated for 36 hours. Fibroblasts and macrophages were separately digested for subsequent experiments.

### 2.11 Real Time-quantitative Polymerase Chain Reaction assay

RT-qPCR assay was conducted to quantitatively detect the mRNA expression level of target genes in related cells and clinical tissues. Total RNA was first extracted from collected cells and clinical tissue samples using a total RNA extraction kit in strict accordance with the manufacturer’s instructions; the purity and concentration of extracted total RNA were detected by a nucleic acid analyzer, and only samples with an A260/A280 ratio of 1.8-2.0 were used for subsequent experiments to ensure RNA integrity and purity. Next, the extracted total RNA was reverse-transcribed into complementary DNA (cDNA) using a reverse transcription kit, with the reaction system prepared on ice to avoid RNA degradation, and the reaction was performed at 42°C for 30 min followed by 85°C for 5 min to inactivate reverse transcriptase, and the obtained cDNA was stored at -20°C for later use. The qPCR reaction was carried out with a fluorescent quantitative PCR instrument, using β-actin(ACTB) as the internal reference gene to normalize target gene expression; specific primers were designed and synthesized by a professional biological company and verified to avoid non-specific amplification, and the reaction system included cDNA template, forward and reverse primers, fluorescent quantitative PCR mix and RNase-free water. The primer sequences are listed in **Supplementary Table 1**. The reaction procedure included pre-denaturation at 95°C for 30 s, followed by 40 cycles of denaturation at 95°C for 5 s, annealing at 60°C for 30 s and extension at 72°C for 30 s, and melting curve analysis was finally performed to confirm PCR product specificity. The Ct value of each sample was recorded, the relative mRNA expression level was calculated by the 2^(-ΔΔCt) method, each sample was set with 3 duplicate wells, and experimental data were expressed as mean ± standard deviation (x ± s) and subjected to statistical analysis using SPSS software. For tissue validation, RT-qPCR was performed using synovial tissues from 3 OA samples and 3 normal control samples, and comparisons between two groups were conducted using an unpaired two-tailed Student’s t-test.

## 3 Results

### 3.1 Distinct cell types were identified in human knee synovial tissue specimens of normal samples and OA samples

**Figure 1:**
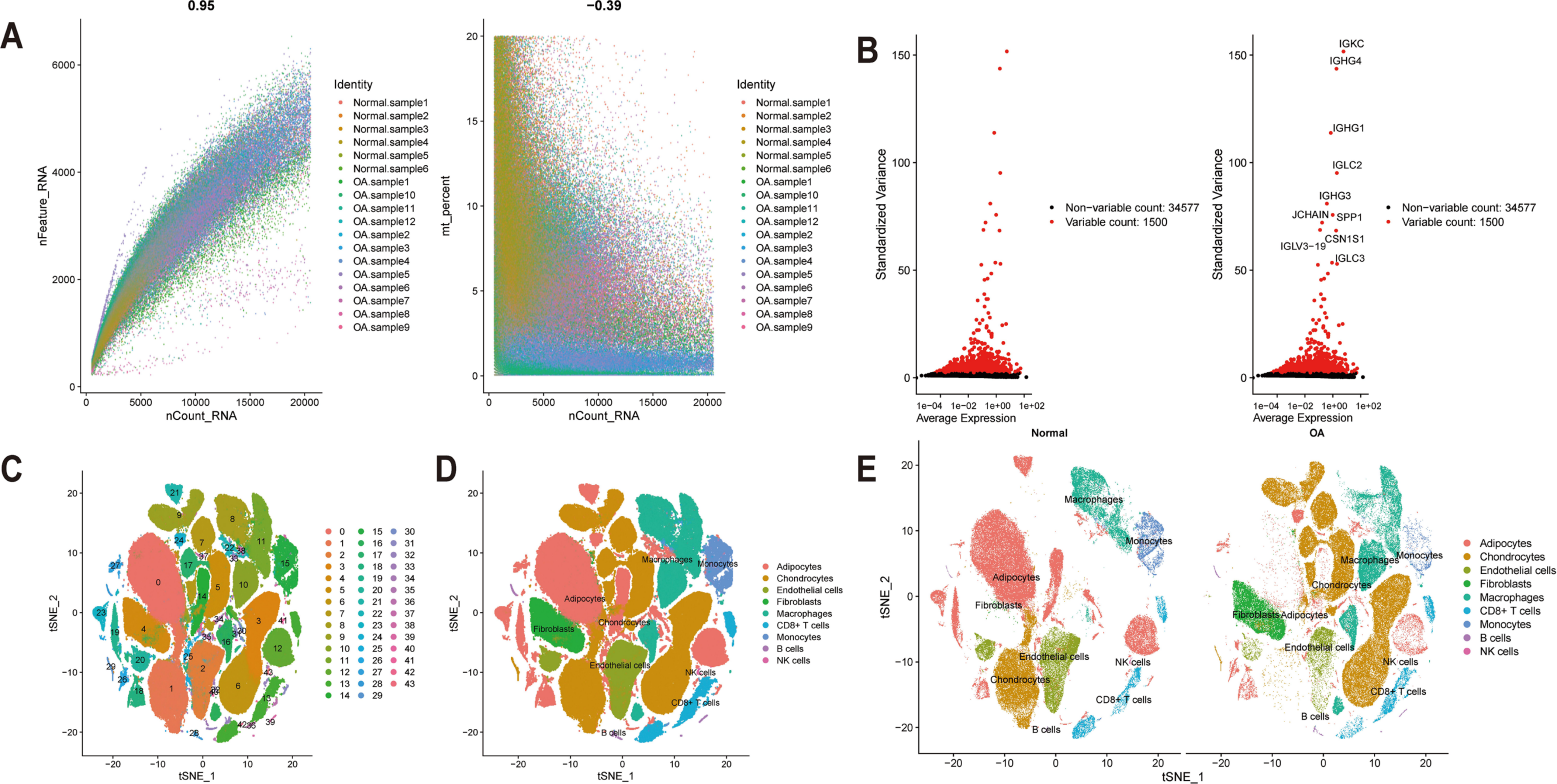
Dimensionality reduction, clustering and annotation. **(A)** Scatter plot of features. **(B)** Highly variable genes. **(C)** 44 clusters were obtained by t-SNE dimension reduction method. **(D)** Distinct cell types were obtained by t-SNE dimension reduction method. **(E)** Distinct cell types in the normal groups and the OA groups, obtained by t-SNE dimension reduction.

To evaluate the quality of the filtered single-cell RNA-seq data, key quality control metrics were examined. A strong positive correlation was observed between the number of detected genes (nFeature_RNA) and UMI counts (nCount_RNA) (r=0.95) (**Figure 1A**). In addition, the percentage of mitochondrial genes (mt%) showed a moderate negative correlation with nCount_RNA (r=−0.39) (**Figure 1A**). These results indicate that the filtered dataset is of high quality for downstream analyses.

Based on the identification of highly variable genes, a total of 1,500 HVGs were identified. Several genes (IGKC, IGHG4, IGHG1, IGLC2, IGHG3, JCHAIN, SPP1, CSN1S1, IGLV3-19, IGLC3) showed high variability (**Figure 1B**).

PCA was subsequently performed and based on the selected principal components (n=30). t-SNE was applied for dimensionality reduction and visualization (n=40). The results showed that all cells were classified into multiple distinct clusters (a total of 44 clusters) (**Figure 1C**). Based on canonical marker genes, these clusters were further annotated, identifying distinct cell types (**Figure 1D**), including adipocytes, fibroblasts, macrophages, among others.

To investigate cellular composition changes between conditions, we compared normal and OA samples. t-SNE visualization revealed distinct distribution patterns of major cell types between the two groups: In OA groups, adipocytes, endothelial cells, and monocytes showed a certain degree of decrease, whereas macrophages was increased (**Figure 1E**).

### 3.2 OA samples undergo substantial cellular remodeling, highlighted by macrophage expansion and increased heterogeneity in cell type proportions

**Figure 2:**
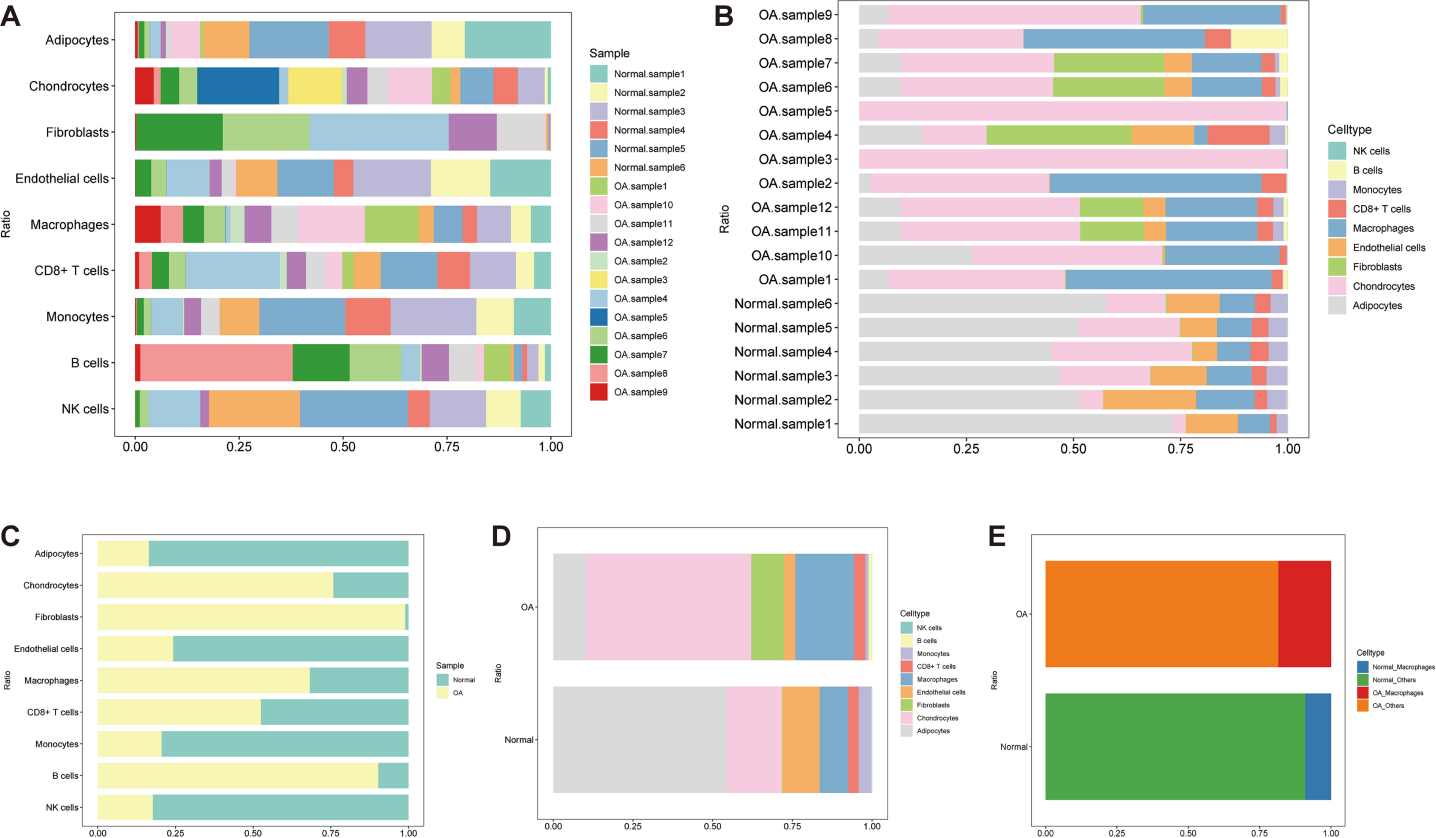
Cell proportion. **(A)** The proportion of 18 samples across distinct cell types. **(B)** The proportion of distinct cell types across 18 samples. **(C)** The proportion of normal groups and OA groups across distinct cell types. **(D)** The proportion of distinct cell types in normal groups and OA groups. **(E)** The proportion of distinct cell types in normal macrophage groups and OA macrophage groups.

Across different samples, substantial heterogeneity in cell type proportions was observed. Compared with normal samples, OA samples showed a marked increase in fibroblasts, B cells, and macrophages, while adipocytes were significantly decreased. Moreover, greater variability in cell proportions was observed among OA samples, for example in fibroblasts and macrophages, whereas cell type distributions in normal samples were relatively stable (**Figures 2A–E**).

### 3.3 Identification of macrophage subpopulations

**Figure 3:**
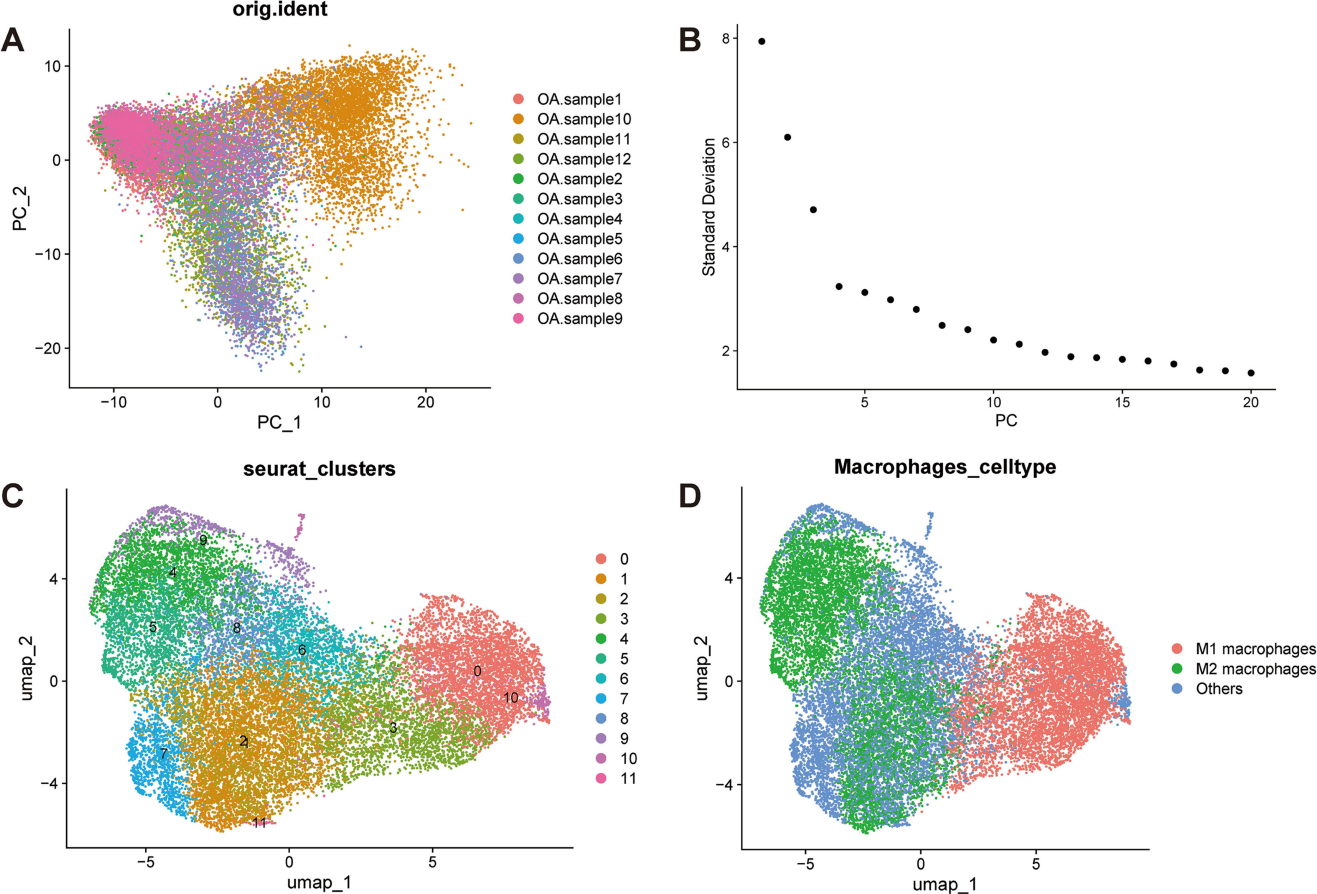
Macrophages subpopulations. **(A)** Dimensionality reduction plot of orig ident. **(B)** The Elbow plot function is used to evaluate PC (n=20). **(C)** 12 clusters were obtained by umap dimension reduction method. **(D)** 3 macrophage subpopulations were obtained by umap dimension reduction method.

Based on PC, the distribution of cells across different samples was evaluated. The results showed that the 12 OA samples (OA.sample1–OA.sample12) showed a relatively homogeneous distribution along the PC1 and PC2 dimensions (**Figure 3A**). Subsequently, the standard deviations of the principal components were assessed (n=15) (**Figure 3B**).

Next, UMAP dimensionality reduction (n=20) and clustering were performed, identifying a total of 12 clusters (cluster 0–11) (**Figure 3C**). Clusters 0 and 3 highly expressed classical pro-inflammatory genes, including IL1B, TNF, IL1A, NLRP3, CCL3, CCL4, CXCL2, CXCL3, and OLR1, suggesting that these clusters exhibit characteristics of M1 macrophages. In contrast, clusters 1, 4, and 5 highly expressed tissue-resident and repair-associated genes such as TREM2, APOE, C1QC, F13A1, LYVE1, STAB1, CD36, and CD74, indicating that they belong to the M2 macrophage subpopulation. Therefore, these clusters was classified into three subtypes: M1 macrophages, M2 macrophages, and others (**Figure 3D**). Among them, M1 and M2 macrophages occupied relatively distinct regions in UMAP space, whereas the others group exhibited a more scattered distribution pattern.

### 3.4 GSVA analysis elucidated the activity changes of pathways associated with different macrophage subpopulations

To systematically evaluate the functional heterogeneity among macrophage subpopulations, GSVA was performed based on both Hallmark and KEGG gene sets. The results revealed substantial differences in biological processes across distinct macrophage populations.

**Figure 4:**
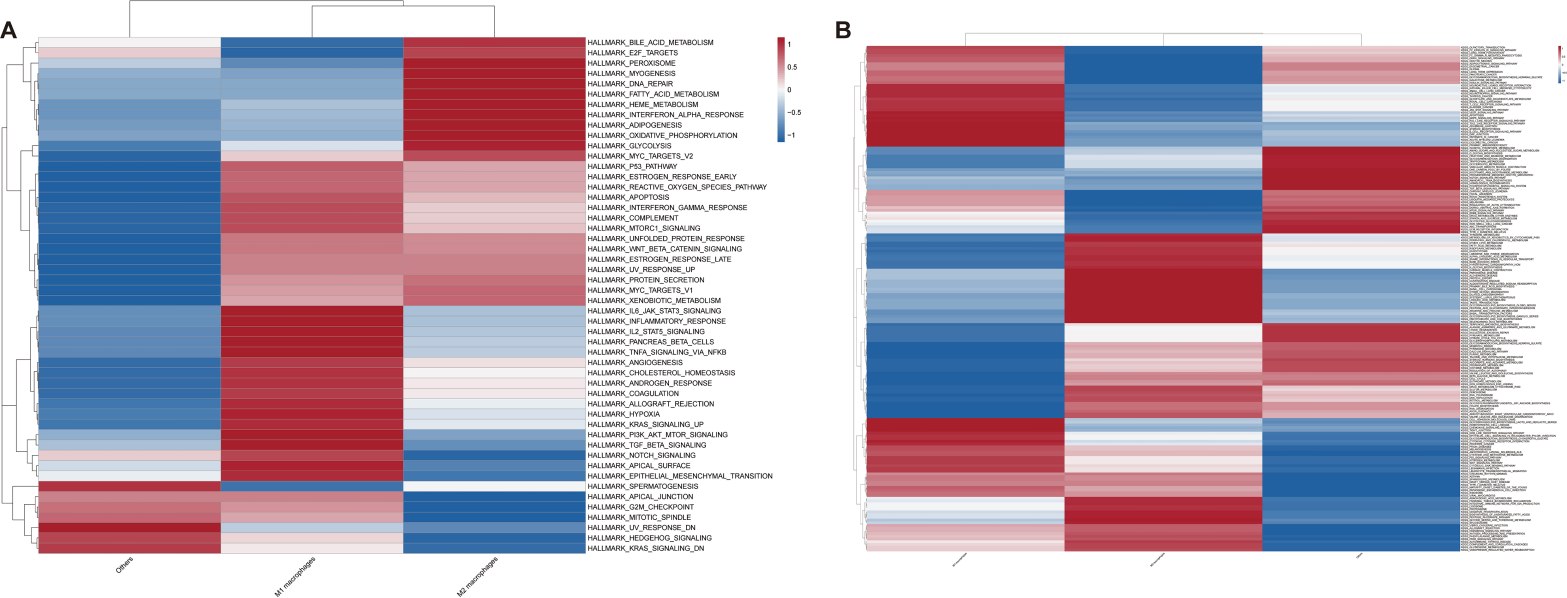
Gene set variation analysis. **(A)** Heatmap of GSVA score of Hallmark. **(B)** Heatmap of GSVA score of KEGG.

Hallmark-based GSVA analysis showed that M1 macrophages exhibited higher activity in inflammation- and immune-related pathways, including HALLMARK_TNFA_SIGNALING_VIA_NFKB and HALLMARK_IL6_JAK_STAT3_SIGNALING. In addition, M1 macrophages significantly enriched glycolysis-related metabolic pathways. In contrast, M2 macrophages displayed relatively higher activity in oxidative phosphorylation, fatty acid metabolism, and angiogenesis pathways, indicating that they actively contribute to anti-inflammatory responses and tissue repair (**Figure 4A**).

Further KEGG-based GSVA analysis provided a more detailed characterization of these differences. M1 macrophages were significantly enriched in multiple immune and inflammatory signaling pathways, including the Toll-like receptor signaling pathway, NOD-like receptor signaling pathway, cytokine–cytokine receptor interaction, chemokine signaling pathway, and JAK–STAT signaling pathway. Meanwhile, M2 macrophages showed higher activity in pathways related to phagocytosis and tissue remodeling, such as Fc gamma R-mediated phagocytosis, endocytosis, lysosome, and ECM–receptor interaction (**Figure 4B**).

Moreover, several pathways closely associated with osteoarthritis progression, including the MAPK signaling pathway, apoptosis, and TGF–βsignaling pathway, showed differential activation across macrophage subpopulations.

### 3.5 hdWGCNA reveals distinct co-expression modules and key hub genes in M1 and M2 macrophages

**Figure 5:**
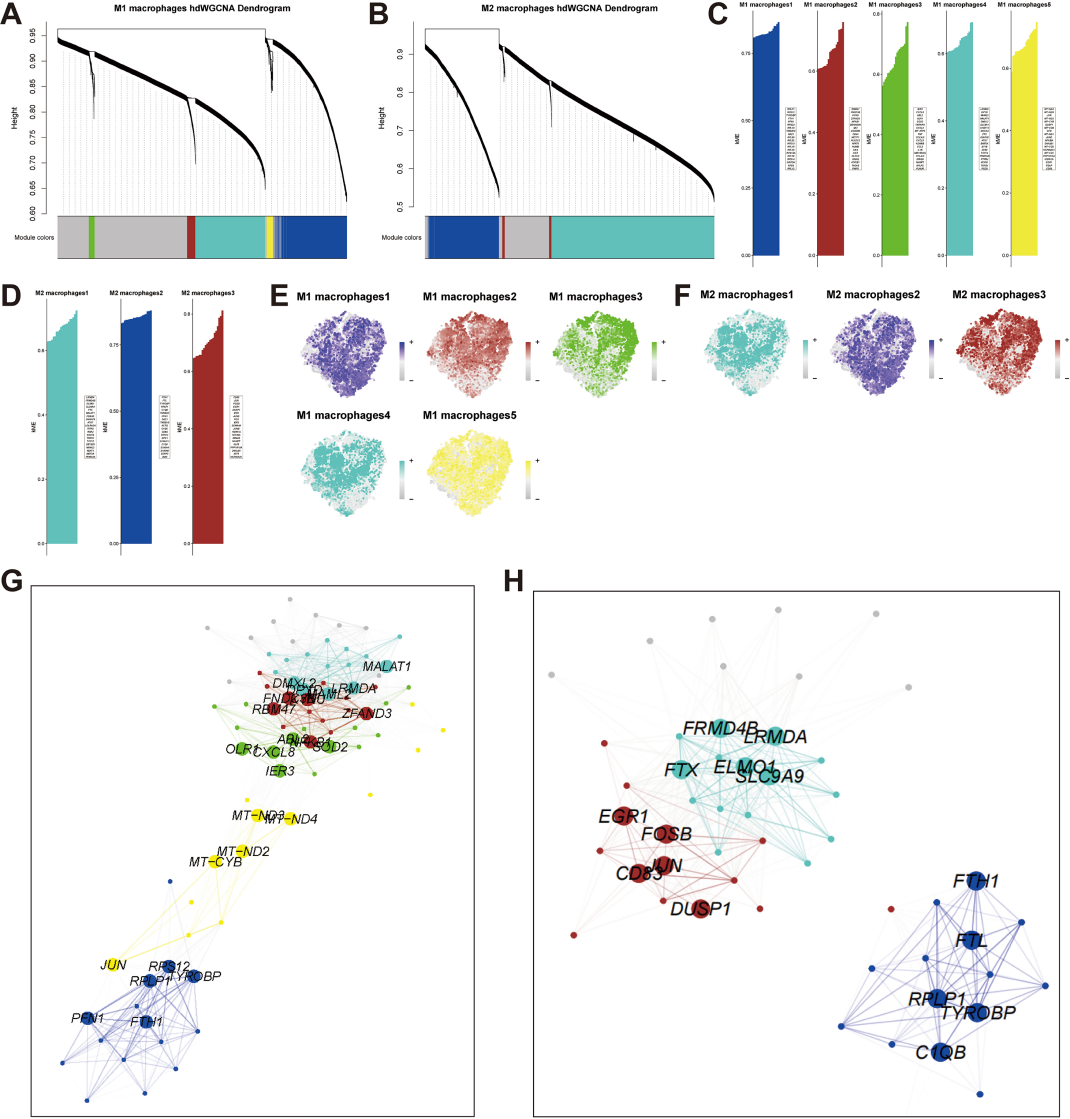
High-dimensional weighted gene co-expression network analysis. **(A)** Dendrogram of M1 macrophages. **(B)** Dendrogram of M2 macrophages. **(C)** KMEs of M1 macrophages. **(D)** KMEs of M2 macrophages. **(E)** Module feature plot of M1 macrophages. **(F)** Module feature plot pf M2 macrophages. **(G)** Module network of M1 macrophages. **(H)** Module network of M2 macrophages.

The dendrogram shows that genes in M1 macrophages are clustered into five major modules (**Figure 5A**), whereas three distinct modules are identified in M2 macrophages, with clear module boundaries (**Figure 5B**).

Module membership analysis in M1 macrophages showed that modules 3 and 5 exhibited relatively high kME values, indicating strong correlations between genes and their respective modules. Module 3 was enriched in inflammatory genes, including CXCL8, TNF, IL1B, and CCL3, whereas module 5 predominantly comprised mitochondrial-related genes such as MT–ND2 and MT–CYB (**Figure 5C**). In M2 macrophages, module 2 displayed relatively high kME values, suggesting strong gene–module correlations. This module included iron homeostasis-related genes (e.g., FTH1 and FTL) as well as canonical M2 markers such as TYROBP and CD68 (**Figure 5D**).

The module feature plots of M1 macrophages reveal varying degrees of expression heterogeneity across co-expression modules (**Figure 5E**). Module 1, module 2and module 5 showed relatively uniform expression, whereas module 3 and module 4 display pronounced spatial heterogeneity with localized high expression in subsets of cells. Similarly, in M2 macrophages, distinct expression patterns are observed across modules (**Figure 5F**). Modules module 2 and module 3 showed relatively uniform expression, while module 1 exhibits marked heterogeneity, with elevated expression restricted to a subset of cells.

In M1 macrophages, the co-expression network comprises five gene modules with extensive interconnectivity, in which FTH1, CXCL8, MT–ND2, and KYNU occupy relatively central positions and exhibit high connectivity (**Figure 5G**). In M2 macrophages, the network consists of three gene modules, with CD83, RPLP1, TYROBP, and ELMO1 showing relatively high connectivity (**Figure 5H**).

### 3.6 Pseudotime analysis suggested dynamic state transitions of OA macrophages

**Figure 6:**
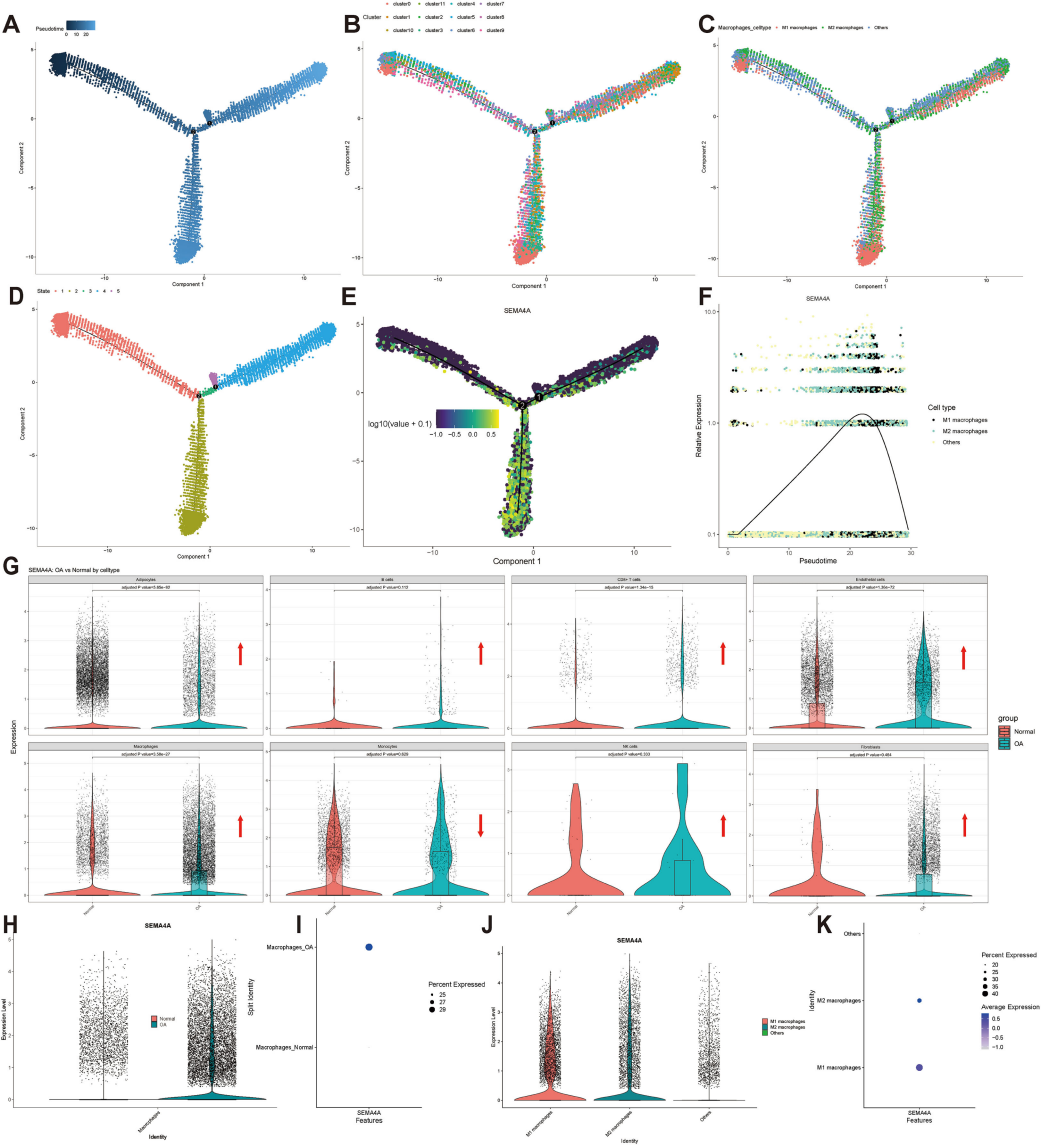
Pseudotime analysis. **(A)** Trajectory of pseudotime of OA macrophages. **(B)** Trajectory of clusters of OA macrophages. **(C)** Trajectory of macrophages subpopulations of OA macrophages. **(D)** Trajectory of states of OA macrophages. **(E)** Trajectory of pseudotime of SEMA4A. **(F)** Trajectory of macrophages subpopulations of SEMA4A. **(G)**Violin plot of expression of SEMA4A across distinct cell types in normal groups and OA groups. **(H)** Violin plot of expression of SEMA4A in normal macrophage groups and OA macrophage groups of 3 cell subpopulations. **(I)** Bubble plot of expression of SEMA4A in normal macrophage groups and OA macrophage groups of 3 cell subpopulations. **(J)** Violin plot of expression of SEMA4A in macrophage subpopulations. **(K)** Bubble plot of expression of SEMA4A in macrophage subpopulations.

We performed pseudotime analysis of macrophages in the OA groups using Monocle. The results showed that different clusters (cluster0–cluster11) of OA macrophages exhibited a continuous pseudotime trajectory with two distinct branch points, corresponding to five cell states (**Figures 6A, B, D**). Further analysis of macrophage subpopulations revealed that M1 macrophages were predominantly distributed at the beginning of the trajectory and at the terminal ends following the branches, whereas M2 macrophages were mainly located in the intermediate regions of the trajectory, with a subset of M2 macrophages also present at the trajectory endpoints (**Figure 6C**).

**Figure 7:**
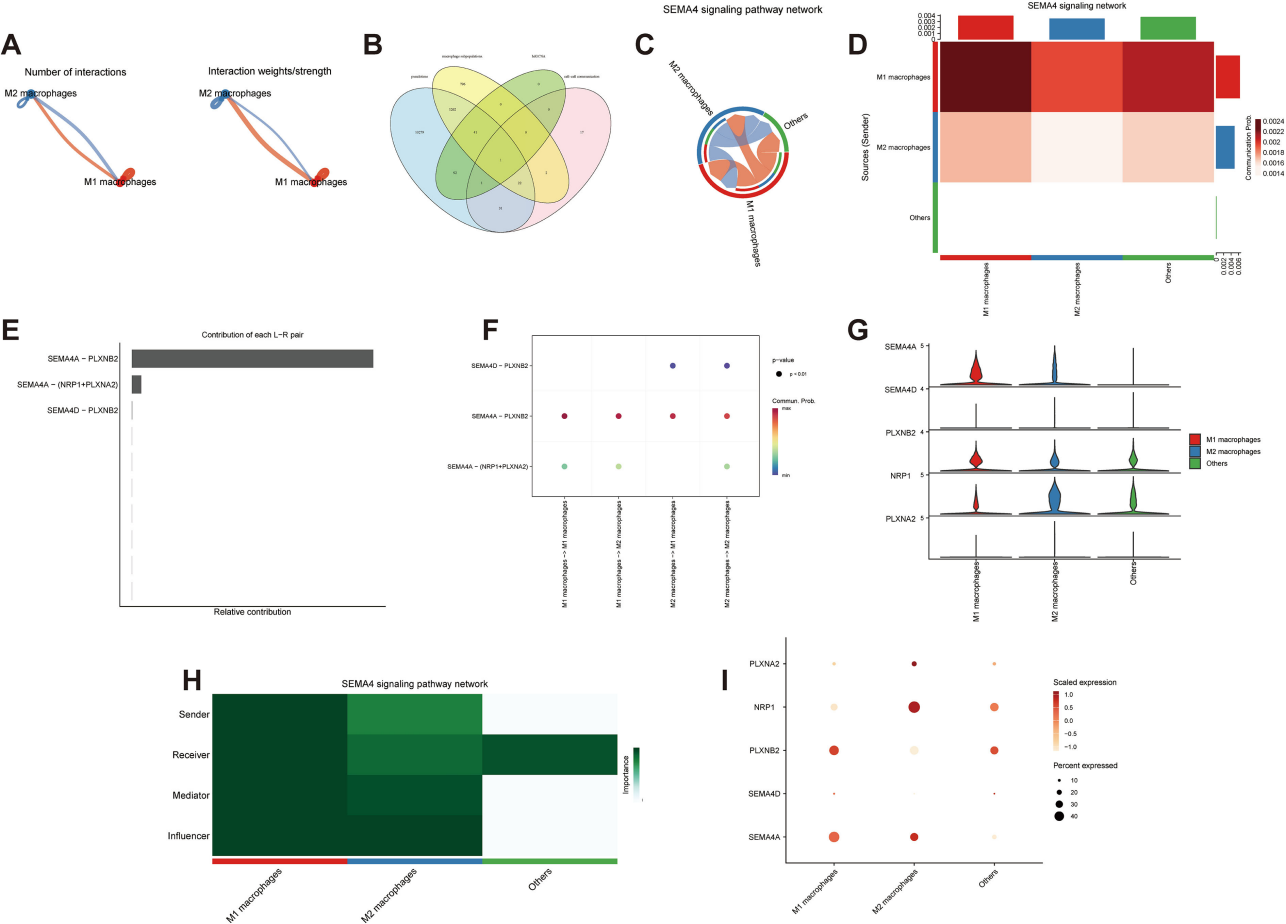
Cell-cell communication analysis. **(A)** Interactions of macrophages subpopulations. **(B)** Venn plot of differentially expressed genes and marker genes **(C)** Circle plot of SEMA4 signaling pathway network of macrophage subpopulations. **(D)** Heatmap of SEMA4 signaling pathway network of macrophage subpopulations. **(E)** Contribution of each L-R pair in SEMA4 signaling pathway network. **(F)** Bubble plot of the relationship between M1 macrophages and M2 macrophages in SEMA4 signaling pathway network. **(G)** Gene expression of macrophages subpopulations in SEMA4 signaling pathway network. **(H)** Signaling role of macrophages subpopulations in SEMA4 signaling pathway network. **(I)** Bubble plot of gene expression of macrophages subpopulations in SEMA4 signaling pathway network.

Intersection analysis was performed among marker genes from macrophage subpopulations, genes with kME > 0.7 derived from hdWGCNA, shared significantly differential genes across pseudotime state branches and genes derived from L–R pairs from cell-cell communication (**Supplementary Tables 2**–**6**). Through this intersection analysis, SEMA4A was identified as the only overlapping gene (**Figure 7B**).

We further investigated the changes in SEMA4A expression along pseudotime in OA macrophage subpopulations. The results showed that the expression of SEMA4A along pseudotime in OA macrophages and their subpopulations. Over time, the gene expression gradually increases and then decreases in the later stages, which is high in early-stage M1 macrophages and mid-to-late-stage M2 macrophages (**Figures 6E, F**).

SEMA4A expression was significantly upregulated in OA compared to the normal groups in multiple cell types, including macrophages, adipocytes, CD8^+^ T cells, and endothelial cells (adjusted P < 0.05). No significant differences were observed in B cells, fibroblasts, monocytes, and NK cells (adjusted P > 0.05) (**Figure 6G**). Given that macrophages were significantly increased in OA samples (**Figure 2C**) and play a critical role in the initiation and progression of inflammation, we subsequently focused on the role of SEMA4A in macrophages.

Macrophages from the OA groups and the normal groups were examined for SEMA4A expression. The results showed that SEMA4A was enriched in macrophages from the OA groups, while exhibiting relatively low expression in macrophages from the normal groups (**Figures 6H, I**).

Subsequently, we analyzed the expression of SEMA4A across different macrophage subpopulations. The results indicated that SEMA4A was highly expressed in both M1 and M2 macrophages, with similar expression levels, suggesting potential interactions between M1 and M2 macrophages. In contrast, the expression of this gene was extremely low in other macrophage subpopulations (**Figures 6J, K**).

### 3.7 Cell-cell communication analysis revealed distinct intercellular communication patterns among macrophage subpopulations

To investigate intercellular communication patterns among macrophage subpopulations using cell-cell communication analysis. Result showed prominent bidirectional communication between M1 and M2 macrophages. In addition, both M1 and M2 macrophages exhibited self-interactions, indicating the presence of autocrine signaling. Overall, the interactions between M1 and M2 macrophages displayed relatively strong communication strength and interaction numbers (**Figure 7A**).

Analysis of the SEMA4 signaling pathway revealed prominent signaling interactions among M1 macrophages, M2 macrophages, and other cell subpopulations (**Figure 7C**). Communication probabilities varied among different cell subpopulations. M1 macrophages showed relatively strong signaling as senders, followed by M2 macrophages, whereas other macrophage subpopulations exhibited minimal signal-sending activity. In contrast, the signal-receiving strength was largely comparable across different macrophage subpopulations (**Figure 7D**).

Multiple ligand–receptor interactions were observed between M1 and M2 macrophages, including both autocrine and paracrine patterns. Among these interactions, the SEMA4A–PLXNB2 pair showed the highest communication probability, followed by SEMA4A–(NRP1+PLXNA2), whereas SEMA4D–PLXNB2 exhibited the lowest level and was only detected when M2 macrophages acted as the sender (**Figures 7E, F**). SEMA4A, as a ligand, showed relatively high expression in both M1 and M2 macrophages, whereas the receptors PLXNB2 and NRP1 were expressed across all macrophage subpopulations. In contrast, PLXNA2 and SEMA4D were minimally expressed or nearly absent across these macrophage subpopulations (**Figures 7G, I**). In SEMA4 signaling pathway network, M1 macrophages demonstrated high importance in signal sending, receiving, mediating, and influencing. In comparison, M2 macrophages were also involved in these processes, but their contributions to signal sending and mediating, particularly receiving, were relatively weaker than those of M1 macrophages. Other cell subpopulations primarily acted as signal receivers (**Figure 7H**).

### 3.8 RT-qPCR assay validated abnormal expression of SEMA4A in OA

**Figure 8:**
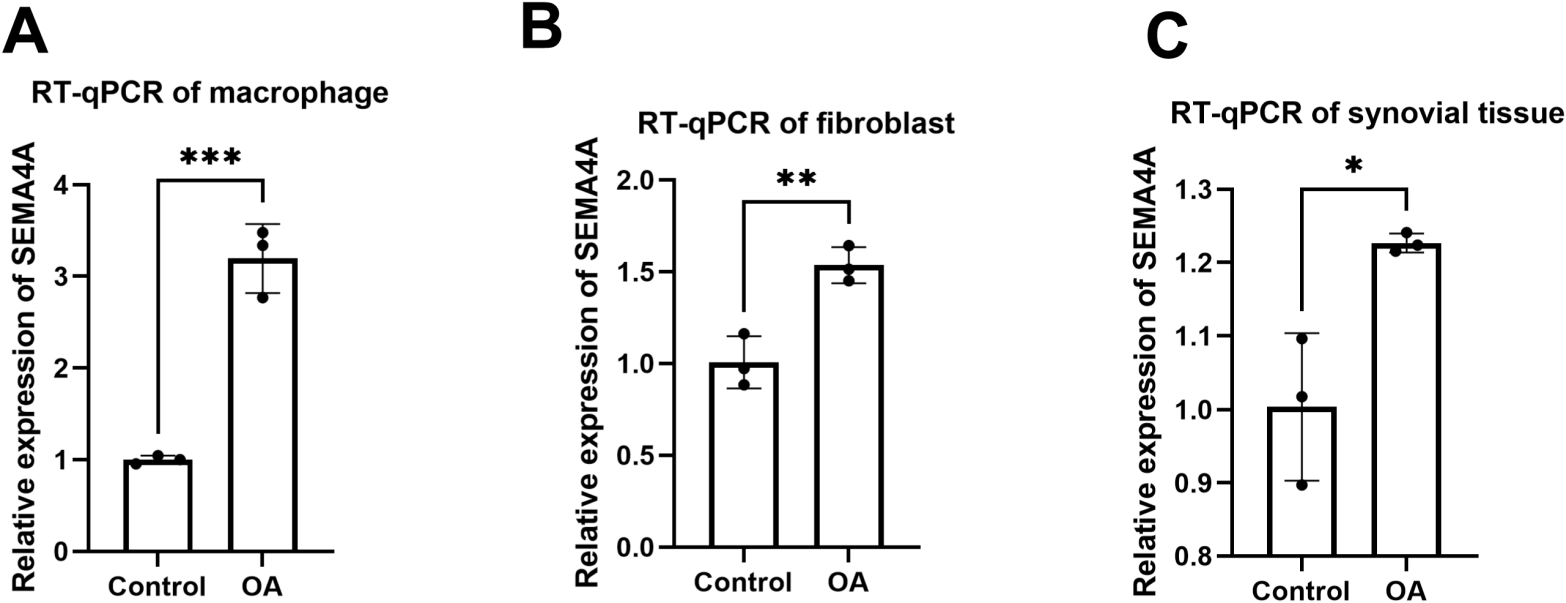
RT-qPCR assay for SEMA4. **(A)** Relative expression of SEMA4 in macrophages. **(B)** Relative expression of SEMA4 in fibroblasts. **(C)** Relative expression of SEMA4 in human OA synovial tissues.

RT−qPCR was performed to detect the mRNA expression level of SEMA4A in inflamed fibroblasts and co−cultured inflamed macrophages, respectively. The results demonstrated that SEMA4A was significantly upregulated in both inflamed fibroblasts and inflamed macrophages compared with the control groups (**Figures 8A, B**). To further verify these findings in clinical specimens, we also examined SEMA4A expression from 3 OA synovial tissues and 3 normal synovial tissues using RT−qPCR. Consistently, SEMA4A expression was markedly increased in OA synovial tissues relative to normal controls (**Figure 8C**). Collectively, these *in vitro* and clinical results confirmed the abnormally high expression of SEMA4A in OA−related cells and tissues, supporting its potential involvement in OA pathogenesis. It should be noted that the ligand–receptor interaction was inferred solely through computational analysis, and the experimental validation was limited to assessing expression levels of the ligand.

## 4 Discussion

OA is a degenerative joint disease characterized by cartilage degradation, chronic inflammation, and progressive joint dysfunction (12). Increasing evidence suggests that the immune microenvironment, particularly macrophage-mediated inflammatory responses, plays a critical role in OA progression. Macrophages regulate inflammation and cartilage homeostasis through cytokine secretion and intercellular communication (13). Therefore, a systematic investigation of macrophage subpopulations and their interactions is essential for understanding OA pathogenesis.

In this study, we performed a comprehensive single-cell transcriptomic analysis of macrophages derived from normal and OA samples. Through cell subtype annotation, GSVA, pseudotime trajectory analysis, and hdWGCNA, we identified significant functional heterogeneity and dynamic changes among macrophage subpopulations. Furthermore, cell-cell communication analysis was applied to explore intercellular communication patterns and identify key signaling pathways and genes.

This study revealed significant heterogeneity in cellular composition across samples, indicating a complex microenvironment in the joints of patients with OA. Compared with the normal groups, macrophages were increased in OA samples. The elevated proportion of macrophages suggests an enhanced inflammatory microenvironment, where macrophages contribute to disease progression by producing inflammatory mediators, growth factors, and proteolytic enzymes (14). The increased abundance of fibroblasts may reflect enhanced tissue remodeling and fibrotic processes, as fibroblasts play key roles in extracellular matrix remodeling and cartilage degradation under pathological conditions (15).

GSVA analysis further demonstrated that M1 macrophages were enriched in inflammation-related and immune-related pathways, including Toll-like receptor, JAK–STAT, and NF–κB signaling pathways, highlighting their pro-inflammatory roles. Consistently, M1 macrophages promote cartilage degradation and synovial inflammation through the secretion of pro-inflammatory cytokines such as TNF–α and IL–1β (16). In contrast, M2 macrophages were enriched in pathways related to phagocytosis, lysosomal activity, and extracellular matrix regulation, including lysosome, endocytosis, and ECM–receptor interaction, suggesting roles in debris clearance and tissue repair (17).

This study applied hdWGCNA to systematically analyze gene co-expression networks in M1 and M2 macrophages derived from osteoarthritic knee joint synovial tissue. Module membership analysis revealed that module 3 in M1 macrophages was enriched in classical inflammatory genes, including CXCL8, TNF, IL1B, and CCL3, indicating that M1 macrophages are in an active inflammatory state (18) and are associated with the pathogenesis of osteoarthritis (19). In contrast, module 2 in M2 macrophages was enriched in iron homeostasis-related genes (e.g., FTH1 and FTL) as well as canonical M2 markers such as TYROBP and CD68, suggesting that M2 macrophages may play an important role in maintaining local tissue homeostasis (20, 21). These findings suggest that the balance between M1 and M2 macrophages is associated with the severity of osteoarthritis.

We first focused on the gene SEMA4A, a key member of the semaphorin family (22). In the pseudotime analysis of this study, SEMA4A expression exhibited a dynamic pattern characterized by an initial increase followed by a subsequent decrease, suggesting its potential involvement in macrophage differentiation and state transitions. Previous studies have demonstrated that SEMA4A plays a critical regulatory role in macrophage activation (23). In addition, SEMA4A exhibited dynamic expression changes along pseudotime, with its expression level increasing initially and then decreasing at later stages, suggesting its potential involvement in macrophage differentiation. Previous studies have shown that SEMA4A forms a positive feedback loop with NF–κB, thereby amplifying cytokine-mediated cartilage catabolism and contributing to the progression of osteoarthritis (24). Moreover, SEMA4A acts as an important pro-inflammatory and tissue-remodeling molecule in rheumatic diseases such as rheumatoid arthritis and systemic sclerosis, supporting our findings (25).

At the ligand–receptor level, SEMA4A–PLXNB2 was identified as the dominant interaction pair, showing the highest communication probability among macrophage subpopulations. SEMA4A was highly expressed in both M1 and M2 macrophages, while its receptor PLXNB2 was broadly expressed across macrophage subpopulations, providing a molecular basis for widespread intercellular communication. In comparison, SEMA4A–(NRP1+PLXNA2) exhibited relatively weaker activity, whereas SEMA4D–PLXNB2 showed minimal expression, suggesting a limited role in this context.

At the pathway level, these interactions collectively indicate that the SEMA4 signaling pathway plays an important role in mediating communication among macrophage subpopulations. In rheumatoid arthritis synovial fibroblasts (RASFs), recombinant human SEMA4A enhances the phosphorylation of STAT3, MAPK, MET, and NF–κB. However, only the NF–κB inhibitor BAY 11-7082 significantly abrogates SEMA4A-induced IL–6 production, indicating that SEMA4A primarily signals through Plexin-B1 to activate NF–κB and subsequently upregulate IL–6. The same study further demonstrates that LPS increases SEMA4A expression in an NF–κB-dependent manner, while SEMA4A in turn activates NF–κB and promotes its own expression, thereby establishing a positive feedback loop (25). In LPS-stimulated THP-1 cells, the expression and secretion of Sema4A are upregulated. Exogenous recombinant human Sema4A (rhSema4A), either alone or in combination with LPS, further enhances the secretion of TNF-α and IL-1β, as determined by ELISA. These findings indicate that, in an inflammatory milieu, Sema4A can potentiate TNF-α (and IL-1β) production by monocyte/macrophage-like cells. This study not only explicitly mentions TNF-α, but also directly demonstrates the TNF-α–upregulating effect of Sema4A, primarily validated in LPS-activated THP-1 cell models and in body fluids from patients with rheumatoid arthritis (26, 27). In STING-activated macrophages, SEMA4D is cleaved and released as soluble SEMA4D via ADAM17-mediated shedding; acting as a cytokine-like molecule, it further amplifies innate immune inflammatory responses (28). These findings support our observation that the SEMA4 pathway is actively involved in macrophage communication, although its role in OA remains to be further investigated.

At the cellular network level, M1 macrophages were identified as central regulators, exhibiting strong contributions in signal sending, receiving, and mediating, whereas M2 macrophages showed relatively weaker involvement. These findings suggest that the SEMA4A–PLXNB2 axis may be primarily driven by M1 macrophages and may influence other cell populations within the inflammatory microenvironment. Consistently, existing studies have shown that under inflammatory conditions, SEMA4A and its receptor PLXNB2 are preferentially induced and function predominantly in M1 macrophages, and they remodel the inflammatory microenvironment through interactions with PLXNB2-positive synovial fibroblasts, T cells, and other cell types (29, 30).

In the joints of patients with OA, SEMA4A is upregulated during the early stages of macrophage differentiation and mediates extensive communication among macrophages, as well as between macrophages and other cell types such as synovial fibroblasts, through its receptor PLXNB2, thereby amplifying inflammatory signaling pathways including NF–κB and cytokines such as IL–6. Given that M1 macrophages predominate in OA and drive chronic synovitis and cartilage degradation, the SEMA4A–PLXNB2 axis is likely primarily driven by M1 macrophages, contributing to the maintenance and amplification of the inflammatory microenvironment in OA (31, 32). Therefore, SEMA4A-mediated macrophage communication may regulate inflammation and play a critical role in OA progression, representing a potential therapeutic target.

However, several limitations should be acknowledged in this study. First, from a sampling perspective, a larger sample size is required to improve the accuracy and robustness of the results. Moreover, the samples analyzed were derived from specific regions and populations, which may limit the generalizability of our findings. Second, regarding data quality, although single-cell RNA sequencing provides a powerful approach to investigate cellular heterogeneity, technical limitations may result in the loss or insufficient capture of gene expression signals in certain cell types, potentially introducing bias into the results. Furthermore, cellular heterogeneity has not been fully elucidated. This is largely because OA is a complex disease involving multiple cell types, including chondrocytes, synovial cells, and various immune cells. While single-cell RNA sequencing can reveal heterogeneity among cell populations, it may fail in some cases to fully capture rare cell types or cells in specific functional states. For instance, cells in acute inflammatory states or at early stages of disease progression may not be adequately identified or characterized, thereby limiting a comprehensive understanding of disease mechanisms. Finally, regarding the interpretation of cell–cell communication, our conclusions based on CellChat analysis are derived from transcriptome co-expression patterns and known databases, which can only infer potential ligand–receptor interactions but cannot demonstrate protein-level binding or functional signal transduction. Therefore, future studies are required to perform perturbation experiments, such as blocking SEMA4A or PLXNB2, to validate the functional role of this signaling axis in OA pathogenesis.

## 5 Conclusion

SEMA4A, through binding to its receptor PLXNB2, constitutes a major ligand–receptor signaling axis mediating communication among macrophage subpopulations as well as between macrophages and other cell types, such as synovial fibroblasts. In OA, SEMA4A is upregulated during the early stages of macrophage differentiation, while its receptor PLXNB2 is widely expressed across multiple macrophage subpopulations, providing a molecular basis for signal transduction and intercellular communication. This SEMA4A–PLXNB2 signaling pathway is primarily driven by pro-inflammatory M1 macrophages and amplifies chronic inflammatory responses by activating the NF–κB pathway and promoting the production of inflammatory cytokines such as IL–6 and TNF–α, thereby facilitating cartilage degradation and tissue remodeling. Therefore, targeting the SEMA4A–PLXNB2 signaling axis may represent a promising therapeutic strategy for alleviating OA and related chronic inflammatory diseases.

## Data Availability

All datasets analyzed for this study are included in the manuscript and/or the **Supplementary Material**.

## References

[1] Katz JN Arant KR Loeser RF . Diagnosis and treatment of hip and knee osteoarthritis: a review. Jama. (2021) 325:568–78. doi: 10.1001/jama.2020.22171. PMID: 33560326 PMC8225295

[2] Mahmoudian A Lohmander LS Mobasheri A Englund M Luyten FP . Early-stage symptomatic osteoarthritis of the knee - time for action. Nat Rev Rheumatol. (2021) 17:621–32. doi: 10.1038/s41584-021-00673-4. PMID: 34465902

[3] Tachmazidou I Hatzikotoulas K Southam L Esparza-Gordillo J Haberland V Zheng J . Identification of new therapeutic targets for osteoarthritis through genome-wide analyses of UK Biobank data. Nat Genet. (2019) 51:230–6. doi: 10.1038/s41588-018-0327-1. PMID: 30664745 PMC6400267

[4] Codispoti G Cavazza L Carniato M Bilancia G Giavaresi G Tschon M . Nanomaterial-based strategies to modulate macrophage polarization in osteoarthritis: a systematic review. Biomater Adv. (2026) 181:214662. doi: 10.1016/j.bioadv.2025.214662. PMID: 41412012

[5] Xie JW Wang Y Xiao K Xu H Luo ZY Li L . Alpha defensin-1 attenuates surgically induced osteoarthritis in association with promoting M1 to M2 macrophage polarization. Osteoarthritis Cartilage. (2021) 29:1048–59. doi: 10.1016/j.joca.2021.04.006. PMID: 33892137

[6] Li H Cao Z Wang L Liu C Lin H Tang Y . Macrophage subsets and death are responsible for atherosclerotic plaque formation. Front Immunol. (2022) 13:843712. doi: 10.3389/fimmu.2022.843712. PMID: 35432323 PMC9007036

[7] Zhu X Lee CW Xu H Wang YF Yung PSH Jiang Y . Phenotypic alteration of macrophages during osteoarthritis: a systematic review. Arthritis Res Ther. (2021) 23:110. doi: 10.1186/s13075-021-02457-3. PMID: 33838669 PMC8035781

[8] Fernandes TL Gomoll AH Lattermann C Hernandez AJ Bueno DF Amano MT . Macrophage: a potential target on cartilage regeneration. Front Immunol. (2020) 11:111. doi: 10.3389/fimmu.2020.00111. PMID: 32117263 PMC7026000

[9] Wu L Cao X Shen B . Development of a macrophage polarization-modulating therapeutic agent for osteoarthritis treatment. J Orthop Surg Res. (2025) 20:279. doi: 10.1186/s13018-025-05679-2. PMID: 40082923 PMC11908040

[10] Zhang K Wang Z He J Lu L Wang W Yang A . Mechanisms of synovial macrophage polarization in osteoarthritis pathogenesis and their therapeutic implications. Front Immunol. (2025) 16:1637731. doi: 10.3389/fimmu.2025.1637731. PMID: 41376619 PMC12685870

[11] Yin X Wang Q Tang Y Wang T Zhang Y Yu T . Research progress on macrophage polarization during osteoarthritis disease progression: a review. J Orthop Surg Res. (2024) 19:584. doi: 10.1186/s13018-024-05052-9. PMID: 39342341 PMC11437810

[12] Ross M Zhou Y English M Sharplin P Hirner M . The effect of intra-articular autologous protein solution on knee osteoarthritis symptoms. Bone Joint J. (2024) 106-b:907–15. doi: 10.1302/0301-620x.106b9.bjj-2024-0258.r1. PMID: 39216848

[13] Mikulkova Z Gallo J Manukyan G Trajerova M Savara J Shrestha B . Complexity of synovial fluid-derived monocyte-macrophage-lineage cells in knee osteoarthritis. Cell Rep. (2024) 43:115011. doi: 10.1016/j.celrep.2024.115011. PMID: 39661512

[14] Shapouri-Moghaddam A Mohammadian S Vazini H Taghadosi M Esmaeili SA Mardani F . Macrophage plasticity, polarization, and function in health and disease. J Cell Physiol. (2018) 233:6425–40. doi: 10.1002/jcp.26429. PMID: 29319160 PMC13482560

[15] Plikus MV Wang X Sinha S Forte E Thompson SM Herzog EL . Fibroblasts: origins, definitions, and functions in health and disease. Cell. (2021) 184:3852–72. doi: 10.1016/j.cell.2021.06.024. PMID: 34297930 PMC8566693

[16] Cutolo M Campitiello R Gotelli E Soldano S . The role of M1/M2 macrophage polarization in rheumatoid arthritis synovitis. Front Immunol. (2022) 13:867260. doi: 10.3389/fimmu.2022.867260. PMID: 35663975 PMC9161083

[17] Yan L Wang J Cai X Liou YC Shen HM Hao J . Macrophage plasticity: signaling pathways, tissue repair, and regeneration. MedComm (2020). (2024) 5:e658. doi: 10.1002/mco2.658. PMID: 39092292 PMC11292402

[18] Hu X Ni S Zhao K Qian J Duan Y . Bioinformatics-led discovery of osteoarthritis biomarkers and inflammatory infiltrates. Front Immunol. (2022) 13:871008. doi: 10.3389/fimmu.2022.871008. PMID: 35734177 PMC9207185

[19] Zhang H Cai D Bai X . Macrophages regulate the progression of osteoarthritis. Osteoarthritis Cartilage. (2020) 28:555–61. doi: 10.1016/j.joca.2020.01.007. PMID: 31982565

[20] Recalcati S Locati M Marini A Santambrogio P Zaninotto F De Pizzol M . Differential regulation of iron homeostasis during human macrophage polarized activation. Eur J Immunol. (2010) 40:824–35. doi: 10.1002/eji.200939889. PMID: 20039303

[21] Winn NC Volk KM Hasty AH . Regulation of tissue iron homeostasis: the macrophage “ferrostat”. JCI Insight. (2020) 5:e132964. doi: 10.1172/jci.insight.132964. PMID: 31996481 PMC7098718

[22] Kumanogoh A Kikutani H . Roles of the semaphorin family in immune regulation. Adv Immunol. (2003) 81:173–98. doi: 10.1016/s0065-2776(03)81005-2 14711056

[23] Meda C Molla F De Pizzol M Regano D Maione F Capano S . Semaphorin 4A exerts a proangiogenic effect by enhancing vascular endothelial growth factor-A expression in macrophages. J Immunol. (2012) 188:4081–92. doi: 10.4049/jimmunol.1101435. PMID: 22442441

[24] Zhang H Wei Q Xiang X Zhou B Chen J Li J . Semaphorin 4A acts in a feed-forward loop with NF-κB pathway to exacerbate catabolic effect of IL-1β on chondrocytes. Int Immunopharmacol. (2019) 69:88–94. doi: 10.1016/j.intimp.2019.01.006. PMID: 30685700

[25] Wang L Song G Zheng Y Tan W Pan J Zhao Y . Expression of Semaphorin 4A and its potential role in rheumatoid arthritis. Arthritis Res Ther. (2015) 17:227. doi: 10.1186/s13075-015-0734-y. PMID: 26303122 PMC4549119

[26] Carvalheiro T Affandi AJ Malvar-Fernández B Dullemond I Cossu M Ottria A . Induction of inflammation and fibrosis by Semaphorin 4A in systemic sclerosis. Arthritis Rheumatol. (2019) 71:1711–22. doi: 10.1002/art.40915. PMID: 31012544 PMC6790618

[27] Ito D Kumanogoh A . The role of Sema4A in angiogenesis, immune responses, carcinogenesis, and retinal systems. Cell Adh Migr. (2016) 10:692–9. doi: 10.1080/19336918.2016.1215785. PMID: 27736304 PMC5160039

[28] Motani K Kosako H . Activation of stimulator of interferon genes (STING) induces ADAM17-mediated shedding of the immune semaphorin SEMA4D. J Biol Chem. (2018) 293:7717–26. doi: 10.1074/jbc.ra118.002175. PMID: 29618514 PMC5961039

[29] Carvalheiro T Rafael-Vidal C Malvar-Fernandez B Lopes AP Pego-Reigosa JM Radstake TRDJ . Semaphorin4A-Plexin D1 axis induces Th2 and Th17 while represses Th1 skewing in an autocrine manner. Int J Mol Sci. (2020) 21:6965. doi: 10.3390/ijms21186965. PMID: 32971928 PMC7555002

[30] Martínez-Ramos S Rafael-Vidal C Marty J Malvar-Fernández B Mouriño C Pérez N . Semaphorin4B is elevated in rheumatoid arthritis and enhances the inflammatory phenotype of macrophages and fibroblast-like synoviocytes. Arthritis Res Ther. (2025) 27:132. doi: 10.1186/s13075-025-03592-x. PMID: 40598553 PMC12219977

[31] Avouac J Pezet S Vandebeuque E Orvain C Gonzalez V Marin G . Semaphorins: from angiogenesis to inflammation in rheumatoid arthritis. Arthritis Rheumatol. (2021) 73:1579–88. doi: 10.1136/annrheumdis-2019-eular.3695 33605067

[32] Zhao K Ruan J Nie L Ye X Li J . Effects of synovial macrophages in osteoarthritis. Front Immunol. (2023) 14:1164137. doi: 10.3389/fimmu.2023.1164137. PMID: 37492583 PMC10364050

