# Supplementary material for: SEMA4A signaling in macrophage subpopulations and its implication in osteoarthritis
Source: Front Immunol. 2026 Jun 5;17:1847788. doi: 10.3389/fimmu.2026.1847788 (PMC13278899; doi:10.3389/fimmu.2026.1847788)
Supplement: Supplementary file 2 [file Table2.docx]

Supplementary Material

# Supplementary Data

## Supplementary Table S1. Primer sequences used for RT-qPCR

| Gene | Forward primer (5′→3′) | Reverse primer (5′→3′) | Product length (bp) |
| --- | --- | --- | --- |
| ACTB | GAGCCCTCATCATCCTCGTG | GCAGAGGTCCTGCATTCCTT | 148 |
| SEMA4A | GTCAAGGCTGAGAACGGGAA | AAATGAGCCCCAGCCTTCTC | 158 |

## Supplementary Table S2. Marker genes from Macrophage subpopulations

| p_val | avg_log2FC | pct.1 | pct.2 | p_val_adj | cluster | gene |
| --- | --- | --- | --- | --- | --- | --- |
| 0 | 2.6151335305731 | 0.78 | 0.242 | 0 | M1 macrophages | TNFAIP3 |
| 0 | 4.71035067134546 | 0.593 | 0.067 | 0 | M1 macrophages | IL1B |
| 0 | 2.86888724094757 | 0.712 | 0.202 | 0 | M1 macrophages | CXCL3 |
| 0 | 3.4702918381485 | 0.786 | 0.276 | 0 | M1 macrophages | OLR1 |
| 0 | 2.68184561670052 | 0.741 | 0.234 | 0 | M1 macrophages | CXCL2 |
| 0 | 3.3577837129396 | 0.614 | 0.139 | 0 | M1 macrophages | LUCAT1 |
| 0 | 2.71902872538471 | 0.853 | 0.379 | 0 | M1 macrophages | NFKB1 |
| 0 | 2.53223833535734 | 0.744 | 0.298 | 0 | M1 macrophages | CCL3 |
| 0 | 3.14523211829099 | 0.588 | 0.149 | 0 | M1 macrophages | MAILR |
| 0 | 3.80540473973559 | 0.497 | 0.064 | 0 | M1 macrophages | BCL2A1 |
| 0 | 2.28323706450046 | 0.718 | 0.288 | 0 | M1 macrophages | CCL4 |
| 0 | 2.3584215796524 | 0.606 | 0.181 | 0 | M1 macrophages | CXCL8 |
| 0 | 2.15130630712227 | 0.87 | 0.457 | 0 | M1 macrophages | PLAUR |
| 0 | 2.73103728489554 | 0.621 | 0.216 | 0 | M1 macrophages | CCL4L2 |
| 0 | 1.65354985292379 | 0.669 | 0.265 | 0 | M1 macrophages | NFKBIZ |
| 0 | 1.61450768141456 | 0.856 | 0.46 | 0 | M1 macrophages | NAMPT |
| 0 | 3.23316521913935 | 0.532 | 0.138 | 0 | M1 macrophages | CCL3L3 |
| 0 | 3.27256898135788 | 0.548 | 0.162 | 0 | M1 macrophages | TNF |
| 0 | 2.24380791988348 | 0.835 | 0.45 | 0 | M1 macrophages | IER3 |
| 0 | 1.94768142897307 | 0.596 | 0.216 | 0 | M1 macrophages | KDM6B |
| 0 | 2.19161499844875 | 0.885 | 0.513 | 0 | M1 macrophages | SOD2 |
| 0 | 2.94653127453402 | 0.509 | 0.139 | 0 | M1 macrophages | GPR183 |
| 0 | 2.52337121634786 | 0.631 | 0.263 | 0 | M1 macrophages | ALCAM |
| 0 | 0.908588368520639 | 0.846 | 0.482 | 0 | M1 macrophages | CD83 |
| 0 | 1.64338682965793 | 0.77 | 0.41 | 0 | M1 macrophages | PPP1R15A |
| 0 | 1.89117745662061 | 0.705 | 0.346 | 0 | M1 macrophages | ABL2 |
| 0 | 1.19839576562881 | 0.721 | 0.367 | 0 | M1 macrophages | NR4A1 |
| 0 | 2.10805948572574 | 0.536 | 0.184 | 0 | M1 macrophages | ZNF267 |
| 0 | 2.00646359371469 | 0.91 | 0.558 | 0 | M1 macrophages | NFKBIA |
| 0 | 1.98669364418834 | 0.547 | 0.2 | 0 | M1 macrophages | HIVEP1 |
| 0 | 2.05744321376826 | 0.57 | 0.223 | 0 | M1 macrophages | HIVEP2 |
| 0 | 4.07062239740932 | 0.408 | 0.061 | 0 | M1 macrophages | MIR155HG |
| 0 | 1.7459432975989 | 0.693 | 0.348 | 0 | M1 macrophages | ELL2 |
| 0 | 2.50955402733187 | 0.867 | 0.524 | 0 | M1 macrophages | KYNU |
| 0 | 2.06906238960519 | 0.525 | 0.185 | 0 | M1 macrophages | ATP13A3 |
| 0 | 1.86449850010412 | 0.649 | 0.313 | 0 | M1 macrophages | FNIP2 |
| 0 | 2.13066817640066 | 0.465 | 0.131 | 0 | M1 macrophages | ENSG00000286533 |
| 0 | 2.6801904743806 | 0.418 | 0.087 | 0 | M1 macrophages | GCH1 |
| 0 | 2.10343171294704 | 0.726 | 0.396 | 0 | M1 macrophages | DENND5A |
| 0 | 2.57371703952624 | 0.4 | 0.074 | 0 | M1 macrophages | IRAK2 |
| 0 | 1.43550288880575 | 0.541 | 0.217 | 0 | M1 macrophages | PDE4B |
| 0 | 2.05301484867582 | 0.562 | 0.238 | 0 | M1 macrophages | ACSL1 |
| 0 | 2.27604637949863 | 0.474 | 0.157 | 0 | M1 macrophages | PPARG |
| 0 | 2.733958898 | 0.445 | 0.129 | 0 | M1 macrophages | NLRP3 |
| 0 | 1.45961632406352 | 0.595 | 0.28 | 0 | M1 macrophages | ICAM1 |
| 0 | 1.25979052424784 | 0.665 | 0.359 | 0 | M1 macrophages | B4GALT1 |
| 0 | 1.71703593110281 | 0.638 | 0.332 | 0 | M1 macrophages | WTAP |
| 0 | 1.80518528828805 | 0.57 | 0.268 | 0 | M1 macrophages | PTPN1 |
| 0 | 2.04787289315154 | 0.563 | 0.261 | 0 | M1 macrophages | SAMSN1 |
| 0 | 2.57031197589013 | 0.521 | 0.221 | 0 | M1 macrophages | EIF4E |
| 0 | 1.55607961234626 | 0.732 | 0.433 | 0 | M1 macrophages | CLIC4 |
| 0 | 3.05906252433938 | 0.418 | 0.119 | 0 | M1 macrophages | SLC39A8 |
| 0 | 1.86790679557852 | 0.487 | 0.191 | 0 | M1 macrophages | USP12 |
| 0 | 1.81959021257767 | 0.461 | 0.169 | 0 | M1 macrophages | NOTCH2NLC |
| 0 | 2.85437324288102 | 0.38 | 0.089 | 0 | M1 macrophages | KMO |
| 0 | 1.67929127252366 | 0.516 | 0.225 | 0 | M1 macrophages | OXSR1 |
| 0 | 2.44210199031232 | 0.498 | 0.209 | 0 | M1 macrophages | SLC16A10 |
| 0 | 1.95511270673366 | 0.53 | 0.242 | 0 | M1 macrophages | ANPEP |
| 0 | 1.29373831932917 | 0.621 | 0.333 | 0 | M1 macrophages | TPRG1 |
| 0 | 2.14268868891217 | 0.556 | 0.27 | 0 | M1 macrophages | PLIN2 |
| 0 | -3.400102618 | 0.122 | 0.406 | 0 | M1 macrophages | F13A1 |
| 0 | 2.12524146419915 | 0.417 | 0.133 | 0 | M1 macrophages | RGS1 |
| 0 | 1.05463796287197 | 0.694 | 0.41 | 0 | M1 macrophages | SIK3 |
| 0 | 1.15086441845868 | 0.658 | 0.375 | 0 | M1 macrophages | ADAM17 |
| 0 | 1.32632800711527 | 0.528 | 0.245 | 0 | M1 macrophages | CDKN1A |
| 0 | 2.0625750384539 | 0.604 | 0.322 | 0 | M1 macrophages | SESTD1 |
| 0 | 1.32900277806732 | 0.817 | 0.536 | 0 | M1 macrophages | PNRC1 |
| 0 | 0.845815030143257 | 0.631 | 0.352 | 0 | M1 macrophages | NR4A2 |
| 0 | 0.915161395850719 | 0.824 | 0.547 | 0 | M1 macrophages | ZFP36 |
| 0 | 3.64083983131293 | 0.33 | 0.055 | 0 | M1 macrophages | KANK1 |
| 0 | 1.28260236230981 | 0.443 | 0.168 | 0 | M1 macrophages | NR4A3 |
| 0 | 1.25168903349957 | 0.692 | 0.417 | 0 | M1 macrophages | TANK |
| 0 | 1.50117874131064 | 0.565 | 0.291 | 0 | M1 macrophages | LPXN |
| 0 | 1.42137972343192 | 0.47 | 0.202 | 0 | M1 macrophages | MAP2K3 |
| 0 | 2.97422012709856 | 0.323 | 0.056 | 0 | M1 macrophages | MIR222HG |
| 0 | 0.946903784950086 | 0.839 | 0.573 | 0 | M1 macrophages | DUSP1 |
| 0 | 4.15495620876447 | 0.311 | 0.047 | 0 | M1 macrophages | AQP9 |
| 0 | 1.040750526 | 0.689 | 0.426 | 0 | M1 macrophages | CFLAR |
| 0 | 1.34377995917757 | 0.641 | 0.379 | 0 | M1 macrophages | ARL8B |
| 0 | 1.06899961160548 | 0.718 | 0.457 | 0 | M1 macrophages | RHOB |
| 0 | 0.961573208490246 | 0.711 | 0.45 | 0 | M1 macrophages | GADD45B |
| 0 | 1.55103275255532 | 0.476 | 0.216 | 0 | M1 macrophages | CSGALNACT2 |
| 0 | 1.1757205169179 | 0.716 | 0.456 | 0 | M1 macrophages | LCP2 |
| 0 | 1.74687909256574 | 0.374 | 0.115 | 0 | M1 macrophages | ZC3H12C |
| 0 | 1.6979110389763 | 0.827 | 0.569 | 0 | M1 macrophages | LIMS1 |
| 0 | 1.14938824710414 | 0.591 | 0.334 | 0 | M1 macrophages | FGR |
| 0 | 2.19211984383582 | 0.336 | 0.08 | 0 | M1 macrophages | DUSP2 |
| 0 | 1.36959692625737 | 0.455 | 0.2 | 0 | M1 macrophages | MAPK6 |
| 0 | 0.981949380923352 | 0.871 | 0.618 | 0 | M1 macrophages | KLF6 |
| 0 | 1.0739579701327 | 0.647 | 0.396 | 0 | M1 macrophages | SGK1 |
| 0 | 1.14950239201568 | 0.637 | 0.388 | 0 | M1 macrophages | ZFAND5 |
| 0 | 2.43418072279644 | 0.371 | 0.122 | 0 | M1 macrophages | GPR132 |
| 0 | 1.7303334838875 | 0.472 | 0.224 | 0 | M1 macrophages | RNF144B |
| 0 | 2.09484410537428 | 0.377 | 0.13 | 0 | M1 macrophages | EIF2AK3 |
| 0 | 0.967701173388232 | 0.733 | 0.487 | 0 | M1 macrophages | SPAG9 |
| 0 | 1.95689122334268 | 0.371 | 0.126 | 0 | M1 macrophages | RIPK2 |
| 0 | 1.01827057412688 | 0.828 | 0.584 | 0 | M1 macrophages | NFE2L2 |
| 0 | 1.19839894461354 | 0.671 | 0.429 | 0 | M1 macrophages | CD58 |
| 0 | 1.872146065 | 0.385 | 0.143 | 0 | M1 macrophages | ARID5B |
| 0 | 1.83633211468693 | 0.362 | 0.12 | 0 | M1 macrophages | B4GALT5 |
| 0 | 1.40960245666601 | 0.404 | 0.163 | 0 | M1 macrophages | SOCS3 |
| 0 | 2.1521288326916 | 0.353 | 0.112 | 0 | M1 macrophages | OTUD1 |
| 0 | 1.73159970049918 | 0.518 | 0.278 | 0 | M1 macrophages | DRAM1 |
| 0 | 1.33809670347247 | 0.709 | 0.471 | 0 | M1 macrophages | NUMB |
| 0 | 1.57005076639441 | 0.799 | 0.564 | 0 | M1 macrophages | ATP2B1 |
| 0 | 1.93093934253315 | 0.335 | 0.1 | 0 | M1 macrophages | B3GNT5 |
| 0 | 1.01617566611823 | 0.745 | 0.513 | 0 | M1 macrophages | PDE4DIP |
| 0 | 1.02301100920776 | 0.884 | 0.652 | 0 | M1 macrophages | RASGEF1B |
| 0 | 2.11765810385946 | 0.916 | 0.691 | 0 | M1 macrophages | CD44 |
| 0 | 2.16886110463677 | 0.313 | 0.088 | 0 | M1 macrophages | THBD |
| 0 | 1.08249959916054 | 0.843 | 0.618 | 0 | M1 macrophages | SRGN |
| 0 | 2.43920249074002 | 0.282 | 0.057 | 0 | M1 macrophages | CYB5D1 |
| 0 | 1.8218084393465 | 0.305 | 0.08 | 0 | M1 macrophages | MAFF |
| 0 | 2.4133374548247 | 0.281 | 0.058 | 0 | M1 macrophages | BIRC3 |
| 0 | 1.4676480924064 | 0.801 | 0.579 | 0 | M1 macrophages | FNDC3B |
| 0 | 2.66887180016979 | 0.278 | 0.057 | 0 | M1 macrophages | ITGAX |
| 0 | 1.0651774915641 | 0.717 | 0.498 | 0 | M1 macrophages | BTG1 |
| 0 | 2.22686773183484 | 0.302 | 0.087 | 0 | M1 macrophages | ACKR3 |
| 0 | 2.26605044528671 | 0.289 | 0.089 | 0 | M1 macrophages | CD109 |
| 0 | 2.04227154762057 | 0.266 | 0.067 | 0 | M1 macrophages | CD300E |
| 0 | 2.05756060991114 | 0.269 | 0.075 | 0 | M1 macrophages | MMP2-AS1 |
| 0 | -1.749591609 | 0.506 | 0.697 | 0 | M1 macrophages | SELENOP |
| 0 | 2.84094025337603 | 0.257 | 0.071 | 0 | M1 macrophages | MMP19 |
| 0 | -1.083927931 | 0.823 | 0.881 | 0 | M1 macrophages | DAB2 |
| 1.97626258336499e-323 | 1.07205794417781 | 0.731 | 0.497 | 7.12976252200586e-319 | M1 macrophages | EPB41L3 |
| 3.95252516672997e-323 | 1.62923055256637 | 0.319 | 0.106 | 1.42595250440117e-318 | M1 macrophages | CSRNP1 |
| 9.09080788347894e-322 | 0.822868655840525 | 0.584 | 0.3 | 3.2796907601227e-317 | M1 macrophages | ATF3 |
| 2.96820410930821e-317 | 1.02764231203699 | 0.686 | 0.441 | 1.07083899651512e-312 | M1 macrophages | MAP3K8 |
| 3.20215575375015e-316 | 1.88685589708725 | 0.362 | 0.144 | 1.15524173128044e-311 | M1 macrophages | GBP2 |
| 2.84085754286033e-315 | 1.14634053347097 | 0.614 | 0.36 | 1.02489617573772e-310 | M1 macrophages | NFAT5 |
| 9.4394690779413e-314 | 0.715632699352151 | 0.817 | 0.604 | 3.40547725924888e-309 | M1 macrophages | JUNB |
| 2.53628131451963e-313 | 1.41457913134704 | 0.451 | 0.21 | 9.15014209839245e-309 | M1 macrophages | STK40 |
| 3.23478496529343e-313 | 2.28804622400172 | 0.251 | 0.07 | 1.16701337192891e-308 | M1 macrophages | CCRL2 |
| 1.74780902753627e-311 | 1.07994108621532 | 0.797 | 0.607 | 6.30557062864259e-307 | M1 macrophages | TAOK3 |
| 1.26016522019415e-310 | 1.87038476492453 | 0.349 | 0.135 | 4.54629806489445e-306 | M1 macrophages | SAV1 |
| 9.99932110540852e-303 | 1.07778192921158 | 0.752 | 0.51 | 3.60745507519823e-298 | M1 macrophages | FGD4 |
| 3.82381391000541e-302 | 0.921011728875754 | 0.676 | 0.424 | 1.37951734431265e-297 | M1 macrophages | PSEN1 |
| 1.28153222525896e-294 | 0.626754610669993 | 0.799 | 0.559 | 4.62338380906674e-290 | M1 macrophages | MCL1 |
| 2.21024172250735e-294 | 0.873955195638286 | 0.807 | 0.588 | 7.97388906228978e-290 | M1 macrophages | PIK3R5 |
| 2.40094648735872e-294 | 0.907955198452761 | 0.669 | 0.429 | 8.66189464244405e-290 | M1 macrophages | BAZ1A |
| 4.92001170187859e-293 | 0.833568522407681 | 0.78 | 0.59 | 1.77499262168674e-288 | M1 macrophages | HNRNPC |
| 1.40842711670959e-292 | 1.2685886694908 | 0.571 | 0.336 | 5.08118250895319e-288 | M1 macrophages | FAM107B |
| 1.07067723253032e-291 | 1.07844129072709 | 0.618 | 0.381 | 3.86268225179963e-287 | M1 macrophages | DSE |
| 3.15467364670845e-291 | 0.885646975707195 | 0.79 | 0.568 | 1.13811161152301e-286 | M1 macrophages | MCTP1 |
| 1.43158313758598e-290 | 1.15211108906957 | 0.436 | 0.195 | 5.16472248546893e-286 | M1 macrophages | LINC01619 |
| 1.13356917931633e-287 | -1.577935677 | 0.376 | 0.597 | 4.08957752821954e-283 | M1 macrophages | MRC1 |
| 3.33157303190809e-287 | 0.923840078616172 | 0.631 | 0.384 | 1.20193160272148e-282 | M1 macrophages | REL |
| 4.24683938588236e-287 | 1.47799355233333 | 0.475 | 0.25 | 1.53213224524478e-282 | M1 macrophages | ITGA5 |
| 8.38808143271595e-287 | 1.27650277264844 | 0.449 | 0.22 | 3.02616813848093e-282 | M1 macrophages | BHLHE40 |
| 3.2628452140767e-286 | 0.727817380621032 | 0.925 | 0.834 | 1.17713666788245e-281 | M1 macrophages | FN1 |
| 5.83765297617611e-285 | 0.973380984766464 | 0.622 | 0.374 | 2.10605006421506e-280 | M1 macrophages | DENND4A |
| 3.11238063487277e-282 | 1.12691359604874 | 0.586 | 0.336 | 1.12285356164305e-277 | M1 macrophages | DOCK10 |
| 6.31200437099842e-280 | 1.28498047404901 | 0.543 | 0.314 | 2.2771818169251e-275 | M1 macrophages | UBE2E1 |
| 4.26310411671154e-279 | -0.774778817 | 0.726 | 0.827 | 1.53800007218602e-274 | M1 macrophages | MS4A6A |
| 2.51061296245009e-276 | -2.085134688 | 0.166 | 0.406 | 9.05753838463117e-272 | M1 macrophages | SLC40A1 |
| 8.09482153152534e-275 | 1.32598153025224 | 0.587 | 0.356 | 2.9203687639284e-270 | M1 macrophages | CAMK2D |
| 1.31163023873024e-274 | 1.69496304709106 | 0.295 | 0.104 | 4.73196841226709e-270 | M1 macrophages | RNF19B |
| 6.42540301568379e-274 | -1.726236378 | 0.27 | 0.501 | 2.31809264596824e-269 | M1 macrophages | EMB |
| 2.08872025776016e-272 | 1.28890434749339 | 0.38 | 0.159 | 7.53547607392131e-268 | M1 macrophages | BCL2 |
| 3.731075147441e-272 | 1.29675533052951 | 0.474 | 0.251 | 1.34605998094229e-267 | M1 macrophages | BID |
| 7.92591602787839e-272 | 0.754181514905504 | 0.87 | 0.688 | 2.85943272537769e-267 | M1 macrophages | DOCK4 |
| 2.86931820573481e-271 | 1.44811082772727 | 0.296 | 0.103 | 1.03516392908295e-266 | M1 macrophages | DOT1L |
| 9.18931770878387e-270 | 1.56881775611202 | 0.457 | 0.239 | 3.31523014979796e-265 | M1 macrophages | INSIG1 |
| 4.14915763386344e-269 | 1.16760714195217 | 0.445 | 0.215 | 1.49689159956891e-264 | M1 macrophages | ETS2 |
| 1.25510369771676e-268 | 1.23650386545919 | 0.527 | 0.291 | 4.52803761025276e-264 | M1 macrophages | IRAK3 |
| 3.12031884349153e-268 | 1.37508319271274 | 0.486 | 0.256 | 1.12571742916644e-263 | M1 macrophages | TLR2 |
| 5.59633514715747e-268 | 1.46938032066564 | 0.314 | 0.117 | 2.01898983104e-263 | M1 macrophages | NEDD9 |
| 9.19956708593429e-267 | -0.908888866 | 0.8 | 0.833 | 3.31892781759251e-262 | M1 macrophages | FCGRT |
| 1.68105951378182e-263 | 0.700085822854333 | 0.693 | 0.471 | 6.06475840787068e-259 | M1 macrophages | JUND |
| 2.48210505233049e-260 | 1.50390990397705 | 0.3 | 0.11 | 8.9546903972927e-256 | M1 macrophages | TP53BP2 |
| 6.22474474941323e-259 | 0.735430617661892 | 0.864 | 0.717 | 2.24570116324581e-254 | M1 macrophages | RAP1B |
| 6.55069076257133e-256 | 1.91095876438033 | 0.353 | 0.155 | 2.36329270641286e-251 | M1 macrophages | RASGRP3 |
| 4.38857660612982e-255 | 0.965522354155765 | 0.67 | 0.467 | 1.58326678219346e-250 | M1 macrophages | LITAF |
| 4.98346689714713e-254 | 1.43185737322142 | 0.353 | 0.149 | 1.79788535248377e-249 | M1 macrophages | ANKRD28 |
| 7.13618509842244e-253 | 1.06094448509802 | 0.682 | 0.483 | 2.57452149795786e-248 | M1 macrophages | GPCPD1 |
| 1.5976711822302e-251 | 0.846243275796453 | 0.72 | 0.509 | 5.76391832413189e-247 | M1 macrophages | CEBPB |
| 5.2499304731494e-251 | 1.15330756998503 | 0.598 | 0.374 | 1.89401741679811e-246 | M1 macrophages | FNDC3A |
| 1.46091014308825e-249 | 0.991007507360052 | 0.615 | 0.369 | 5.27052552321949e-245 | M1 macrophages | SIPA1L1 |
| 5.55660218459825e-244 | 1.0764930755839 | 0.447 | 0.23 | 2.00465537013751e-239 | M1 macrophages | STX11 |
| 3.73227056887147e-243 | 1.89274047428526 | 0.255 | 0.087 | 1.34649125313176e-238 | M1 macrophages | DHX34 |
| 1.43768851131809e-241 | 1.14297688942961 | 0.351 | 0.151 | 5.18674884228229e-237 | M1 macrophages | RELB |
| 2.90031705676179e-241 | -1.759392435 | 0.268 | 0.483 | 1.04634738456795e-236 | M1 macrophages | TPM1 |
| 6.90691860531841e-240 | 0.915598440032005 | 0.581 | 0.352 | 2.49180902524072e-235 | M1 macrophages | STK10 |
| 1.82999445612998e-239 | 1.29215908186386 | 0.31 | 0.123 | 6.60207099938014e-235 | M1 macrophages | ENSG00000287124 |
| 2.22888517116906e-239 | -2.068546942 | 0.209 | 0.429 | 8.04114903202661e-235 | M1 macrophages | LYVE1 |
| 4.2656721477054e-237 | -1.198078516 | 0.724 | 0.793 | 1.53892654072768e-232 | M1 macrophages | LGMN |
| 5.74737908514896e-237 | 1.51078611746814 | 0.289 | 0.111 | 2.07348195254919e-232 | M1 macrophages | ACSL5 |
| 6.90114477528827e-237 | 0.557457380365746 | 0.961 | 0.858 | 2.48972600058075e-232 | M1 macrophages | MT-ND4 |
| 1.95351794294936e-235 | 0.577532613656001 | 0.971 | 0.892 | 7.0477066827784e-231 | M1 macrophages | MT-ATP6 |
| 1.1622861414819e-234 | 0.836229536042555 | 0.707 | 0.513 | 4.19317971262425e-230 | M1 macrophages | IFNGR2 |
| 2.34219547257147e-233 | 1.40552596876822 | 0.278 | 0.103 | 8.44993860639609e-229 | M1 macrophages | FOSL2 |
| 7.05231728898935e-232 | 0.725369710597456 | 0.873 | 0.744 | 2.54426450834869e-227 | M1 macrophages | RBM47 |
| 8.75014746229876e-230 | 0.795860658754386 | 0.859 | 0.704 | 3.15679069997352e-225 | M1 macrophages | FMNL2 |
| 1.20698098374904e-229 | -2.272628571 | 0.258 | 0.459 | 4.35442529507142e-225 | M1 macrophages | MAMDC2 |
| 1.6157325153326e-229 | 0.852375953561368 | 0.719 | 0.526 | 5.82907819556541e-225 | M1 macrophages | PLEK |
| 3.7466313097342e-228 | 0.837871286001561 | 0.602 | 0.375 | 1.35167217761281e-223 | M1 macrophages | GPBP1 |
| 3.68425578505628e-226 | -1.171755431 | 0.406 | 0.596 | 1.32916895957475e-221 | M1 macrophages | GGTA1 |
| 4.02703412101788e-226 | 2.0940998493381 | 0.389 | 0.197 | 1.45283309983962e-221 | M1 macrophages | ZFX |
| 1.95273610921986e-224 | 1.15468591292126 | 0.354 | 0.157 | 7.04488606123247e-220 | M1 macrophages | ZHX2 |
| 3.89295449374281e-224 | 1.08257677296212 | 0.575 | 0.358 | 1.40446119270759e-219 | M1 macrophages | MITF |
| 5.5736174339132e-222 | 0.983728264155885 | 0.469 | 0.255 | 2.01079396163287e-217 | M1 macrophages | PITPNB |
| 1.44760116069847e-221 | 0.697585614252464 | 0.743 | 0.54 | 5.22251070745188e-217 | M1 macrophages | ABR |
| 7.08653715645947e-221 | 1.88986709524248 | 0.252 | 0.093 | 2.55661000993588e-216 | M1 macrophages | SERPINB9 |
| 1.29034726613663e-219 | 0.826272376284219 | 0.729 | 0.541 | 4.65518583204113e-215 | M1 macrophages | ATP1B3 |
| 2.11150767435291e-218 | -2.401742811 | 0.12 | 0.318 | 7.61768623676299e-214 | M1 macrophages | SCN9A |
| 9.45860040933307e-218 | 0.868701956816964 | 0.544 | 0.331 | 3.41237926967509e-213 | M1 macrophages | UBAP1 |
| 1.28389376111265e-216 | 1.17532780683067 | 0.494 | 0.285 | 4.63190352196612e-212 | M1 macrophages | MYO1E |
| 1.46717767429841e-214 | 0.488604490049589 | 0.965 | 0.846 | 5.29313689556636e-210 | M1 macrophages | MT-ND3 |
| 4.45319552713391e-213 | 0.447838994444241 | 0.972 | 0.892 | 1.6065793503241e-208 | M1 macrophages | MT-CO2 |
| 7.2868152156508e-212 | 1.58678212289934 | 0.317 | 0.14 | 2.62886432535034e-207 | M1 macrophages | C5AR2 |
| 9.72243477691396e-211 | 0.420756547423467 | 0.672 | 0.446 | 3.50756279446725e-206 | M1 macrophages | FOSB |
| 2.18429343470154e-210 | 1.61390767491549 | 0.261 | 0.101 | 7.88027542437274e-206 | M1 macrophages | PIM3 |
| 8.98854070796616e-210 | 0.654222136938339 | 0.806 | 0.637 | 3.24279583121295e-205 | M1 macrophages | ZFAND3 |
| 2.22490251747616e-208 | 1.15046195208933 | 0.35 | 0.162 | 8.02678081229876e-204 | M1 macrophages | IER5 |
| 2.35394265746776e-208 | 1.02770081038938 | 0.587 | 0.39 | 8.49231892534645e-204 | M1 macrophages | UBAC2 |
| 5.81513219157145e-208 | 0.515347246852864 | 0.948 | 0.829 | 2.09792524075323e-203 | M1 macrophages | MT-ND2 |
| 1.64032383198267e-207 | 1.08812472463413 | 0.399 | 0.201 | 5.91779628864389e-203 | M1 macrophages | TRAF3 |
| 5.36627213986443e-207 | 1.17955837672307 | 0.313 | 0.136 | 1.93598999989889e-202 | M1 macrophages | BCL6 |
| 9.48121550560567e-207 | -2.814954201 | 0.136 | 0.322 | 3.42053811795736e-202 | M1 macrophages | NAV2 |
| 2.61502644376746e-206 | 1.62819204415586 | 0.319 | 0.149 | 9.43423090117987e-202 | M1 macrophages | TPRA1 |
| 1.73267432913249e-204 | 0.596455604731151 | 0.865 | 0.736 | 6.25096917721129e-200 | M1 macrophages | RAB7A |
| 2.91225510051793e-204 | 1.34043698404903 | 0.432 | 0.247 | 1.05065427261385e-199 | M1 macrophages | RND3 |
| 1.23247786849331e-201 | 1.49756847707495 | 0.352 | 0.174 | 4.4464104061633e-197 | M1 macrophages | MADD |
| 1.33175527957353e-199 | 1.09066527274874 | 0.396 | 0.207 | 4.80457352211742e-195 | M1 macrophages | TIPARP |
| 1.44501964095681e-199 | 1.4912300220543 | 0.25 | 0.095 | 5.21319735867988e-195 | M1 macrophages | GK |
| 1.54399392704612e-198 | 1.17364379628828 | 0.312 | 0.139 | 5.57026689060428e-194 | M1 macrophages | TRIB1 |
| 7.78597987709957e-198 | -1.387522965 | 0.53 | 0.684 | 2.80894796026121e-193 | M1 macrophages | COLEC12 |
| 1.21950643391699e-197 | 1.38906645102624 | 0.338 | 0.163 | 4.39961336164231e-193 | M1 macrophages | CXCR4 |
| 4.6747075444208e-197 | -1.187116297 | 0.476 | 0.644 | 1.68649424080069e-192 | M1 macrophages | STAB1 |
| 8.35706672124114e-196 | 0.789787304862567 | 0.551 | 0.349 | 3.01497896102217e-191 | M1 macrophages | C5AR1 |
| 7.5225706142791e-195 | 0.884751300082698 | 0.42 | 0.221 | 2.71391780051347e-190 | M1 macrophages | PPARD |
| 3.4070080935339e-193 | 0.917986301087453 | 0.445 | 0.239 | 1.22914630990423e-188 | M1 macrophages | RYBP |
| 1.18986775244897e-192 | 0.383302050890251 | 0.981 | 0.914 | 4.29268589051013e-188 | M1 macrophages | MT-CO3 |
| 3.83513484481207e-192 | -1.17490614 | 0.608 | 0.721 | 1.38360159796285e-187 | M1 macrophages | ADAP2 |
| 3.9563216296728e-192 | 0.577662171027173 | 0.747 | 0.538 | 1.42732215433706e-187 | M1 macrophages | FLNA |
| 3.56575137367472e-190 | 0.958128440124573 | 0.353 | 0.169 | 1.28641612308063e-185 | M1 macrophages | PPP1R15B |
| 1.15849942904767e-188 | 1.21933168359198 | 0.317 | 0.147 | 4.17951839017529e-184 | M1 macrophages | BTG3 |
| 2.21854906216681e-188 | 1.06085484869311 | 0.264 | 0.103 | 8.00385945157921e-184 | M1 macrophages | RIPK2-DT |
| 1.09518485124141e-185 | 1.23117259313362 | 0.338 | 0.167 | 3.95109838782363e-181 | M1 macrophages | TGIF1 |
| 1.48321187024215e-183 | 0.650725166286494 | 0.704 | 0.498 | 5.35098346427259e-179 | M1 macrophages | FOXO3 |
| 2.43980390494685e-183 | 1.13427726489731 | 0.596 | 0.399 | 8.80208054787676e-179 | M1 macrophages | TET2 |
| 8.38553602325623e-183 | 0.432551192118148 | 0.61 | 0.392 | 3.02524983111015e-178 | M1 macrophages | EGR1 |
| 1.83665848432627e-181 | 0.922057780165068 | 0.601 | 0.421 | 6.62611281390387e-177 | M1 macrophages | DNAJB1 |
| 5.29910007409317e-180 | 1.11992883446695 | 0.344 | 0.167 | 1.91175633373059e-175 | M1 macrophages | TCF7L2 |
| 9.71664335646546e-180 | 0.676105350711286 | 0.625 | 0.431 | 3.50547342371204e-175 | M1 macrophages | NAP1L1 |
| 8.28952058598152e-179 | 0.829694453487415 | 0.592 | 0.397 | 2.99061034180455e-174 | M1 macrophages | CTNNB1 |
| 3.25527593181497e-178 | -2.616335072 | 0.104 | 0.272 | 1.17440589792089e-173 | M1 macrophages | ABCA6 |
| 1.28512047603086e-177 | 0.401804478773495 | 0.96 | 0.873 | 4.63632914137654e-173 | M1 macrophages | MT-CYB |
| 1.30625683500967e-177 | 0.519744744093359 | 0.83 | 0.665 | 4.7125827836644e-173 | M1 macrophages | SDCBP |
| 5.70998352033052e-177 | 0.907757621221095 | 0.522 | 0.321 | 2.05999075462964e-172 | M1 macrophages | JARID2 |
| 7.73066924985128e-177 | 0.875431085439677 | 0.438 | 0.244 | 2.78899354526885e-172 | M1 macrophages | MAP4K3 |
| 5.98797815666621e-174 | 1.31024416295089 | 0.298 | 0.139 | 2.16028287958047e-169 | M1 macrophages | PDGFB |
| 2.09553474830785e-173 | -1.314448633 | 0.321 | 0.489 | 7.56006071147022e-169 | M1 macrophages | TMEM176B |
| 3.62035508096863e-173 | 0.464175753101797 | 0.939 | 0.839 | 1.30611550256105e-168 | M1 macrophages | SAT1 |
| 1.16840574578037e-172 | 1.26826123053971 | 0.577 | 0.418 | 4.21525740905182e-168 | M1 macrophages | SMS |
| 3.37193988576105e-172 | 0.966033737509006 | 0.526 | 0.335 | 1.21649475258601e-167 | M1 macrophages | KIF13B |
| 5.10477536899399e-171 | 0.659618049617236 | 0.793 | 0.626 | 1.84164980987196e-166 | M1 macrophages | KMT2E |
| 1.28101322744432e-170 | 0.581784258131826 | 0.831 | 0.711 | 4.62151142065086e-166 | M1 macrophages | RTN4 |
| 1.86988468634196e-170 | 0.737432390270541 | 0.63 | 0.433 | 6.74598298291591e-166 | M1 macrophages | RAPGEF1 |
| 8.01220604291816e-170 | 1.04065963724448 | 0.296 | 0.135 | 2.89056357410359e-165 | M1 macrophages | MB21D2 |
| 2.25873678227671e-169 | 0.961352345762509 | 0.449 | 0.265 | 8.14884468941969e-165 | M1 macrophages | SNTB1 |
| 2.73290246239321e-169 | 0.958039311665871 | 0.379 | 0.201 | 9.85949221357599e-165 | M1 macrophages | PAG1 |
| 1.82914777409085e-168 | 0.998543885203143 | 0.36 | 0.187 | 6.59901642458754e-164 | M1 macrophages | PNPLA8 |
| 4.02205317587321e-168 | 0.411821290485463 | 0.66 | 0.451 | 1.45103612425978e-163 | M1 macrophages | IER2 |
| 9.04840366399136e-165 | -1.749388651 | 0.245 | 0.419 | 3.26439258985816e-160 | M1 macrophages | RNF150 |
| 1.52037866393667e-164 | 0.718494270586837 | 0.767 | 0.63 | 5.48507010588432e-160 | M1 macrophages | ABCA1 |
| 1.92440383193922e-164 | 1.06148308557075 | 0.425 | 0.248 | 6.94267170448712e-160 | M1 macrophages | P2RX7 |
| 9.41189639711023e-164 | 0.925770723507915 | 0.545 | 0.381 | 3.39552986318546e-159 | M1 macrophages | FLOT1 |
| 5.65729044381777e-163 | 0.512187836317487 | 0.964 | 0.901 | 2.04098067341614e-158 | M1 macrophages | NEAT1 |
| 1.97487299236595e-161 | 0.952242767625954 | 0.657 | 0.496 | 7.12474929455865e-157 | M1 macrophages | HSPA1A |
| 2.5740879428762e-161 | 0.94663978180584 | 0.501 | 0.31 | 9.28653707151446e-157 | M1 macrophages | FABP5 |
| 6.1514113142303e-161 | 1.28019807954434 | 0.399 | 0.229 | 2.21924465983486e-156 | M1 macrophages | SLC1A3 |
| 1.47527338688467e-159 | 0.737918965813641 | 0.599 | 0.42 | 5.32234379786382e-155 | M1 macrophages | PDLIM5 |
| 4.17838145000323e-159 | -0.98290044 | 0.529 | 0.643 | 1.50743467571766e-154 | M1 macrophages | SNX6 |
| 3.04730013237416e-158 | 0.691950417537891 | 0.604 | 0.429 | 1.09937446875662e-153 | M1 macrophages | MARCKS |
| 3.0479118590463e-158 | 0.844257966721849 | 0.883 | 0.768 | 1.09959516138813e-153 | M1 macrophages | MBNL1 |
| 7.71543548192498e-157 | 0.562828713758683 | 0.642 | 0.45 | 2.78349765881407e-152 | M1 macrophages | SKIL |
| 2.01073961407379e-155 | 0.54448639780233 | 0.78 | 0.615 | 7.25414530569401e-151 | M1 macrophages | LINC-PINT |
| 1.52572782295407e-154 | 0.592835741450631 | 0.602 | 0.423 | 5.5043682668714e-150 | M1 macrophages | DNAJB6 |
| 3.07011074208161e-154 | 0.499429115469911 | 0.846 | 0.703 | 1.10760385242078e-149 | M1 macrophages | QKI |
| 8.00418793472043e-154 | 0.531059778619637 | 0.692 | 0.514 | 2.88767088120909e-149 | M1 macrophages | LHFPL2 |
| 5.76300451980244e-153 | 0.611119813440294 | 0.587 | 0.398 | 2.07911914060913e-148 | M1 macrophages | ADK |
| 5.91815914984659e-151 | -1.32528838 | 0.458 | 0.592 | 2.13509427649016e-146 | M1 macrophages | AP2A2 |
| 6.1967646037639e-150 | 1.15564494733737 | 0.256 | 0.115 | 2.2356067660999e-145 | M1 macrophages | ST18 |
| 8.2686567284061e-150 | 0.850753764450396 | 0.398 | 0.23 | 2.98308328790707e-145 | M1 macrophages | AZIN1 |
| 1.12753427750475e-148 | 0.560334305805524 | 0.66 | 0.485 | 4.0678054129539e-144 | M1 macrophages | KIF1B |
| 1.84111544628083e-148 | 0.717534507430146 | 0.578 | 0.41 | 6.64219219554736e-144 | M1 macrophages | ABI1 |
| 6.0914426743464e-148 | 0.823947647404236 | 0.571 | 0.4 | 2.19760977362395e-143 | M1 macrophages | MAP2K1 |
| 1.08624376742612e-147 | 0.587092265879666 | 0.856 | 0.729 | 3.91884163974321e-143 | M1 macrophages | MSR1 |
| 5.14499583977414e-147 | 0.87735572942064 | 0.269 | 0.122 | 1.85616014911532e-142 | M1 macrophages | CDK14 |
| 5.7071853492395e-147 | 0.613632190524609 | 0.723 | 0.564 | 2.05898125844514e-142 | M1 macrophages | HCK |
| 1.23746905573534e-146 | 0.894798018202461 | 0.574 | 0.416 | 4.4644171123764e-142 | M1 macrophages | TBC1D12 |
| 3.9678708615766e-146 | 0.59816472801817 | 0.573 | 0.39 | 1.43148877073099e-141 | M1 macrophages | HLA-DRB6 |
| 5.22742422511469e-146 | 0.623634418706542 | 0.614 | 0.427 | 1.88589783769463e-141 | M1 macrophages | RAPH1 |
| 1.22034492518886e-145 | 0.845894768345801 | 0.411 | 0.243 | 4.40263838660385e-141 | M1 macrophages | SORL1 |
| 1.40517814769432e-145 | 0.311908081158076 | 0.957 | 0.854 | 5.06946120343681e-141 | M1 macrophages | MT-ND1 |
| 5.67571745917158e-145 | 0.974873189482361 | 0.278 | 0.134 | 2.04762858774533e-140 | M1 macrophages | EGR2 |
| 9.94441113074192e-145 | 0.774934692604004 | 0.334 | 0.173 | 3.58764520363776e-140 | M1 macrophages | NFKBID |
| 1.16862945101369e-144 | 0.76258585865306 | 0.426 | 0.254 | 4.2160644704221e-140 | M1 macrophages | AGAP3 |
| 1.38292337819557e-144 | 0.344394047124342 | 0.975 | 0.918 | 4.98917267151617e-140 | M1 macrophages | MT-CO1 |
| 7.22831192192598e-143 | 0.843427121767168 | 0.472 | 0.306 | 2.60775809207323e-138 | M1 macrophages | RALGDS |
| 2.45506886093544e-142 | 0.76319832378293 | 0.466 | 0.291 | 8.85715192959677e-138 | M1 macrophages | CYRIA |
| 4.83982259785223e-142 | 0.598735854452833 | 0.53 | 0.335 | 1.74606279862715e-137 | M1 macrophages | BRAF |
| 6.37477479812443e-141 | 0.927583340455939 | 0.356 | 0.198 | 2.29982750391935e-136 | M1 macrophages | CLN8 |
| 2.5066241246788e-138 | 1.18695843886798 | 0.258 | 0.123 | 9.04314785460369e-134 | M1 macrophages | TAGAP |
| 2.7992056183152e-138 | 0.701353606080405 | 0.483 | 0.308 | 1.00986941091958e-133 | M1 macrophages | GNA13 |
| 5.34493597976918e-138 | 0.784638736628186 | 0.418 | 0.254 | 1.92829255342133e-133 | M1 macrophages | ID2 |
| 9.09733014282634e-138 | 0.772438522866873 | 0.464 | 0.295 | 3.28204379562746e-133 | M1 macrophages | SLC11A2 |
| 1.34806284352495e-136 | 0.688876196122333 | 0.444 | 0.268 | 4.86340632058498e-132 | M1 macrophages | REV3L |
| 1.41123196838575e-136 | 1.1133689675677 | 0.283 | 0.143 | 5.09130157234528e-132 | M1 macrophages | ATP2B1-AS1 |
| 1.87697465796985e-136 | 0.90706632625651 | 0.381 | 0.219 | 6.77156147355784e-132 | M1 macrophages | NIBAN1 |
| 3.95491296960689e-136 | 0.646793841086724 | 0.613 | 0.448 | 1.42681395204508e-131 | M1 macrophages | EZR |
| 5.880025817039e-136 | -1.161502697 | 0.458 | 0.587 | 2.12133691401316e-131 | M1 macrophages | SLCO2B1 |
| 5.32491945632415e-134 | -1.165822447 | 0.84 | 0.783 | 1.92107119225806e-129 | M1 macrophages | RNASE1 |
| 1.13362016610283e-133 | 0.564158735123835 | 0.747 | 0.578 | 4.08976147324917e-129 | M1 macrophages | ATXN1 |
| 8.78444477583447e-133 | 0.368642434580436 | 0.614 | 0.417 | 3.1691641417778e-128 | M1 macrophages | CMIP |
| 1.220352156037e-132 | 0.904586255991764 | 0.33 | 0.177 | 4.40266447333467e-128 | M1 macrophages | MIR181A1HG |
| 1.69054605492611e-132 | 0.770589817905911 | 0.302 | 0.154 | 6.09898300235692e-128 | M1 macrophages | TNFRSF10B |
| 8.46606086531401e-132 | 0.917620447740444 | 0.27 | 0.132 | 3.05430077837933e-127 | M1 macrophages | LINC00910 |
| 2.75273558871207e-131 | 0.937714426989405 | 0.386 | 0.236 | 9.93104418339654e-127 | M1 macrophages | UPP1 |
| 1.92263652980765e-130 | 0.708650991780462 | 0.564 | 0.399 | 6.93629580858706e-126 | M1 macrophages | ST3GAL1 |
| 1.46917969386477e-129 | 0.590910051679302 | 0.685 | 0.512 | 5.30035958155594e-125 | M1 macrophages | PTPRJ |
| 4.96958931136232e-128 | 0.534915351938051 | 0.592 | 0.419 | 1.79287873586018e-123 | M1 macrophages | CHD2 |
| 4.41506748034193e-127 | 0.796864849188813 | 0.348 | 0.194 | 1.59282389488296e-122 | M1 macrophages | ZNF804A |
| 7.28193899392297e-127 | 0.605171153879501 | 0.703 | 0.556 | 2.62710513083759e-122 | M1 macrophages | MGAT1 |
| 2.86095352308046e-126 | 1.37252801357759 | 0.377 | 0.229 | 1.03214620252174e-121 | M1 macrophages | STK38L |
| 3.85465093206455e-126 | 0.611141280528653 | 0.515 | 0.34 | 1.39064241676093e-121 | M1 macrophages | C9orf72 |
| 3.87047832197144e-126 | 0.62152821565113 | 0.344 | 0.188 | 1.39635246421764e-121 | M1 macrophages | MAPKAPK2 |
| 6.82631964607601e-126 | 0.60018415144543 | 0.511 | 0.344 | 2.46273133871484e-121 | M1 macrophages | ATP1A1 |
| 8.03930293999004e-126 | 0.448185942199611 | 0.792 | 0.665 | 2.90033932166021e-121 | M1 macrophages | TNFAIP2 |
| 1.02442465619802e-125 | 1.00618544041894 | 0.321 | 0.178 | 3.6958168321656e-121 | M1 macrophages | ELOVL5 |
| 1.05471819699168e-125 | 0.89132914472383 | 0.367 | 0.218 | 3.80510683928687e-121 | M1 macrophages | NPC1 |
| 1.7373243399891e-124 | 0.637584467548612 | 0.761 | 0.607 | 6.26774502137868e-120 | M1 macrophages | CHST11 |
| 1.90307972714996e-124 | 0.348694852787302 | 0.657 | 0.475 | 6.8657407316389e-120 | M1 macrophages | AFF4 |
| 2.05089029853926e-124 | -1.439675121 | 0.177 | 0.328 | 7.39899693004008e-120 | M1 macrophages | GAS6 |
| 5.24807020929195e-123 | 0.806380899763166 | 0.351 | 0.201 | 1.89334628940626e-118 | M1 macrophages | CYLD |
| 7.93804189970582e-123 | 0.953614998096015 | 0.38 | 0.227 | 2.86380737615687e-118 | M1 macrophages | ATP2C1 |
| 9.35147815416636e-122 | 0.789170186765796 | 0.324 | 0.178 | 3.3737327736786e-117 | M1 macrophages | WNT2B |
| 7.80511496165578e-121 | 0.522570929045137 | 0.812 | 0.663 | 2.81585132471656e-116 | M1 macrophages | MAML2 |
| 1.30126557641792e-120 | 1.10183049021343 | 0.292 | 0.155 | 4.69457582004294e-116 | M1 macrophages | MAP4K4 |
| 1.71280174584222e-120 | -1.155126423 | 0.763 | 0.725 | 6.17927485847499e-116 | M1 macrophages | C1QC |
| 4.79579395446165e-119 | 0.805518980488874 | 0.416 | 0.264 | 1.73017858495113e-114 | M1 macrophages | ATP6V1H |
| 7.26002715799883e-119 | -1.83878353 | 0.142 | 0.28 | 2.61919999779124e-114 | M1 macrophages | FAM13A |
| 8.69204601127194e-119 | 0.444049840951584 | 0.516 | 0.339 | 3.13582943948658e-114 | M1 macrophages | TSC22D2 |
| 1.92966208812996e-118 | 0.570928498504237 | 0.588 | 0.431 | 6.96164191534647e-114 | M1 macrophages | PAPSS2 |
| 2.35405000893132e-118 | 0.449230340354084 | 0.7 | 0.521 | 8.49270621722151e-114 | M1 macrophages | GSTO1 |
| 3.05634803238184e-118 | 0.643359911208969 | 0.583 | 0.424 | 1.1026386796424e-113 | M1 macrophages | RHOQ |
| 4.55555930249354e-118 | 0.792919555883606 | 0.38 | 0.234 | 1.64350912956059e-113 | M1 macrophages | XBP1 |
| 1.10224870549366e-117 | 0.565341769776128 | 0.299 | 0.152 | 3.97658265480948e-113 | M1 macrophages | IGKC |
| 1.01308345543313e-116 | -1.789256106 | 0.116 | 0.251 | 3.65490118216611e-112 | M1 macrophages | MVB12B |
| 1.71899693191676e-116 | 0.349328845604799 | 0.97 | 0.839 | 6.20162523127608e-112 | M1 macrophages | VIM |
| 4.4620254254723e-115 | 0.978027208425742 | 0.312 | 0.179 | 1.60976491274764e-110 | M1 macrophages | GABARAPL1 |
| 9.19904142734234e-115 | -1.659211196 | 0.283 | 0.414 | 3.3187381757423e-110 | M1 macrophages | NRP1 |
| 2.37679149850842e-114 | -0.704522897 | 0.639 | 0.695 | 8.57475068916883e-110 | M1 macrophages | PMP22 |
| 3.4248846435091e-114 | 0.616549496827422 | 0.535 | 0.381 | 1.23559563283878e-109 | M1 macrophages | PABPC4 |
| 3.43797017798482e-114 | 0.579895473071086 | 0.466 | 0.306 | 1.24031650111158e-109 | M1 macrophages | RABGEF1 |
| 4.85826355838538e-114 | 0.554429821660142 | 0.503 | 0.338 | 1.75271574395869e-109 | M1 macrophages | TIAM1 |
| 9.65245305882912e-114 | -1.000700406 | 0.598 | 0.639 | 3.48231549003378e-109 | M1 macrophages | FOLR2 |
| 1.28239142076797e-113 | 0.541572697630796 | 0.314 | 0.171 | 4.62648352870459e-109 | M1 macrophages | CRY1 |
| 1.42071069195016e-113 | 0.745724810176314 | 0.445 | 0.292 | 5.12549796334859e-109 | M1 macrophages | IL6R |
| 2.91265733685518e-113 | 0.834421671441006 | 0.315 | 0.177 | 1.05079938741724e-108 | M1 macrophages | GNA12 |
| 3.33221214021897e-113 | -0.826222866 | 0.492 | 0.597 | 1.2021621738268e-108 | M1 macrophages | SNX2 |
| 3.54971572003604e-113 | -1.632105356 | 0.282 | 0.414 | 1.2806309403174e-108 | M1 macrophages | WWP1 |
| 8.82560627399059e-113 | 0.622515823184161 | 0.489 | 0.338 | 3.18401397546759e-108 | M1 macrophages | TUBA1C |
| 2.48686589729882e-112 | 0.550310859555757 | 0.582 | 0.429 | 8.97186609768494e-108 | M1 macrophages | RHEB |
| 6.68371047597329e-112 | 0.626617901163174 | 0.5 | 0.34 | 2.41128222841688e-107 | M1 macrophages | NOTCH2 |
| 7.36469433279551e-112 | -1.142129716 | 0.297 | 0.441 | 2.65696077444264e-107 | M1 macrophages | RCSD1 |
| 2.74104948460794e-111 | 0.840123349760874 | 0.284 | 0.152 | 9.88888422562006e-107 | M1 macrophages | LONRF3 |
| 5.7393502633756e-111 | 0.48141165298707 | 0.613 | 0.45 | 2.07058539451801e-106 | M1 macrophages | ARHGAP10 |
| 2.04932027851656e-110 | 0.778086121169977 | 0.317 | 0.179 | 7.39333276880421e-106 | M1 macrophages | NEDD4L |
| 3.3851094085553e-110 | 0.392948699048878 | 0.749 | 0.611 | 1.2212459213245e-105 | M1 macrophages | GNB1 |
| 8.4611323416601e-110 | 0.601362452283895 | 0.485 | 0.327 | 3.05252271490071e-105 | M1 macrophages | HIF1A |
| 2.31808838166366e-109 | 0.472100735067003 | 0.406 | 0.252 | 8.36296745452797e-105 | M1 macrophages | MIDN |
| 2.8386177701054e-109 | 0.479572067977668 | 0.686 | 0.539 | 1.02408813292093e-104 | M1 macrophages | JAK1 |
| 4.85024905369567e-109 | 0.308082649696986 | 0.886 | 0.762 | 1.74982435110179e-104 | M1 macrophages | PABPC1 |
| 5.04440726705225e-109 | 0.838795111132211 | 0.348 | 0.208 | 1.81987080973444e-104 | M1 macrophages | CYTIP |
| 1.04207198541417e-108 | -0.603257329 | 0.853 | 0.816 | 3.75948310177872e-104 | M1 macrophages | ITM2B |
| 1.54771759496753e-108 | 0.507699269884388 | 0.651 | 0.496 | 5.58370076736435e-104 | M1 macrophages | SLC11A1 |
| 7.10983019419767e-108 | -0.946481237 | 0.466 | 0.588 | 2.56501343916069e-103 | M1 macrophages | SIGLEC1 |
| 1.46426400464385e-107 | 0.446190385488987 | 0.837 | 0.725 | 5.2826252495536e-103 | M1 macrophages | APLP2 |
| 1.64442566018394e-107 | 0.953944924689997 | 0.3 | 0.172 | 5.93259445424559e-103 | M1 macrophages | MRAS |
| 1.0888443585117e-106 | -1.136123801 | 0.844 | 0.87 | 3.92822379220266e-102 | M1 macrophages | RBPJ |
| 1.3151752391686e-106 | 0.713457256343852 | 0.288 | 0.154 | 4.74475771034857e-102 | M1 macrophages | PHLPP1 |
| 2.1992744431115e-106 | -1.504954923 | 0.414 | 0.523 | 7.93432240841337e-102 | M1 macrophages | ITSN1 |
| 3.24289493283234e-106 | 0.456595174095756 | 0.69 | 0.56 | 1.16993920491792e-101 | M1 macrophages | RPL23 |
| 3.90472109765312e-106 | 0.793321986376233 | 0.417 | 0.273 | 1.40870623040032e-101 | M1 macrophages | ITGAV |
| 9.93823045376023e-106 | 0.635786823502117 | 0.51 | 0.356 | 3.58541540080308e-101 | M1 macrophages | NR1H3 |
| 1.44687841110278e-105 | -0.598761216 | 0.667 | 0.732 | 5.21990324373548e-101 | M1 macrophages | TGFBI |
| 4.06785191093452e-105 | 0.898010859239765 | 0.696 | 0.583 | 1.46755893390785e-100 | M1 macrophages | SNX9 |
| 2.11614746142739e-104 | 0.710318512647196 | 0.365 | 0.228 | 7.6344251965916e-100 | M1 macrophages | TOM1 |
| 2.38647152985313e-104 | 0.370570247670201 | 0.849 | 0.733 | 8.60967333825116e-100 | M1 macrophages | HSP90AA1 |
| 4.89568195098284e-104 | 0.763902840203328 | 0.406 | 0.267 | 1.76621517745608e-99 | M1 macrophages | ATP6V1C1 |
| 2.50073233579206e-103 | 0.909992499476088 | 0.294 | 0.162 | 9.02189204783701e-99 | M1 macrophages | ENSG00000253557 |
| 6.53673055870264e-103 | 0.578419224711469 | 0.46 | 0.309 | 2.35825628366315e-98 | M1 macrophages | MYO1G |
| 1.04442287484266e-102 | 0.54344667286175 | 0.541 | 0.394 | 3.76796440556988e-98 | M1 macrophages | IQSEC1 |
| 1.76984782267101e-102 | 0.320668733668231 | 0.735 | 0.57 | 6.38507998985018e-98 | M1 macrophages | WSB1 |
| 5.64341805841489e-102 | 0.414515724962356 | 0.702 | 0.564 | 2.03597593293434e-97 | M1 macrophages | ACTR3 |
| 1.66947333482756e-101 | 0.703256653395124 | 0.4 | 0.259 | 6.02295895005739e-97 | M1 macrophages | METRNL |
| 3.10675899109209e-101 | -1.656697967 | 0.5 | 0.577 | 1.12082544121629e-96 | M1 macrophages | PDE4D |
| 7.06625583531938e-101 | 0.3500190059587 | 0.853 | 0.693 | 2.54929311770817e-96 | M1 macrophages | ANXA5 |
| 7.27292313273426e-101 | -2.205454054 | 0.149 | 0.268 | 2.62385247859654e-96 | M1 macrophages | HRH1 |
| 9.14190925776606e-101 | 0.427362287027449 | 0.723 | 0.562 | 3.29812660292426e-96 | M1 macrophages | CTSL |
| 2.95680888168296e-100 | 0.598084031759561 | 0.439 | 0.295 | 1.06672794024476e-95 | M1 macrophages | DDX21 |
| 3.37588853061236e-100 | 0.768333297772545 | 0.368 | 0.236 | 1.21791930518902e-95 | M1 macrophages | ECE1 |
| 1.0698478773515e-99 | 0.994052622199785 | 0.417 | 0.283 | 3.85969018712102e-95 | M1 macrophages | TNFAIP8 |
| 2.12297490873092e-99 | -1.699132637 | 0.189 | 0.313 | 7.65905657822855e-95 | M1 macrophages | MS4A4E |
| 2.31145619596216e-99 | 0.500784850534712 | 0.417 | 0.267 | 8.33904051817268e-95 | M1 macrophages | MECP2 |
| 7.49222119048038e-99 | 0.687172941553563 | 0.389 | 0.247 | 2.70296863888961e-94 | M1 macrophages | SERTAD2 |
| 8.00337365727158e-99 | 0.750795989165054 | 0.427 | 0.287 | 2.88737711433387e-94 | M1 macrophages | RGCC |
| 2.92293367567307e-98 | 0.375400632341378 | 0.651 | 0.483 | 1.05450678217257e-93 | M1 macrophages | TRIO |
| 3.44158912901799e-98 | 0.713327745185656 | 0.387 | 0.247 | 1.24162211007582e-93 | M1 macrophages | AGPAT4 |
| 1.51808574395993e-97 | -1.210295266 | 0.222 | 0.349 | 5.47679793848423e-93 | M1 macrophages | TMEM176A |
| 2.92162215818388e-97 | 0.408458041819263 | 0.616 | 0.457 | 1.054033626008e-92 | M1 macrophages | PTPRM |
| 3.3165802731928e-97 | 0.512825878611775 | 0.62 | 0.475 | 1.19652266515977e-92 | M1 macrophages | AHR |
| 4.26090453160695e-97 | 0.519543561978328 | 0.394 | 0.249 | 1.53720652786784e-92 | M1 macrophages | CLK1 |
| 4.33578462019863e-97 | 0.748141137024598 | 0.322 | 0.189 | 1.56422101742906e-92 | M1 macrophages | CPEB2 |
| 1.3739349887123e-96 | -0.815264559 | 0.546 | 0.618 | 4.95674525877736e-92 | M1 macrophages | MS4A4A |
| 1.48508016975966e-96 | 0.444357315710414 | 0.527 | 0.377 | 5.35772372844194e-92 | M1 macrophages | PLEKHM2 |
| 2.22535401916676e-96 | 0.565928916498377 | 0.453 | 0.312 | 8.02840969494793e-92 | M1 macrophages | ATF4 |
| 2.38701188597475e-96 | 0.337374950362823 | 0.547 | 0.38 | 8.61162278103112e-92 | M1 macrophages | BACH1 |
| 3.71863627881242e-96 | 0.373583567615731 | 0.745 | 0.61 | 1.34157241030716e-91 | M1 macrophages | MSN |
| 8.24699891734351e-96 | 0.599608176265144 | 0.304 | 0.173 | 2.97526979941002e-91 | M1 macrophages | XYLT1 |
| 1.95121187926333e-95 | -0.903393096 | 0.816 | 0.832 | 7.03938709681831e-91 | M1 macrophages | FRMD4B |
| 2.47758083304807e-95 | 0.415702712573546 | 0.757 | 0.626 | 8.93836837138752e-91 | M1 macrophages | CCDC88A |
| 1.81077975857042e-94 | 0.363395174441914 | 0.727 | 0.583 | 6.53275013499449e-90 | M1 macrophages | CYTH1 |
| 2.41029441093459e-94 | 0.526865209721889 | 0.471 | 0.326 | 8.6956191463287e-90 | M1 macrophages | YME1L1 |
| 6.60436922439435e-94 | 0.782965069813216 | 0.257 | 0.141 | 2.38265828508475e-89 | M1 macrophages | MIR22HG |
| 1.56860263495746e-93 | 1.06108004758005 | 0.252 | 0.139 | 5.65904772613605e-89 | M1 macrophages | SLC2A3 |
| 3.66759090614826e-93 | -0.619282086 | 0.854 | 0.778 | 1.32315677121111e-88 | M1 macrophages | C1QA |
| 1.15949067715665e-92 | 0.375360906040838 | 0.702 | 0.537 | 4.18309451597806e-88 | M1 macrophages | RUNX1 |
| 1.79852713949256e-92 | 0.636850836247837 | 0.416 | 0.282 | 6.48854636114732e-88 | M1 macrophages | PEA15 |
| 7.53698321320342e-92 | 0.791424858225722 | 0.299 | 0.178 | 2.7191174338274e-87 | M1 macrophages | ARRDC3 |
| 3.73296543710707e-91 | 0.657907999756493 | 0.348 | 0.215 | 1.34674194074512e-86 | M1 macrophages | PDE8A |
| 3.99800853258036e-91 | 0.547334911948928 | 0.393 | 0.255 | 1.44236153829901e-86 | M1 macrophages | YTHDF3 |
| 4.43875096300907e-91 | 0.622040485588528 | 0.536 | 0.406 | 1.60136818492478e-86 | M1 macrophages | PILRA |
| 1.09476940137101e-90 | -1.456383968 | 0.176 | 0.3 | 3.94959956932619e-86 | M1 macrophages | GNG2 |
| 6.61031007283997e-90 | -1.502556633 | 0.207 | 0.33 | 2.38480156497848e-85 | M1 macrophages | TBC1D14 |
| 9.29380112893428e-90 | 0.400284070912449 | 0.591 | 0.442 | 3.35292463328562e-85 | M1 macrophages | CPEB4 |
| 1.136179339014e-89 | -0.978682735 | 0.471 | 0.569 | 4.09899420136081e-85 | M1 macrophages | MAF |
| 1.24505226335641e-89 | 0.557006375333534 | 0.541 | 0.404 | 4.49177505051093e-85 | M1 macrophages | PDCD6IP |
| 2.58008555813371e-89 | 0.300829743805166 | 0.997 | 0.977 | 9.30817466807899e-85 | M1 macrophages | MALAT1 |
| 3.59531479132184e-89 | -1.088480131 | 0.349 | 0.469 | 1.29708171726518e-84 | M1 macrophages | TPCN1 |
| 3.599393841006e-89 | 0.642542149039641 | 0.386 | 0.252 | 1.29855331601974e-84 | M1 macrophages | VRK2 |
| 7.78082820047709e-89 | 0.393671260539849 | 0.783 | 0.641 | 2.80708938988612e-84 | M1 macrophages | DLEU2 |
| 8.79306654945923e-89 | 0.603695287910893 | 0.508 | 0.374 | 3.17227461904841e-84 | M1 macrophages | EML4 |
| 1.03614184761725e-88 | 0.711555580732959 | 0.409 | 0.274 | 3.73808894364876e-84 | M1 macrophages | SGMS2 |
| 2.49656973933224e-88 | 0.853100834822205 | 0.342 | 0.219 | 9.00687464858892e-84 | M1 macrophages | DDX60L |
| 4.09215031391347e-88 | 0.512923719839186 | 0.41 | 0.267 | 1.47632506875056e-83 | M1 macrophages | CAMSAP2 |
| 6.63185724311235e-88 | 0.772281131114146 | 0.305 | 0.187 | 2.39257513759764e-83 | M1 macrophages | ARFGAP3 |
| 1.48814457214171e-87 | 0.529424453161209 | 0.411 | 0.275 | 5.36877917291564e-83 | M1 macrophages | FERMT2 |
| 1.55181066739474e-87 | 0.92541298338278 | 0.254 | 0.145 | 5.59846734476001e-83 | M1 macrophages | ARID5A |
| 4.16084472972721e-87 | 0.425738992050347 | 0.627 | 0.477 | 1.50110795314368e-82 | M1 macrophages | USP15 |
| 4.69932051096446e-87 | 0.907020671326586 | 0.259 | 0.147 | 1.69537386074065e-82 | M1 macrophages | USP53 |
| 4.79509580865222e-87 | 0.60616552916008 | 0.436 | 0.305 | 1.72992671488746e-82 | M1 macrophages | HSPA1B |
| 6.23558929770893e-87 | 0.580301552074598 | 0.591 | 0.453 | 2.24961355093445e-82 | M1 macrophages | NRIP1 |
| 7.42506270374979e-87 | 0.322332505684141 | 0.508 | 0.365 | 2.67873987163181e-82 | M1 macrophages | BTG2 |
| 1.39770259122008e-86 | 0.321093270123182 | 0.878 | 0.778 | 5.04249163834469e-82 | M1 macrophages | CTSS |
| 1.40259952604943e-86 | 0.472138040514952 | 0.536 | 0.393 | 5.06015831012852e-82 | M1 macrophages | OGDH |
| 1.71949676343863e-86 | 0.543210044511401 | 0.63 | 0.501 | 6.20342847345754e-82 | M1 macrophages | CD55 |
| 2.08745677568872e-86 | -1.261145254 | 0.32 | 0.435 | 7.53091780965219e-82 | M1 macrophages | ST6GAL1 |
| 4.24114945424221e-86 | 0.311241554484145 | 0.811 | 0.654 | 1.53007948860696e-81 | M1 macrophages | TYMP |
| 5.27338783629591e-86 | 0.32099740645321 | 0.761 | 0.62 | 1.90248012970048e-81 | M1 macrophages | FPR3 |
| 6.25147754775352e-86 | 0.363366533916744 | 0.637 | 0.504 | 2.25534555490304e-81 | M1 macrophages | RPS20 |
| 7.66882188187894e-86 | -0.626836121 | 0.532 | 0.611 | 2.76668087032547e-81 | M1 macrophages | RNASET2 |
| 1.57552550955155e-85 | 0.647972893058071 | 0.357 | 0.232 | 5.68402338080913e-81 | M1 macrophages | ACTN1 |
| 2.2907734691518e-85 | 0.839283238126298 | 0.298 | 0.179 | 8.26442344465896e-81 | M1 macrophages | ACVR2A |
| 2.39673061711285e-85 | 0.54322590623653 | 0.329 | 0.199 | 8.64668504735804e-81 | M1 macrophages | SPRED2 |
| 5.61450148219697e-85 | 0.699192220454665 | 0.354 | 0.23 | 2.0255436997322e-80 | M1 macrophages | ABCG1 |
| 3.44368495062417e-84 | 1.04022409301935 | 0.287 | 0.175 | 1.24237821963668e-79 | M1 macrophages | ZNF331 |
| 6.28192585796791e-84 | 0.524580184716574 | 0.477 | 0.347 | 2.26633039177908e-79 | M1 macrophages | P2RX4 |
| 9.50421861884014e-84 | -1.135348913 | 0.363 | 0.469 | 3.42883695111896e-79 | M1 macrophages | FLI1 |
| 2.97730128071553e-83 | 1.08086645450942 | 0.294 | 0.179 | 1.07412098304374e-78 | M1 macrophages | CRADD |
| 3.31391987297244e-83 | 0.3217461857037 | 0.768 | 0.635 | 1.19556287257227e-78 | M1 macrophages | RPS11 |
| 5.13903409277528e-83 | 0.76826814351174 | 0.262 | 0.151 | 1.85400932965054e-78 | M1 macrophages | VPS37B |
| 1.40827104739707e-82 | 0.352215733741399 | 0.647 | 0.514 | 5.08061945769442e-78 | M1 macrophages | MYO9B |
| 1.53363082947056e-82 | -1.374151526 | 0.274 | 0.389 | 5.53287994348095e-78 | M1 macrophages | RGL1 |
| 2.489330485643e-81 | 0.394593925760935 | 0.513 | 0.367 | 8.98075759305427e-77 | M1 macrophages | PPP6R3 |
| 4.07800099680297e-81 | 0.594655419968283 | 0.314 | 0.192 | 1.47122041961661e-76 | M1 macrophages | GAS2L3 |
| 8.65249230907006e-81 | 0.304505454373265 | 0.725 | 0.589 | 3.1215596503432e-76 | M1 macrophages | RPL27A |
| 8.88518584361491e-81 | -0.82212815 | 0.475 | 0.571 | 3.20550849680095e-76 | M1 macrophages | CSF1R |
| 2.33784564443037e-80 | 0.429058339967328 | 0.572 | 0.434 | 8.43424573141145e-76 | M1 macrophages | LTA4H |
| 2.53675403603395e-80 | 0.395578737308702 | 0.702 | 0.579 | 9.15184753579968e-76 | M1 macrophages | RAB10 |
| 7.31090542846079e-80 | 0.409413898803909 | 0.515 | 0.377 | 2.6375553514258e-75 | M1 macrophages | COPA |
| 9.76314951372155e-80 | -1.36499884 | 0.187 | 0.302 | 3.52225145006532e-75 | M1 macrophages | APPL2 |
| 1.06620489122845e-79 | 0.563788369394406 | 0.337 | 0.216 | 3.8465473860849e-75 | M1 macrophages | IPO7 |
| 1.73711115805459e-79 | -0.601667535 | 0.677 | 0.724 | 6.26697592491356e-75 | M1 macrophages | LILRB5 |
| 3.89397405334661e-79 | 0.579499545337522 | 0.324 | 0.204 | 1.40482901922586e-74 | M1 macrophages | HIPK2 |
| 4.05125367940539e-79 | 0.417965931864288 | 0.479 | 0.339 | 1.46157078991908e-74 | M1 macrophages | ESYT2 |
| 4.94076809298139e-79 | 0.321535609325151 | 0.714 | 0.586 | 1.78248090490489e-74 | M1 macrophages | CD81 |
| 8.3890017485038e-79 | 0.566041378150816 | 0.313 | 0.192 | 3.02650016080772e-74 | M1 macrophages | MAST2 |
| 1.48556856583048e-78 | 0.47517699611081 | 0.483 | 0.351 | 5.35948571494663e-74 | M1 macrophages | ANKRD10 |
| 8.17599580727076e-78 | 0.505282795582227 | 0.427 | 0.3 | 2.94965400738907e-73 | M1 macrophages | TBC1D1 |
| 1.06318433444523e-77 | 0.395413527213344 | 0.549 | 0.409 | 3.83565012337807e-73 | M1 macrophages | ERBIN |
| 1.89663988405555e-77 | 0.332394108455755 | 0.61 | 0.455 | 6.8425077097072e-73 | M1 macrophages | GAB2 |
| 2.14156170751386e-77 | 0.443040423568411 | 0.4 | 0.268 | 7.72611217219775e-73 | M1 macrophages | RCOR1 |
| 2.50909801915627e-77 | 0.694930918046035 | 0.442 | 0.321 | 9.05207292371009e-73 | M1 macrophages | PDE3A |
| 3.03924742907966e-77 | 0.375504163329207 | 0.486 | 0.344 | 1.09646929498907e-72 | M1 macrophages | PHF20 |
| 4.35990617360517e-77 | -0.96732554 | 0.236 | 0.355 | 1.57292335025154e-72 | M1 macrophages | GPR34 |
| 4.42569817775761e-77 | 0.44321573180887 | 0.535 | 0.398 | 1.59665913158961e-72 | M1 macrophages | EMILIN2 |
| 4.64516049011435e-77 | 0.704549249964274 | 0.269 | 0.161 | 1.67583455001856e-72 | M1 macrophages | PLK3 |
| 4.65051250116118e-77 | 0.637705787520108 | 0.607 | 0.476 | 1.67776539504392e-72 | M1 macrophages | DMXL2 |
| 6.91726868417201e-77 | 0.467102952240718 | 0.527 | 0.387 | 2.49554302318874e-72 | M1 macrophages | CCNH |
| 1.17785009236446e-76 | 0.455055387551624 | 0.329 | 0.208 | 4.24932977822327e-72 | M1 macrophages | RASA3 |
| 1.47199950928079e-76 | 0.373988185080528 | 0.446 | 0.311 | 5.31053262963229e-72 | M1 macrophages | UBE2R2 |
| 3.10006580863875e-76 | 0.521216479778842 | 0.58 | 0.46 | 1.1184107417826e-71 | M1 macrophages | PRNP |
| 5.98997832626836e-76 | -1.100375105 | 0.255 | 0.373 | 2.16100448076784e-71 | M1 macrophages | SCARB1 |
| 1.12596824169337e-75 | 0.82964194181771 | 0.259 | 0.155 | 4.06215562555716e-71 | M1 macrophages | TES |
| 1.79825580818593e-74 | -1.309868573 | 0.157 | 0.268 | 6.48756747919237e-70 | M1 macrophages | GABRB2 |
| 2.11923057815053e-74 | 0.620120053922352 | 0.252 | 0.143 | 7.64554815679366e-70 | M1 macrophages | SLC27A4 |
| 1.78623431060084e-73 | -1.077074549 | 0.216 | 0.331 | 6.44419752235465e-69 | M1 macrophages | TRIM14 |
| 2.7552065530374e-73 | 0.466618710290421 | 0.679 | 0.56 | 9.93995868139302e-69 | M1 macrophages | HSPB1 |
| 2.93936947081182e-73 | 0.388788459764641 | 0.505 | 0.361 | 1.06043632398478e-68 | M1 macrophages | KLF7 |
| 3.76149004005183e-73 | 0.351662370317009 | 0.651 | 0.525 | 1.3570327617495e-68 | M1 macrophages | KCTD12 |
| 4.67625576730789e-73 | 0.468097131373891 | 0.503 | 0.368 | 1.68705279317167e-68 | M1 macrophages | ARHGAP31 |
| 5.84506587647342e-71 | 0.503412115775206 | 0.336 | 0.214 | 2.10872441625531e-66 | M1 macrophages | VCAN |
| 7.73376473405125e-71 | -1.299735585 | 0.422 | 0.499 | 2.79011030310367e-66 | M1 macrophages | PDGFC |
| 9.2175709717812e-71 | 0.458741846810747 | 0.486 | 0.362 | 3.3254230794895e-66 | M1 macrophages | EFHD2 |
| 1.03632748088772e-70 | -0.781655369 | 0.443 | 0.537 | 3.73875865279863e-66 | M1 macrophages | BLVRB |
| 2.27311565520872e-70 | 0.715909108203953 | 0.281 | 0.178 | 8.2007193492965e-66 | M1 macrophages | RILPL2 |
| 2.3353370192151e-70 | -1.004043288 | 0.242 | 0.356 | 8.4251953642223e-66 | M1 macrophages | CHID1 |
| 4.14070466589518e-70 | -0.5356919 | 0.729 | 0.762 | 1.493842022315e-65 | M1 macrophages | ARHGAP18 |
| 2.04729409621289e-69 | 0.561553548812825 | 0.32 | 0.206 | 7.38602291090723e-65 | M1 macrophages | E2F3 |
| 4.05864914520323e-69 | 0.471718386891985 | 0.397 | 0.274 | 1.46423885211497e-64 | M1 macrophages | CTTNBP2NL |
| 4.92648028459234e-69 | 0.595696911686024 | 0.375 | 0.262 | 1.77732629227238e-64 | M1 macrophages | SLC43A3 |
| 9.33832142367264e-69 | -0.77598883 | 0.797 | 0.74 | 3.36898622001838e-64 | M1 macrophages | PLTP |
| 9.85096986950657e-69 | 0.440779568935343 | 0.528 | 0.401 | 3.55393439982189e-64 | M1 macrophages | PITPNA |
| 1.66688289539991e-68 | 0.440066348519606 | 0.455 | 0.324 | 6.01361342173424e-64 | M1 macrophages | RAPGEF2 |
| 3.33968454357241e-68 | -1.042895181 | 0.274 | 0.385 | 1.20485799278462e-63 | M1 macrophages | RASSF4 |
| 4.32074668647993e-68 | 0.85290359205549 | 0.353 | 0.245 | 1.55879578208137e-63 | M1 macrophages | ACSL4 |
| 5.45267703572116e-68 | 0.783205571603033 | 0.362 | 0.256 | 1.96716229417712e-63 | M1 macrophages | RALA |
| 6.37141045563695e-68 | 0.303942407115686 | 0.737 | 0.611 | 2.29861375008014e-63 | M1 macrophages | HSP90AB1 |
| 6.78330795636501e-68 | 0.544303596627188 | 0.266 | 0.161 | 2.4472140114178e-63 | M1 macrophages | AGO2 |
| 6.8458261657651e-68 | 0.567013686347226 | 0.335 | 0.222 | 2.46976870582307e-63 | M1 macrophages | DDHD1 |
| 1.01320526758509e-67 | 0.387853940984151 | 0.578 | 0.434 | 3.65534064386673e-63 | M1 macrophages | ATF6 |
| 1.44731334541169e-67 | 0.478254798605476 | 0.419 | 0.296 | 5.22147235624175e-63 | M1 macrophages | LCP1 |
| 1.49864281200946e-67 | 0.533008773062484 | 0.386 | 0.271 | 5.40665367288652e-63 | M1 macrophages | TUBB4B |
| 1.91114513634897e-67 | -0.710833362 | 0.453 | 0.54 | 6.89483830840616e-63 | M1 macrophages | CD302 |
| 2.87094167313917e-67 | 0.468029404894841 | 0.501 | 0.385 | 1.03574962741842e-62 | M1 macrophages | YWHAQ |
| 1.6988407237381e-66 | -0.822467509 | 0.425 | 0.5 | 6.12890767902995e-62 | M1 macrophages | ALOX5AP |
| 1.87055136492471e-66 | 0.321970289278093 | 0.615 | 0.483 | 6.74838815923889e-62 | M1 macrophages | EIF4G2 |
| 3.528044524398e-66 | -0.980065114 | 0.151 | 0.257 | 1.27281262306707e-61 | M1 macrophages | TMEM37 |
| 4.8824235649353e-66 | 0.321699497055619 | 0.618 | 0.499 | 1.76143194952171e-61 | M1 macrophages | TMEM165 |
| 1.47209339974992e-65 | 0.494330135710241 | 0.408 | 0.294 | 5.31087135827778e-61 | M1 macrophages | ATP6V1B2 |
| 4.06155299988389e-65 | 0.450826167321988 | 0.517 | 0.404 | 1.46528647576811e-60 | M1 macrophages | RNF145 |
| 6.16448573509399e-65 | 1.12506437073024 | 0.43 | 0.33 | 2.22396151864986e-60 | M1 macrophages | SARNP |
| 1.24549160728266e-64 | 0.393142373758449 | 0.439 | 0.312 | 4.49336007159364e-60 | M1 macrophages | TAB2 |
| 4.25630431696572e-64 | 0.532789431203703 | 0.397 | 0.287 | 1.53554690843172e-59 | M1 macrophages | CLINT1 |
| 4.72273056818416e-64 | 0.504718531893589 | 0.275 | 0.172 | 1.7038195070838e-59 | M1 macrophages | UBAP2 |
| 5.8857018395047e-64 | -1.046738651 | 0.545 | 0.593 | 2.12338465263811e-59 | M1 macrophages | DNAAF9 |
| 1.44063037284203e-63 | -1.180594062 | 0.171 | 0.273 | 5.19736219610219e-59 | M1 macrophages | WLS |
| 1.96333443870887e-63 | 0.512119369752859 | 0.261 | 0.158 | 7.08312165453e-59 | M1 macrophages | PLEKHG2 |
| 1.96384811715371e-63 | -0.966404076 | 0.217 | 0.324 | 7.08497485225545e-59 | M1 macrophages | LTC4S |
| 2.00451683535609e-63 | 0.535984373184265 | 0.371 | 0.256 | 7.23169538691417e-59 | M1 macrophages | HYCC1 |
| 5.57642783105682e-63 | -0.725503166 | 0.584 | 0.652 | 2.01180786861037e-58 | M1 macrophages | HTRA1 |
| 7.76284785711517e-63 | 0.317228169393667 | 0.477 | 0.343 | 2.80060262141144e-58 | M1 macrophages | GLS |
| 8.22467338247691e-63 | -1.132565419 | 0.302 | 0.396 | 2.9672154161962e-58 | M1 macrophages | INPP5D |
| 1.00881982764322e-62 | -0.827348366 | 0.321 | 0.423 | 3.63951929218846e-58 | M1 macrophages | GYPC |
| 1.56179681578071e-62 | -1.262582432 | 0.163 | 0.262 | 5.63449437229207e-58 | M1 macrophages | CCDC170 |
| 2.22805326118255e-62 | 0.54572895161271 | 0.284 | 0.182 | 8.03814775036828e-58 | M1 macrophages | TBK1 |
| 4.42162475130639e-62 | -1.652325696 | 0.192 | 0.28 | 1.59518956152881e-57 | M1 macrophages | SOX5 |
| 1.22499159329994e-61 | 0.468367008850688 | 0.43 | 0.315 | 4.41940217114818e-57 | M1 macrophages | CD9 |
| 1.6010332321369e-61 | 0.397766067735091 | 0.446 | 0.322 | 5.77604759158029e-57 | M1 macrophages | TREM1 |
| 2.89819008433958e-61 | 0.436856405544786 | 0.577 | 0.47 | 1.04558003672719e-56 | M1 macrophages | NRP2 |
| 3.77321553569013e-61 | 0.472941493263703 | 0.306 | 0.2 | 1.36126296881093e-56 | M1 macrophages | AHCYL1 |
| 4.16889523631344e-61 | -1.126271278 | 0.185 | 0.285 | 1.5040123344048e-56 | M1 macrophages | BLNK |
| 4.19894320364521e-61 | 0.65817487780153 | 0.481 | 0.37 | 1.51485273957908e-56 | M1 macrophages | MT2A |
| 5.69004429756961e-61 | -0.59857952 | 0.864 | 0.789 | 2.05279728123419e-56 | M1 macrophages | C1QB |
| 1.23444609018806e-60 | -0.967592661 | 0.348 | 0.443 | 4.45351115957148e-56 | M1 macrophages | MGAT4A |
| 1.62484888728689e-60 | 0.308515203989915 | 0.669 | 0.548 | 5.8619673306649e-56 | M1 macrophages | CD84 |
| 1.6805402042584e-60 | 0.802287474610872 | 0.326 | 0.221 | 6.06288489490304e-56 | M1 macrophages | GLIS3 |
| 3.55799550838203e-60 | -1.266378292 | 0.255 | 0.346 | 1.28361803955899e-55 | M1 macrophages | FCGR2C |
| 4.18803033160837e-60 | 0.543660104606278 | 0.539 | 0.428 | 1.51091570273435e-55 | M1 macrophages | ZNF438 |
| 4.42737682362708e-60 | 0.314430197622573 | 0.922 | 0.852 | 1.59726473665994e-55 | M1 macrophages | ZEB2 |
| 5.12661120575156e-60 | 0.442683870968539 | 0.465 | 0.354 | 1.84952752469899e-55 | M1 macrophages | CHMP4B |
| 1.48861376883974e-59 | -1.167306849 | 0.155 | 0.251 | 5.37047189384315e-55 | M1 macrophages | NAIP |
| 1.49966705925519e-59 | 0.376668479162129 | 0.429 | 0.307 | 5.41034884967495e-55 | M1 macrophages | STRN3 |
| 6.22443889563026e-59 | 0.421478257026232 | 0.325 | 0.217 | 2.24559082037653e-54 | M1 macrophages | RAB21 |
| 6.55597904291534e-59 | 0.401074691188606 | 0.42 | 0.3 | 2.36520055931257e-54 | M1 macrophages | LCOR |
| 7.76398018735953e-59 | -0.970723407 | 0.622 | 0.644 | 2.8010111321937e-54 | M1 macrophages | SLC9A9 |
| 8.37251116180771e-59 | 0.645414265981491 | 0.294 | 0.195 | 3.02055085184537e-54 | M1 macrophages | SASH1 |
| 1.63245610378292e-58 | 0.431813725469289 | 0.474 | 0.366 | 5.88941188561764e-54 | M1 macrophages | ARF6 |
| 2.22346552314753e-58 | 0.505069810005345 | 0.334 | 0.229 | 8.02159656785933e-54 | M1 macrophages | IRF1 |
| 4.95601256263976e-58 | 0.367350998563353 | 0.486 | 0.369 | 1.78798065222355e-53 | M1 macrophages | CLEC7A |
| 8.7830122089494e-58 | 0.353987872586814 | 0.542 | 0.429 | 3.16864731462268e-53 | M1 macrophages | CORO1C |
| 1.66514914748517e-57 | -0.638552216 | 0.473 | 0.551 | 6.00735857938224e-53 | M1 macrophages | CPVL |
| 1.76425174825761e-57 | 0.549095134918843 | 0.315 | 0.212 | 6.36489103218897e-53 | M1 macrophages | GPD2 |
| 2.79799244344655e-57 | 0.634877139326967 | 0.301 | 0.203 | 1.00943173382221e-52 | M1 macrophages | ZFP91 |
| 3.68791537990245e-57 | 0.305395045062619 | 0.473 | 0.341 | 1.33048923160741e-52 | M1 macrophages | SLCO3A1 |
| 3.94127493333481e-57 | 0.318378454472369 | 0.389 | 0.276 | 1.4218937576992e-52 | M1 macrophages | UBE2H |
| 4.30729101313895e-57 | 0.35056256323802 | 0.341 | 0.232 | 1.55394137881014e-52 | M1 macrophages | IL6ST |
| 4.49061323019496e-57 | -1.16338147 | 0.166 | 0.26 | 1.62007853505743e-52 | M1 macrophages | FGD2 |
| 7.73426610926649e-57 | -1.029662946 | 0.173 | 0.271 | 2.79029118424007e-52 | M1 macrophages | IGFBP4 |
| 1.68016028048805e-56 | 0.62142725520878 | 0.276 | 0.183 | 6.06151424391675e-52 | M1 macrophages | ARL4A |
| 2.85977399360014e-56 | 0.330099399135932 | 0.435 | 0.321 | 1.03172066367112e-51 | M1 macrophages | PPP1CB |
| 3.30370449979874e-56 | 0.649245562879541 | 0.34 | 0.241 | 1.19187747239239e-51 | M1 macrophages | BASP1 |
| 3.4440024179585e-56 | 0.328865372297867 | 0.381 | 0.262 | 1.24249275232689e-51 | M1 macrophages | PHACTR1 |
| 4.75480855977431e-56 | -0.770416666 | 0.284 | 0.389 | 1.71539228410978e-51 | M1 macrophages | HPGDS |
| 5.26073267371598e-56 | 0.394972025634913 | 0.531 | 0.415 | 1.89791452669651e-51 | M1 macrophages | PTPN12 |
| 9.90857528547418e-56 | 0.489301346852645 | 0.28 | 0.182 | 3.57471670574052e-51 | M1 macrophages | ATP11C |
| 1.84008256906413e-55 | -0.73040065 | 0.331 | 0.427 | 6.63846588441267e-51 | M1 macrophages | OTULINL |
| 2.59170722416115e-55 | -0.942304406 | 0.373 | 0.459 | 9.35010215260617e-51 | M1 macrophages | CTSC |
| 4.05794810009338e-55 | -0.500544442 | 0.762 | 0.734 | 1.46398593607069e-50 | M1 macrophages | CD99 |
| 6.26258738960556e-55 | -0.989247652 | 0.311 | 0.404 | 2.259353652548e-50 | M1 macrophages | ENTPD1 |
| 1.79496093466267e-54 | 0.344466406807697 | 0.366 | 0.253 | 6.4756805639825e-50 | M1 macrophages | ADNP |
| 4.69600228659034e-54 | -0.841563008 | 0.428 | 0.508 | 1.6941767449332e-49 | M1 macrophages | RIN2 |
| 7.77945361799956e-54 | 0.346534830532949 | 0.504 | 0.392 | 2.8065934817657e-49 | M1 macrophages | SIRPA |
| 9.9222491801977e-54 | 0.336756127697818 | 0.314 | 0.212 | 3.57964983673992e-49 | M1 macrophages | IL4R |
| 1.77863975570224e-53 | 0.562870818836024 | 0.312 | 0.217 | 6.41679864664696e-49 | M1 macrophages | FPR1 |
| 1.8478008341272e-53 | -0.829042383 | 0.326 | 0.409 | 6.66631106928071e-49 | M1 macrophages | FUCA1 |
| 3.73429731205617e-53 | 0.511943597803793 | 0.599 | 0.475 | 1.34722244127051e-48 | M1 macrophages | FMN1 |
| 5.95426966174295e-53 | -1.398910119 | 0.174 | 0.26 | 2.148121865867e-48 | M1 macrophages | EPB41L1 |
| 1.00140076025322e-52 | 0.374524416709869 | 0.27 | 0.174 | 3.61275352276554e-48 | M1 macrophages | CHASERR |
| 1.45358657858046e-52 | 0.332417950637437 | 0.732 | 0.63 | 5.24410429954472e-48 | M1 macrophages | LYN |
| 1.57455189408229e-52 | -0.688767048 | 0.405 | 0.479 | 5.68051086828067e-48 | M1 macrophages | CREG1 |
| 2.53755139142707e-52 | -0.874763844 | 0.478 | 0.535 | 9.15472415485144e-48 | M1 macrophages | EPS15 |
| 3.62441211719057e-52 | 0.508034477050065 | 0.288 | 0.196 | 1.30757915951884e-47 | M1 macrophages | PDLIM7 |
| 8.0081191614332e-52 | -0.775965008 | 0.606 | 0.65 | 2.88908914987025e-47 | M1 macrophages | MEF2C |
| 8.35711073599963e-52 | 0.348204657339911 | 0.729 | 0.613 | 3.01499484022659e-47 | M1 macrophages | SLC8A1 |
| 9.37702531676154e-52 | 0.49422204774056 | 0.368 | 0.261 | 3.38294942352806e-47 | M1 macrophages | GBE1 |
| 1.52186654305516e-51 | 0.347040064003211 | 0.262 | 0.167 | 5.49043792738009e-47 | M1 macrophages | ERN1 |
| 6.42374517997762e-51 | -0.449101272 | 0.759 | 0.749 | 2.31749454858053e-46 | M1 macrophages | CALM2 |
| 8.77492328030956e-51 | 0.336676682482455 | 0.424 | 0.318 | 3.16572907183728e-46 | M1 macrophages | ACTN4 |
| 2.5276104478351e-50 | 0.418132831896398 | 0.335 | 0.236 | 9.11886021265469e-46 | M1 macrophages | NAA50 |
| 4.54203573217443e-50 | 0.357780491339928 | 0.452 | 0.346 | 1.63863023109657e-45 | M1 macrophages | ADA2 |
| 1.36981668982877e-49 | 0.452534675452068 | 0.302 | 0.207 | 4.94188767189526e-45 | M1 macrophages | PER1 |
| 4.5824017393247e-49 | -1.286819217 | 0.179 | 0.263 | 1.65319307549617e-44 | M1 macrophages | GCNT1 |
| 5.66484504673631e-49 | 0.340014515103073 | 0.398 | 0.296 | 2.04370614751106e-44 | M1 macrophages | EIF1AX |
| 7.05264664717267e-49 | 0.509350077077537 | 0.379 | 0.286 | 2.54438333090049e-44 | M1 macrophages | CYCS |
| 1.23275268757279e-48 | -0.803878618 | 0.218 | 0.312 | 4.44740187095635e-44 | M1 macrophages | RENBP |
| 2.12299567957569e-48 | -0.74515827 | 0.299 | 0.393 | 7.65913151320523e-44 | M1 macrophages | CRYL1 |
| 2.56733860794963e-48 | -1.1606852 | 0.166 | 0.253 | 9.26218749589987e-44 | M1 macrophages | RCN3 |
| 7.11125577636141e-48 | 0.429636861533298 | 0.431 | 0.324 | 2.56552774643791e-43 | M1 macrophages | SUSD6 |
| 8.23018286964775e-48 | -0.652445473 | 0.611 | 0.654 | 2.96920307388282e-43 | M1 macrophages | SYK |
| 8.61918564054495e-48 | 0.390588515516905 | 0.331 | 0.235 | 3.1095436035394e-43 | M1 macrophages | ZNF800 |
| 1.3157469028284e-47 | -0.921245487 | 0.21 | 0.299 | 4.74682010133401e-43 | M1 macrophages | ARHGAP25 |
| 7.96387029982599e-47 | 0.526426193362233 | 0.357 | 0.263 | 2.87312548806822e-42 | M1 macrophages | ACSL3 |
| 8.57682119438609e-47 | 0.707574986182329 | 0.341 | 0.253 | 3.09425978229867e-42 | M1 macrophages | TALAM1 |
| 1.07041598086205e-46 | 0.317636596321653 | 0.502 | 0.399 | 3.86173973415602e-42 | M1 macrophages | CAPN2 |
| 3.85587823052435e-46 | 0.363977465238145 | 0.332 | 0.237 | 1.39108518922627e-41 | M1 macrophages | IRF2BP2 |
| 4.0057925813476e-46 | -0.717046786 | 0.358 | 0.439 | 1.44516978957277e-41 | M1 macrophages | TNFRSF1A |
| 4.45247912455131e-46 | 0.482892408126643 | 0.311 | 0.222 | 1.60632089376437e-41 | M1 macrophages | IRAK1 |
| 5.7860810954087e-46 | -0.734569341 | 0.426 | 0.49 | 2.08744447679059e-41 | M1 macrophages | PEPD |
| 8.46882613028869e-46 | -1.033252634 | 0.246 | 0.33 | 3.05529840302425e-41 | M1 macrophages | SLC2A9 |
| 1.64545702558956e-45 | -0.659028852 | 0.409 | 0.48 | 5.93631531121946e-41 | M1 macrophages | TSPAN4 |
| 2.187746198805e-45 | 0.444086433106999 | 0.304 | 0.213 | 7.89273196142881e-41 | M1 macrophages | RNF19A |
| 2.40688416372892e-45 | 0.349870168915902 | 0.32 | 0.225 | 8.68331599748483e-41 | M1 macrophages | DOCK5 |
| 2.8955534024524e-45 | -0.867676522 | 0.281 | 0.364 | 1.04462880100275e-40 | M1 macrophages | FCGR2B |
| 3.38523845781665e-45 | 0.42733568031386 | 0.446 | 0.34 | 1.22129247842651e-40 | M1 macrophages | GSAP |
| 4.43314341310298e-45 | 0.415980474200544 | 0.281 | 0.193 | 1.59934514914516e-40 | M1 macrophages | DNM1L |
| 9.8856215003709e-45 | 0.394330548266306 | 0.345 | 0.25 | 3.56643566868881e-40 | M1 macrophages | DEFB1 |
| 1.11065403950179e-44 | 0.374553214465454 | 0.293 | 0.202 | 4.00690657831061e-40 | M1 macrophages | SLC30A7 |
| 1.12688499888138e-44 | 0.360762341077568 | 0.309 | 0.217 | 4.06546301046435e-40 | M1 macrophages | ZNFX1 |
| 1.85504753638264e-44 | 0.312211241810762 | 0.584 | 0.464 | 6.69245499700766e-40 | M1 macrophages | DIAPH2 |
| 3.92154118075751e-44 | 0.332772753685086 | 0.603 | 0.5 | 1.41477441178189e-39 | M1 macrophages | TPM4 |
| 9.06470899528457e-44 | 0.324538792471837 | 0.664 | 0.562 | 3.27027506422881e-39 | M1 macrophages | SLC43A2 |
| 1.31207517797811e-43 | 0.415318106396123 | 0.278 | 0.188 | 4.73357361959161e-39 | M1 macrophages | DGKH |
| 2.16038367972157e-43 | -0.890621143 | 0.318 | 0.398 | 7.79401620133151e-39 | M1 macrophages | ENSG00000234147 |
| 2.43846131496947e-43 | 0.308981393772266 | 0.328 | 0.232 | 8.79723688601534e-39 | M1 macrophages | MCU |
| 5.10262072076214e-43 | -0.469954913 | 0.725 | 0.731 | 1.84087247742936e-38 | M1 macrophages | LRP1 |
| 6.94001233897344e-43 | 0.373727155390862 | 0.314 | 0.226 | 2.50374825153145e-38 | M1 macrophages | SERTAD1 |
| 7.93804158530342e-43 | 0.791078973399264 | 0.254 | 0.175 | 2.86380726272991e-38 | M1 macrophages | ZNF277 |
| 9.29220286723314e-43 | 0.323921507543646 | 0.369 | 0.269 | 3.3523480284117e-38 | M1 macrophages | XPR1 |
| 1.59447279719464e-42 | -0.589299766 | 0.503 | 0.567 | 5.75237951043911e-38 | M1 macrophages | MARCHF1 |
| 1.8577963715905e-42 | 0.446273529790904 | 0.312 | 0.228 | 6.70237196978705e-38 | M1 macrophages | PNRC2 |
| 6.81004084541098e-42 | 0.355698078545489 | 0.345 | 0.253 | 2.45685843579892e-37 | M1 macrophages | MAP3K20 |
| 7.55218696854887e-42 | 0.377616745008109 | 0.683 | 0.579 | 2.72460249264337e-37 | M1 macrophages | DAPK1 |
| 1.02479849289802e-41 | -0.954177278 | 0.29 | 0.366 | 3.69716552282818e-37 | M1 macrophages | ATRN |
| 1.93538151394508e-41 | 0.616030632242652 | 0.31 | 0.228 | 6.98227588785966e-37 | M1 macrophages | SH3BP5 |
| 2.56894887099827e-41 | -0.870004904 | 0.238 | 0.316 | 9.26799684190045e-37 | M1 macrophages | CTSK |
| 6.24784143304984e-41 | -0.614068322 | 0.345 | 0.434 | 2.25403375380139e-36 | M1 macrophages | SNCA |
| 7.68269742894391e-41 | -1.007914474 | 0.33 | 0.395 | 2.77168675144009e-36 | M1 macrophages | ATP8B4 |
| 1.37389607632359e-40 | -0.79592792 | 0.23 | 0.31 | 4.95660487455262e-36 | M1 macrophages | GLMP |
| 1.37654683148227e-40 | 0.668512642332256 | 0.469 | 0.396 | 4.9661680039386e-36 | M1 macrophages | IL18 |
| 1.52209462359437e-40 | 0.454108825528067 | 0.298 | 0.217 | 5.49126077354141e-36 | M1 macrophages | RAB20 |
| 2.06952822135434e-40 | 0.327615174709824 | 0.406 | 0.314 | 7.46623696418006e-36 | M1 macrophages | SLC15A3 |
| 2.24795949935465e-40 | 0.329718028557964 | 0.715 | 0.629 | 8.10996348582178e-36 | M1 macrophages | RNF13 |
| 1.25201901043859e-39 | 0.323090826908899 | 0.27 | 0.188 | 4.51690898395931e-35 | M1 macrophages | ERGIC1 |
| 1.29370794056009e-39 | -0.528576304 | 0.431 | 0.505 | 4.66731013715863e-35 | M1 macrophages | OSTF1 |
| 4.55753282327597e-39 | 0.305957991778093 | 0.437 | 0.346 | 1.64422111665327e-34 | M1 macrophages | LINC00963 |
| 7.30737896204753e-39 | -0.699852653 | 0.559 | 0.599 | 2.63628310813789e-34 | M1 macrophages | RNF213 |
| 9.98569916230172e-39 | 0.417435266768172 | 0.443 | 0.346 | 3.60254068678359e-34 | M1 macrophages | CBLB |
| 1.01575618194823e-38 | 0.36971315839581 | 0.26 | 0.179 | 3.66454357761464e-34 | M1 macrophages | DNMBP |
| 1.82296569031642e-38 | -0.866587054 | 0.244 | 0.323 | 6.57671332095455e-34 | M1 macrophages | CYTH4 |
| 2.12788430687562e-38 | 0.314676863227735 | 0.349 | 0.26 | 7.67676821391517e-34 | M1 macrophages | SLC6A6 |
| 2.58287503802755e-38 | 0.325313693096255 | 0.322 | 0.234 | 9.31823827469199e-34 | M1 macrophages | VCL |
| 3.1140527361354e-38 | 0.361466517575544 | 0.258 | 0.181 | 1.12345680561557e-33 | M1 macrophages | SUN2 |
| 4.7079970953259e-38 | -0.426786736 | 0.742 | 0.725 | 1.69850411208073e-33 | M1 macrophages | VSIG4 |
| 5.04051652661848e-38 | -0.625046415 | 0.406 | 0.481 | 1.81846714730815e-33 | M1 macrophages | CMTM7 |
| 8.23386418193677e-38 | 0.414530195225643 | 0.306 | 0.226 | 2.97053118091733e-33 | M1 macrophages | PSMB4 |
| 3.99133070426958e-37 | 0.372071088883852 | 0.323 | 0.239 | 1.43995237817934e-32 | M1 macrophages | CCR1 |
| 6.04602736214349e-37 | -0.635008254 | 0.278 | 0.361 | 2.18122529144051e-32 | M1 macrophages | BLVRA |
| 7.9709329174339e-37 | 0.471045578248275 | 0.256 | 0.183 | 2.87567346862263e-32 | M1 macrophages | SEC22B |
| 8.2758600158252e-37 | 0.423687186003161 | 0.386 | 0.307 | 2.98568201790926e-32 | M1 macrophages | SELENOK |
| 1.06270872920881e-36 | -0.406092433 | 0.336 | 0.438 | 3.83393428236662e-32 | M1 macrophages | ENSG00000280441 |
| 1.46561864836162e-36 | 0.380118212990776 | 0.301 | 0.22 | 5.2875123976942e-32 | M1 macrophages | KLF10 |
| 1.88647080775386e-36 | 0.330672129133237 | 0.393 | 0.31 | 6.80582073313359e-32 | M1 macrophages | LACTB |
| 2.73659394628901e-36 | -0.850549799 | 0.204 | 0.282 | 9.87280998002686e-32 | M1 macrophages | PARL |
| 7.14196395690963e-36 | 0.304672424006881 | 0.475 | 0.385 | 2.57660633673429e-31 | M1 macrophages | ZFAS1 |
| 9.29255675260207e-36 | -0.908506535 | 0.228 | 0.3 | 3.35247569963625e-31 | M1 macrophages | MAP3K1 |
| 1.05650112729316e-35 | -0.612089733 | 0.39 | 0.464 | 3.81153911693553e-31 | M1 macrophages | STX7 |
| 1.40866156106563e-35 | -0.878774561 | 0.375 | 0.437 | 5.08202831385647e-31 | M1 macrophages | IQGAP2 |
| 1.99137013381565e-35 | -0.895721344 | 0.222 | 0.295 | 7.18426603176671e-31 | M1 macrophages | SPTLC2 |
| 2.94234849616231e-35 | 0.327128306915231 | 0.392 | 0.301 | 1.06151106696048e-30 | M1 macrophages | RUFY3 |
| 3.42978037665403e-35 | 0.372511766882181 | 0.297 | 0.218 | 1.23736186648547e-30 | M1 macrophages | UBAP2L |
| 4.98978744266524e-35 | -0.895314145 | 0.279 | 0.347 | 1.80016561569034e-30 | M1 macrophages | PER3 |
| 5.36346816553262e-35 | 0.317062032038185 | 0.32 | 0.24 | 1.9349784100792e-30 | M1 macrophages | PPP4R2 |
| 1.00410503113534e-34 | -0.408900217 | 0.476 | 0.548 | 3.62250972082698e-30 | M1 macrophages | NPL |
| 1.40028429552256e-34 | -0.52110398 | 0.45 | 0.505 | 5.05180565295674e-30 | M1 macrophages | HLA-DMB |
| 4.91780264533084e-34 | -1.071587051 | 0.225 | 0.295 | 1.77419566035601e-29 | M1 macrophages | FAM20A |
| 5.73107563896982e-34 | -0.427847203 | 0.286 | 0.374 | 2.06760015827114e-29 | M1 macrophages | ALDOA |
| 8.58877783783358e-34 | 0.511068424757378 | 0.322 | 0.249 | 3.09857338055522e-29 | M1 macrophages | STX4 |
| 1.37879772702148e-33 | 0.300935381537115 | 0.464 | 0.369 | 4.97428855977541e-29 | M1 macrophages | ZCCHC7 |
| 5.79442037288133e-33 | 0.343330710368133 | 0.281 | 0.202 | 2.0904530379244e-28 | M1 macrophages | CMSS1 |
| 7.80845124214375e-33 | 0.8263761540178 | 0.334 | 0.27 | 2.8170549546282e-28 | M1 macrophages | PLAU |
| 9.80941730558539e-33 | -0.854658643 | 0.241 | 0.312 | 3.53894348133604e-28 | M1 macrophages | RESF1 |
| 1.66200847048086e-32 | -0.429654333 | 0.608 | 0.654 | 5.99602795895381e-28 | M1 macrophages | SAMHD1 |
| 2.85976975915751e-32 | 0.327510684853081 | 0.263 | 0.191 | 1.03171913601126e-27 | M1 macrophages | MAGT1 |
| 4.24957683642278e-32 | -0.921240303 | 0.301 | 0.36 | 1.53311983527625e-27 | M1 macrophages | ANKS1A |
| 1.3363924456239e-31 | 0.306445727628349 | 0.383 | 0.303 | 4.82130302607735e-27 | M1 macrophages | CCDC93 |
| 1.77376857923661e-31 | 0.356970783690477 | 0.276 | 0.203 | 6.39922490331192e-27 | M1 macrophages | NSMAF |
| 2.46091736605155e-31 | 0.351935080932356 | 0.582 | 0.486 | 8.87825158150418e-27 | M1 macrophages | PSD3 |
| 3.07175567974842e-31 | 0.365069080147859 | 0.313 | 0.24 | 1.10819729658284e-26 | M1 macrophages | RNMT |
| 4.51589772241007e-31 | 0.370077265770029 | 0.311 | 0.238 | 1.62920042131388e-26 | M1 macrophages | PNP |
| 4.9011277827316e-31 | -0.896241714 | 0.275 | 0.338 | 1.76817987017608e-26 | M1 macrophages | HECTD2 |
| 9.53884983412344e-31 | -0.908950492 | 0.257 | 0.322 | 3.44133085465671e-26 | M1 macrophages | DGLUCY |
| 1.03107547287477e-30 | 0.329321837676441 | 0.365 | 0.284 | 3.71981098349029e-26 | M1 macrophages | SAMD4A |
| 5.57831359145922e-30 | -0.449402619 | 0.648 | 0.673 | 2.01248819439074e-25 | M1 macrophages | BMP2K |
| 8.4395215253997e-30 | -0.962889138 | 0.198 | 0.262 | 3.04472618071845e-25 | M1 macrophages | MAP3K14 |
| 9.31716732874743e-30 | 0.318357198454854 | 0.265 | 0.196 | 3.36135445719221e-25 | M1 macrophages | NXF1 |
| 9.9264357043441e-30 | -0.827292132 | 0.26 | 0.321 | 3.58116020905622e-25 | M1 macrophages | TNRC6A |
| 1.29950420280345e-29 | 0.300813813095409 | 0.394 | 0.319 | 4.68822131245401e-25 | M1 macrophages | UGP2 |
| 1.61411955211987e-29 | -0.889926752 | 0.198 | 0.264 | 5.82325910818284e-25 | M1 macrophages | UACA |
| 1.84995399837071e-29 | 0.338016780410076 | 0.263 | 0.194 | 6.67407903992202e-25 | M1 macrophages | PTBP1 |
| 5.85332432846852e-29 | -0.717136393 | 0.352 | 0.409 | 2.11170381798159e-24 | M1 macrophages | PARP8 |
| 6.72718467865365e-29 | -0.771067639 | 0.214 | 0.283 | 2.42696641651788e-24 | M1 macrophages | SIRPB2 |
| 7.18451186798939e-29 | 0.341789887824607 | 0.372 | 0.293 | 2.59195634661453e-24 | M1 macrophages | RANBP2 |
| 1.0032653404988e-28 | -0.713365276 | 0.466 | 0.515 | 3.61948036891751e-24 | M1 macrophages | MYO1F |
| 1.04745287701762e-28 | 0.304297999752738 | 0.424 | 0.352 | 3.77889574441646e-24 | M1 macrophages | SQOR |
| 1.28439398058263e-28 | 0.336972686368084 | 0.436 | 0.36 | 4.63370816374797e-24 | M1 macrophages | GLRX |
| 4.89047914393079e-28 | -0.338695246 | 0.862 | 0.749 | 1.76433816075591e-23 | M1 macrophages | NPC2 |
| 7.71726017577459e-28 | -0.625320583 | 0.265 | 0.332 | 2.7841559536142e-23 | M1 macrophages | EPHX1 |
| 1.26869404551112e-27 | -0.544422646 | 0.667 | 0.679 | 4.57706750799045e-23 | M1 macrophages | TXNIP |
| 1.3983904182123e-27 | -0.30908924 | 0.79 | 0.772 | 5.04497311178452e-23 | M1 macrophages | CAPZB |
| 1.63552278993467e-27 | -0.654620939 | 0.259 | 0.326 | 5.9004755692473e-23 | M1 macrophages | GNPDA1 |
| 1.70011353738899e-27 | -0.713181217 | 0.203 | 0.269 | 6.13349960883826e-23 | M1 macrophages | TM6SF1 |
| 2.10009177737829e-27 | -0.805450587 | 0.362 | 0.412 | 7.57650110524765e-23 | M1 macrophages | ATM |
| 2.73197338103119e-27 | 0.301707920989468 | 0.252 | 0.187 | 9.85614036674623e-23 | M1 macrophages | KCTD20 |
| 3.55745978793605e-27 | 0.308654769221516 | 0.295 | 0.23 | 1.28342476769369e-22 | M1 macrophages | MRPL18 |
| 4.18257180411159e-27 | -0.447137768 | 0.517 | 0.543 | 1.50894642976934e-22 | M1 macrophages | AKR1A1 |
| 5.00310555930628e-27 | -0.777830024 | 0.197 | 0.263 | 1.80497039263093e-22 | M1 macrophages | PLA2G15 |
| 6.47163811314469e-27 | -0.920747543 | 0.234 | 0.293 | 2.33477288207921e-22 | M1 macrophages | TBC1D4 |
| 6.57987290010245e-27 | -0.780233445 | 0.296 | 0.356 | 2.37382074616996e-22 | M1 macrophages | EEF2K |
| 6.64755843740061e-27 | -0.527817772 | 0.446 | 0.499 | 2.39823965746102e-22 | M1 macrophages | CIRBP |
| 8.74331787100049e-27 | -0.700267134 | 0.528 | 0.563 | 3.15432678832085e-22 | M1 macrophages | DNM1 |
| 1.0099776800371e-26 | -0.929435433 | 0.251 | 0.308 | 3.64369647626985e-22 | M1 macrophages | SH3PXD2A |
| 1.07908043702951e-26 | -0.418074366 | 0.957 | 0.861 | 3.89299849267138e-22 | M1 macrophages | B2M |
| 1.3258350981375e-26 | -0.598076057 | 0.309 | 0.373 | 4.78321528355064e-22 | M1 macrophages | TM9SF2 |
| 1.70229348485109e-26 | -0.774177286 | 0.367 | 0.411 | 6.14136420529729e-22 | M1 macrophages | PLCL2 |
| 1.81280015205039e-26 | -0.641690348 | 0.345 | 0.404 | 6.54003910855221e-22 | M1 macrophages | TLR4 |
| 2.99947964959622e-26 | -0.58142756 | 0.263 | 0.324 | 1.08212227318483e-21 | M1 macrophages | FABP3 |
| 4.1721021516989e-26 | -0.58894099 | 0.223 | 0.292 | 1.50516929326841e-21 | M1 macrophages | VPS36 |
| 4.3828896859509e-26 | -0.483317521 | 0.211 | 0.286 | 1.58121511200051e-21 | M1 macrophages | ENSG00000278996 |
| 6.82843760667409e-26 | 0.410860459429094 | 0.33 | 0.267 | 2.46349543535981e-21 | M1 macrophages | CLIC2 |
| 7.10165334227838e-26 | -0.863608789 | 0.191 | 0.251 | 2.56206347629377e-21 | M1 macrophages | CCDC186 |
| 7.67827912527989e-26 | -0.555098763 | 0.351 | 0.415 | 2.77009276002722e-21 | M1 macrophages | TMEM14B |
| 2.04816515819613e-25 | -0.438904625 | 0.866 | 0.824 | 7.38916544122417e-21 | M1 macrophages | CFD |
| 2.12715659119926e-25 | -0.453321289 | 0.578 | 0.605 | 7.67414283406955e-21 | M1 macrophages | WASF2 |
| 2.48498325505475e-25 | -0.943165998 | 0.214 | 0.274 | 8.96507408926101e-21 | M1 macrophages | PCED1B |
| 4.0007428172194e-25 | -0.707718452 | 0.223 | 0.287 | 1.44334798616824e-20 | M1 macrophages | PHYKPL |
| 4.02535380823204e-25 | -0.629947751 | 0.434 | 0.481 | 1.45222689339587e-20 | M1 macrophages | WIPF1 |
| 4.62061995113522e-25 | -0.596907722 | 0.339 | 0.393 | 1.66698105977105e-20 | M1 macrophages | PDIA6 |
| 5.54198818850037e-25 | -0.932552742 | 0.387 | 0.426 | 1.99938307876528e-20 | M1 macrophages | NAALADL2 |
| 5.89198954604873e-25 | 0.309060750625727 | 0.332 | 0.264 | 2.125653068528e-20 | M1 macrophages | IGF2R |
| 6.04475287706748e-25 | -0.502114857 | 0.624 | 0.613 | 2.18076549545963e-20 | M1 macrophages | NUPR1 |
| 6.94052760349849e-25 | -0.951303468 | 0.393 | 0.425 | 2.50393414351415e-20 | M1 macrophages | DIP2B |
| 7.29627265384725e-25 | -0.578599086 | 0.209 | 0.276 | 2.63227628532847e-20 | M1 macrophages | GIMAP4 |
| 9.34296607170454e-25 | -0.625142804 | 0.289 | 0.353 | 3.37066186968885e-20 | M1 macrophages | MCOLN1 |
| 9.43171308731634e-25 | -0.56314103 | 0.427 | 0.478 | 3.40267913051112e-20 | M1 macrophages | PECAM1 |
| 1.59674972196008e-24 | -0.56394726 | 0.707 | 0.701 | 5.7605939719154e-20 | M1 macrophages | TBXAS1 |
| 3.24711790109821e-24 | -0.444036959 | 0.8 | 0.707 | 1.1714627251792e-19 | M1 macrophages | CD68 |
| 3.92918931041081e-24 | -0.70801763 | 0.272 | 0.33 | 1.41753362751691e-19 | M1 macrophages | OSBPL11 |
| 6.9113232774474e-24 | -0.583304867 | 0.266 | 0.332 | 2.4933980988047e-19 | M1 macrophages | NCF4 |
| 7.08961823730117e-24 | 0.352565736797808 | 0.3 | 0.238 | 2.55772157147114e-19 | M1 macrophages | KIFC3 |
| 1.32696021640837e-23 | -0.318160857 | 0.757 | 0.692 | 4.78727437273648e-19 | M1 macrophages | AIF1 |
| 2.57944059616296e-23 | -0.57852133 | 0.279 | 0.343 | 9.30584783877712e-19 | M1 macrophages | DRAM2 |
| 4.75058430294288e-23 | -0.587283083 | 0.295 | 0.355 | 1.7138682989727e-18 | M1 macrophages | LAP3 |
| 5.58222385996585e-23 | -0.652449714 | 0.23 | 0.292 | 2.01389890195988e-18 | M1 macrophages | SMIM7 |
| 8.8294516874416e-23 | -0.544689948 | 0.192 | 0.255 | 3.18540128527831e-18 | M1 macrophages | DDOST |
| 9.65412359563404e-23 | -0.791848319 | 0.239 | 0.297 | 3.48291816959689e-18 | M1 macrophages | CCM2 |
| 1.22114151799221e-22 | -0.954317644 | 0.281 | 0.336 | 4.40551225446049e-18 | M1 macrophages | CD163L1 |
| 1.44595306549819e-22 | -0.738399629 | 0.219 | 0.275 | 5.21656487439781e-18 | M1 macrophages | DNAJC13 |
| 1.69248250902746e-22 | 0.302876311360777 | 0.251 | 0.194 | 6.10596914781837e-18 | M1 macrophages | PMEPA1 |
| 2.43579448950633e-22 | -0.529079195 | 0.367 | 0.421 | 8.78761577979198e-18 | M1 macrophages | SCAMP2 |
| 2.74435898945935e-22 | -0.85304266 | 0.212 | 0.265 | 9.90082392627248e-18 | M1 macrophages | C2CD5 |
| 2.81784648173519e-22 | -0.63247312 | 0.252 | 0.312 | 1.0165944752156e-17 | M1 macrophages | DERA |
| 3.30726556819165e-22 | -0.855151523 | 0.199 | 0.256 | 1.1931621990365e-17 | M1 macrophages | PDK4 |
| 3.50865741893297e-22 | -0.559609493 | 0.305 | 0.367 | 1.26581833702845e-17 | M1 macrophages | FEZ2 |
| 7.36923178712808e-22 | -0.522238044 | 0.585 | 0.61 | 2.6585977518422e-17 | M1 macrophages | ME1 |
| 7.56243764079648e-22 | -0.948966018 | 0.303 | 0.344 | 2.72830062767014e-17 | M1 macrophages | TNRC18 |
| 9.85092336113507e-22 | -0.666729992 | 0.235 | 0.294 | 3.5539176209967e-17 | M1 macrophages | NEK6 |
| 1.06492531399084e-21 | -0.591958452 | 0.219 | 0.282 | 3.84193105528476e-17 | M1 macrophages | PSTPIP1 |
| 2.05922153595835e-21 | -0.760772138 | 0.282 | 0.338 | 7.42905353527693e-17 | M1 macrophages | MPHOSPH8 |
| 5.9756234500352e-21 | -0.437731172 | 0.451 | 0.494 | 2.1558256720692e-16 | M1 macrophages | NDFIP1 |
| 7.7297709186022e-21 | -0.53072137 | 0.308 | 0.361 | 2.78866945430412e-16 | M1 macrophages | IFITM3 |
| 2.03852112670854e-20 | -0.762063569 | 0.252 | 0.306 | 7.35437266882639e-16 | M1 macrophages | NEU1 |
| 2.9078979658992e-20 | -0.685575738 | 0.481 | 0.506 | 1.04908234915745e-15 | M1 macrophages | NFIA |
| 2.97506330481029e-20 | -0.353561839 | 0.457 | 0.502 | 1.07331358847641e-15 | M1 macrophages | RGS10 |
| 3.07415552041124e-20 | -0.487451671 | 0.511 | 0.514 | 1.10906308709876e-15 | M1 macrophages | SERPINF1 |
| 6.13542704739878e-20 | -0.633460958 | 0.206 | 0.262 | 2.21347801589006e-15 | M1 macrophages | CCDC50 |
| 7.10205690906542e-20 | -0.506320116 | 0.713 | 0.706 | 2.56220907108353e-15 | M1 macrophages | MYO5A |
| 7.7436692615921e-20 | -0.771264504 | 0.296 | 0.34 | 2.79368355950458e-15 | M1 macrophages | ADCY7 |
| 7.79319779446086e-20 | -0.765801524 | 0.237 | 0.286 | 2.81155196830765e-15 | M1 macrophages | OGA |
| 9.99206911296294e-20 | -0.567408553 | 0.28 | 0.337 | 3.60483877388364e-15 | M1 macrophages | CALCOCO2 |
| 1.04526394752428e-19 | -0.78623424 | 0.215 | 0.266 | 3.77099874348335e-15 | M1 macrophages | ATP2B4 |
| 1.0988359979961e-19 | 0.350024593405726 | 0.378 | 0.321 | 3.96427062997054e-15 | M1 macrophages | CEBPD |
| 1.32764955657597e-19 | -0.542392504 | 0.226 | 0.282 | 4.78976130525914e-15 | M1 macrophages | SDHD |
| 1.45757760115347e-19 | -0.560092321 | 0.395 | 0.44 | 5.25850271168136e-15 | M1 macrophages | TENT2 |
| 1.48572640832182e-19 | -0.924349118 | 0.268 | 0.314 | 5.36005516330262e-15 | M1 macrophages | EDA |
| 1.92045957382935e-19 | -0.652882632 | 0.196 | 0.251 | 6.92844200450414e-15 | M1 macrophages | DHRS3 |
| 2.36914567914921e-19 | -0.433870394 | 0.622 | 0.635 | 8.54716686666659e-15 | M1 macrophages | ANXA4 |
| 3.32339794336484e-19 | -0.664971951 | 0.406 | 0.442 | 1.19898227602773e-14 | M1 macrophages | DOCK11 |
| 7.4493932830729e-19 | -0.556469612 | 0.283 | 0.341 | 2.68751761473421e-14 | M1 macrophages | ARHGAP4 |
| 9.51849660124233e-19 | -0.446598577 | 0.482 | 0.516 | 3.43398801883019e-14 | M1 macrophages | CD4 |
| 1.3458362796236e-18 | -0.623880301 | 0.325 | 0.375 | 4.85537354599808e-14 | M1 macrophages | OSBPL1A |
| 1.73546688653778e-18 | -0.626590744 | 0.319 | 0.369 | 6.26104388656236e-14 | M1 macrophages | ZNF106 |
| 2.44306991285796e-18 | -0.70048412 | 0.25 | 0.297 | 8.81386332461766e-14 | M1 macrophages | SERPING1 |
| 2.55024339631291e-18 | -0.437033693 | 0.443 | 0.476 | 9.20051310087809e-14 | M1 macrophages | TMEM59 |
| 2.75007130461803e-18 | -0.748756868 | 0.206 | 0.255 | 9.92143224567047e-14 | M1 macrophages | CARD8 |
| 3.75136378511327e-18 | -0.520572777 | 0.3 | 0.348 | 1.35337951275532e-13 | M1 macrophages | TCN2 |
| 3.9994840566182e-18 | -0.778765405 | 0.271 | 0.316 | 1.44289386310615e-13 | M1 macrophages | KCNQ1 |
| 5.45416394301376e-18 | -0.641661247 | 0.498 | 0.519 | 1.96769872572107e-13 | M1 macrophages | SGMS1 |
| 6.82296171778948e-18 | -0.826447285 | 0.225 | 0.27 | 2.46151989892691e-13 | M1 macrophages | ARHGAP5 |
| 1.19396340654898e-17 | -0.491472562 | 0.55 | 0.441 | 4.30746178180674e-13 | M1 macrophages | MAN1A1 |
| 1.21338722129978e-17 | -0.686052381 | 0.244 | 0.294 | 4.37753707828323e-13 | M1 macrophages | VAV1 |
| 1.33887354426038e-17 | -0.306439586 | 0.191 | 0.25 | 4.83025408562818e-13 | M1 macrophages | LINGO1 |
| 1.7280055878783e-17 | -0.786195917 | 0.26 | 0.305 | 6.23412575938854e-13 | M1 macrophages | ZCCHC2 |
| 3.48684098788505e-17 | -0.458498187 | 0.329 | 0.375 | 1.25794762319929e-12 | M1 macrophages | AKR1B1 |
| 7.62824688012572e-17 | -0.720032875 | 0.253 | 0.299 | 2.75204262694296e-12 | M1 macrophages | MPEG1 |
| 9.02875121259517e-17 | -0.470990343 | 0.281 | 0.335 | 3.25730257496796e-12 | M1 macrophages | APPL1 |
| 9.17542559510691e-17 | -0.791816031 | 0.224 | 0.269 | 3.31021829194672e-12 | M1 macrophages | LPAR6 |
| 9.39500075222968e-17 | -0.712984867 | 0.391 | 0.42 | 3.3894344213819e-12 | M1 macrophages | AFF1 |
| 9.48107085068792e-17 | -0.371853082 | 0.625 | 0.607 | 3.42048593080268e-12 | M1 macrophages | ASAH1 |
| 9.95217204706709e-17 | -0.77044056 | 0.207 | 0.252 | 3.59044510942039e-12 | M1 macrophages | RAB11FIP1 |
| 1.65022404690366e-16 | -0.439690215 | 0.319 | 0.367 | 5.95351329401435e-12 | M1 macrophages | TMEM14C |
| 1.92522272673645e-16 | -0.636997962 | 0.424 | 0.457 | 6.94562603124707e-12 | M1 macrophages | AKAP9 |
| 2.1263681719387e-16 | -0.383830944 | 0.493 | 0.502 | 7.67129845390325e-12 | M1 macrophages | PPIB |
| 2.47995824355473e-16 | -0.405735379 | 0.309 | 0.361 | 8.94694535527242e-12 | M1 macrophages | GNPTG |
| 2.73471988486425e-16 | -0.657558014 | 0.492 | 0.504 | 9.86604892862475e-12 | M1 macrophages | TNRC6B |
| 3.09874636866787e-16 | -0.711149219 | 0.223 | 0.266 | 1.11793472742431e-11 | M1 macrophages | LRRC8D |
| 6.6793265100429e-16 | -0.58329776 | 0.457 | 0.491 | 2.40970062502818e-11 | M1 macrophages | NFIC |
| 7.26379448688054e-16 | -0.47949709 | 0.257 | 0.31 | 2.62055913703189e-11 | M1 macrophages | DECR1 |
| 8.85876778497559e-16 | -0.448153882 | 0.215 | 0.267 | 3.19597765378564e-11 | M1 macrophages | TXNDC12 |
| 1.30590871985138e-15 | -0.553000429 | 0.379 | 0.421 | 4.71132688860782e-11 | M1 macrophages | SPATS2L |
| 1.52653218443782e-15 | -0.735508855 | 0.217 | 0.259 | 5.50727016179631e-11 | M1 macrophages | AGO4 |
| 1.79803354199615e-15 | -0.725995806 | 0.3 | 0.338 | 6.48676560945951e-11 | M1 macrophages | NKTR |
| 2.03337587878439e-15 | -0.369818305 | 0.628 | 0.629 | 7.33581015789045e-11 | M1 macrophages | AP1B1 |
| 2.58914951868715e-15 | -0.824354383 | 0.281 | 0.316 | 9.34087471856764e-11 | M1 macrophages | DNMT3A |
| 3.26185290809803e-15 | 0.302255111444912 | 0.3 | 0.25 | 1.17677867365452e-10 | M1 macrophages | SLC8B1 |
| 3.31770637677712e-15 | -0.331135049 | 0.567 | 0.569 | 1.19692892954988e-10 | M1 macrophages | CLTA |
| 3.37961760324231e-15 | 0.346761583717649 | 0.27 | 0.221 | 1.21926464272173e-10 | M1 macrophages | FGFR1 |
| 3.8319894789325e-15 | -0.491470874 | 0.248 | 0.298 | 1.38246684431448e-10 | M1 macrophages | IRF2 |
| 8.51758942230197e-15 | -0.573112977 | 0.229 | 0.276 | 3.07289073588388e-10 | M1 macrophages | LYPLAL1 |
| 8.61334969106244e-15 | -0.447866853 | 0.242 | 0.293 | 3.1074381680446e-10 | M1 macrophages | FAM204A |
| 8.98035099031814e-15 | -0.50320856 | 0.28 | 0.327 | 3.23984122677708e-10 | M1 macrophages | TECR |
| 1.07218561461291e-14 | -0.711575922 | 0.258 | 0.3 | 3.86812404183901e-10 | M1 macrophages | GIT2 |
| 1.08976149921406e-14 | -0.390904115 | 0.241 | 0.291 | 3.93153256071457e-10 | M1 macrophages | PPCS |
| 1.16193873695817e-14 | -0.55645644 | 0.225 | 0.272 | 4.191926381324e-10 | M1 macrophages | CAT |
| 1.20372232765635e-14 | -0.437308114 | 0.289 | 0.334 | 4.34266904148582e-10 | M1 macrophages | TMED9 |
| 1.83940155095141e-14 | -0.451594243 | 0.362 | 0.407 | 6.63600897536739e-10 | M1 macrophages | UNC93B1 |
| 3.17200283490405e-14 | -0.615138295 | 0.228 | 0.272 | 1.14436346274833e-09 | M1 macrophages | CYBRD1 |
| 3.25329674506902e-14 | -0.55443885 | 0.211 | 0.255 | 1.17369186671855e-09 | M1 macrophages | FOXN2 |
| 3.36794975497962e-14 | -0.424077795 | 0.233 | 0.283 | 1.215055233104e-09 | M1 macrophages | LTBR |
| 3.41961164309892e-14 | -0.595217092 | 0.436 | 0.455 | 1.2336932924808e-09 | M1 macrophages | RREB1 |
| 3.9322936327186e-14 | -0.39426802 | 0.331 | 0.374 | 1.41865357387589e-09 | M1 macrophages | SPCS1 |
| 5.43638087874832e-14 | -0.389275735 | 0.577 | 0.589 | 1.96128312962603e-09 | M1 macrophages | A2M |
| 7.18588960039948e-14 | -0.344274276 | 0.456 | 0.478 | 2.59245339113612e-09 | M1 macrophages | CTSH |
| 8.91345684222417e-14 | -1.01080166 | 0.471 | 0.479 | 3.21570782496922e-09 | M1 macrophages | SFMBT2 |
| 9.10736637342577e-14 | -0.661147137 | 0.234 | 0.274 | 3.28566456654081e-09 | M1 macrophages | IKBKB |
| 9.55928956950555e-14 | -0.389502741 | 0.352 | 0.399 | 3.44870489799052e-09 | M1 macrophages | HMGN3 |
| 1.08765633182791e-13 | -0.502466541 | 0.436 | 0.467 | 3.92393774833554e-09 | M1 macrophages | MKNK1 |
| 1.14425035707249e-13 | -0.408112722 | 0.49 | 0.487 | 4.12811201321041e-09 | M1 macrophages | COX6A1 |
| 1.23712052519473e-13 | -0.324180013 | 0.656 | 0.619 | 4.46315971874503e-09 | M1 macrophages | COMT |
| 1.63169171966397e-13 | -0.394289513 | 0.366 | 0.399 | 5.88665421703169e-09 | M1 macrophages | CIAO2A |
| 2.05438492272501e-13 | -0.856515367 | 0.465 | 0.469 | 7.411604485715e-09 | M1 macrophages | FCHSD2 |
| 2.25190177801125e-13 | -0.657521243 | 0.306 | 0.338 | 8.1241860445312e-09 | M1 macrophages | INPP4A |
| 2.65334419897727e-13 | -0.501295886 | 0.259 | 0.305 | 9.57246986665031e-09 | M1 macrophages | EPN1 |
| 3.73625586183472e-13 | -0.821330018 | 0.436 | 0.444 | 1.34792902727411e-08 | M1 macrophages | AUTS2 |
| 4.58820703469997e-13 | -0.731093209 | 0.406 | 0.428 | 1.65528745190871e-08 | M1 macrophages | PNISR |
| 4.98482627239429e-13 | -0.711064046 | 0.279 | 0.315 | 1.79837577429169e-08 | M1 macrophages | NSD1 |
| 6.10212650117969e-13 | -0.526881825 | 0.218 | 0.262 | 2.2014641778306e-08 | M1 macrophages | PPA2 |
| 6.31646035418608e-13 | -0.664334713 | 0.269 | 0.305 | 2.27878940197971e-08 | M1 macrophages | LRRK1 |
| 7.31264215282972e-13 | -0.651290652 | 0.442 | 0.461 | 2.63818190947638e-08 | M1 macrophages | TTC7A |
| 7.71409360456498e-13 | -0.569751533 | 0.335 | 0.367 | 2.78301354971891e-08 | M1 macrophages | JAK2 |
| 7.98706512980964e-13 | -0.635574124 | 0.28 | 0.316 | 2.88149348688142e-08 | M1 macrophages | JPX |
| 8.40494143540956e-13 | -0.798014527 | 0.221 | 0.255 | 3.03225072165271e-08 | M1 macrophages | ENSG00000291015 |
| 9.14795531045669e-13 | -0.420175552 | 0.287 | 0.332 | 3.30030783735346e-08 | M1 macrophages | MLEC |
| 9.3279876474305e-13 | -0.410425381 | 0.205 | 0.252 | 3.3652581035635e-08 | M1 macrophages | ERLEC1 |
| 1.0457827377681e-12 | -0.429559033 | 0.468 | 0.496 | 3.77287038304597e-08 | M1 macrophages | MERTK |
| 1.30502161997851e-12 | -0.498958932 | 0.246 | 0.291 | 4.70812649839648e-08 | M1 macrophages | NIPSNAP2 |
| 1.37157609248344e-12 | -0.375330485 | 0.492 | 0.503 | 4.94823506885251e-08 | M1 macrophages | CD59 |
| 1.49971028103824e-12 | -0.416453856 | 0.687 | 0.682 | 5.41050478090167e-08 | M1 macrophages | TNS1 |
| 1.62627036292044e-12 | -0.38470897 | 0.429 | 0.457 | 5.86709558830806e-08 | M1 macrophages | UCP2 |
| 2.66019121783755e-12 | -0.371392051 | 0.462 | 0.494 | 9.59717185659253e-08 | M1 macrophages | BNIP3L |
| 2.85348210460166e-12 | -0.383737779 | 0.406 | 0.428 | 1.02945073887714e-07 | M1 macrophages | ISCU |
| 3.86266457594594e-12 | -0.35423384 | 0.243 | 0.289 | 1.39353349906402e-07 | M1 macrophages | MRPL34 |
| 4.1159833076883e-12 | -0.681420358 | 0.35 | 0.38 | 1.48492329791471e-07 | M1 macrophages | DNAJC5 |
| 4.56854405239132e-12 | -0.437585214 | 0.472 | 0.495 | 1.64819363778122e-07 | M1 macrophages | NCKAP1L |
| 4.68112863601635e-12 | -0.489134786 | 0.331 | 0.37 | 1.68881077801562e-07 | M1 macrophages | LY96 |
| 5.00394823505795e-12 | -0.655388182 | 0.23 | 0.266 | 1.80527440476186e-07 | M1 macrophages | RELL1 |
| 5.80378110924857e-12 | -0.708393938 | 0.231 | 0.267 | 2.09383011078361e-07 | M1 macrophages | ENSG00000291214 |
| 5.82532565390592e-12 | -0.53477311 | 0.289 | 0.331 | 2.10160273615964e-07 | M1 macrophages | ARHGAP30 |
| 6.82761712250797e-12 | -0.401365735 | 0.233 | 0.277 | 2.4631994292872e-07 | M1 macrophages | SLC66A3 |
| 1.1061245931979e-11 | -0.441218068 | 0.268 | 0.312 | 3.99056569488005e-07 | M1 macrophages | PSMG2 |
| 1.54267914319045e-11 | -0.333239411 | 0.341 | 0.383 | 5.56552354488818e-07 | M1 macrophages | GSTK1 |
| 1.59937509571575e-11 | -0.344460623 | 0.239 | 0.285 | 5.77006553281372e-07 | M1 macrophages | HACD4 |
| 1.64141040069773e-11 | -0.555497063 | 0.393 | 0.421 | 5.92171630259721e-07 | M1 macrophages | TGFBR1 |
| 1.7899574977996e-11 | -0.555682956 | 0.363 | 0.393 | 6.45762966481162e-07 | M1 macrophages | PCM1 |
| 3.30185362406323e-11 | -0.500083294 | 0.333 | 0.37 | 1.19120973195329e-06 | M1 macrophages | DDX46 |
| 3.71914495515254e-11 | -0.329557608 | 0.533 | 0.544 | 1.34175592547038e-06 | M1 macrophages | HEXA |
| 3.91696883885261e-11 | -0.415498545 | 0.287 | 0.324 | 1.41312484799286e-06 | M1 macrophages | TNFSF13 |
| 4.0325340222633e-11 | -0.366307819 | 0.227 | 0.271 | 1.45481729921193e-06 | M1 macrophages | ABRACL |
| 4.5009296855424e-11 | -0.407251046 | 0.404 | 0.433 | 1.62380040265313e-06 | M1 macrophages | ARL5A |
| 4.59060954339867e-11 | -0.531242332 | 0.443 | 0.47 | 1.65615420497194e-06 | M1 macrophages | SRSF11 |
| 4.72369813779242e-11 | -0.526747651 | 0.401 | 0.431 | 1.70416857717137e-06 | M1 macrophages | PLXND1 |
| 4.79057309276523e-11 | -0.403952527 | 0.395 | 0.426 | 1.72829505467691e-06 | M1 macrophages | HMGN2 |
| 4.79154818099261e-11 | -0.427691472 | 0.238 | 0.279 | 1.7286468372567e-06 | M1 macrophages | SELPLG |
| 5.02071403871831e-11 | -0.332429081 | 0.337 | 0.376 | 1.8113230037484e-06 | M1 macrophages | KDELR1 |
| 5.05846868945141e-11 | -0.605539582 | 0.247 | 0.284 | 1.82494374909338e-06 | M1 macrophages | STK38 |
| 5.10631716345473e-11 | -0.444994676 | 0.22 | 0.261 | 1.84220604305956e-06 | M1 macrophages | WASHC3 |
| 6.4728477121919e-11 | -0.328803296 | 0.352 | 0.394 | 2.33520926912747e-06 | M1 macrophages | FARP1 |
| 7.07123632716618e-11 | -0.565741557 | 0.227 | 0.264 | 2.55108992975174e-06 | M1 macrophages | CCPG1 |
| 7.31249181655953e-11 | -0.542461499 | 0.321 | 0.352 | 2.63812767266018e-06 | M1 macrophages | CBL |
| 8.36294465983313e-11 | -0.441804533 | 0.31 | 0.349 | 3.017099544928e-06 | M1 macrophages | PTPN18 |
| 8.57846759287925e-11 | -0.370024396 | 0.376 | 0.404 | 3.09485375348305e-06 | M1 macrophages | MIR99AHG |
| 9.89596719011267e-11 | -0.712851312 | 0.511 | 0.509 | 3.57016808317695e-06 | M1 macrophages | CHN2 |
| 1.20930855120145e-10 | -0.407038132 | 0.468 | 0.497 | 4.36282246016946e-06 | M1 macrophages | VOPP1 |
| 1.24967005344234e-10 | -0.311282523 | 0.536 | 0.548 | 4.50843465180395e-06 | M1 macrophages | HCLS1 |
| 1.25183751819008e-10 | -0.678133077 | 0.244 | 0.276 | 4.51625421437433e-06 | M1 macrophages | ATF7 |
| 1.69606468565278e-10 | -0.584718344 | 0.268 | 0.301 | 6.11889256642954e-06 | M1 macrophages | ARID4A |
| 1.71664199078159e-10 | -0.445077826 | 0.313 | 0.35 | 6.19312931014273e-06 | M1 macrophages | KLF3 |
| 2.12257211502471e-10 | -0.575503763 | 0.259 | 0.294 | 7.65760341937463e-06 | M1 macrophages | PHF3 |
| 2.17574728364418e-10 | -0.548717288 | 0.225 | 0.262 | 7.84944347520309e-06 | M1 macrophages | DCP2 |
| 2.24101544980537e-10 | -0.342015938 | 0.243 | 0.283 | 8.08491143826285e-06 | M1 macrophages | NDUFB3 |
| 2.26364904278963e-10 | -0.359805385 | 0.469 | 0.485 | 8.16656665167216e-06 | M1 macrophages | SKP1 |
| 2.57160751384999e-10 | -0.325953124 | 0.482 | 0.5 | 9.27758842771663e-06 | M1 macrophages | SCP2 |
| 2.59192273504616e-10 | -0.551045371 | 0.231 | 0.265 | 9.35087965122601e-06 | M1 macrophages | MBOAT1 |
| 2.68618243381857e-10 | -0.624396083 | 0.223 | 0.257 | 9.69094036648724e-06 | M1 macrophages | SPOP |
| 2.73559553513636e-10 | -0.557926889 | 0.27 | 0.304 | 9.86920801211146e-06 | M1 macrophages | LONP2 |
| 2.95513671531793e-10 | -0.456025756 | 0.365 | 0.4 | 1.06612467278525e-05 | M1 macrophages | RNF135 |
| 3.36506959879024e-10 | -0.318086385 | 0.483 | 0.496 | 1.21401615915556e-05 | M1 macrophages | REEP5 |
| 3.49265494086553e-10 | -0.453607392 | 0.343 | 0.372 | 1.26004512301606e-05 | M1 macrophages | SNX13 |
| 4.51394236245356e-10 | -0.319379042 | 0.289 | 0.329 | 1.62849498610237e-05 | M1 macrophages | TMEM179B |
| 4.78447137874679e-10 | -0.454561241 | 0.402 | 0.427 | 1.72609373931048e-05 | M1 macrophages | CTNND1 |
| 4.79802996920371e-10 | -0.510617263 | 0.311 | 0.346 | 1.73098527198962e-05 | M1 macrophages | MAX |
| 5.04604827397773e-10 | -0.646256164 | 0.25 | 0.282 | 1.82046283580295e-05 | M1 macrophages | ZRANB2 |
| 5.89609452286808e-10 | -0.422885598 | 0.269 | 0.304 | 2.12713402101512e-05 | M1 macrophages | MYDGF |
| 6.68175621115197e-10 | -0.420996151 | 0.349 | 0.386 | 2.4105771882973e-05 | M1 macrophages | MIS18BP1 |
| 7.24130407971484e-10 | -0.529912235 | 0.282 | 0.319 | 2.61244527283872e-05 | M1 macrophages | MGMT |
| 7.28793674717591e-10 | -0.596910901 | 0.345 | 0.376 | 2.62926894027865e-05 | M1 macrophages | ACYP2 |
| 7.32632832043293e-10 | -0.580877002 | 0.221 | 0.253 | 2.64311946816259e-05 | M1 macrophages | PHC3 |
| 7.38474107220482e-10 | -0.542847525 | 0.361 | 0.384 | 2.66419303661933e-05 | M1 macrophages | SETX |
| 7.58184765517264e-10 | -0.347665963 | 0.307 | 0.343 | 2.73530317855663e-05 | M1 macrophages | SRP9 |
| 7.82020548147832e-10 | -0.488426577 | 0.219 | 0.257 | 2.82129553155293e-05 | M1 macrophages | UQCC5 |
| 8.90166338266045e-10 | -0.355674673 | 0.252 | 0.289 | 3.21145309856241e-05 | M1 macrophages | MPC2 |
| 9.91041852094533e-10 | -0.301233587 | 0.732 | 0.668 | 3.57538168980145e-05 | M1 macrophages | HLA-E |
| 1.30614425909997e-09 | -0.334197775 | 0.315 | 0.352 | 4.71217664355498e-05 | M1 macrophages | ERP29 |
| 1.55729913188109e-09 | -0.727052455 | 0.233 | 0.26 | 5.6182680780874e-05 | M1 macrophages | FBXL20 |
| 1.93632536269464e-09 | -0.343452103 | 0.864 | 0.763 | 6.98568101099346e-05 | M1 macrophages | TYROBP |
| 1.93719956693658e-09 | -0.47267943 | 0.248 | 0.285 | 6.98883487763711e-05 | M1 macrophages | ZBTB8OS |
| 1.94845611790648e-09 | -0.543840716 | 0.219 | 0.253 | 7.0294451365712e-05 | M1 macrophages | RCBTB2 |
| 2.02923605041843e-09 | -0.736437332 | 0.308 | 0.332 | 7.32087489909457e-05 | M1 macrophages | OXR1 |
| 2.09753798139248e-09 | -0.397330624 | 0.265 | 0.301 | 7.56728777546965e-05 | M1 macrophages | TCEAL4 |
| 2.21854921728252e-09 | -0.398428051 | 0.232 | 0.268 | 8.00386001119013e-05 | M1 macrophages | OSTC |
| 2.25500006110613e-09 | -0.4712439 | 0.421 | 0.444 | 8.13536372045258e-05 | M1 macrophages | IFI16 |
| 2.30231850574993e-09 | -0.536974738 | 0.387 | 0.408 | 8.30607447319401e-05 | M1 macrophages | EPS8 |
| 3.43853738090313e-09 | -0.494535474 | 0.221 | 0.254 | 0.000124052113090842 | M1 macrophages | ZZZ3 |
| 4.76100698986524e-09 | -0.361906264 | 0.453 | 0.456 | 0.000171762849173368 | M1 macrophages | UQCR10 |
| 4.99579725990741e-09 | -0.327327278 | 0.266 | 0.304 | 0.00018023337774568 | M1 macrophages | UBE2F |
| 5.32660473996525e-09 | -0.360923713 | 0.217 | 0.255 | 0.000192167919203726 | M1 macrophages | CNPY2 |
| 5.93827922003412e-09 | -0.502455155 | 0.336 | 0.366 | 0.000214235299421171 | M1 macrophages | PRMT2 |
| 6.33793980810136e-09 | -0.592942009 | 0.303 | 0.327 | 0.000228653854456873 | M1 macrophages | ARID1A |
| 6.89429026865168e-09 | -0.356261925 | 0.443 | 0.469 | 0.000248725310022147 | M1 macrophages | NUCKS1 |
| 7.19221775427394e-09 | -0.599897546 | 0.268 | 0.296 | 0.000259473639920941 | M1 macrophages | APC |
| 7.40559388704179e-09 | -0.455268476 | 0.282 | 0.317 | 0.000267171610662807 | M1 macrophages | ANP32A |
| 1.02012772896631e-08 | -0.660136859 | 0.269 | 0.294 | 0.000368031480779174 | M1 macrophages | MLXIP |
| 1.1936515037245e-08 | -0.505008058 | 0.329 | 0.353 | 0.000430633652998688 | M1 macrophages | OSBPL3 |
| 1.20292659189901e-08 | -0.426695185 | 0.262 | 0.298 | 0.000433979826559405 | M1 macrophages | PRSS23 |
| 1.25265621211904e-08 | -0.313121891 | 0.489 | 0.485 | 0.000451920781646185 | M1 macrophages | PPDPF |
| 1.9384085151248e-08 | -0.301382905 | 0.273 | 0.31 | 0.000699319640001574 | M1 macrophages | ADIPOR1 |
| 2.18584850836533e-08 | -0.320219956 | 0.461 | 0.463 | 0.000788588566362959 | M1 macrophages | UQCRQ |
| 2.95743631382746e-08 | -0.388865162 | 0.224 | 0.262 | 0.00106695429893953 | M1 macrophages | TNFRSF14 |
| 3.03416997677826e-08 | -0.496907404 | 0.225 | 0.26 | 0.00109463750252229 | M1 macrophages | ANAPC5 |
| 3.07951060136915e-08 | -0.39484937 | 0.364 | 0.394 | 0.00111099503965595 | M1 macrophages | HP1BP3 |
| 3.402560020123e-08 | -0.412210957 | 0.23 | 0.263 | 0.00122754157845978 | M1 macrophages | SDHC |
| 4.18840871008116e-08 | -0.620064277 | 0.278 | 0.303 | 0.00151105221033598 | M1 macrophages | ERICH1 |
| 4.83602807541189e-08 | -0.355483478 | 0.326 | 0.358 | 0.00174469384876635 | M1 macrophages | MMP24OS |
| 5.18008754927126e-08 | -0.677404589 | 0.225 | 0.25 | 0.00186882018515059 | M1 macrophages | RHOT1 |
| 5.37557737435402e-08 | -0.305302167 | 0.5 | 0.522 | 0.0019393470493457 | M1 macrophages | PAK2 |
| 5.99656421205313e-08 | -0.339633892 | 0.278 | 0.309 | 0.00216338047078241 | M1 macrophages | IFITM2 |
| 7.52152419424802e-08 | -0.325431485 | 0.474 | 0.487 | 0.00271354028355886 | M1 macrophages | GAA |
| 8.25805265674649e-08 | -0.69657886 | 0.276 | 0.296 | 0.00297925765697443 | M1 macrophages | MIR3667HG |
| 8.5489522922159e-08 | -0.648770277 | 0.458 | 0.458 | 0.00308420551846273 | M1 macrophages | MGAT5 |
| 8.94374321304288e-08 | -0.53479399 | 0.26 | 0.288 | 0.00322663423896948 | M1 macrophages | SEC14L1 |
| 9.01550848581223e-08 | -0.308642308 | 0.259 | 0.294 | 0.00325252499642648 | M1 macrophages | CUTA |
| 1.04869628787468e-07 | -0.353502616 | 0.305 | 0.333 | 0.00378338159776549 | M1 macrophages | ATRAID |
| 1.06830439212974e-07 | -0.521862472 | 0.241 | 0.271 | 0.00385412175548647 | M1 macrophages | U2SURP |
| 1.28577150511199e-07 | -0.507939451 | 0.313 | 0.338 | 0.00463867785899253 | M1 macrophages | NPEPPS |
| 1.29108754090907e-07 | -0.618625499 | 0.415 | 0.415 | 0.00465785652133764 | M1 macrophages | STARD13 |
| 1.36045454090102e-07 | -0.489373102 | 0.328 | 0.35 | 0.00490811184720861 | M1 macrophages | ATF7IP |
| 1.41486933120216e-07 | -0.424736927 | 0.246 | 0.278 | 0.00510442408617805 | M1 macrophages | KRCC1 |
| 1.42718780713216e-07 | -0.327484589 | 0.483 | 0.48 | 0.0051488654517907 | M1 macrophages | PEBP1 |
| 1.59321799869219e-07 | -0.716470826 | 0.441 | 0.432 | 0.00574785257388182 | M1 macrophages | DISC1 |
| 1.76619123207994e-07 | -0.331990096 | 0.373 | 0.403 | 0.00637188810797481 | M1 macrophages | SLC38A6 |
| 1.81343946108378e-07 | -0.341913963 | 0.454 | 0.47 | 0.00654234554375196 | M1 macrophages | TGFBR2 |
| 2.42611143965901e-07 | -0.3275095 | 0.301 | 0.335 | 0.00875268224085781 | M1 macrophages | PSMF1 |
| 2.6844380678378e-07 | -0.539062567 | 0.657 | 0.629 | 0.00968464721733844 | M1 macrophages | FRMD4A |
| 2.74543002998852e-07 | -0.309364399 | 0.235 | 0.269 | 0.00990468791918959 | M1 macrophages | COX16 |
| 3.0806068672382e-07 | -0.345892087 | 0.34 | 0.367 | 0.0111139053949353 | M1 macrophages | RPN2 |
| 3.10731604510046e-07 | -0.615493097 | 0.671 | 0.627 | 0.0112102640959089 | M1 macrophages | SNX29 |
| 3.12991708062387e-07 | -0.398380603 | 0.249 | 0.281 | 0.0112918018517668 | M1 macrophages | RNPEP |
| 3.52443629500351e-07 | -0.786000033 | 0.254 | 0.272 | 0.0127151088214842 | M1 macrophages | SIPA1L3 |
| 3.55182588986933e-07 | -0.487756859 | 0.44 | 0.446 | 0.0128139222628816 | M1 macrophages | GRK3 |
| 3.82856210510446e-07 | -0.481967555 | 0.235 | 0.259 | 0.0138123035065854 | M1 macrophages | RBM26 |
| 4.55612624720451e-07 | -0.463581338 | 0.436 | 0.442 | 0.0164371366620397 | M1 macrophages | KANSL1 |
| 7.34544273857753e-07 | -0.474083544 | 0.384 | 0.404 | 0.0265001537679662 | M1 macrophages | UBE2K |
| 7.36449355764827e-07 | -0.434801233 | 0.473 | 0.472 | 0.0265688834079277 | M1 macrophages | WDFY3 |
| 9.08867500792876e-07 | -0.53303935 | 0.259 | 0.285 | 0.0327892128261046 | M1 macrophages | PRPF4B |
| 9.11456832060644e-07 | -0.321973523 | 0.329 | 0.36 | 0.0328826281302518 | M1 macrophages | STMP1 |
| 1.02424155688461e-06 | -0.601357661 | 0.251 | 0.274 | 0.0369515626477262 | M1 macrophages | PPP6R2 |
| 1.203003839646e-06 | -0.352243385 | 0.307 | 0.335 | 0.0434007695229089 | M1 macrophages | ST8SIA4 |
| 1.32443398891617e-06 | -0.44154662 | 0.325 | 0.351 | 0.0477816050181286 | M1 macrophages | LSM14A |
| 1.67949416055411e-06 | -0.30594138 | 0.33 | 0.359 | 0.0605911108303105 | M1 macrophages | PAIP2 |
| 1.91076625805765e-06 | -0.531076294 | 0.456 | 0.46 | 0.068934714291946 | M1 macrophages | ATRX |
| 1.96245461818278e-06 | -0.454148681 | 0.373 | 0.39 | 0.0707994752601803 | M1 macrophages | SENP6 |
| 2.00071319151502e-06 | -0.611291501 | 0.23 | 0.251 | 0.0721797298102875 | M1 macrophages | HERC2 |
| 2.02855820409623e-06 | -0.416864119 | 0.509 | 0.508 | 0.0731842943291797 | M1 macrophages | VPS13C |
| 2.35208173016373e-06 | -0.342384096 | 0.233 | 0.262 | 0.0848560525791169 | M1 macrophages | EPSTI1 |
| 2.44571163836204e-06 | -0.340786208 | 0.242 | 0.272 | 0.0882339387771874 | M1 macrophages | CTBS |
| 2.71636260478109e-06 | -0.307193958 | 0.235 | 0.265 | 0.0979982136926874 | M1 macrophages | IER3IP1 |
| 4.0355857593261e-06 | -0.568464935 | 0.247 | 0.271 | 0.145591827439208 | M1 macrophages | MOB3A |
| 4.39250569710529e-06 | -0.403797119 | 0.353 | 0.374 | 0.158468428034468 | M1 macrophages | EPC1 |
| 4.6448258645248e-06 | -0.432965626 | 0.313 | 0.337 | 0.167571382714461 | M1 macrophages | PCMTD1 |
| 5.12040302074956e-06 | -0.457379546 | 0.259 | 0.281 | 0.184728779779582 | M1 macrophages | SNX14 |
| 7.71736007326538e-06 | -0.418252155 | 0.564 | 0.565 | 0.278419199363195 | M1 macrophages | GOLGA4 |
| 8.22559143480208e-06 | -0.481469161 | 0.671 | 0.634 | 0.296754662193355 | M1 macrophages | FTX |
| 9.81305810953577e-06 | -0.356225888 | 0.601 | 0.588 | 0.354025697417722 | M1 macrophages | SON |
| 1.01361506032951e-05 | -0.530359459 | 0.264 | 0.285 | 0.365681905315077 | M1 macrophages | MAPRE2 |
| 1.02022918938567e-05 | -0.353695116 | 0.227 | 0.254 | 0.368068084654668 | M1 macrophages | RALB |
| 1.06352452319492e-05 | -0.461723842 | 0.265 | 0.283 | 0.38368774223303 | M1 macrophages | ZNF292 |
| 1.25821808912975e-05 | -0.364440364 | 0.275 | 0.304 | 0.453927340015339 | M1 macrophages | UBE2E3 |
| 1.26294111051558e-05 | -0.313464533 | 0.243 | 0.272 | 0.455631264440706 | M1 macrophages | VPS4B |
| 1.28390449812309e-05 | -0.390223927 | 0.381 | 0.4 | 0.463194225787867 | M1 macrophages | FKBP15 |
| 1.33533785059894e-05 | -0.434754756 | 0.47 | 0.476 | 0.481749836360581 | M1 macrophages | TACC1 |
| 1.34150007492701e-05 | -0.346948075 | 0.259 | 0.287 | 0.483972982031417 | M1 macrophages | SORBS3 |
| 1.56579322547992e-05 | -0.329369787 | 0.34 | 0.366 | 0.56489122195639 | M1 macrophages | LY86 |
| 1.57307043713644e-05 | -0.502154589 | 0.235 | 0.256 | 0.567516621605712 | M1 macrophages | DENND4B |
| 1.61312795592211e-05 | -0.348301535 | 0.239 | 0.268 | 0.58196817265802 | M1 macrophages | NDUFV1 |
| 1.72548787333929e-05 | -0.591910251 | 0.249 | 0.265 | 0.622504260064616 | M1 macrophages | ATXN7L1 |
| 1.74516946836879e-05 | -0.376497306 | 0.238 | 0.264 | 0.629604789103409 | M1 macrophages | SNX5 |
| 1.80236182305883e-05 | -0.340419298 | 0.294 | 0.317 | 0.650238074904933 | M1 macrophages | LRPAP1 |
| 1.9699648673504e-05 | -0.526920393 | 0.242 | 0.261 | 0.710704225194005 | M1 macrophages | UBE4B |
| 1.99454930180057e-05 | -0.661969963 | 0.283 | 0.294 | 0.719573551610593 | M1 macrophages | ABCC1 |
| 2.24865821182494e-05 | -0.496785566 | 0.237 | 0.259 | 0.811248423080082 | M1 macrophages | CORO7 |
| 2.29078115157454e-05 | -0.538983394 | 0.372 | 0.383 | 0.826445116053547 | M1 macrophages | EYA2 |
| 2.44910573472135e-05 | -0.519171942 | 0.358 | 0.37 | 0.883563875915421 | M1 macrophages | DNM2 |
| 2.56965332366943e-05 | -0.506867295 | 0.267 | 0.286 | 0.92705382958022 | M1 macrophages | GIGYF2 |
| 2.61836211361275e-05 | -0.42518453 | 0.387 | 0.395 | 0.944626499728071 | M1 macrophages | PHKB |
| 0 | 2.51843302870781 | 0.558 | 0.206 | 0 | M2 macrophages | F13A1 |
| 0 | 2.28189198393145 | 0.582 | 0.256 | 0 | M2 macrophages | LYVE1 |
| 0 | 1.70286237083486 | 0.537 | 0.235 | 0 | M2 macrophages | SLC40A1 |
| 0 | 1.93534392251065 | 0.823 | 0.55 | 0 | M2 macrophages | SELENOP |
| 0 | -2.352309261 | 0.238 | 0.505 | 0 | M2 macrophages | TPRG1 |
| 0 | -2.777022256 | 0.244 | 0.511 | 0 | M2 macrophages | OLR1 |
| 0 | 1.38531387870456 | 0.608 | 0.347 | 0 | M2 macrophages | EMB |
| 0 | 1.32098826235576 | 0.696 | 0.451 | 0 | M2 macrophages | MRC1 |
| 0 | 1.29846038895591 | 0.599 | 0.36 | 0 | M2 macrophages | TMEM176B |
| 0 | 1.09440049377891 | 0.699 | 0.462 | 0 | M2 macrophages | GGTA1 |
| 0 | 3.37544828020735 | 0.287 | 0.061 | 0 | M2 macrophages | TTN |
| 0 | 2.18330045800463 | 0.29 | 0.089 | 0 | M2 macrophages | FGF13 |
| 0 | 1.00993364979262 | 0.729 | 0.53 | 0 | M2 macrophages | MS4A4A |
| 0 | 1.28611319638215 | 0.754 | 0.562 | 0 | M2 macrophages | FOLR2 |
| 0 | -2.165566619 | 0.504 | 0.682 | 0 | M2 macrophages | KYNU |
| 0 | -1.243424637 | 0.536 | 0.709 | 0 | M2 macrophages | PIK3R5 |
| 0 | 1.30931461360311 | 0.879 | 0.719 | 0 | M2 macrophages | LGMN |
| 0 | -1.612619159 | 0.646 | 0.8 | 0 | M2 macrophages | FMNL2 |
| 0 | -0.977091538 | 0.784 | 0.899 | 0 | M2 macrophages | FN1 |
| 0 | 0.958376521362405 | 0.891 | 0.789 | 0 | M2 macrophages | FCGRT |
| 0 | -0.887509941 | 0.828 | 0.901 | 0 | M2 macrophages | PLXDC2 |
| 0 | 1.29266644700952 | 0.842 | 0.777 | 0 | M2 macrophages | RNASE1 |
| 0 | 0.789040850039867 | 0.861 | 0.808 | 0 | M2 macrophages | ITM2B |
| 3.06320700421573e-322 | 1.25172127769202 | 0.789 | 0.708 | 1.10511319091091e-317 | M2 macrophages | C1QC |
| 8.3270402026652e-314 | -1.272150923 | 0.656 | 0.782 | 3.00414629391552e-309 | M2 macrophages | DOCK4 |
| 1.89811328190447e-312 | -1.878845528 | 0.257 | 0.484 | 6.84782328712677e-308 | M2 macrophages | DOCK10 |
| 2.44825950366862e-306 | 1.00883426884654 | 0.816 | 0.726 | 8.83258581138529e-302 | M2 macrophages | PLTP |
| 5.65039564916617e-306 | 1.11451083326478 | 0.662 | 0.433 | 2.03849323834968e-301 | M2 macrophages | BLVRB |
| 4.85004954886586e-304 | 1.49550806905841 | 0.437 | 0.208 | 1.74975237574434e-299 | M2 macrophages | GAS6 |
| 1.58984450091743e-300 | -1.562606395 | 0.686 | 0.79 | 5.73568200595982e-296 | M2 macrophages | CD44 |
| 2.6340789203129e-297 | 2.01742795663491 | 0.301 | 0.106 | 9.50296652081286e-293 | M2 macrophages | FXYD6 |
| 3.47811030026893e-297 | 1.33304561129036 | 0.464 | 0.236 | 1.25479785302802e-292 | M2 macrophages | TMEM176A |
| 7.68878444958105e-286 | 0.635410281213549 | 0.871 | 0.761 | 2.77388276587536e-281 | M2 macrophages | MS4A6A |
| 8.89898941376339e-285 | -1.346873361 | 0.54 | 0.695 | 3.21048841080342e-280 | M2 macrophages | LIMS1 |
| 3.53178017287923e-278 | 1.00302705848977 | 0.733 | 0.526 | 1.27416033296964e-273 | M2 macrophages | STAB1 |
| 3.01328304049001e-275 | 0.718863346567604 | 0.838 | 0.78 | 1.08710212251758e-270 | M2 macrophages | C1QA |
| 1.35312865069585e-269 | 1.48271879553019 | 0.41 | 0.197 | 4.88168223311542e-265 | M2 macrophages | NAV2 |
| 4.2152200560438e-261 | -1.16460953 | 0.405 | 0.596 | 1.52072493961892e-256 | M2 macrophages | ABCC3 |
| 9.12252907077763e-248 | 1.8777232539512 | 0.253 | 0.089 | 3.29113481286445e-243 | M2 macrophages | CD36 |
| 3.18135471579264e-247 | 1.125250814 | 0.537 | 0.321 | 1.14773734081651e-242 | M2 macrophages | GYPC |
| 2.72921817616511e-244 | 1.72899627745979 | 0.268 | 0.096 | 9.84620041415085e-240 | M2 macrophages | IGF1 |
| 4.79700622568001e-243 | -2.075059777 | 0.237 | 0.435 | 1.73061593603858e-238 | M2 macrophages | ALCAM |
| 4.70399691650544e-240 | 2.12231462006416 | 0.32 | 0.141 | 1.69706096756767e-235 | M2 macrophages | CCL2 |
| 8.53732407190367e-240 | -2.875904809 | 0.094 | 0.28 | 3.08001040542069e-235 | M2 macrophages | IL1B |
| 4.56943624441927e-237 | 1.44084509695156 | 0.393 | 0.195 | 1.64851551389914e-232 | M2 macrophages | SCN9A |
| 1.5328135409784e-236 | 1.35935843289686 | 0.539 | 0.331 | 5.52993141178778e-232 | M2 macrophages | MAMDC2 |
| 3.56503520871793e-234 | -0.636263234 | 0.978 | 0.985 | 1.28615775224917e-229 | M2 macrophages | MALAT1 |
| 1.07749128556821e-227 | -1.535725367 | 0.377 | 0.548 | 3.88726531094442e-223 | M2 macrophages | DENND5A |
| 6.0012114882067e-223 | 1.22787922176345 | 0.569 | 0.366 | 2.16505706860033e-218 | M2 macrophages | CTSC |
| 1.44086203897168e-217 | 0.652966954150234 | 0.917 | 0.838 | 5.19819797799815e-213 | M2 macrophages | DAB2 |
| 1.03899209249935e-215 | -1.912594054 | 0.148 | 0.339 | 3.74837177210992e-211 | M2 macrophages | LUCAT1 |
| 1.72473380835107e-215 | -1.039350832 | 0.477 | 0.636 | 6.22232216038814e-211 | M2 macrophages | TANC2 |
| 5.35241277599478e-215 | 0.960167435179937 | 0.594 | 0.42 | 1.93098995719564e-210 | M2 macrophages | ALOX5AP |
| 1.68568186038851e-214 | 1.17970552393452 | 0.425 | 0.226 | 6.08143444772363e-210 | M2 macrophages | LTC4S |
| 5.63669221287399e-206 | -1.219089803 | 0.572 | 0.691 | 2.03354944963855e-201 | M2 macrophages | CHST11 |
| 2.38226981952373e-200 | -1.074066268 | 0.565 | 0.693 | 8.59451482789578e-196 | M2 macrophages | KCNMA1 |
| 4.46307270849226e-200 | 0.992820397079816 | 0.589 | 0.411 | 1.61014274104275e-195 | M2 macrophages | PEPD |
| 3.36632062241969e-199 | -1.058369197 | 0.434 | 0.595 | 1.21446749095035e-194 | M2 macrophages | SLC11A1 |
| 3.2280949527086e-198 | 1.49155989726274 | 0.338 | 0.166 | 1.16459981608868e-193 | M2 macrophages | ABCA6 |
| 3.62435091421321e-198 | 0.699386828514713 | 0.76 | 0.637 | 1.3075570793207e-193 | M2 macrophages | PMP22 |
| 1.00708585653903e-196 | 0.649654381568524 | 0.864 | 0.821 | 3.63326364463586e-192 | M2 macrophages | CFD |
| 1.02000767334144e-195 | -1.259103054 | 0.292 | 0.466 | 3.6798816831139e-191 | M2 macrophages | FGR |
| 5.77083781436993e-194 | 0.960930195663604 | 0.659 | 0.481 | 2.08194515829024e-189 | M2 macrophages | MAF |
| 6.53726759457853e-193 | -1.808325438 | 0.108 | 0.28 | 2.3584500300961e-188 | M2 macrophages | BCL2 |
| 7.739554355353e-193 | -0.904301165 | 0.708 | 0.794 | 2.7921990247807e-188 | M2 macrophages | MSR1 |
| 3.05953742659211e-189 | -2.055462284 | 0.193 | 0.363 | 1.10378931739163e-184 | M2 macrophages | KCNQ3 |
| 3.78111160898723e-189 | 0.598443312784143 | 0.809 | 0.722 | 1.36411163517432e-184 | M2 macrophages | CALM2 |
| 2.73478907847452e-186 | -1.45972263 | 0.41 | 0.568 | 9.86629855841253e-182 | M2 macrophages | NFKB1 |
| 5.48938215758529e-185 | 0.655081255090168 | 0.833 | 0.798 | 1.98040440099205e-180 | M2 macrophages | C1QB |
| 5.31925783284248e-182 | -1.908054099 | 0.129 | 0.294 | 1.91902864835458e-177 | M2 macrophages | ZNF804A |
| 1.67942558802819e-180 | 0.667533492683516 | 0.686 | 0.539 | 6.05886369392929e-176 | M2 macrophages | RNASET2 |
| 3.06346491469453e-180 | -1.441213841 | 0.153 | 0.326 | 1.10520623727434e-175 | M2 macrophages | PCNX2 |
| 4.54985388895509e-179 | -1.644095907 | 0.214 | 0.381 | 1.64145078751833e-174 | M2 macrophages | ANPEP |
| 1.00081108774775e-178 | -1.121105979 | 0.447 | 0.586 | 3.61062616126756e-174 | M2 macrophages | NUMB |
| 2.15917143159291e-176 | -3.362434474 | 0.104 | 0.256 | 7.78964277375774e-172 | M2 macrophages | SLC39A8 |
| 2.4476206631953e-175 | 0.96315050058571 | 0.444 | 0.259 | 8.83028106660967e-171 | M2 macrophages | GPR34 |
| 5.31629579286396e-175 | 0.889659300919992 | 0.502 | 0.326 | 1.91796003319153e-170 | M2 macrophages | FUCA1 |
| 2.83658371790429e-174 | -1.880649818 | 0.139 | 0.303 | 1.02335430790833e-169 | M2 macrophages | PPARG |
| 2.74371424049297e-170 | 0.532162839035086 | 0.851 | 0.836 | 9.89849786542649e-166 | M2 macrophages | CST3 |
| 1.51163937150496e-169 | -0.912769866 | 0.592 | 0.696 | 5.45354136057846e-165 | M2 macrophages | TAOK3 |
| 9.55264442782484e-167 | 1.45133143716592 | 0.279 | 0.131 | 3.44630753022637e-162 | M2 macrophages | CCL18 |
| 8.35793106123995e-166 | -0.896746761 | 0.479 | 0.63 | 3.01529078896354e-161 | M2 macrophages | FGD4 |
| 9.15436163940185e-166 | 1.414528406 | 0.337 | 0.182 | 3.30261904864701e-161 | M2 macrophages | HRH1 |
| 2.43744510232543e-164 | 0.795155310494963 | 0.569 | 0.401 | 8.79357069565947e-160 | M2 macrophages | CREG1 |
| 3.76715308415297e-162 | -0.963272397 | 0.486 | 0.605 | 1.35907581816987e-157 | M2 macrophages | LHFPL2 |
| 8.26803843994061e-161 | -1.133948144 | 0.582 | 0.674 | 2.98286022797737e-156 | M2 macrophages | FNDC3B |
| 7.14008230135059e-160 | 0.774535086105389 | 0.572 | 0.402 | 2.57592749185825e-155 | M2 macrophages | TSPAN4 |
| 1.37621366941078e-159 | 1.07393396320828 | 0.529 | 0.363 | 4.96496605513328e-155 | M2 macrophages | IQGAP2 |
| 2.32355601747016e-159 | 1.04728802088814 | 0.452 | 0.283 | 8.38269304422708e-155 | M2 macrophages | FCGR2B |
| 5.29689685181046e-158 | 0.689365888781888 | 0.705 | 0.562 | 1.91096147722766e-153 | M2 macrophages | SNX6 |
| 8.44902178477153e-158 | 1.14113664599681 | 0.484 | 0.322 | 3.04815358929203e-153 | M2 macrophages | NRP1 |
| 1.13983300766417e-157 | -1.090444714 | 0.415 | 0.54 | 4.11217554175002e-153 | M2 macrophages | CD58 |
| 7.04663983463187e-157 | 0.57088131482125 | 0.881 | 0.891 | 2.54221625314014e-152 | M2 macrophages | B2M |
| 2.32137000317643e-156 | -1.377653957 | 0.213 | 0.371 | 8.37480656045961e-152 | M2 macrophages | SNTB1 |
| 4.49491353373958e-156 | -1.010969404 | 0.458 | 0.581 | 1.62162995556723e-151 | M2 macrophages | GPCPD1 |
| 2.67666936614853e-155 | 0.713936644276768 | 0.749 | 0.634 | 9.65662007225406e-151 | M2 macrophages | CD14 |
| 1.70735170856108e-150 | -1.458113499 | 0.258 | 0.406 | 6.15961275897581e-146 | M2 macrophages | PDE3A |
| 1.91978460464105e-148 | 0.505725470616559 | 0.797 | 0.773 | 6.92600691816353e-144 | M2 macrophages | NPC2 |
| 2.00053331478583e-148 | -0.81541129 | 0.686 | 0.757 | 7.21732403975285e-144 | M2 macrophages | DST |
| 1.32798614392607e-147 | -1.787626297 | 0.174 | 0.326 | 4.79097561144207e-143 | M2 macrophages | MAILR |
| 2.61389012434878e-144 | 0.588014130184321 | 0.73 | 0.663 | 9.4301314016131e-140 | M2 macrophages | HLA-E |
| 5.0912639159426e-144 | 1.09423484991384 | 0.391 | 0.232 | 1.83677528295461e-139 | M2 macrophages | MPEG1 |
| 4.15399705360722e-143 | 1.05205686359628 | 0.345 | 0.19 | 1.49863751702988e-138 | M2 macrophages | IGFBP4 |
| 8.44142412945686e-143 | 1.21976580886138 | 0.478 | 0.324 | 3.04541258318415e-138 | M2 macrophages | WWP1 |
| 2.76234324720517e-142 | -0.690243123 | 0.696 | 0.772 | 9.96570573294208e-138 | M2 macrophages | CYRIB |
| 3.88903264599671e-142 | 1.13612773261813 | 0.344 | 0.193 | 1.40304630769623e-137 | M2 macrophages | WLS |
| 2.54284704529865e-141 | -0.964264897 | 0.329 | 0.486 | 9.17382928532393e-137 | M2 macrophages | NFAT5 |
| 7.11083620988841e-136 | 0.836158504433876 | 0.453 | 0.291 | 2.56537637944144e-131 | M2 macrophages | IFITM3 |
| 1.00664019117672e-135 | -0.77977515 | 0.524 | 0.636 | 3.63165581770826e-131 | M2 macrophages | ABR |
| 1.03752264105519e-135 | 0.734885287026854 | 0.59 | 0.442 | 3.74307043213481e-131 | M2 macrophages | NINJ1 |
| 1.13512835488336e-135 | -1.620193987 | 0.242 | 0.38 | 4.09520256591269e-131 | M2 macrophages | ENSG00000253496 |
| 2.14162119298609e-135 | -1.218450706 | 0.195 | 0.34 | 7.72632677793591e-131 | M2 macrophages | SORL1 |
| 3.08833946275868e-135 | 0.902772220411373 | 0.328 | 0.175 | 1.11418022797945e-130 | M2 macrophages | TMEM37 |
| 5.75683722412532e-135 | -0.928867563 | 0.4 | 0.534 | 2.07689416534769e-130 | M2 macrophages | RAPGEF1 |
| 1.16576696653411e-134 | -0.739872304 | 0.593 | 0.709 | 4.20573748516511e-130 | M2 macrophages | APBB1IP |
| 1.53060652723519e-134 | -1.265121449 | 0.219 | 0.363 | 5.5219691683064e-130 | M2 macrophages | FERMT2 |
| 1.02686508630571e-132 | 0.622297747888603 | 0.748 | 0.585 | 3.70462117186511e-128 | M2 macrophages | COLEC12 |
| 6.52525650761722e-132 | 0.908034086184615 | 0.397 | 0.237 | 2.35411679025307e-127 | M2 macrophages | NEU1 |
| 8.36708267992889e-132 | 1.19874119476164 | 0.633 | 0.515 | 3.01859241843795e-127 | M2 macrophages | PDE4D |
| 1.38518925573523e-131 | -1.448338582 | 0.172 | 0.313 | 4.99734727791598e-127 | M2 macrophages | NIBAN1 |
| 2.50480472550197e-129 | -1.519598703 | 0.259 | 0.392 | 9.03658400819345e-125 | M2 macrophages | SAMSN1 |
| 2.62949039415691e-129 | -0.848328195 | 0.655 | 0.731 | 9.48641249499989e-125 | M2 macrophages | MAML2 |
| 4.57491179729149e-129 | 0.514056344000653 | 0.761 | 0.704 | 1.65049092910885e-124 | M2 macrophages | FCER1G |
| 7.11371966908382e-127 | -0.8359449 | 0.449 | 0.573 | 2.56641664501537e-122 | M2 macrophages | RASAL2 |
| 5.35939203901577e-126 | -1.129579603 | 0.314 | 0.457 | 1.93350786591572e-121 | M2 macrophages | FNIP2 |
| 8.74258804328072e-126 | -1.393907435 | 0.23 | 0.368 | 3.15406348837438e-121 | M2 macrophages | TLR2 |
| 1.59041084308176e-125 | -0.626333187 | 0.661 | 0.747 | 5.73772519858606e-121 | M2 macrophages | RASGEF1B |
| 1.71434149401586e-123 | -0.728321839 | 0.769 | 0.817 | 6.18482980796101e-119 | M2 macrophages | MBNL1 |
| 4.68232519422057e-123 | -0.81571792 | 0.65 | 0.729 | 1.68924246031896e-118 | M2 macrophages | DPYD |
| 5.25599608443622e-123 | -1.143025835 | 0.304 | 0.442 | 1.89620570738205e-118 | M2 macrophages | TNFAIP3 |
| 5.88197405972176e-123 | 0.536225390788082 | 0.778 | 0.723 | 2.12203978152582e-118 | M2 macrophages | CD99 |
| 7.76965403775722e-122 | 1.07342760615154 | 0.257 | 0.128 | 2.80305808720167e-117 | M2 macrophages | GATM |
| 1.79950051234737e-120 | -1.388260552 | 0.133 | 0.266 | 6.49205799839561e-116 | M2 macrophages | MIR181A1HG |
| 6.98197990711899e-120 | -1.231051232 | 0.184 | 0.322 | 2.51888889109132e-115 | M2 macrophages | USP12 |
| 1.02988147038274e-119 | -1.076901566 | 0.361 | 0.49 | 3.7155033806998e-115 | M2 macrophages | ELL2 |
| 1.19558453408638e-119 | -0.604225013 | 0.703 | 0.766 | 4.31331032362344e-115 | M2 macrophages | CAMK1D |
| 4.69290455026799e-119 | -1.270337988 | 0.218 | 0.354 | 1.69305917460018e-114 | M2 macrophages | OXSR1 |
| 1.62323571035748e-118 | 0.722851683365271 | 0.64 | 0.494 | 5.85614747225667e-114 | M2 macrophages | CSF1R |
| 1.8823820930028e-118 | -1.259707009 | 0.338 | 0.464 | 6.79106987692621e-114 | M2 macrophages | CAMK2D |
| 2.03377087945678e-118 | -1.247612163 | 0.207 | 0.345 | 7.33723520181621e-114 | M2 macrophages | HIVEP1 |
| 6.07818800776731e-118 | -1.062985068 | 0.269 | 0.404 | 2.19282788756221e-113 | M2 macrophages | IRAK3 |
| 1.60081944722078e-117 | 0.592042022312721 | 0.655 | 0.522 | 5.77527631973842e-113 | M2 macrophages | SNX2 |
| 9.34743142292234e-115 | -0.73158827 | 0.451 | 0.571 | 3.37227283444769e-110 | M2 macrophages | TRIO |
| 1.11662379443517e-114 | -0.647506879 | 0.518 | 0.638 | 4.02844366318376e-110 | M2 macrophages | FLNA |
| 5.42936336020046e-114 | -0.732633751 | 0.562 | 0.659 | 1.95875141945952e-109 | M2 macrophages | ATXN1 |
| 2.33274545463662e-113 | -1.109288544 | 0.201 | 0.336 | 8.41584577669253e-109 | M2 macrophages | CSGALNACT2 |
| 9.35807548453717e-113 | 0.774022570088781 | 0.415 | 0.261 | 3.37611289255647e-108 | M2 macrophages | NCF4 |
| 1.93715244383302e-112 | -0.928378702 | 0.516 | 0.605 | 6.98866487161637e-108 | M2 macrophages | PLAUR |
| 1.16002679276798e-111 | 0.477329158787644 | 0.785 | 0.725 | 4.18502866026905e-107 | M2 macrophages | UBC |
| 1.47188568784248e-111 | -0.823468527 | 0.49 | 0.598 | 5.31012199602933e-107 | M2 macrophages | PTPRJ |
| 1.8350614654394e-111 | -0.799650308 | 0.37 | 0.494 | 6.62035124886571e-107 | M2 macrophages | ADK |
| 3.6090797171792e-111 | 1.09335764416555 | 0.449 | 0.309 | 1.30204768956674e-106 | M2 macrophages | RGL1 |
| 6.03419906491157e-111 | -0.850984856 | 0.296 | 0.43 | 2.17695799664815e-106 | M2 macrophages | TIAM1 |
| 9.5408935281187e-111 | -0.684990692 | 0.483 | 0.601 | 3.44206815813938e-106 | M2 macrophages | RABGAP1L |
| 1.50392839636736e-110 | 0.511108019721462 | 0.621 | 0.481 | 5.42572247557452e-106 | M2 macrophages | CPVL |
| 1.5610145657587e-110 | -0.551416057 | 0.705 | 0.777 | 5.63167224888766e-106 | M2 macrophages | MEF2A |
| 2.80609761114067e-110 | -0.700976828 | 0.486 | 0.591 | 1.01235583517122e-105 | M2 macrophages | CUX1 |
| 5.26213726539538e-110 | 0.868224448312914 | 0.375 | 0.237 | 1.89842126123669e-105 | M2 macrophages | SERPING1 |
| 1.46905780120574e-109 | -1.154636106 | 0.194 | 0.327 | 5.29991982940996e-105 | M2 macrophages | ATP13A3 |
| 1.55286866263692e-109 | 0.739340071134937 | 0.411 | 0.263 | 5.6022842741952e-105 | M2 macrophages | EPHX1 |
| 1.64874678268388e-109 | -0.522715632 | 0.701 | 0.768 | 5.94818376788864e-105 | M2 macrophages | GNAQ |
| 3.79196918242653e-109 | -1.034441072 | 0.207 | 0.341 | 1.36802872194402e-104 | M2 macrophages | NAV1 |
| 8.23089491057855e-109 | -0.606706693 | 0.855 | 0.881 | 2.96945995688943e-104 | M2 macrophages | ZEB2 |
| 8.90946126118003e-109 | 1.2095449866106 | 0.326 | 0.196 | 3.21426633919592e-104 | M2 macrophages | PDK4 |
| 1.18242222374747e-108 | 0.718348069599255 | 0.397 | 0.251 | 4.26582465661373e-104 | M2 macrophages | IFITM2 |
| 1.36776539432327e-108 | 1.03417383681299 | 0.579 | 0.447 | 4.93448721310006e-104 | M2 macrophages | ITSN1 |
| 1.60887209975628e-108 | -1.039857173 | 0.574 | 0.642 | 5.80432787429073e-104 | M2 macrophages | SOD2 |
| 5.09765484413126e-108 | -0.914092195 | 0.453 | 0.551 | 1.83908093811723e-103 | M2 macrophages | CLIC4 |
| 1.6226763724494e-107 | -1.266333856 | 0.246 | 0.373 | 5.85412954888569e-103 | M2 macrophages | ACSL1 |
| 2.19067141886308e-107 | 0.771886356162966 | 0.381 | 0.239 | 7.90328527783234e-103 | M2 macrophages | GLMP |
| 1.8720633644445e-106 | -0.987951613 | 0.183 | 0.315 | 6.75384299990641e-102 | M2 macrophages | ZBTB7C |
| 1.01942788382446e-105 | -0.944506267 | 0.375 | 0.486 | 3.67778997647351e-101 | M2 macrophages | DSE |
| 3.18214833139828e-105 | -1.269666297 | 0.13 | 0.252 | 1.14802365351856e-100 | M2 macrophages | XYLT1 |
| 6.18022228935274e-105 | 0.56655786938004 | 0.606 | 0.474 | 2.22963879532979e-100 | M2 macrophages | LAMP1 |
| 8.25735788115389e-105 | 1.01612823415714 | 0.352 | 0.22 | 2.97900700278389e-100 | M2 macrophages | GNG2 |
| 8.43976128323382e-104 | 0.529902338069767 | 0.59 | 0.453 | 3.04481267815227e-99 | M2 macrophages | CD59 |
| 8.61715779561015e-104 | 0.472188739809605 | 0.735 | 0.642 | 3.10881201792227e-99 | M2 macrophages | PLD3 |
| 1.68568752499075e-103 | 0.677599345709221 | 0.46 | 0.308 | 6.08145488390913e-99 | M2 macrophages | HPGDS |
| 1.89524795876209e-103 | 0.509427620490371 | 0.776 | 0.743 | 6.837486060826e-99 | M2 macrophages | HLA-B |
| 1.99470422734757e-103 | 0.496690018375563 | 0.76 | 0.719 | 7.19629444100182e-99 | M2 macrophages | CD68 |
| 3.98462606118466e-103 | 0.615187324720116 | 0.726 | 0.671 | 1.43753354409359e-98 | M2 macrophages | HLA-C |
| 2.22164907873988e-102 | 0.460168798938983 | 0.779 | 0.68 | 8.01504338136985e-98 | M2 macrophages | TGFBI |
| 4.48653024363149e-102 | 0.818773019500559 | 0.273 | 0.147 | 1.61860551599493e-97 | M2 macrophages | SULT1A1 |
| 1.21377180199551e-101 | 0.75749797323534 | 0.401 | 0.259 | 4.37892453005921e-97 | M2 macrophages | GNPDA1 |
| 9.99779312457973e-101 | -1.133498046 | 0.218 | 0.34 | 3.60690382555463e-96 | M2 macrophages | P2RX7 |
| 3.75446337324043e-99 | -0.91757992 | 0.597 | 0.671 | 1.35449775116395e-94 | M2 macrophages | SLC8A1 |
| 4.25678173881635e-99 | -0.521753584 | 0.727 | 0.776 | 1.53571914791277e-94 | M2 macrophages | RAP1B |
| 5.83130296105706e-99 | 0.529931885912149 | 0.524 | 0.369 | 2.10375916926056e-94 | M2 macrophages | TPM1 |
| 9.16730421668332e-99 | -1.156432397 | 0.17 | 0.294 | 3.30728834225284e-94 | M2 macrophages | NOTCH2NLC |
| 3.45484867951017e-98 | -1.096237458 | 0.199 | 0.32 | 1.24640575810688e-93 | M2 macrophages | MTHFD1L |
| 2.65505564090295e-97 | 0.671886287338596 | 0.454 | 0.315 | 9.57864423568558e-93 | M2 macrophages | AKR1B1 |
| 1.27985647099427e-96 | -0.56243318 | 0.616 | 0.687 | 4.61733819040604e-92 | M2 macrophages | CCDC88A |
| 1.80000756410134e-96 | -0.905668309 | 0.272 | 0.393 | 6.49388728900841e-92 | M2 macrophages | MYO1G |
| 1.99948521274774e-96 | 0.697112707949974 | 0.431 | 0.289 | 7.21354280203004e-92 | M2 macrophages | BLVRA |
| 4.24395612592966e-96 | -1.02434341 | 0.394 | 0.493 | 1.53109205155164e-91 | M2 macrophages | ZNF438 |
| 5.04178459855069e-96 | -0.969568569 | 0.328 | 0.441 | 1.81892462961913e-91 | M2 macrophages | FAM107B |
| 6.16835457469235e-96 | -0.751073257 | 0.433 | 0.541 | 2.22535727991176e-91 | M2 macrophages | PRKAG2 |
| 1.4825315244911e-95 | -0.668629236 | 0.421 | 0.534 | 5.34852898090654e-91 | M2 macrophages | PSEN1 |
| 1.58704669430703e-95 | 0.741753924089638 | 0.38 | 0.249 | 5.72558835905149e-91 | M2 macrophages | CTSK |
| 2.21274364374909e-95 | -0.614950822 | 0.586 | 0.672 | 7.98291524355357e-91 | M2 macrophages | UBE2E2 |
| 2.71253300011098e-95 | -0.403512049 | 0.89 | 0.928 | 9.7860053045004e-91 | M2 macrophages | MT-CO2 |
| 3.02510148550178e-95 | 1.15794811512215 | 0.358 | 0.233 | 1.09136586292448e-90 | M2 macrophages | FAM20A |
| 4.15212458738681e-95 | -1.549595179 | 0.213 | 0.332 | 1.49796198739154e-90 | M2 macrophages | SLC16A10 |
| 5.1124540442647e-95 | 0.451368022806996 | 0.765 | 0.712 | 1.84442004554937e-90 | M2 macrophages | H3-3B |
| 7.29037900214597e-95 | 0.673563666976166 | 0.508 | 0.369 | 2.6301500326042e-90 | M2 macrophages | TNFRSF1A |
| 1.86738871842175e-94 | -0.706702235 | 0.39 | 0.505 | 6.73697827945015e-90 | M2 macrophages | DIAPH1 |
| 5.59488065283668e-94 | -1.08463062 | 0.24 | 0.364 | 2.01846509312389e-89 | M2 macrophages | HIVEP2 |
| 6.07252610173441e-94 | 0.584899535324186 | 0.543 | 0.4 | 2.19078524172272e-89 | M2 macrophages | UCP2 |
| 6.96951986261587e-94 | 0.676982525196482 | 0.282 | 0.158 | 2.51439368083593e-89 | M2 macrophages | FKBP2 |
| 1.08841130306254e-93 | 0.77647259201118 | 0.644 | 0.508 | 3.92666145805873e-89 | M2 macrophages | AP2A2 |
| 3.28532769989161e-93 | -0.613582897 | 0.432 | 0.542 | 1.1852476742899e-88 | M2 macrophages | SKIL |
| 1.34086195860691e-92 | 0.530750410250242 | 0.667 | 0.543 | 4.83742768806615e-88 | M2 macrophages | A2M |
| 3.43458777869243e-92 | -0.925601537 | 0.397 | 0.493 | 1.23909623291887e-87 | M2 macrophages | TBC1D12 |
| 1.233074517378e-91 | -0.564241738 | 0.643 | 0.707 | 4.44856293634462e-87 | M2 macrophages | ZFAND3 |
| 1.25374171864103e-91 | -1.202314474 | 0.164 | 0.276 | 4.52312399834126e-87 | M2 macrophages | HIPK2 |
| 2.05673115828654e-91 | -0.425225564 | 0.613 | 0.743 | 7.42006899975034e-87 | M2 macrophages | TYMP |
| 2.82496407908485e-91 | 0.568757172761865 | 0.597 | 0.474 | 1.01916229081144e-86 | M2 macrophages | CD302 |
| 3.10835193116511e-91 | -0.97868366 | 0.265 | 0.385 | 1.12140012620644e-86 | M2 macrophages | MYO1E |
| 4.14829695955426e-91 | -0.602546659 | 0.395 | 0.513 | 1.49658109409839e-86 | M2 macrophages | CMIP |
| 1.71471461102402e-90 | 0.461897047698353 | 0.64 | 0.531 | 6.18617590219136e-86 | M2 macrophages | CLTA |
| 2.52264772391215e-90 | -0.70578922 | 0.527 | 0.606 | 9.10095619355785e-86 | M2 macrophages | PDE4DIP |
| 2.6731881861301e-90 | -0.576179539 | 0.629 | 0.707 | 9.64406101910155e-86 | M2 macrophages | DLEU2 |
| 2.75659247242939e-90 | -0.856046633 | 0.33 | 0.445 | 9.94495866278352e-86 | M2 macrophages | ARHGAP31 |
| 1.46428694869739e-89 | 0.901938755289044 | 0.317 | 0.195 | 5.28270802481558e-85 | M2 macrophages | CITED2 |
| 5.15548051897623e-89 | -0.736003682 | 0.352 | 0.473 | 1.85994270683105e-84 | M2 macrophages | PBX3 |
| 1.15806505217237e-88 | -0.685816744 | 0.433 | 0.538 | 4.17795128872225e-84 | M2 macrophages | PTPRM |
| 1.20628559765194e-88 | 0.669199247098971 | 0.421 | 0.29 | 4.35191655064892e-84 | M2 macrophages | TCN2 |
| 1.44678186495784e-88 | -1.458136144 | 0.153 | 0.261 | 5.21955493420841e-84 | M2 macrophages | MADD |
| 2.47822434953664e-88 | -0.550335047 | 0.616 | 0.682 | 8.94068998582332e-84 | M2 macrophages | FPR3 |
| 3.10439587098885e-88 | -0.474869015 | 0.894 | 0.925 | 1.11997289837665e-83 | M2 macrophages | MT-ATP6 |
| 1.10667988158006e-87 | 0.518884506948475 | 0.618 | 0.498 | 3.9925690087764e-83 | M2 macrophages | HSPA8 |
| 1.98669759914479e-87 | -1.139587444 | 0.274 | 0.385 | 7.16740892843468e-83 | M2 macrophages | CXCL3 |
| 5.0700768676283e-87 | 0.667637643235303 | 0.425 | 0.289 | 1.82913163153426e-82 | M2 macrophages | MCOLN1 |
| 6.25313881020978e-87 | -0.814718861 | 0.375 | 0.486 | 2.25594488855938e-82 | M2 macrophages | ABL2 |
| 7.26601426833838e-87 | -0.6374416 | 0.526 | 0.617 | 2.62135996758844e-82 | M2 macrophages | UTRN |
| 1.71795566632124e-86 | -0.755298046 | 0.363 | 0.465 | 6.19786865738715e-82 | M2 macrophages | MYOF |
| 1.6900900108041e-85 | -0.688003604 | 0.433 | 0.529 | 6.09733773197795e-81 | M2 macrophages | ARHGAP10 |
| 2.75410831958317e-85 | 0.542904729502146 | 0.564 | 0.451 | 9.93599658456019e-81 | M2 macrophages | HLA-DMB |
| 3.47531733130402e-85 | -0.798564642 | 0.366 | 0.473 | 1.25379023361455e-80 | M2 macrophages | PITPNA |
| 4.6199384802964e-85 | -0.836843306 | 0.591 | 0.651 | 1.66673520553653e-80 | M2 macrophages | ATP2B1 |
| 1.40929682850765e-84 | -1.068070704 | 0.285 | 0.39 | 5.08432016820706e-80 | M2 macrophages | PTPN1 |
| 4.35385917550645e-84 | 0.653793559065847 | 0.402 | 0.268 | 1.57074177474746e-79 | M2 macrophages | TECR |
| 9.62859158946274e-84 | 0.589814786369472 | 0.492 | 0.361 | 3.47370698773047e-79 | M2 macrophages | SCAMP2 |
| 1.02914562216228e-83 | 0.515088861309475 | 0.547 | 0.426 | 3.71284866107485e-79 | M2 macrophages | TMEM59 |
| 2.37901672244961e-83 | 0.624703272835993 | 0.343 | 0.213 | 8.58277862958145e-79 | M2 macrophages | GIMAP4 |
| 6.05448573402051e-83 | -0.590355008 | 0.511 | 0.601 | 2.18427681826258e-78 | M2 macrophages | DOCK8 |
| 9.97138915042655e-83 | 0.474560642760347 | 0.66 | 0.588 | 3.59737806379939e-78 | M2 macrophages | ASAH1 |
| 1.41117935524134e-82 | -1.016192261 | 0.17 | 0.278 | 5.09111175990417e-78 | M2 macrophages | GPD2 |
| 5.74442895119805e-82 | 0.585714700545507 | 0.371 | 0.241 | 2.07241763272372e-77 | M2 macrophages | RENBP |
| 9.58446170803804e-82 | 0.467985006456888 | 0.441 | 0.301 | 3.45778625040888e-77 | M2 macrophages | ALDOA |
| 1.22867452405507e-81 | -0.628339502 | 0.315 | 0.44 | 4.43268908043348e-77 | M2 macrophages | ARHGAP15 |
| 2.53054106504464e-80 | 1.14022824716772 | 0.528 | 0.443 | 9.12943300036154e-76 | M2 macrophages | MAN1A1 |
| 3.63002379847466e-80 | -0.727895098 | 0.51 | 0.591 | 1.3096036857757e-75 | M2 macrophages | EPB41L3 |
| 4.11097162994056e-80 | -0.746128183 | 0.564 | 0.632 | 1.48311523493366e-75 | M2 macrophages | DAPK1 |
| 8.93414830288946e-80 | -0.551813767 | 0.56 | 0.64 | 3.22317268323343e-75 | M2 macrophages | EXOC4 |
| 1.08088811658115e-79 | -1.156847057 | 0.18 | 0.283 | 3.8995200581898e-75 | M2 macrophages | CYTIP |
| 3.46847215940424e-79 | -0.987865955 | 0.213 | 0.321 | 1.25132070094827e-74 | M2 macrophages | ZNF267 |
| 4.60634072061515e-79 | -0.850374751 | 0.457 | 0.543 | 1.66182954177633e-74 | M2 macrophages | DMXL2 |
| 4.76394463097544e-79 | -0.473179842 | 0.863 | 0.899 | 1.71868830451701e-74 | M2 macrophages | MT-ND4 |
| 8.01346331998056e-79 | -1.083438858 | 0.184 | 0.29 | 2.89101716194939e-74 | M2 macrophages | DDX60L |
| 2.19820219637263e-78 | -1.277658493 | 0.286 | 0.377 | 7.93045406385353e-74 | M2 macrophages | DRAM1 |
| 5.32526602756945e-78 | -1.017873226 | 0.246 | 0.349 | 1.92119622476623e-73 | M2 macrophages | ITGA5 |
| 1.11356180043831e-77 | 0.618965105836929 | 0.41 | 0.279 | 4.01739690744131e-73 | M2 macrophages | CHID1 |
| 1.56565158934473e-77 | 0.408831651719794 | 0.773 | 0.678 | 5.648401238879e-73 | M2 macrophages | LILRB5 |
| 1.76216594704234e-77 | -1.006246253 | 0.207 | 0.308 | 6.35736608714464e-73 | M2 macrophages | ECE1 |
| 4.06617879621179e-77 | 0.511918101304896 | 0.443 | 0.308 | 1.46695532430933e-72 | M2 macrophages | TMEM14C |
| 4.60926936719646e-77 | -0.707416015 | 0.455 | 0.54 | 1.66288610960347e-72 | M2 macrophages | MAP3K8 |
| 5.92472740349483e-77 | -1.266930098 | 0.21 | 0.312 | 2.13746390535883e-72 | M2 macrophages | SLC1A3 |
| 1.41031849008592e-76 | 0.493077022782612 | 0.557 | 0.453 | 5.08800601668296e-72 | M2 macrophages | COX6A1 |
| 1.65123527844166e-76 | 0.438556797369972 | 0.767 | 0.701 | 5.95716151403399e-72 | M2 macrophages | MS4A7 |
| 2.06436593076044e-76 | -0.938865102 | 0.177 | 0.286 | 7.44761296840443e-72 | M2 macrophages | VCAN |
| 2.14105456474473e-75 | -1.005218988 | 0.254 | 0.357 | 7.72428255322956e-71 | M2 macrophages | HIVEP3 |
| 2.38069150041584e-75 | 0.647216279277683 | 0.268 | 0.157 | 8.58882072605022e-71 | M2 macrophages | DNPH1 |
| 6.31114292847715e-75 | -0.910151574 | 0.199 | 0.307 | 2.2768710343067e-70 | M2 macrophages | ATP2C1 |
| 2.74864053504405e-74 | 1.03030993099678 | 0.305 | 0.2 | 9.91627045827843e-70 | M2 macrophages | EPB41L1 |
| 5.61104141863596e-74 | 0.962291634069379 | 0.369 | 0.257 | 2.0242954126013e-69 | M2 macrophages | TBC1D14 |
| 8.50455944327716e-74 | 0.367643321539439 | 0.7 | 0.633 | 3.0681899103511e-69 | M2 macrophages | ATP6V0C |
| 1.03110871272324e-73 | -0.629779145 | 0.415 | 0.51 | 3.71993090289162e-69 | M2 macrophages | CCNY |
| 1.18445435941142e-73 | -0.846957145 | 0.352 | 0.454 | 4.27315599244857e-69 | M2 macrophages | MITF |
| 3.44149237961627e-73 | 0.622139362202907 | 0.338 | 0.218 | 1.24158720579416e-68 | M2 macrophages | CAT |
| 5.56737686355877e-73 | -1.258656094 | 0.229 | 0.328 | 2.0085425510661e-68 | M2 macrophages | RNF144B |
| 1.23667043217992e-72 | -0.73183393 | 0.372 | 0.472 | 4.46153591817551e-68 | M2 macrophages | FNDC3A |
| 1.51118297819652e-72 | -0.939576742 | 0.193 | 0.293 | 5.45189483043958e-68 | M2 macrophages | VCL |
| 2.02957517399672e-72 | -1.482721083 | 0.181 | 0.277 | 7.32209835522798e-68 | M2 macrophages | GPR183 |
| 4.0316410783387e-72 | 0.446434323738987 | 0.68 | 0.633 | 1.45449515183225e-67 | M2 macrophages | HLA-DMA |
| 6.14130433931033e-72 | -0.843842029 | 0.305 | 0.401 | 2.21559836649299e-67 | M2 macrophages | LPXN |
| 6.93960213183252e-72 | -0.808733156 | 0.222 | 0.325 | 2.50360026110122e-67 | M2 macrophages | VRK2 |
| 8.29125170027299e-72 | 0.700746761173605 | 0.748 | 0.696 | 2.99123487590749e-67 | M2 macrophages | FOS |
| 1.21274425539487e-71 | -1.48585735 | 0.186 | 0.285 | 4.37521745018808e-67 | M2 macrophages | ZFX |
| 2.30561609733657e-71 | 0.40976531798011 | 0.807 | 0.784 | 8.31797119436113e-67 | M2 macrophages | TYROBP |
| 2.86367917960228e-71 | -0.848657882 | 0.264 | 0.365 | 1.03312953762511e-66 | M2 macrophages | CIITA |
| 3.78487040964103e-71 | 0.582970052460594 | 0.289 | 0.176 | 1.36546769768619e-66 | M2 macrophages | TMEM35B |
| 5.66047318032877e-71 | 0.615931047743989 | 0.294 | 0.181 | 2.04212890926721e-66 | M2 macrophages | HEBP1 |
| 1.32005310063947e-70 | -0.663616738 | 0.406 | 0.491 | 4.76235557117702e-66 | M2 macrophages | MAPKAP1 |
| 5.57709573895717e-70 | 0.429437973881727 | 0.743 | 0.64 | 2.01204882974358e-65 | M2 macrophages | TXNIP |
| 9.420813191425e-70 | -0.778820993 | 0.388 | 0.49 | 3.3987467750704e-65 | M2 macrophages | TET2 |
| 1.05439250574485e-69 | 0.579063314516099 | 0.314 | 0.197 | 3.80393184297569e-65 | M2 macrophages | DDOST |
| 1.70673403341348e-69 | -0.755852015 | 0.388 | 0.475 | 6.15738437234583e-65 | M2 macrophages | UBAC2 |
| 2.0951900858625e-69 | -0.872443027 | 0.359 | 0.446 | 7.55881727276615e-65 | M2 macrophages | MDFIC |
| 2.84134248961538e-69 | -0.542712812 | 0.45 | 0.542 | 1.02507112997854e-64 | M2 macrophages | SSH2 |
| 3.62942862815295e-69 | -0.567261517 | 0.351 | 0.459 | 1.30938896617874e-64 | M2 macrophages | BTBD9 |
| 7.04960102520616e-69 | 0.564568307630254 | 0.336 | 0.218 | 2.54328456186363e-64 | M2 macrophages | ABRACL |
| 7.21769283361375e-69 | 0.500976489173589 | 0.567 | 0.443 | 2.60392704358283e-64 | M2 macrophages | BNIP3L |
| 8.11512644697428e-69 | -0.934875091 | 0.25 | 0.347 | 2.92769416827491e-64 | M2 macrophages | ITGAV |
| 8.57073491391699e-69 | 0.618344065089611 | 0.418 | 0.297 | 3.09206403489383e-64 | M2 macrophages | LAP3 |
| 1.04117163736281e-68 | -1.162816245 | 0.206 | 0.305 | 3.75623491611382e-64 | M2 macrophages | STK38L |
| 1.10451590882874e-68 | -0.788763547 | 0.278 | 0.376 | 3.98476204428145e-64 | M2 macrophages | SLC11A2 |
| 1.68486516076555e-68 | -0.582717095 | 0.543 | 0.616 | 6.07848804049388e-64 | M2 macrophages | SLC43A2 |
| 2.29917934037189e-68 | 0.525006746654786 | 0.425 | 0.305 | 8.29474930625965e-64 | M2 macrophages | GNPTG |
| 2.66226849387355e-68 | -0.624001174 | 0.412 | 0.501 | 9.60466604534761e-64 | M2 macrophages | PDLIM5 |
| 2.67173043307202e-68 | 0.433474799848911 | 0.502 | 0.373 | 9.63880188339391e-64 | M2 macrophages | HMGN2 |
| 1.74397606520344e-67 | 0.767172179991653 | 0.288 | 0.183 | 6.29174245043443e-63 | M2 macrophages | CNRIP1 |
| 3.67345621725068e-67 | -0.555898313 | 0.4 | 0.494 | 1.32527279949753e-62 | M2 macrophages | MAP3K2 |
| 1.51297242491832e-66 | -0.826359179 | 0.319 | 0.411 | 5.45835061737783e-62 | M2 macrophages | UBE2E1 |
| 1.53362093649239e-66 | 0.30965672748512 | 0.785 | 0.739 | 5.53284425258361e-62 | M2 macrophages | EIF1 |
| 1.56437083053465e-66 | 0.492744902674607 | 0.399 | 0.276 | 5.64378064531986e-62 | M2 macrophages | TMEM179B |
| 2.57975736827993e-66 | 0.686789263516547 | 0.419 | 0.299 | 9.3069906575435e-62 | M2 macrophages | SCARB1 |
| 6.6353474780014e-66 | 0.460740994317872 | 0.561 | 0.468 | 2.39383430963857e-61 | M2 macrophages | PPIB |
| 7.49502862829782e-66 | -0.760503213 | 0.403 | 0.48 | 2.70398147823101e-61 | M2 macrophages | ARL8B |
| 1.28484036800317e-65 | 0.973606105614877 | 0.26 | 0.165 | 4.63531859564504e-61 | M2 macrophages | ATP1B1 |
| 1.85214799390902e-65 | -0.792855988 | 0.178 | 0.276 | 6.68199431762556e-61 | M2 macrophages | RASA3 |
| 2.09782882334559e-65 | 0.499288598518947 | 0.401 | 0.28 | 7.56833704598387e-61 | M2 macrophages | TMED9 |
| 2.65265852384057e-65 | 0.852740770487189 | 0.307 | 0.206 | 9.56999615645963e-61 | M2 macrophages | FAM13A |
| 5.14509340722409e-65 | 0.514380747107363 | 0.448 | 0.324 | 1.85619534852424e-60 | M2 macrophages | CRYL1 |
| 1.45536546937721e-64 | -0.883753387 | 0.301 | 0.397 | 5.25052200387217e-60 | M2 macrophages | FABP5 |
| 1.99922171703628e-64 | -0.42545094 | 0.838 | 0.876 | 7.2125921885518e-60 | M2 macrophages | MT-ND2 |
| 2.56603404437332e-64 | -0.805248099 | 0.187 | 0.285 | 9.25748102188562e-60 | M2 macrophages | DOCK5 |
| 4.68093759506578e-64 | 0.562664112025246 | 0.3 | 0.192 | 1.68874185617188e-59 | M2 macrophages | CBR1 |
| 9.00431198112884e-64 | 0.535084023344501 | 0.483 | 0.367 | 3.24848563343185e-59 | M2 macrophages | NCOA4 |
| 1.03148520157064e-63 | 0.310317671429782 | 0.753 | 0.688 | 3.72128916170639e-59 | M2 macrophages | AIF1 |
| 1.43292899750053e-63 | -1.41633193 | 0.188 | 0.282 | 5.16957794428266e-59 | M2 macrophages | CCL3L3 |
| 1.44518156563508e-63 | 0.436807105322548 | 0.483 | 0.359 | 5.21378153434168e-59 | M2 macrophages | EID1 |
| 1.59800278171538e-63 | -0.649497378 | 0.523 | 0.598 | 5.76511463559457e-59 | M2 macrophages | NAMPT |
| 2.60105814076003e-63 | 0.570040988108384 | 0.389 | 0.272 | 9.38383745441995e-59 | M2 macrophages | EIF3D |
| 2.97931023811948e-63 | 0.390126729655497 | 0.669 | 0.61 | 1.07484575460637e-58 | M2 macrophages | ARPC5 |
| 3.59847962418041e-63 | 0.551077990608422 | 0.291 | 0.184 | 1.29822349401556e-58 | M2 macrophages | IDH1 |
| 6.01591869104825e-63 | 0.385436333545464 | 0.711 | 0.658 | 2.17036298616948e-58 | M2 macrophages | TAGLN2 |
| 7.94874340125146e-63 | -0.667539383 | 0.231 | 0.334 | 2.86766815686949e-58 | M2 macrophages | MAP4K3 |
| 2.04938079446915e-62 | 0.329251300992216 | 0.745 | 0.677 | 7.39355109220633e-58 | M2 macrophages | TPM3 |
| 2.05988280800299e-62 | -0.545036958 | 0.42 | 0.512 | 7.43143920643239e-58 | M2 macrophages | RAPH1 |
| 3.6402896756182e-62 | 0.47646982275451 | 0.54 | 0.432 | 1.31330730627278e-57 | M2 macrophages | LAPTM4A |
| 8.55838493228543e-62 | 0.512139452106106 | 0.387 | 0.272 | 3.08760853202062e-57 | M2 macrophages | PRDX3 |
| 1.25848500153406e-61 | 0.457871842257683 | 0.559 | 0.446 | 4.54023634003442e-57 | M2 macrophages | CIRBP |
| 1.43015450590175e-61 | 0.551801072749296 | 0.285 | 0.179 | 5.15956841094173e-57 | M2 macrophages | EMC4 |
| 1.4798409658356e-61 | -0.867600169 | 0.366 | 0.446 | 5.3388222524451e-57 | M2 macrophages | WTAP |
| 2.35384198852603e-61 | -0.366051404 | 0.642 | 0.714 | 8.49195574200535e-57 | M2 macrophages | ZBTB20 |
| 2.49928840345872e-61 | -0.752740938 | 0.359 | 0.439 | 9.01668277315803e-57 | M2 macrophages | EML4 |
| 4.63097329262178e-61 | 0.447207982445114 | 0.579 | 0.469 | 1.67071623477916e-56 | M2 macrophages | CD4 |
| 9.69811507126288e-61 | -0.470899928 | 0.577 | 0.633 | 3.49878897425951e-56 | M2 macrophages | RAB10 |
| 1.07402967917528e-60 | 0.326402033304134 | 0.707 | 0.645 | 3.87477687356065e-56 | M2 macrophages | GABARAP |
| 1.13019259771584e-60 | -0.914854121 | 0.162 | 0.259 | 4.07739583477943e-56 | M2 macrophages | ENSG00000286533 |
| 1.16313353439946e-60 | 0.412490133864438 | 0.454 | 0.335 | 4.19623685205294e-56 | M2 macrophages | DAD1 |
| 1.37872413161369e-60 | 0.410170804370555 | 0.444 | 0.32 | 4.97402304962272e-56 | M2 macrophages | SPCS1 |
| 2.26256978548807e-60 | 0.511778237380306 | 0.284 | 0.18 | 8.1626730151053e-56 | M2 macrophages | SBDS |
| 2.29729615390402e-60 | 0.852785186096685 | 0.273 | 0.178 | 8.28795533443952e-56 | M2 macrophages | LIMCH1 |
| 4.26862828705008e-60 | -0.724151938 | 0.196 | 0.292 | 1.53999302711906e-55 | M2 macrophages | MCU |
| 4.92111259099689e-60 | -0.782327447 | 0.199 | 0.297 | 1.77538978945395e-55 | M2 macrophages | LINC01619 |
| 5.37384812819821e-60 | 0.511992295077009 | 0.297 | 0.19 | 1.93872318921007e-55 | M2 macrophages | ECH1 |
| 5.88464202266443e-60 | -0.906697168 | 0.323 | 0.407 | 2.12300230251665e-55 | M2 macrophages | CXCL2 |
| 6.62599098774195e-60 | -0.394709844 | 0.65 | 0.754 | 2.39045876864766e-55 | M2 macrophages | COTL1 |
| 7.31412182793822e-60 | -0.730161041 | 0.219 | 0.315 | 2.63871573186527e-55 | M2 macrophages | SCFD2 |
| 1.10205069073304e-59 | -1.092660367 | 0.346 | 0.431 | 3.97586827695758e-55 | M2 macrophages | SESTD1 |
| 1.17304310601267e-59 | -0.690960218 | 0.338 | 0.432 | 4.23198761356192e-55 | M2 macrophages | NR1H3 |
| 4.75545604360953e-59 | 1.02725058051441 | 0.385 | 0.287 | 1.71562587685301e-54 | M2 macrophages | CD163L1 |
| 1.34412725993246e-58 | -0.70204506 | 0.278 | 0.366 | 4.84920791565834e-54 | M2 macrophages | TBC1D1 |
| 2.25715821741541e-58 | -0.750877695 | 0.216 | 0.308 | 8.14314970096958e-54 | M2 macrophages | EEIG2 |
| 2.2856947789761e-58 | 0.474244594184968 | 0.367 | 0.256 | 8.24610105411209e-54 | M2 macrophages | MYDGF |
| 3.88482523927077e-58 | -0.816364944 | 0.196 | 0.289 | 1.40152840157171e-53 | M2 macrophages | TRAF3 |
| 5.12943701369416e-58 | -0.339355955 | 0.462 | 0.584 | 1.85054699143044e-53 | M2 macrophages | XIST |
| 6.43331804818087e-58 | 0.412982631496113 | 0.612 | 0.527 | 2.32094815224221e-53 | M2 macrophages | RAB5C |
| 6.49254017252725e-58 | -0.632033723 | 0.345 | 0.431 | 2.34231371804265e-53 | M2 macrophages | CLEC7A |
| 7.97391602579788e-58 | 0.412404190580194 | 0.548 | 0.446 | 2.8767496846271e-53 | M2 macrophages | PEBP1 |
| 8.31763145787552e-58 | -0.460816731 | 0.912 | 0.922 | 3.00075190105775e-53 | M2 macrophages | NEAT1 |
| 1.15715901201273e-57 | -1.015507493 | 0.275 | 0.357 | 4.17468256763831e-53 | M2 macrophages | LINC01094 |
| 1.45999835485065e-57 | -0.367089793 | 0.856 | 0.892 | 5.26723606479468e-53 | M2 macrophages | MT-ND3 |
| 2.94473313630676e-57 | 0.332940214641519 | 0.826 | 0.799 | 1.06237137358539e-52 | M2 macrophages | TPT1 |
| 4.07285641471249e-57 | -0.519289186 | 0.528 | 0.589 | 1.46936440873583e-52 | M2 macrophages | IFNGR2 |
| 5.78999424961118e-57 | -0.639333877 | 0.21 | 0.304 | 2.08885622543223e-52 | M2 macrophages | TANGO6 |
| 1.12971665926046e-56 | 0.392674642139055 | 0.566 | 0.45 | 4.07567879161394e-52 | M2 macrophages | RGS10 |
| 1.15529866438532e-56 | -0.352497154 | 0.503 | 0.577 | 4.16797099150291e-52 | M2 macrophages | MYO9B |
| 1.78023037064385e-56 | -0.518675956 | 0.375 | 0.464 | 6.42253710817181e-52 | M2 macrophages | OGDH |
| 2.37801646846128e-56 | -0.413730801 | 0.588 | 0.642 | 8.57917001326777e-52 | M2 macrophages | CYTH1 |
| 4.47303162665132e-56 | 0.357395887291425 | 0.364 | 0.247 | 1.613735619947e-51 | M2 macrophages | ARF5 |
| 6.50744581353962e-56 | -0.674293432 | 0.321 | 0.406 | 2.34769122615069e-51 | M2 macrophages | ZDHHC20 |
| 8.05018367162331e-56 | -0.463945795 | 0.447 | 0.528 | 2.90426476321154e-51 | M2 macrophages | CFLAR |
| 8.49606505637779e-56 | -0.871766959 | 0.2 | 0.284 | 3.06512539038942e-51 | M2 macrophages | KIFC3 |
| 1.12984071138009e-55 | -1.676036494 | 0.222 | 0.297 | 4.07612633444595e-51 | M2 macrophages | TNF |
| 1.40409580074834e-55 | 0.493384925863091 | 0.386 | 0.276 | 5.0655564203598e-51 | M2 macrophages | TNFSF13 |
| 1.44513835918251e-55 | -0.653172475 | 0.398 | 0.475 | 5.21362565842272e-51 | M2 macrophages | MAP2K1 |
| 2.47747945637117e-55 | -0.484089352 | 0.443 | 0.522 | 8.93800263475026e-51 | M2 macrophages | TANK |
| 2.94534610901625e-55 | 0.455911194825943 | 0.434 | 0.321 | 1.06259251574979e-50 | M2 macrophages | RPN2 |
| 5.08144378189116e-55 | -0.329947906 | 0.874 | 0.91 | 1.83323247319287e-50 | M2 macrophages | MT-CYB |
| 6.79067765546912e-55 | -0.696893094 | 0.166 | 0.257 | 2.44987277776359e-50 | M2 macrophages | MAST2 |
| 1.2627784283255e-54 | 0.387992787446732 | 0.55 | 0.444 | 4.55572573586991e-50 | M2 macrophages | SKP1 |
| 1.89935007161798e-54 | -0.628824995 | 0.339 | 0.425 | 6.85228525337619e-50 | M2 macrophages | ZCCHC7 |
| 2.02862083508186e-54 | 0.405828448033729 | 0.418 | 0.302 | 7.31865538672483e-50 | M2 macrophages | ERP29 |
| 2.07603614909815e-54 | -0.633438553 | 0.447 | 0.515 | 7.48971561510138e-50 | M2 macrophages | ARHGAP22 |
| 1.18191549571009e-53 | -0.554878631 | 0.576 | 0.626 | 4.26399653387328e-49 | M2 macrophages | HCK |
| 1.37182892322366e-53 | -0.568694058 | 0.492 | 0.566 | 4.94914720631402e-49 | M2 macrophages | ETV6 |
| 1.39487079353548e-53 | -0.619616857 | 0.266 | 0.358 | 5.03227536183795e-49 | M2 macrophages | RUFY3 |
| 1.56412120102174e-53 | -0.541714407 | 0.4 | 0.484 | 5.64288005692615e-49 | M2 macrophages | ADAM17 |
| 2.17072112051981e-53 | -0.63850321 | 0.299 | 0.387 | 7.83131058649932e-49 | M2 macrophages | KDM2B |
| 2.51924678803413e-53 | 0.360614653574382 | 0.611 | 0.53 | 9.08868663719075e-49 | M2 macrophages | MYL12A |
| 2.62194867773637e-53 | -0.465635065 | 0.542 | 0.62 | 9.4592042446695e-49 | M2 macrophages | PLEC |
| 2.97227340102149e-53 | 0.523931434918306 | 0.263 | 0.168 | 1.07230707488652e-48 | M2 macrophages | SLC39A1 |
| 3.0556907797694e-53 | 0.374830668667013 | 0.58 | 0.489 | 1.10240156261741e-48 | M2 macrophages | RNH1 |
| 3.31637545724928e-53 | 0.337428972438916 | 0.669 | 0.61 | 1.19644877371182e-48 | M2 macrophages | ATP6V0B |
| 3.80509880285064e-53 | -0.661765143 | 0.331 | 0.42 | 1.37276549510443e-48 | M2 macrophages | KIF13B |
| 4.65226316366286e-53 | -0.699079469 | 0.183 | 0.273 | 1.67839698155465e-48 | M2 macrophages | L3MBTL4 |
| 4.94498073698627e-53 | -0.668883726 | 0.641 | 0.666 | 1.78400070048253e-48 | M2 macrophages | NFKBIA |
| 5.7681776691568e-53 | -0.780903469 | 0.196 | 0.284 | 2.0809854577017e-48 | M2 macrophages | DDHD1 |
| 6.4266345418226e-53 | -0.58336305 | 0.25 | 0.34 | 2.31853694365334e-48 | M2 macrophages | PTK2B |
| 7.05769306129442e-53 | -0.65492338 | 0.218 | 0.308 | 2.54620392572319e-48 | M2 macrophages | SYNE3 |
| 1.00733688054978e-52 | -0.371545998 | 0.797 | 0.813 | 3.63416926395943e-48 | M2 macrophages | LRMDA |
| 1.67092686922917e-52 | 0.481559189661584 | 0.263 | 0.168 | 6.02820286611807e-48 | M2 macrophages | PSENEN |
| 1.79628233195325e-52 | 0.332692610891863 | 0.6 | 0.49 | 6.48044776898773e-48 | M2 macrophages | ALDH1A1 |
| 2.24095222710618e-52 | 0.371723619016328 | 0.277 | 0.178 | 8.08468334973098e-48 | M2 macrophages | DBNDD2 |
| 2.59830648574197e-52 | -0.415076813 | 0.548 | 0.617 | 9.37391030861132e-48 | M2 macrophages | SBF2 |
| 2.6897540099991e-52 | 0.781052977451677 | 0.491 | 0.417 | 9.70382554187377e-48 | M2 macrophages | AUTS2 |
| 3.18157109566538e-52 | 0.406156974425673 | 0.337 | 0.231 | 1.1478154041832e-47 | M2 macrophages | SELPLG |
| 3.9013765144139e-52 | -1.188552522 | 0.255 | 0.333 | 1.4074996051051e-47 | M2 macrophages | EIF4E |
| 5.94598822774829e-52 | -0.61249261 | 0.231 | 0.323 | 2.14513417292475e-47 | M2 macrophages | CDYL |
| 7.04871943829202e-52 | -0.643776686 | 0.257 | 0.346 | 2.54296651175261e-47 | M2 macrophages | PITPNB |
| 7.5953520361482e-52 | 0.306658999445352 | 0.848 | 0.821 | 2.74017515408119e-47 | M2 macrophages | CTSZ |
| 8.5236019895659e-52 | -0.711546826 | 0.221 | 0.309 | 3.07505988977569e-47 | M2 macrophages | STK40 |
| 1.26160610067363e-51 | 0.381131668594104 | 0.475 | 0.368 | 4.55149632940027e-47 | M2 macrophages | NDUFC2 |
| 1.2823060237676e-51 | 0.420675665295811 | 0.41 | 0.3 | 4.62617544194635e-47 | M2 macrophages | CEBPD |
| 1.8505111731766e-51 | -0.382512759 | 0.631 | 0.684 | 6.67608915946921e-47 | M2 macrophages | EIF4G3 |
| 2.54605944863248e-51 | 0.828648319440532 | 0.386 | 0.292 | 9.18541867283138e-47 | M2 macrophages | DUSP6 |
| 3.11198208187353e-51 | -0.786823897 | 0.205 | 0.288 | 1.12270977567751e-46 | M2 macrophages | FMNL3 |
| 4.25168927111272e-51 | -0.420753467 | 0.587 | 0.654 | 1.53388193833934e-46 | M2 macrophages | MCTP1 |
| 5.28684254450211e-51 | -0.562855628 | 0.246 | 0.339 | 1.90733418478003e-46 | M2 macrophages | CAMSAP2 |
| 7.08885956600636e-51 | -0.856637032 | 0.215 | 0.295 | 2.55744786562811e-46 | M2 macrophages | ACTN1 |
| 9.96240873843352e-51 | 0.445188724616865 | 0.27 | 0.174 | 3.59413820056466e-46 | M2 macrophages | EMC7 |
| 1.15424335097667e-50 | 0.69825343698605 | 0.271 | 0.183 | 4.16416373731854e-46 | M2 macrophages | MVB12B |
| 1.38207546894727e-50 | -0.424968902 | 0.41 | 0.498 | 4.98611366932106e-46 | M2 macrophages | ERC1 |
| 2.28063564266074e-50 | 0.416378512426348 | 0.8 | 0.748 | 8.22784920802716e-46 | M2 macrophages | ZFP36L1 |
| 3.06920288352237e-50 | 0.37923071571374 | 0.422 | 0.31 | 1.10727632428837e-45 | M2 macrophages | BST2 |
| 3.21703892636821e-50 | 0.3838087305527 | 0.728 | 0.686 | 1.16061113346586e-45 | M2 macrophages | GPX1 |
| 3.86751311889397e-50 | -0.834363755 | 0.206 | 0.288 | 1.39528270790338e-45 | M2 macrophages | NPC1 |
| 4.19600731092299e-50 | -0.380911028 | 0.765 | 0.789 | 1.51379355756169e-45 | M2 macrophages | RBM47 |
| 4.40563791723609e-50 | 0.401159156337474 | 0.371 | 0.263 | 1.58942199140126e-45 | M2 macrophages | ADIPOR1 |
| 6.91802463607058e-50 | 0.419123882874935 | 0.355 | 0.247 | 2.49581574795518e-45 | M2 macrophages | CUTA |
| 7.6465580288543e-50 | 0.3966632415987 | 0.516 | 0.425 | 2.75864874006976e-45 | M2 macrophages | SYNGR2 |
| 8.14854346292757e-50 | -0.561687526 | 0.302 | 0.388 | 2.93975002512038e-45 | M2 macrophages | SLC39A11 |
| 1.56576851921613e-49 | 0.36369936554759 | 0.439 | 0.328 | 5.64882308677604e-45 | M2 macrophages | NDUFB11 |
| 2.02938326192908e-49 | 0.395741705072858 | 0.301 | 0.202 | 7.32140599406154e-45 | M2 macrophages | ADI1 |
| 3.17743370647375e-49 | 0.418953115895531 | 0.452 | 0.351 | 1.14632275828454e-44 | M2 macrophages | CTSA |
| 3.59450492746884e-49 | 0.376595647356444 | 0.447 | 0.332 | 1.29678954268293e-44 | M2 macrophages | CNPY3 |
| 3.9053833547475e-49 | 0.520540468365992 | 0.275 | 0.18 | 1.40894515289225e-44 | M2 macrophages | GINM1 |
| 4.70257391127291e-49 | 0.527443869512691 | 0.439 | 0.338 | 1.69654758996993e-44 | M2 macrophages | MYADM |
| 5.06492708586161e-49 | -0.771084635 | 0.313 | 0.392 | 1.82727374476629e-44 | M2 macrophages | SMURF2 |
| 5.75743924889327e-49 | -0.632953275 | 0.337 | 0.418 | 2.07711135782323e-44 | M2 macrophages | ATP9B |
| 7.49599798155596e-49 | 0.421603942848772 | 0.403 | 0.296 | 2.70433119180594e-44 | M2 macrophages | SRP9 |
| 7.54973439425154e-49 | 0.374772507326754 | 0.264 | 0.169 | 2.72371767741413e-44 | M2 macrophages | IMP3 |
| 8.49133625066374e-49 | -0.494485222 | 0.36 | 0.446 | 3.06341937915196e-44 | M2 macrophages | PPM1L |
| 1.12710364302666e-48 | -0.451536824 | 0.474 | 0.543 | 4.06625181294728e-44 | M2 macrophages | USP15 |
| 1.17282906283664e-48 | 0.411508536783156 | 0.396 | 0.289 | 4.23121540999574e-44 | M2 macrophages | ATRAID |
| 1.3172765361838e-48 | -0.503409212 | 0.359 | 0.446 | 4.75233855959029e-44 | M2 macrophages | STK10 |
| 1.34132872857166e-48 | -0.450249853 | 0.333 | 0.424 | 4.83911165406798e-44 | M2 macrophages | MRTFA |
| 1.51551270227974e-48 | 0.579304545160978 | 0.423 | 0.318 | 5.4675151760146e-44 | M2 macrophages | RASSF4 |
| 1.81739509014786e-48 | 0.460009429995329 | 0.563 | 0.463 | 6.55661626672644e-44 | M2 macrophages | HMOX1 |
| 1.95652807646521e-48 | 0.544913200588274 | 0.886 | 0.92 | 7.05856634146356e-44 | M2 macrophages | CD74 |
| 2.32856050913389e-48 | -0.322874053 | 0.473 | 0.586 | 8.40074774880233e-44 | M2 macrophages | TIMD4 |
| 2.52010003553518e-48 | 0.380218642901367 | 0.625 | 0.512 | 9.09176489820029e-44 | M2 macrophages | SLCO2B1 |
| 2.78046972099846e-48 | -0.511234326 | 0.247 | 0.341 | 1.00311006124462e-43 | M2 macrophages | PDE4B |
| 3.87171931849127e-48 | 0.540552968164565 | 0.309 | 0.212 | 1.39680017853209e-43 | M2 macrophages | PLA2G15 |
| 3.9449467343412e-48 | 0.493923161224043 | 0.629 | 0.515 | 1.42321843334827e-43 | M2 macrophages | SIGLEC1 |
| 4.02231543000289e-48 | -0.642184369 | 0.405 | 0.468 | 1.45113073768214e-43 | M2 macrophages | ST3GAL1 |
| 6.5359942766161e-48 | -0.696294841 | 0.225 | 0.307 | 2.35799065517479e-43 | M2 macrophages | MAP3K20 |
| 8.7650468610097e-48 | 0.563276742834505 | 0.27 | 0.18 | 3.16216595604647e-43 | M2 macrophages | TRMT1 |
| 9.82567453720384e-48 | 0.746916378206851 | 0.469 | 0.374 | 3.54480860278703e-43 | M2 macrophages | BTG2 |
| 1.45981195522267e-47 | 0.442923305297133 | 0.288 | 0.194 | 5.26656359085682e-43 | M2 macrophages | C14orf119 |
| 1.62271851658747e-47 | 0.486330660393496 | 0.271 | 0.178 | 5.85428159229263e-43 | M2 macrophages | TMED4 |
| 7.96357409352613e-47 | 0.488222748927033 | 0.268 | 0.177 | 2.87301862572142e-42 | M2 macrophages | YIPF3 |
| 1.76096777918609e-46 | -0.6024642 | 0.175 | 0.256 | 6.35304345696966e-42 | M2 macrophages | VWA8 |
| 2.66595644711295e-46 | -0.811122616 | 0.257 | 0.331 | 9.6179710742494e-42 | M2 macrophages | SOCS6 |
| 3.63743138853166e-46 | -0.379471364 | 0.475 | 0.544 | 1.31227612204057e-41 | M2 macrophages | MANBA |
| 3.66380250386965e-46 | 0.420852270374311 | 0.267 | 0.175 | 1.32179002932105e-41 | M2 macrophages | DDRGK1 |
| 4.35974124852539e-46 | -0.435367937 | 0.405 | 0.489 | 1.57286385023051e-41 | M2 macrophages | FNIP1 |
| 4.64659677254974e-46 | 0.421311983727322 | 0.318 | 0.221 | 1.67635271763277e-41 | M2 macrophages | SDHC |
| 4.74809121421857e-46 | -0.454052907 | 0.693 | 0.735 | 1.71296886735363e-41 | M2 macrophages | FYB1 |
| 5.00275834825572e-46 | 0.351413043982345 | 0.304 | 0.206 | 1.80484512930021e-41 | M2 macrophages | MRPS34 |
| 5.19986429738664e-46 | 0.31347031485252 | 0.692 | 0.66 | 1.87595504256818e-41 | M2 macrophages | RNASEK |
| 6.20367141840754e-46 | 0.405680432534735 | 0.266 | 0.175 | 2.23809853761889e-41 | M2 macrophages | TCEAL3 |
| 7.53810145123403e-46 | 0.420168480001715 | 0.297 | 0.2 | 2.7195208605617e-41 | M2 macrophages | NDUFB6 |
| 9.04895791262512e-46 | -0.567007141 | 0.397 | 0.466 | 3.26459254613776e-41 | M2 macrophages | HLA-DRB6 |
| 1.09139713385628e-45 | -0.665583643 | 0.234 | 0.316 | 3.93743343981332e-41 | M2 macrophages | HYCC1 |
| 2.30320625517314e-45 | -0.499022867 | 0.399 | 0.473 | 8.30927720678815e-41 | M2 macrophages | PTPN12 |
| 2.93695295710799e-45 | -0.565233282 | 0.25 | 0.333 | 1.05956451833585e-40 | M2 macrophages | SIPA1L2 |
| 3.00188027041215e-45 | 0.375653522532094 | 0.276 | 0.184 | 1.08298834515659e-40 | M2 macrophages | NDUFB5 |
| 3.21525573174967e-45 | -0.439672156 | 0.42 | 0.5 | 1.15996781034333e-40 | M2 macrophages | LTA4H |
| 3.72225695627529e-45 | -0.466149419 | 0.398 | 0.477 | 1.34287864211544e-40 | M2 macrophages | VTI1A |
| 3.81828592773436e-45 | 0.542195288975081 | 0.253 | 0.168 | 1.37752301414873e-40 | M2 macrophages | CD33 |
| 4.00653765571014e-45 | 0.384003285891751 | 0.691 | 0.603 | 1.44543859005055e-40 | M2 macrophages | HTRA1 |
| 5.97236479649683e-45 | -0.351545234 | 0.403 | 0.484 | 2.15465004763216e-40 | M2 macrophages | KDM2A |
| 7.18762545709721e-45 | -0.545481792 | 0.391 | 0.459 | 2.59307963615696e-40 | M2 macrophages | IQSEC1 |
| 9.15310491375101e-45 | 0.381848905905303 | 0.485 | 0.389 | 3.30216565973395e-40 | M2 macrophages | ISCU |
| 1.16599650451361e-44 | 0.750182957352295 | 0.508 | 0.426 | 4.20656558933373e-40 | M2 macrophages | EGR1 |
| 1.1922093011659e-44 | 0.390958289308938 | 0.352 | 0.25 | 4.30113349581623e-40 | M2 macrophages | CORO1B |
| 1.8264823663747e-44 | 0.392382802087769 | 0.501 | 0.411 | 6.58940043316999e-40 | M2 macrophages | ATP5MC3 |
| 2.43213873541786e-44 | 0.325379355685772 | 0.463 | 0.355 | 8.774426915767e-40 | M2 macrophages | PSME1 |
| 2.74059131528674e-44 | -0.400948948 | 0.333 | 0.421 | 9.88723128815998e-40 | M2 macrophages | KDM4C |
| 2.91058419593708e-44 | 0.372987484667097 | 0.526 | 0.423 | 1.05005146036822e-39 | M2 macrophages | GMFG |
| 3.78761917329328e-44 | 0.441213850518258 | 0.301 | 0.206 | 1.36645936914902e-39 | M2 macrophages | PIGT |
| 4.0150598459543e-44 | 0.467234535347077 | 0.356 | 0.258 | 1.44851314062493e-39 | M2 macrophages | EPN1 |
| 4.1615623108147e-44 | 0.424709189990599 | 0.307 | 0.21 | 1.50136683487262e-39 | M2 macrophages | SMIM26 |
| 4.98042920056396e-44 | -0.740605645 | 0.364 | 0.426 | 1.79678944268746e-39 | M2 macrophages | NCEH1 |
| 5.1787307865745e-44 | -0.588001136 | 0.352 | 0.423 | 1.86833070587248e-39 | M2 macrophages | JAZF1 |
| 5.29705182848499e-44 | 0.377060954728622 | 0.404 | 0.296 | 1.91101738816253e-39 | M2 macrophages | APH1A |
| 9.51118277421573e-44 | 0.422688643574181 | 0.364 | 0.264 | 3.43134940945381e-39 | M2 macrophages | TRIM14 |
| 1.34188073619189e-43 | -0.463239311 | 0.331 | 0.413 | 4.84110313195949e-39 | M2 macrophages | DYM |
| 1.52815693684706e-43 | 0.393546464179654 | 0.27 | 0.18 | 5.51313178106314e-39 | M2 macrophages | TMEM205 |
| 1.72256609707497e-43 | 0.474771621243099 | 0.257 | 0.172 | 6.21450170841739e-39 | M2 macrophages | DSC2 |
| 2.27547603323611e-43 | -0.624701324 | 0.22 | 0.3 | 8.20923488510592e-39 | M2 macrophages | MAPK6 |
| 2.56795154681906e-43 | 0.371435238912367 | 0.259 | 0.171 | 9.26439879545913e-39 | M2 macrophages | PCBD1 |
| 2.86555086725778e-43 | 0.376131972894864 | 0.385 | 0.281 | 1.03380478638059e-38 | M2 macrophages | RAB32 |
| 3.014874912603e-43 | 0.398921005118468 | 0.436 | 0.335 | 1.08767642221978e-38 | M2 macrophages | RNF150 |
| 3.61965281255053e-43 | -0.556013306 | 0.261 | 0.342 | 1.30586214518386e-38 | M2 macrophages | TMEM117 |
| 4.27196621692989e-43 | 0.320537474068688 | 0.485 | 0.382 | 1.5411972520818e-38 | M2 macrophages | WDR83OS |
| 4.89693992039616e-43 | 0.572373956726844 | 0.536 | 0.446 | 1.76666901508132e-38 | M2 macrophages | PDGFC |
| 5.84820856448392e-43 | -0.591366663 | 0.325 | 0.406 | 2.10985820380886e-38 | M2 macrophages | SLCO3A1 |
| 5.99217410908235e-43 | 0.366754522709202 | 0.374 | 0.271 | 2.16179665333364e-38 | M2 macrophages | NDUFC1 |
| 6.14788474442467e-43 | -0.454402954 | 0.394 | 0.473 | 2.21797237924609e-38 | M2 macrophages | B4GALT1 |
| 6.19560446755714e-43 | 0.404097038086012 | 0.32 | 0.225 | 2.23518822376059e-38 | M2 macrophages | RNASE6 |
| 7.9528454329084e-43 | 0.540249421836004 | 0.473 | 0.376 | 2.86914804683036e-38 | M2 macrophages | SPATS2L |
| 1.08033831621543e-42 | -0.386227799 | 0.557 | 0.605 | 3.89753654341041e-38 | M2 macrophages | ITSN2 |
| 1.15327194727878e-42 | 0.385711658802118 | 0.275 | 0.184 | 4.16065920419765e-38 | M2 macrophages | SNRPC |
| 1.21737795709299e-42 | 0.362200492154843 | 0.316 | 0.22 | 4.39193445580437e-38 | M2 macrophages | TXNDC12 |
| 1.27421265306389e-42 | -0.31838411 | 0.686 | 0.726 | 4.59697698845861e-38 | M2 macrophages | LPP |
| 1.36172397313002e-42 | -0.478110888 | 0.421 | 0.482 | 4.91269157786117e-38 | M2 macrophages | CORO1C |
| 2.68163007573311e-42 | -0.976186501 | 0.196 | 0.264 | 9.67451682422233e-38 | M2 macrophages | LINC02542 |
| 3.41265582527344e-42 | 0.354845153294104 | 0.453 | 0.348 | 1.2311838420839e-37 | M2 macrophages | VPS28 |
| 3.75187960371204e-42 | 0.330831239292838 | 0.576 | 0.487 | 1.35356560463119e-37 | M2 macrophages | ACP5 |
| 4.75878663803716e-42 | 0.444107898074976 | 0.26 | 0.173 | 1.71682745540467e-37 | M2 macrophages | NME3 |
| 5.04222819701092e-42 | -0.553740085 | 0.259 | 0.335 | 1.81908466663563e-37 | M2 macrophages | CTTNBP2NL |
| 5.76849848091903e-42 | 0.320141421599816 | 0.504 | 0.41 | 2.08110119696116e-37 | M2 macrophages | SLC25A5 |
| 6.18238214574656e-42 | 0.321082376479123 | 0.432 | 0.33 | 2.23041800672098e-37 | M2 macrophages | KDELR1 |
| 6.22530181042957e-42 | 0.377043680815473 | 0.301 | 0.208 | 2.24590213414868e-37 | M2 macrophages | ECHS1 |
| 7.50009992607589e-42 | -0.477113982 | 0.446 | 0.504 | 2.7058110503304e-37 | M2 macrophages | RPS6KA2 |
| 7.78427262338893e-42 | -0.327973128 | 0.65 | 0.694 | 2.80833203434002e-37 | M2 macrophages | ARHGAP24 |
| 8.00852542480431e-42 | 0.430426189251218 | 0.438 | 0.346 | 2.88923571750665e-37 | M2 macrophages | PDIA6 |
| 1.14805842493306e-41 | 0.403729151733666 | 0.352 | 0.256 | 4.14185037963101e-37 | M2 macrophages | MMADHC |
| 1.15437361969569e-41 | 0.426532746054618 | 0.285 | 0.195 | 4.16463370777612e-37 | M2 macrophages | GET3 |
| 1.32734572443325e-41 | -0.510289756 | 0.392 | 0.457 | 4.78866517003782e-37 | M2 macrophages | TFEC |
| 1.47172662159411e-41 | -0.629354059 | 0.263 | 0.338 | 5.30954813272506e-37 | M2 macrophages | SGMS2 |
| 1.64619608098097e-41 | -0.612439226 | 0.27 | 0.351 | 5.93898160135503e-37 | M2 macrophages | FRY |
| 1.72927578562026e-41 | -0.555024009 | 0.235 | 0.316 | 6.2387082517822e-37 | M2 macrophages | ZNF407 |
| 1.74563018540561e-41 | 0.436957425362348 | 0.297 | 0.207 | 6.29771001988783e-37 | M2 macrophages | EIF4A3 |
| 1.87221102907247e-41 | -0.54461258 | 0.453 | 0.512 | 6.75437572958476e-37 | M2 macrophages | NRIP1 |
| 1.98026668340091e-41 | 0.34514462943224 | 0.588 | 0.518 | 7.14420811370546e-37 | M2 macrophages | NDUFA4 |
| 2.07902819562122e-41 | 0.339163625004019 | 0.341 | 0.244 | 7.50051002134268e-37 | M2 macrophages | PPCS |
| 2.75275286396016e-41 | -0.396455308 | 0.371 | 0.451 | 9.93110650730908e-37 | M2 macrophages | FAF1 |
| 3.24610574302874e-41 | 0.422017482443435 | 0.42 | 0.322 | 1.17109756891248e-36 | M2 macrophages | TM9SF2 |
| 3.2798170231388e-41 | -0.513891946 | 0.421 | 0.477 | 1.18325958743779e-36 | M2 macrophages | ABI1 |
| 3.97888367597306e-41 | -0.606638102 | 0.237 | 0.311 | 1.4354618637808e-36 | M2 macrophages | SLC6A6 |
| 6.10340739758127e-41 | 0.315050363972164 | 0.714 | 0.637 | 2.20192628682539e-36 | M2 macrophages | HMGB1 |
| 6.56586962721835e-41 | -0.478643184 | 0.267 | 0.348 | 2.36876878541157e-36 | M2 macrophages | WDR7 |
| 6.70656632061505e-41 | -0.462007435 | 0.431 | 0.492 | 2.41952793148829e-36 | M2 macrophages | MAP4 |
| 7.12225249779708e-41 | -0.648330351 | 0.185 | 0.257 | 2.56949503363025e-36 | M2 macrophages | GABBR1 |
| 7.84430839718213e-41 | 0.306881975652223 | 0.274 | 0.183 | 2.8299911404514e-36 | M2 macrophages | SURF1 |
| 8.83048989735074e-41 | 0.357499569869624 | 0.336 | 0.239 | 3.18577584026723e-36 | M2 macrophages | NDUFB3 |
| 1.88047368715134e-40 | -0.843210588 | 0.231 | 0.302 | 6.78418492113589e-36 | M2 macrophages | TALAM1 |
| 2.56947499704177e-40 | 0.34924040230679 | 0.446 | 0.354 | 9.26989494682761e-36 | M2 macrophages | GRINA |
| 4.04594138364014e-40 | 0.313350063925573 | 0.574 | 0.488 | 1.45965427297585e-35 | M2 macrophages | C2 |
| 4.86088190923428e-40 | -0.428839352 | 0.369 | 0.445 | 1.75366036639445e-35 | M2 macrophages | LARP4B |
| 8.49880105937031e-40 | 0.420605734921274 | 0.326 | 0.236 | 3.06611245818903e-35 | M2 macrophages | SDHD |
| 1.13021698392507e-39 | -0.662026295 | 0.182 | 0.256 | 4.07748381290649e-35 | M2 macrophages | FAM135A |
| 1.27264806198176e-39 | 0.304025023699858 | 0.625 | 0.534 | 4.5913324132116e-35 | M2 macrophages | TUBA1B |
| 1.45476652718707e-39 | -0.713348998 | 0.245 | 0.312 | 5.24836120013279e-35 | M2 macrophages | ARHGEF3 |
| 1.6157278849952e-39 | -0.656208165 | 0.203 | 0.277 | 5.82906149069718e-35 | M2 macrophages | PAG1 |
| 1.9385761467461e-39 | -0.351119066 | 0.355 | 0.436 | 6.99380116461589e-35 | M2 macrophages | PPP6R3 |
| 2.00411816085394e-39 | 0.315989281641336 | 0.622 | 0.548 | 7.23025708891278e-35 | M2 macrophages | MFSD1 |
| 2.07697863485001e-39 | 0.429714048244614 | 0.272 | 0.186 | 7.49311582094837e-35 | M2 macrophages | ACAA2 |
| 3.28475715327468e-39 | 0.325778876220624 | 0.436 | 0.337 | 1.1850418381869e-34 | M2 macrophages | GSTK1 |
| 4.01642975364982e-39 | 0.347471809949612 | 0.497 | 0.398 | 1.44900736222425e-34 | M2 macrophages | MAP1LC3B |
| 5.36204891680111e-39 | 0.524196030788204 | 0.306 | 0.222 | 1.93446638771434e-34 | M2 macrophages | CYP1B1 |
| 5.98661015954341e-39 | -0.34757856 | 0.524 | 0.579 | 2.15978934725848e-34 | M2 macrophages | WNK1 |
| 6.38034642599193e-39 | -0.596237288 | 0.31 | 0.376 | 2.30183758010511e-34 | M2 macrophages | RALGDS |
| 8.05339054248411e-39 | 0.419679512908393 | 0.416 | 0.326 | 2.90542170601199e-34 | M2 macrophages | IGSF6 |
| 8.91212809676933e-39 | 0.374018473342859 | 0.25 | 0.168 | 3.21522845347147e-34 | M2 macrophages | EIF4EBP3 |
| 9.27259146496894e-39 | 0.303722374929791 | 0.516 | 0.425 | 3.34527282281685e-34 | M2 macrophages | UQCR10 |
| 1.53865856555901e-38 | 0.318021258905389 | 0.279 | 0.192 | 5.55101850696723e-34 | M2 macrophages | PSMB8 |
| 1.77829597843104e-38 | 0.310418009523381 | 0.391 | 0.289 | 6.41555840138568e-34 | M2 macrophages | DUSP23 |
| 2.16614691605577e-38 | -0.479632259 | 0.402 | 0.464 | 7.81480822905441e-34 | M2 macrophages | PDCD6IP |
| 2.35429186891298e-38 | 0.448939754677171 | 0.9 | 0.843 | 8.49357877547738e-34 | M2 macrophages | RBPJ |
| 2.47002200374892e-38 | -0.350329076 | 0.607 | 0.65 | 8.91109838292499e-34 | M2 macrophages | DOCK2 |
| 3.77907409932823e-38 | -0.336628047 | 0.629 | 0.666 | 1.36337656281465e-33 | M2 macrophages | NFE2L2 |
| 3.85334894130475e-38 | 0.300527088034007 | 0.57 | 0.491 | 1.39017269755451e-33 | M2 macrophages | SARAF |
| 4.45221475771282e-38 | 0.382410354237234 | 0.28 | 0.195 | 1.60622551814005e-33 | M2 macrophages | MESD |
| 4.65847400287318e-38 | 0.331545679847855 | 0.427 | 0.326 | 1.68063766601656e-33 | M2 macrophages | PGLS |
| 5.67468318614961e-38 | 0.39958021449238 | 0.365 | 0.277 | 2.04725545306719e-33 | M2 macrophages | FABP3 |
| 5.77640945273302e-38 | -0.517066956 | 0.457 | 0.52 | 2.08395523826249e-33 | M2 macrophages | DIAPH2 |
| 5.8362537138711e-38 | 0.321630671064393 | 0.503 | 0.408 | 2.10554525235327e-33 | M2 macrophages | SOD1 |
| 6.30930726032031e-38 | 0.335105275073266 | 0.891 | 0.908 | 2.27620878030576e-33 | M2 macrophages | CTSB |
| 6.97620597000624e-38 | 0.422584647981397 | 0.261 | 0.178 | 2.51680582779915e-33 | M2 macrophages | NDUFS3 |
| 7.7740837900855e-38 | -0.715646492 | 0.284 | 0.347 | 2.80465620894915e-33 | M2 macrophages | IGSF21 |
| 9.2314525391416e-38 | -0.403077564 | 0.54 | 0.585 | 3.33043113254611e-33 | M2 macrophages | CHD9 |
| 9.30666027207114e-38 | 0.341234972336652 | 0.372 | 0.275 | 3.35756382635511e-33 | M2 macrophages | SSR2 |
| 1.34799221750686e-37 | 0.33804335757551 | 0.705 | 0.648 | 4.86315152309952e-33 | M2 macrophages | SQSTM1 |
| 1.56457002532741e-37 | 0.302259417108058 | 0.465 | 0.372 | 5.64449928037371e-33 | M2 macrophages | DHRS7 |
| 1.74453561795744e-37 | -0.427511046 | 0.261 | 0.34 | 6.29376114890507e-33 | M2 macrophages | ATP11A |
| 2.84657140116969e-37 | -0.710033769 | 0.297 | 0.357 | 1.02695756439999e-32 | M2 macrophages | TMEM51 |
| 3.77957145195488e-37 | -0.60908898 | 0.263 | 0.33 | 1.36355599272176e-32 | M2 macrophages | ATP6V1H |
| 5.1432722920014e-37 | -0.75726399 | 0.197 | 0.265 | 1.85553834478535e-32 | M2 macrophages | ANK2 |
| 5.18743274191785e-37 | 0.311769328015312 | 0.485 | 0.393 | 1.8714701103017e-32 | M2 macrophages | ATP5PO |
| 7.23011377999779e-37 | 0.351886790834808 | 0.263 | 0.181 | 2.6084081484098e-32 | M2 macrophages | RPL22L1 |
| 8.57505703217074e-37 | -0.591406272 | 0.308 | 0.378 | 3.09362332549624e-32 | M2 macrophages | RASA1 |
| 1.4594217252592e-36 | 0.308481281324949 | 0.483 | 0.385 | 5.26515575821761e-32 | M2 macrophages | ARL6IP1 |
| 1.83210655426848e-36 | 0.315984696318328 | 0.499 | 0.413 | 6.60969081583439e-32 | M2 macrophages | NUCB1 |
| 2.02023796623852e-36 | 0.311187292952602 | 0.56 | 0.481 | 7.2884125107987e-32 | M2 macrophages | DAZAP2 |
| 2.04225085421576e-36 | 0.333570100853374 | 0.372 | 0.279 | 7.36782840675419e-32 | M2 macrophages | LRPAP1 |
| 2.21729579102672e-36 | 0.330230951220139 | 0.467 | 0.366 | 7.99933802528708e-32 | M2 macrophages | RCSD1 |
| 3.36044967192717e-36 | 0.346609086905191 | 0.33 | 0.241 | 1.21234942814116e-31 | M2 macrophages | MDH2 |
| 4.49055041897419e-36 | 0.328846156805005 | 0.382 | 0.287 | 1.62005587465332e-31 | M2 macrophages | SDF4 |
| 4.61400285700089e-36 | -0.545412979 | 0.204 | 0.278 | 1.66459381072021e-31 | M2 macrophages | PDE8A |
| 5.14198381818703e-36 | -0.390093274 | 0.444 | 0.507 | 1.85507350208734e-31 | M2 macrophages | NCOR2 |
| 5.37090688422417e-36 | -0.556334727 | 0.191 | 0.262 | 1.93766207662155e-31 | M2 macrophages | E2F3 |
| 6.21713769412688e-36 | 0.46790364095989 | 0.279 | 0.2 | 2.24295676591015e-31 | M2 macrophages | S100A13 |
| 6.22789916165767e-36 | 0.314378147669945 | 0.399 | 0.302 | 2.24683918055124e-31 | M2 macrophages | TWF2 |
| 6.27984350937181e-36 | -0.343149882 | 0.541 | 0.606 | 2.26557914287607e-31 | M2 macrophages | RUNX1 |
| 7.32557481546361e-36 | 0.548482338963727 | 0.289 | 0.21 | 2.64284762617481e-31 | M2 macrophages | NASP |
| 7.43812278100373e-36 | -0.440434684 | 0.358 | 0.422 | 2.68345155570271e-31 | M2 macrophages | CLIP1 |
| 8.23090494027333e-36 | -0.55212015 | 0.259 | 0.328 | 2.96946357530241e-31 | M2 macrophages | RAP1GDS1 |
| 9.86162128551687e-36 | -0.429335729 | 0.452 | 0.503 | 3.55777711117592e-31 | M2 macrophages | NAP1L1 |
| 9.9704568325158e-36 | 0.38502547547168 | 0.274 | 0.191 | 3.59704171146673e-31 | M2 macrophages | MRPL43 |
| 1.00666328675673e-35 | 0.600268854881501 | 0.685 | 0.641 | 3.63173913963224e-31 | M2 macrophages | JUN |
| 1.3896441852381e-35 | -0.43938248 | 0.327 | 0.392 | 5.01341932708351e-31 | M2 macrophages | EHBP1L1 |
| 1.43761328105797e-35 | -0.331616472 | 0.452 | 0.523 | 5.18647743407285e-31 | M2 macrophages | GAB2 |
| 1.73916596702541e-35 | -0.456216643 | 0.434 | 0.499 | 6.27438905923759e-31 | M2 macrophages | SFMBT2 |
| 1.81057899607043e-35 | 0.407204576633471 | 0.257 | 0.177 | 6.5320258441233e-31 | M2 macrophages | LRRC25 |
| 2.05866721907401e-35 | -0.352749982 | 0.259 | 0.337 | 7.42705372625329e-31 | M2 macrophages | RALGAPA1 |
| 2.45398079625895e-35 | -0.406492517 | 0.301 | 0.377 | 8.85322651866341e-31 | M2 macrophages | RABGEF1 |
| 2.84740029050495e-35 | -0.368019356 | 0.46 | 0.517 | 1.02725660280547e-30 | M2 macrophages | MYH9 |
| 3.23385896907861e-35 | -0.671922662 | 0.198 | 0.265 | 1.16667930027449e-30 | M2 macrophages | ASCC3 |
| 3.39725870229426e-35 | 0.353952263694782 | 0.294 | 0.21 | 1.2256290220267e-30 | M2 macrophages | CHMP5 |
| 5.92862568106476e-35 | -0.471293 | 0.255 | 0.327 | 2.13887028695773e-30 | M2 macrophages | AGAP3 |
| 6.48369889144801e-35 | -0.68684708 | 0.276 | 0.335 | 2.3391240490677e-30 | M2 macrophages | BID |
| 6.51221223954985e-35 | -0.37491807 | 0.333 | 0.409 | 2.3494108096624e-30 | M2 macrophages | NF1 |
| 8.83906746264898e-35 | 0.488044185763082 | 0.29 | 0.213 | 3.18887036849987e-30 | M2 macrophages | RAB11FIP1 |
| 8.8393926360257e-35 | 0.30899271267539 | 0.498 | 0.406 | 3.18898768129899e-30 | M2 macrophages | ATP5F1B |
| 8.89471129220058e-35 | -0.479283806 | 0.209 | 0.282 | 3.2089449928872e-30 | M2 macrophages | RPTOR |
| 1.00226037190122e-34 | 0.345431734751667 | 0.363 | 0.276 | 3.61585474370802e-30 | M2 macrophages | FGL2 |
| 1.02223540771285e-34 | -0.548529623 | 0.653 | 0.672 | 3.68791868040564e-30 | M2 macrophages | BMP2K |
| 1.02443790570207e-34 | 0.328365610541813 | 0.336 | 0.247 | 3.69586463240136e-30 | M2 macrophages | PSMA2 |
| 1.04845968728388e-34 | -0.324869457 | 0.356 | 0.434 | 3.78252801381406e-30 | M2 macrophages | BCAS3 |
| 1.12108997439688e-34 | -0.323817037 | 0.521 | 0.581 | 4.04455630063162e-30 | M2 macrophages | EXOC6B |
| 1.29242451135254e-34 | -0.318203973 | 0.446 | 0.514 | 4.66267990960654e-30 | M2 macrophages | BIRC6 |
| 1.37125905088193e-34 | -0.46486093 | 0.328 | 0.404 | 4.94709127786675e-30 | M2 macrophages | JARID2 |
| 2.02186910672642e-34 | 0.443148798188943 | 0.312 | 0.231 | 7.29429717633692e-30 | M2 macrophages | DNASE2 |
| 2.02539926115307e-34 | -0.444143242 | 0.327 | 0.399 | 7.30703291446193e-30 | M2 macrophages | VAV3 |
| 2.70255729672085e-34 | 0.322411672944087 | 0.486 | 0.39 | 9.7500159593798e-30 | M2 macrophages | C3AR1 |
| 2.78236733049381e-34 | -0.351112812 | 0.376 | 0.45 | 1.00379466182225e-29 | M2 macrophages | ANKRD44 |
| 2.91423909176546e-34 | 0.35182175631876 | 0.46 | 0.364 | 1.05137003713623e-29 | M2 macrophages | TMEM14B |
| 2.93122095667713e-34 | -0.497506086 | 0.185 | 0.254 | 1.05749658454041e-29 | M2 macrophages | PIK3CB |
| 3.0931951784141e-34 | -0.304047635 | 0.336 | 0.419 | 1.11593202451646e-29 | M2 macrophages | BRAF |
| 3.11281917098445e-34 | -0.399667129 | 0.563 | 0.61 | 1.12301177231606e-29 | M2 macrophages | ATP1B3 |
| 3.16015750766002e-34 | -0.566694868 | 0.311 | 0.377 | 1.14009002403851e-29 | M2 macrophages | SUSD6 |
| 3.33788167969888e-34 | 0.32670687394864 | 0.403 | 0.31 | 1.20420757358497e-29 | M2 macrophages | NAPA |
| 3.36215817911373e-34 | 0.305244528759766 | 0.482 | 0.399 | 1.21296580627886e-29 | M2 macrophages | SERPINB1 |
| 3.64141493244805e-34 | -0.681597237 | 0.245 | 0.305 | 1.31371326517928e-29 | M2 macrophages | BHLHE40 |
| 3.92739513196886e-34 | -0.467222785 | 0.365 | 0.426 | 1.41688634176041e-29 | M2 macrophages | FMNL1 |
| 4.02762525550405e-34 | 0.316840566854918 | 0.379 | 0.289 | 1.45304636342819e-29 | M2 macrophages | MLEC |
| 6.68459934191687e-34 | 0.444837316701586 | 0.445 | 0.358 | 2.41160290458335e-29 | M2 macrophages | TLR4 |
| 8.06280199551261e-34 | 0.303979567240796 | 0.303 | 0.219 | 2.90881707592108e-29 | M2 macrophages | NAGA |
| 1.41889601587574e-33 | 0.409612709052797 | 0.27 | 0.19 | 5.11895115647492e-29 | M2 macrophages | GPAA1 |
| 1.46148590410574e-33 | -0.644100988 | 0.187 | 0.254 | 5.27260269624228e-29 | M2 macrophages | PHC2 |
| 1.49580416408124e-33 | -0.546905199 | 0.283 | 0.348 | 5.3964126827559e-29 | M2 macrophages | CCDC93 |
| 1.67944213356656e-33 | -0.434355587 | 0.546 | 0.58 | 6.05892338526809e-29 | M2 macrophages | CEBPB |
| 1.75255804934411e-33 | 0.303319003695167 | 0.709 | 0.643 | 6.32270367461874e-29 | M2 macrophages | MAFB |
| 2.70086810220782e-33 | -0.441785291 | 0.343 | 0.41 | 9.74392185233515e-29 | M2 macrophages | MBD2 |
| 3.47157216583956e-33 | -0.320597396 | 0.491 | 0.549 | 1.25243909026994e-28 | M2 macrophages | PIAS1 |
| 3.50645009426367e-33 | -0.392778532 | 0.368 | 0.439 | 1.26502200050751e-28 | M2 macrophages | AKT3 |
| 3.71650850724985e-33 | 0.317736651586803 | 0.288 | 0.205 | 1.34080477416053e-28 | M2 macrophages | CACYBP |
| 3.74270652399656e-33 | 0.540038234082647 | 0.272 | 0.199 | 1.35025623266224e-28 | M2 macrophages | NAIP |
| 4.45911097776258e-33 | -0.429982806 | 0.287 | 0.359 | 1.60871346744741e-28 | M2 macrophages | LCOR |
| 4.54427377132936e-33 | -0.659585977 | 0.283 | 0.346 | 1.63943764848249e-28 | M2 macrophages | KDM6B |
| 4.656882935012e-33 | -0.556138508 | 0.331 | 0.396 | 1.68006365646428e-28 | M2 macrophages | CBLB |
| 5.62014237807319e-33 | -0.526852576 | 0.476 | 0.532 | 2.02757876573746e-28 | M2 macrophages | PSD3 |
| 5.90734026829348e-33 | -0.58055282 | 0.251 | 0.322 | 2.13119114859224e-28 | M2 macrophages | RTN1 |
| 7.40670277919697e-33 | 0.305692251992699 | 0.643 | 0.583 | 2.67211616165089e-28 | M2 macrophages | HSP90B1 |
| 8.09587175011676e-33 | -0.343419236 | 0.356 | 0.432 | 2.92074765128962e-28 | M2 macrophages | CDKAL1 |
| 8.9372464840989e-33 | 0.35213555179796 | 0.305 | 0.221 | 3.22429041406836e-28 | M2 macrophages | AGPAT2 |
| 1.77994797078222e-32 | -0.797651259 | 0.384 | 0.445 | 6.42151829419102e-28 | M2 macrophages | CCL3 |
| 1.99188585503461e-32 | -0.485710412 | 0.544 | 0.569 | 7.18612659920835e-28 | M2 macrophages | BTG1 |
| 2.21407893985473e-32 | -0.413012892 | 0.334 | 0.404 | 7.98773259131393e-28 | M2 macrophages | NFKBIZ |
| 2.34137742668331e-32 | -0.363121056 | 0.391 | 0.457 | 8.44698734224536e-28 | M2 macrophages | CTBP2 |
| 2.51993805116636e-32 | 0.500240520207062 | 0.478 | 0.394 | 9.09118050719288e-28 | M2 macrophages | PLXND1 |
| 2.56208105380521e-32 | 0.338486904157263 | 0.449 | 0.364 | 9.24321981781304e-28 | M2 macrophages | BCAP31 |
| 2.8600722378481e-32 | 0.332229992345148 | 0.516 | 0.439 | 1.03182826124846e-27 | M2 macrophages | TGOLN2 |
| 3.1997221550249e-32 | -0.464249385 | 0.243 | 0.315 | 1.15436376186833e-27 | M2 macrophages | LRCH1 |
| 3.384955432497e-32 | -0.40005468 | 0.264 | 0.334 | 1.22119037138194e-27 | M2 macrophages | MECP2 |
| 3.67391668356947e-32 | 0.306030683517882 | 0.479 | 0.396 | 1.32543892193136e-27 | M2 macrophages | ATP6AP2 |
| 3.77385641222021e-32 | 0.418116555818102 | 0.356 | 0.275 | 1.36149417783668e-27 | M2 macrophages | TNFRSF1B |
| 3.78246282794958e-32 | -0.30093312 | 0.559 | 0.637 | 1.36459911443937e-27 | M2 macrophages | LST1 |
| 4.03659634124624e-32 | -0.349595561 | 0.397 | 0.469 | 1.4562828620314e-27 | M2 macrophages | DENND4A |
| 5.24554143219718e-32 | 0.310608402816926 | 0.309 | 0.225 | 1.89243398249378e-27 | M2 macrophages | PNPLA2 |
| 6.47695753637488e-32 | -0.431137196 | 0.384 | 0.443 | 2.33669197039796e-27 | M2 macrophages | USP3 |
| 6.51434271683489e-32 | -0.431064435 | 0.213 | 0.283 | 2.35017942195252e-27 | M2 macrophages | TRAPPC10 |
| 6.53162404280032e-32 | 0.311212228340564 | 0.288 | 0.207 | 2.35641400592107e-27 | M2 macrophages | MPV17 |
| 7.20873354188654e-32 | -0.335513956 | 0.421 | 0.488 | 2.60069479990641e-27 | M2 macrophages | NSMCE2 |
| 9.04472373332788e-32 | 0.397538048762518 | 0.379 | 0.291 | 3.2630649812727e-27 | M2 macrophages | CALCOCO2 |
| 1.13176439266839e-31 | 0.345555175474938 | 0.307 | 0.226 | 4.08306639942975e-27 | M2 macrophages | MDH1 |
| 2.74197649444507e-31 | -0.389457852 | 0.236 | 0.309 | 9.89222859900948e-27 | M2 macrophages | CPEB3 |
| 2.88003379707283e-31 | 0.333442333010721 | 0.27 | 0.193 | 1.03902979296997e-26 | M2 macrophages | GM2A |
| 2.96078313179328e-31 | 0.321130061333165 | 0.348 | 0.261 | 1.06816173045706e-26 | M2 macrophages | TCEAL4 |
| 3.24815230742871e-31 | 0.306099408213551 | 0.286 | 0.205 | 1.17183590795106e-26 | M2 macrophages | FUNDC2 |
| 3.51347266834233e-31 | 0.334825505471255 | 0.612 | 0.519 | 1.26755553455786e-26 | M2 macrophages | CPM |
| 3.62216066531605e-31 | -0.555843535 | 0.235 | 0.302 | 1.30676690322607e-26 | M2 macrophages | WDFY4 |
| 3.73412433495027e-31 | -0.462979377 | 0.243 | 0.311 | 1.34716003632001e-26 | M2 macrophages | DENND1B |
| 3.89918084033268e-31 | 0.414882365615338 | 0.684 | 0.614 | 1.40670747176682e-26 | M2 macrophages | SLC9A9 |
| 6.2657589560294e-31 | 0.30525706371057 | 0.604 | 0.543 | 2.26049785856672e-26 | M2 macrophages | CALR |
| 6.90280976847592e-31 | 0.383177970209813 | 0.376 | 0.29 | 2.49032668017306e-26 | M2 macrophages | ARF4 |
| 7.93655844265906e-31 | -0.440917014 | 0.217 | 0.286 | 2.86327218935811e-26 | M2 macrophages | AVL9 |
| 1.01151755480692e-30 | 0.416216448609981 | 0.297 | 0.22 | 3.64925188247693e-26 | M2 macrophages | CCDC50 |
| 1.42025785341323e-30 | 0.336671538052774 | 0.335 | 0.251 | 5.12386425775892e-26 | M2 macrophages | SAT2 |
| 1.5743900750925e-30 | -0.388975632 | 0.292 | 0.359 | 5.67992707391123e-26 | M2 macrophages | ASAP2 |
| 1.65187191246573e-30 | 0.301210813489552 | 0.286 | 0.205 | 5.95945829860263e-26 | M2 macrophages | FAAP20 |
| 1.68167046344738e-30 | 0.350376056488986 | 0.409 | 0.319 | 6.06696253097912e-26 | M2 macrophages | FEZ2 |
| 2.01925495894184e-30 | -0.945975941 | 0.29 | 0.353 | 7.28486611537447e-26 | M2 macrophages | CCL4L2 |
| 2.34701829822819e-30 | 0.394381354844248 | 0.269 | 0.193 | 8.46733791451784e-26 | M2 macrophages | PARP1 |
| 2.69256471120036e-30 | -0.332950578 | 0.467 | 0.52 | 9.71396570859752e-26 | M2 macrophages | COP1 |
| 2.83291052461801e-30 | -0.405771526 | 0.348 | 0.411 | 1.02202912996644e-25 | M2 macrophages | C9orf72 |
| 4.03656553507384e-30 | -0.378163856 | 0.308 | 0.376 | 1.45627174808859e-25 | M2 macrophages | FTO |
| 4.92065510117917e-30 | 0.584693561292762 | 0.551 | 0.485 | 1.77522474085241e-25 | M2 macrophages | KLF4 |
| 4.9439720082825e-30 | 0.604634057137244 | 0.553 | 0.489 | 1.78363678142808e-25 | M2 macrophages | IER2 |
| 6.48707581368667e-30 | 0.305043400929448 | 0.252 | 0.178 | 2.34034234130374e-25 | M2 macrophages | POLR3GL |
| 7.33001924809562e-30 | -0.349900037 | 0.327 | 0.395 | 2.64445104413546e-25 | M2 macrophages | KIF13A |
| 7.90175813814621e-30 | -0.409501456 | 0.242 | 0.31 | 2.85071728349901e-25 | M2 macrophages | DLG1 |
| 8.74051267109096e-30 | 0.310732234073929 | 0.373 | 0.288 | 3.15331475634949e-25 | M2 macrophages | FUCA2 |
| 9.64519620117385e-30 | 0.367879036935909 | 0.496 | 0.403 | 3.47969743349749e-25 | M2 macrophages | TPCN1 |
| 1.01142527768733e-29 | -0.313149786 | 0.68 | 0.702 | 3.64891897431258e-25 | M2 macrophages | TNS3 |
| 1.02925206982489e-29 | -0.464443967 | 0.315 | 0.375 | 3.71323269230725e-25 | M2 macrophages | SPIRE1 |
| 1.25508850210185e-29 | 0.543456837440832 | 0.258 | 0.19 | 4.52798278903285e-25 | M2 macrophages | GLA |
| 1.38849282659265e-29 | -0.323642382 | 0.557 | 0.595 | 5.00926557049829e-25 | M2 macrophages | CD84 |
| 1.74224060239686e-29 | 0.406473795613323 | 0.38 | 0.296 | 6.28548142126715e-25 | M2 macrophages | ARHGAP4 |
| 1.76288502479636e-29 | -0.438343065 | 0.35 | 0.412 | 6.35996030395781e-25 | M2 macrophages | PHACTR2 |
| 2.11932874645292e-29 | 0.627974513084062 | 0.274 | 0.205 | 7.6459023185782e-25 | M2 macrophages | RCN3 |
| 3.11722730953833e-29 | 0.596122649868782 | 0.452 | 0.396 | 1.12460209646214e-24 | M2 macrophages | STARD13 |
| 3.13049788341311e-29 | -0.431594068 | 0.344 | 0.407 | 1.12938972139895e-24 | M2 macrophages | NOTCH2 |
| 4.50835336665463e-29 | -0.655416027 | 0.391 | 0.43 | 1.62647864408799e-24 | M2 macrophages | IL18 |
| 4.73218294582585e-29 | -0.394927069 | 0.381 | 0.435 | 1.70722964136559e-24 | M2 macrophages | COPA |
| 4.81897511595998e-29 | -0.31671514 | 0.636 | 0.663 | 1.73854165258488e-24 | M2 macrophages | PICALM |
| 4.93903327880664e-29 | -0.343092527 | 0.395 | 0.462 | 1.78185503599507e-24 | M2 macrophages | GPBP1 |
| 5.1776038713907e-29 | -0.370647625 | 0.471 | 0.518 | 1.86792414868162e-24 | M2 macrophages | VMP1 |
| 6.55504940692953e-29 | 0.301509276161344 | 0.354 | 0.271 | 2.36486517453797e-24 | M2 macrophages | HMGN1 |
| 6.6010431763381e-29 | -0.715234176 | 0.239 | 0.295 | 2.3814583467275e-24 | M2 macrophages | ACSL4 |
| 7.10407659923426e-29 | 0.363374252505537 | 0.414 | 0.331 | 2.56293771470574e-24 | M2 macrophages | LY96 |
| 1.1977732438454e-28 | -0.437320185 | 0.193 | 0.259 | 4.32120653182104e-24 | M2 macrophages | GPHN |
| 1.51667050818485e-28 | 0.476764496458498 | 0.319 | 0.243 | 5.4716921923785e-24 | M2 macrophages | APPL2 |
| 1.61644663427757e-28 | 0.358797342517471 | 0.289 | 0.215 | 5.83165452248317e-24 | M2 macrophages | SDC3 |
| 1.91099635558205e-28 | -0.358376722 | 0.195 | 0.262 | 6.89430155203337e-24 | M2 macrophages | ATAD2B |
| 2.07048439275258e-28 | -0.305846429 | 0.53 | 0.578 | 7.46968654373347e-24 | M2 macrophages | MACF1 |
| 2.63921824623993e-28 | -0.495389952 | 0.201 | 0.266 | 9.5215076669598e-24 | M2 macrophages | CDK6 |
| 3.15340067396301e-28 | -0.436332072 | 0.218 | 0.281 | 1.13765236114563e-23 | M2 macrophages | GMDS |
| 3.2451634203266e-28 | -0.328401191 | 0.554 | 0.593 | 1.17075760715123e-23 | M2 macrophages | ITGAM |
| 3.37287764620311e-28 | -0.409591421 | 0.238 | 0.305 | 1.2168330684207e-23 | M2 macrophages | RAPGEF6 |
| 4.6873672610549e-28 | 0.408379147430803 | 0.572 | 0.502 | 1.69106148677078e-23 | M2 macrophages | CLTC |
| 4.73113107707295e-28 | -0.663452762 | 0.245 | 0.296 | 1.70685015867561e-23 | M2 macrophages | UPP1 |
| 6.02801389821606e-28 | -0.98088905 | 0.327 | 0.363 | 2.17472657405941e-23 | M2 macrophages | PLIN2 |
| 6.13713515816687e-28 | -0.490778576 | 0.198 | 0.258 | 2.21409425101186e-23 | M2 macrophages | SUSD1 |
| 7.22216894691911e-28 | 0.521258252150831 | 0.261 | 0.195 | 2.60554189098001e-23 | M2 macrophages | TENT5A |
| 7.61452154938462e-28 | -0.30086138 | 0.543 | 0.581 | 2.74709093937149e-23 | M2 macrophages | CTNNA1 |
| 7.83244613648908e-28 | -0.387895212 | 0.404 | 0.453 | 2.82571159266116e-23 | M2 macrophages | RNF145 |
| 1.31839038237682e-27 | -0.302525983 | 0.262 | 0.329 | 4.75635698250086e-23 | M2 macrophages | CLASP1 |
| 1.3297276759994e-27 | -0.544009167 | 0.201 | 0.261 | 4.79725853670303e-23 | M2 macrophages | ITPKB |
| 1.57424277973455e-27 | -0.304271903 | 0.345 | 0.409 | 5.67939567644833e-23 | M2 macrophages | STK3 |
| 1.67466476122285e-27 | 0.309759629709831 | 0.403 | 0.318 | 6.04168805906369e-23 | M2 macrophages | PSMD8 |
| 1.68185897943768e-27 | -0.427602311 | 0.243 | 0.307 | 6.06764264011731e-23 | M2 macrophages | CAMKMT |
| 1.76562923032522e-27 | -0.302399658 | 0.389 | 0.465 | 6.3698605742443e-23 | M2 macrophages | SIPA1L1 |
| 2.45694103089393e-27 | -0.462481585 | 0.432 | 0.473 | 8.86390615715602e-23 | M2 macrophages | ZFAND5 |
| 2.63519858681467e-27 | -0.55694411 | 0.22 | 0.281 | 9.50700594165128e-23 | M2 macrophages | FAM168A |
| 2.7281088405869e-27 | -0.756769443 | 0.235 | 0.287 | 9.84219826418537e-23 | M2 macrophages | BASP1 |
| 3.52101234719085e-27 | -0.321184157 | 0.272 | 0.342 | 1.27027562449604e-22 | M2 macrophages | REV3L |
| 3.65033067951509e-27 | -0.482561521 | 0.211 | 0.27 | 1.31692979924866e-22 | M2 macrophages | IPO7 |
| 5.22275170851523e-27 | -0.360833754 | 0.195 | 0.26 | 1.88421213388104e-22 | M2 macrophages | POLR2J3 |
| 5.43405332555964e-27 | 0.33651820845564 | 0.306 | 0.231 | 1.96044341826215e-22 | M2 macrophages | SNX5 |
| 5.65728768205475e-27 | 0.349496651385416 | 0.723 | 0.754 | 2.04097967705489e-22 | M2 macrophages | HLA-DPA1 |
| 7.96204086437418e-27 | -0.351730332 | 0.445 | 0.493 | 2.87246548264027e-22 | M2 macrophages | DENND4C |
| 9.02445506514503e-27 | -0.369706509 | 0.42 | 0.468 | 3.25575265385237e-22 | M2 macrophages | CAB39 |
| 1.04043281940795e-26 | 0.322783516059788 | 0.313 | 0.236 | 3.75356948257807e-22 | M2 macrophages | RAB14 |
| 1.44373706537702e-26 | -0.38506274 | 0.486 | 0.532 | 5.20857021076069e-22 | M2 macrophages | AHR |
| 1.77695548088716e-26 | -0.435399533 | 0.313 | 0.367 | 6.41072228839662e-22 | M2 macrophages | ACTN4 |
| 1.9547828122843e-26 | -0.63163767 | 0.331 | 0.381 | 7.05226995187807e-22 | M2 macrophages | DLEU1 |
| 2.19116014746403e-26 | -0.440152649 | 0.267 | 0.327 | 7.90504846400597e-22 | M2 macrophages | ACER3 |
| 2.27770836737351e-26 | -0.32454048 | 0.312 | 0.378 | 8.21728847697341e-22 | M2 macrophages | GATAD2A |
| 2.56307545349134e-26 | -0.52813876 | 0.242 | 0.302 | 9.24680731356072e-22 | M2 macrophages | MOB3B |
| 2.58382133942434e-26 | -0.695952593 | 0.546 | 0.566 | 9.32165224624118e-22 | M2 macrophages | IER3 |
| 2.63463769584631e-26 | 0.319725384043329 | 0.361 | 0.28 | 9.50498241530475e-22 | M2 macrophages | ANP32A |
| 2.89548672953233e-26 | 0.301729193941412 | 0.452 | 0.365 | 1.04460474741338e-21 | M2 macrophages | UNC93B1 |
| 2.97456561177283e-26 | 0.375430883164799 | 0.279 | 0.209 | 1.07313403575929e-21 | M2 macrophages | HSD17B11 |
| 3.53552422613915e-26 | -0.339488286 | 0.361 | 0.426 | 1.27551107506422e-21 | M2 macrophages | FBXL17 |
| 4.22660967239495e-26 | 0.327052697108481 | 0.448 | 0.368 | 1.52483397150992e-21 | M2 macrophages | SEC62 |
| 4.46092649504684e-26 | -0.328431208 | 0.257 | 0.32 | 1.60936845161805e-21 | M2 macrophages | PCCA |
| 4.53901349155141e-26 | 0.307205015899065 | 0.52 | 0.435 | 1.637539897347e-21 | M2 macrophages | EIF5 |
| 4.54684345135974e-26 | -0.325309787 | 0.471 | 0.512 | 1.64036471194705e-21 | M2 macrophages | SND1 |
| 4.86541054669426e-26 | 0.319288468681346 | 0.357 | 0.278 | 1.75529416293089e-21 | M2 macrophages | YPEL5 |
| 5.39154832690982e-26 | 0.386396334006356 | 0.538 | 0.458 | 1.94510888989926e-21 | M2 macrophages | RIN2 |
| 5.60654302088029e-26 | 0.39894964593985 | 0.459 | 0.378 | 2.02267252564298e-21 | M2 macrophages | SRSF3 |
| 5.77320173670109e-26 | -0.35700172 | 0.407 | 0.456 | 2.08279799054965e-21 | M2 macrophages | SGK3 |
| 6.05640609875183e-26 | -0.352576601 | 0.245 | 0.309 | 2.1849696282467e-21 | M2 macrophages | PDSS2 |
| 6.66929438709593e-26 | -0.408281246 | 0.198 | 0.261 | 2.4060813360326e-21 | M2 macrophages | PRKCE |
| 6.78150993654948e-26 | -0.383912282 | 0.429 | 0.474 | 2.44656533980896e-21 | M2 macrophages | NUDT3 |
| 7.19736376412217e-26 | 0.830242744717945 | 0.287 | 0.238 | 2.59659292518236e-21 | M2 macrophages | SOX5 |
| 7.21834003632257e-26 | 0.401465842429311 | 0.324 | 0.249 | 2.60416053490409e-21 | M2 macrophages | SMIM7 |
| 1.08509347489912e-25 | 0.321833467619389 | 0.318 | 0.243 | 3.91469172939356e-21 | M2 macrophages | LTBR |
| 1.14490531401716e-25 | 0.505266679122671 | 0.347 | 0.276 | 4.13047490137971e-21 | M2 macrophages | CYTH4 |
| 1.22777399583179e-25 | -0.327044571 | 0.401 | 0.457 | 4.42944024476235e-21 | M2 macrophages | TBL1XR1 |
| 1.23369871358371e-25 | -0.470513435 | 0.242 | 0.3 | 4.45081484899597e-21 | M2 macrophages | PLCG2 |
| 1.31199965783573e-25 | 0.35080457581349 | 0.299 | 0.226 | 4.73330116557398e-21 | M2 macrophages | TM6SF1 |
| 1.31519647483704e-25 | -0.411253383 | 0.357 | 0.405 | 4.7448343222696e-21 | M2 macrophages | ANKRD10 |
| 1.35297753979106e-25 | 0.305933708257933 | 0.51 | 0.452 | 4.88113707030422e-21 | M2 macrophages | CTSH |
| 1.46688142865769e-25 | -0.384662303 | 0.355 | 0.412 | 5.29206813016836e-21 | M2 macrophages | FAM172A |
| 1.92703030686738e-25 | -0.518959447 | 0.312 | 0.363 | 6.95214723808544e-21 | M2 macrophages | IKZF1 |
| 1.94613708074514e-25 | 0.306994372388345 | 0.443 | 0.364 | 7.02107874620425e-21 | M2 macrophages | TRAM1 |
| 2.42223204525824e-25 | 0.368732720921925 | 0.261 | 0.194 | 8.73868654967816e-21 | M2 macrophages | CALML4 |
| 2.46863529326503e-25 | -0.403254675 | 0.25 | 0.307 | 8.90609554751226e-21 | M2 macrophages | DOCK1 |
| 2.80029332517664e-25 | -0.394828442 | 0.21 | 0.269 | 1.01026182292398e-20 | M2 macrophages | AOPEP |
| 4.38730213986758e-25 | -0.449989476 | 0.249 | 0.308 | 1.58280699300003e-20 | M2 macrophages | SERTAD2 |
| 4.7230562367192e-25 | -0.366005956 | 0.29 | 0.35 | 1.70393699852118e-20 | M2 macrophages | ARFGEF1 |
| 5.46112527014187e-25 | 0.333569292239887 | 0.354 | 0.278 | 1.97021016370908e-20 | M2 macrophages | PRKAR1A |
| 5.57900664499917e-25 | -0.668818996 | 0.254 | 0.303 | 2.01273822731635e-20 | M2 macrophages | RALA |
| 5.58905238835438e-25 | -0.310116819 | 0.306 | 0.37 | 2.01636243014661e-20 | M2 macrophages | TAB2 |
| 7.47826760322173e-25 | 0.30746339584026 | 0.404 | 0.323 | 2.6979346032143e-20 | M2 macrophages | HNRNPUL1 |
| 7.83443223724828e-25 | 0.34632182829619 | 0.267 | 0.198 | 2.82642811823206e-20 | M2 macrophages | PNKD |
| 8.34031640427576e-25 | -0.439861898 | 0.304 | 0.36 | 3.00893594917057e-20 | M2 macrophages | CYRIA |
| 9.44229717557646e-25 | 0.35724423948138 | 0.317 | 0.243 | 3.40649755203272e-20 | M2 macrophages | SSR1 |
| 1.08526224298913e-24 | -0.643455087 | 0.232 | 0.283 | 3.91530059403187e-20 | M2 macrophages | ABCG1 |
| 1.1097748465803e-24 | -0.358828077 | 0.237 | 0.298 | 4.00373471400773e-20 | M2 macrophages | PPARD |
| 1.13350178335329e-24 | -0.397640511 | 0.305 | 0.362 | 4.08933438380366e-20 | M2 macrophages | SLC25A13 |
| 1.30555701564327e-24 | -0.554863985 | 0.254 | 0.306 | 4.71005804533622e-20 | M2 macrophages | GRK5 |
| 1.38169640670697e-24 | 0.374300484320848 | 0.49 | 0.41 | 4.98474612647674e-20 | M2 macrophages | IFI16 |
| 1.62448629201666e-24 | -0.429365754 | 0.197 | 0.256 | 5.86065919570852e-20 | M2 macrophages | INVS |
| 2.21354071427841e-24 | -0.390648401 | 0.414 | 0.467 | 7.98579083490221e-20 | M2 macrophages | IMMP2L |
| 2.47502263345168e-24 | -0.57917831 | 0.237 | 0.288 | 8.92913915470363e-20 | M2 macrophages | HK1 |
| 2.95862229684483e-24 | -0.353714972 | 0.355 | 0.41 | 1.06738216603271e-19 | M2 macrophages | UBAP1 |
| 3.35268639658719e-24 | 0.57690129574386 | 0.647 | 0.678 | 1.20954867129676e-19 | M2 macrophages | HLA-DRB5 |
| 3.49981312660449e-24 | -0.345089156 | 0.649 | 0.664 | 1.2626275816851e-19 | M2 macrophages | LYN |
| 3.79288401114521e-24 | 0.302524218425356 | 0.251 | 0.184 | 1.36835876470086e-19 | M2 macrophages | HSD17B12 |
| 5.54289403511922e-24 | -0.463961742 | 0.266 | 0.32 | 1.99970988104996e-19 | M2 macrophages | CXCL8 |
| 6.28564699759987e-24 | -0.374711969 | 0.262 | 0.322 | 2.2676728673241e-19 | M2 macrophages | CERS6 |
| 6.39494665152253e-24 | -0.530360398 | 0.274 | 0.324 | 2.30710490346978e-19 | M2 macrophages | LPCAT2 |
| 7.60141145699613e-24 | -0.367932656 | 0.217 | 0.277 | 2.74236121134049e-19 | M2 macrophages | SMURF1 |
| 8.41813138382326e-24 | -0.458552599 | 0.334 | 0.388 | 3.03700925934192e-19 | M2 macrophages | GSAP |
| 1.12932428881625e-23 | 0.322670320051422 | 0.324 | 0.249 | 4.07426323676239e-19 | M2 macrophages | EIF1B |
| 1.36468164264183e-23 | 0.398657086220785 | 0.296 | 0.228 | 4.92336196215892e-19 | M2 macrophages | SERTAD1 |
| 1.44415637595211e-23 | -0.416971239 | 0.356 | 0.399 | 5.21008295752242e-19 | M2 macrophages | P2RX4 |
| 1.53804910909479e-23 | -0.536484092 | 0.291 | 0.336 | 5.54881977088128e-19 | M2 macrophages | PEA15 |
| 1.58911455398801e-23 | -0.421031296 | 0.44 | 0.484 | 5.73304857642254e-19 | M2 macrophages | RHOQ |
| 1.64496437598365e-23 | -0.421689127 | 0.202 | 0.26 | 5.9345379792362e-19 | M2 macrophages | SPATA5 |
| 1.86822354260467e-23 | -0.329269085 | 0.444 | 0.491 | 6.73999007465485e-19 | M2 macrophages | ZMIZ1 |
| 2.27209037678467e-23 | -0.404894833 | 0.271 | 0.327 | 8.19702045232606e-19 | M2 macrophages | FARS2 |
| 3.27033542473322e-23 | -0.388637172 | 0.44 | 0.481 | 1.179838911181e-18 | M2 macrophages | SGK1 |
| 3.33202545590028e-23 | -0.386301667 | 0.197 | 0.255 | 1.20209482372514e-18 | M2 macrophages | DIS3L2 |
| 3.55254386411266e-23 | 0.310436866565178 | 0.535 | 0.457 | 1.28165124985592e-18 | M2 macrophages | TGFB1 |
| 4.66511638807718e-23 | 0.323196209619461 | 0.301 | 0.229 | 1.68303403932661e-18 | M2 macrophages | PPP2R1A |
| 4.85338882382978e-23 | -0.306824786 | 0.301 | 0.362 | 1.75095708597307e-18 | M2 macrophages | STRN3 |
| 8.27773827441086e-23 | -0.42050912 | 0.25 | 0.307 | 2.98635963725921e-18 | M2 macrophages | USP25 |
| 8.8095925229257e-23 | -0.303546547 | 0.286 | 0.346 | 3.1782366944959e-18 | M2 macrophages | ARMH3 |
| 1.37547005637729e-22 | 0.371745467975627 | 0.508 | 0.435 | 4.96228332239234e-18 | M2 macrophages | DNAJA1 |
| 2.23845590815091e-22 | -0.35844738 | 0.495 | 0.528 | 8.07567737983602e-18 | M2 macrophages | CCDC91 |
| 2.41201328386304e-22 | 0.404036628551176 | 0.518 | 0.452 | 8.70182032419269e-18 | M2 macrophages | TACC1 |
| 3.63469839489343e-22 | -0.342098429 | 0.215 | 0.273 | 1.3112901399257e-17 | M2 macrophages | AHI1 |
| 3.73271331093888e-22 | -0.335087405 | 0.361 | 0.407 | 1.34665098118742e-17 | M2 macrophages | PPFIA1 |
| 3.94920570119958e-22 | -0.377181418 | 0.407 | 0.445 | 1.42475494082177e-17 | M2 macrophages | BABAM2 |
| 3.98014909410119e-22 | 0.509253974537254 | 0.328 | 0.268 | 1.43591838867889e-17 | M2 macrophages | SESN1 |
| 4.32390459692876e-22 | -0.421704575 | 0.206 | 0.259 | 1.55993506143399e-17 | M2 macrophages | ATR |
| 4.452869401678e-22 | -0.363888701 | 0.278 | 0.335 | 1.60646169404337e-17 | M2 macrophages | AFTPH |
| 5.62217685332951e-22 | 0.373537230679568 | 0.324 | 0.258 | 2.02831274337569e-17 | M2 macrophages | SEC14L1 |
| 7.06820505508233e-22 | -0.369498214 | 0.224 | 0.28 | 2.54999633772205e-17 | M2 macrophages | MOB1B |
| 7.22916018516752e-22 | 0.601559951093412 | 0.278 | 0.22 | 2.60806412000289e-17 | M2 macrophages | ARID3A |
| 7.38052213392137e-22 | 0.435000968744672 | 0.272 | 0.208 | 2.66267097025481e-17 | M2 macrophages | DCAF12 |
| 1.21521439075881e-21 | -0.60948034 | 0.227 | 0.273 | 4.38412895754057e-17 | M2 macrophages | PCED1B |
| 1.39553752580209e-21 | -0.36557522 | 0.513 | 0.539 | 5.03468073183622e-17 | M2 macrophages | LCP2 |
| 1.73407695640508e-21 | -0.329770781 | 0.259 | 0.317 | 6.2560294356226e-17 | M2 macrophages | XPR1 |
| 1.96538777375158e-21 | 0.372610873170387 | 0.858 | 0.812 | 7.09052947136358e-17 | M2 macrophages | FRMD4B |
| 2.59963086219398e-21 | 0.36292784691353 | 0.252 | 0.19 | 9.37868826153721e-17 | M2 macrophages | CCDC47 |
| 2.8851941318534e-21 | -0.454802106 | 0.262 | 0.31 | 1.04089148694875e-16 | M2 macrophages | TAF1D |
| 3.30686898589422e-21 | -0.302650607 | 0.317 | 0.371 | 1.19301912404106e-16 | M2 macrophages | SMG1 |
| 4.83180914919276e-21 | 0.594988889297935 | 0.553 | 0.489 | 1.74317178675427e-16 | M2 macrophages | FOSB |
| 5.13748525243908e-21 | -0.418252117 | 0.341 | 0.385 | 1.85345055452245e-16 | M2 macrophages | SEPTIN9 |
| 5.27169172327196e-21 | -0.544780948 | 0.237 | 0.282 | 1.90186822300483e-16 | M2 macrophages | CPPED1 |
| 6.16474977529318e-21 | -0.409959173 | 0.526 | 0.541 | 2.22405677643252e-16 | M2 macrophages | SNX24 |
| 8.17511156311106e-21 | 0.309135265019078 | 0.38 | 0.308 | 2.94933499862358e-16 | M2 macrophages | NAGK |
| 8.24198180924195e-21 | 0.373197644230239 | 0.303 | 0.238 | 2.97345977732022e-16 | M2 macrophages | CYBRD1 |
| 9.75651064178314e-21 | -0.39131337 | 0.467 | 0.494 | 3.5198563442361e-16 | M2 macrophages | ALOX5 |
| 1.01891819058561e-20 | -0.455445066 | 0.252 | 0.299 | 3.67595115617571e-16 | M2 macrophages | FGD5 |
| 1.13636465521224e-20 | -0.387009266 | 0.371 | 0.411 | 4.09966276660918e-16 | M2 macrophages | EFHD2 |
| 1.27707012202059e-20 | -0.376481666 | 0.204 | 0.256 | 4.60728587921369e-16 | M2 macrophages | RNF19A |
| 1.61972718610724e-20 | -0.551656035 | 0.218 | 0.263 | 5.84348976931908e-16 | M2 macrophages | IRAK1 |
| 2.06706663511359e-20 | -0.405227934 | 0.47 | 0.496 | 7.45735629949931e-16 | M2 macrophages | ARHGEF10L |
| 2.22612871222257e-20 | -0.321741862 | 0.402 | 0.442 | 8.03120455508536e-16 | M2 macrophages | CAPN2 |
| 2.36278700293549e-20 | -0.433889896 | 0.484 | 0.524 | 8.52422667049035e-16 | M2 macrophages | FMN1 |
| 2.56456982447119e-20 | -0.381270716 | 0.309 | 0.358 | 9.25219855574473e-16 | M2 macrophages | SMAD2 |
| 3.25877066508452e-20 | -0.421092274 | 0.414 | 0.442 | 1.17566669284254e-15 | M2 macrophages | ITGB5 |
| 3.89070009188872e-20 | -0.379200656 | 0.297 | 0.348 | 1.40364787215069e-15 | M2 macrophages | RPS6KC1 |
| 5.22562776104093e-20 | -0.33041989 | 0.471 | 0.507 | 1.88524972735074e-15 | M2 macrophages | EZR |
| 6.43562354181259e-20 | -0.336618873 | 0.314 | 0.369 | 2.32177990517973e-15 | M2 macrophages | NLK |
| 6.60215812880291e-20 | 0.582142562854381 | 0.258 | 0.208 | 2.38186058812822e-15 | M2 macrophages | TBC1D9 |
| 8.09438166187066e-20 | -0.327071214 | 0.299 | 0.349 | 2.92021007215308e-15 | M2 macrophages | GMDS-DT |
| 9.39398118179236e-20 | -0.340020365 | 0.316 | 0.364 | 3.38906659095523e-15 | M2 macrophages | WWC2 |
| 1.28264475503823e-19 | -0.305949884 | 0.608 | 0.63 | 4.62739748275143e-15 | M2 macrophages | CD81 |
| 1.33161703376852e-19 | -0.381300475 | 0.338 | 0.382 | 4.80407477272669e-15 | M2 macrophages | CLIP4 |
| 1.35975783435439e-19 | 0.326038277797587 | 0.302 | 0.238 | 4.90559833900032e-15 | M2 macrophages | PARL |
| 1.45997220263625e-19 | -0.473316433 | 0.272 | 0.315 | 5.2671417154508e-15 | M2 macrophages | CSK |
| 1.52273142795321e-19 | -0.450426644 | 0.203 | 0.252 | 5.49355817262679e-15 | M2 macrophages | SSH1 |
| 1.76539260683506e-19 | -0.386283713 | 0.346 | 0.384 | 6.36900690767885e-15 | M2 macrophages | STX12 |
| 2.90945954580693e-19 | -0.315161984 | 0.341 | 0.384 | 1.04964572034077e-14 | M2 macrophages | CHCHD3 |
| 2.97460798795227e-19 | -0.469412918 | 0.356 | 0.393 | 1.07314932381354e-14 | M2 macrophages | TCIRG1 |
| 3.38821430221798e-19 | 0.434801625803856 | 0.349 | 0.292 | 1.22236607381118e-14 | M2 macrophages | CEMIP2 |
| 3.44399599101708e-19 | -0.309123954 | 0.365 | 0.41 | 1.24249043367923e-14 | M2 macrophages | GSK3B |
| 3.52451999415558e-19 | -0.477436191 | 0.353 | 0.388 | 1.27154107829151e-14 | M2 macrophages | LRRFIP2 |
| 4.78368220515795e-19 | -0.330409986 | 0.27 | 0.323 | 1.72580902915483e-14 | M2 macrophages | WDPCP |
| 5.20010385911509e-19 | -0.341233644 | 0.26 | 0.316 | 1.87604146925295e-14 | M2 macrophages | RYBP |
| 5.49216428778999e-19 | -0.319323727 | 0.283 | 0.334 | 1.981408110106e-14 | M2 macrophages | THADA |
| 5.76418986251984e-19 | -0.490908214 | 0.248 | 0.293 | 2.07954677670128e-14 | M2 macrophages | ST3GAL6 |
| 6.54685865285388e-19 | -0.355628349 | 0.222 | 0.272 | 2.36191019619009e-14 | M2 macrophages | CBFB |
| 1.36059699840153e-18 | -0.324894175 | 0.339 | 0.383 | 4.90862579113319e-14 | M2 macrophages | SKI |
| 1.69851697032497e-18 | 0.330490628075627 | 0.453 | 0.4 | 6.1277396738414e-14 | M2 macrophages | NCF1 |
| 2.25388108855468e-18 | 0.309380040164816 | 0.294 | 0.229 | 8.1313268031787e-14 | M2 macrophages | TNFRSF14 |
| 2.55913813713255e-18 | -0.300283426 | 0.213 | 0.265 | 9.23260265733311e-14 | M2 macrophages | SCLT1 |
| 2.71263157565639e-18 | -0.455541843 | 0.357 | 0.391 | 9.78636093549557e-14 | M2 macrophages | EYA2 |
| 3.13765476720433e-18 | -0.369429242 | 0.26 | 0.306 | 1.13197171036431e-13 | M2 macrophages | CUL4A |
| 3.96569855332746e-18 | 0.311835723927924 | 0.271 | 0.216 | 1.43070506708395e-13 | M2 macrophages | FGFR1 |
| 4.97789198186739e-18 | -0.402426825 | 0.312 | 0.359 | 1.7958740902983e-13 | M2 macrophages | RAD51B |
| 5.64439806290526e-18 | -0.360053799 | 0.426 | 0.466 | 2.03632948915433e-13 | M2 macrophages | CTNNB1 |
| 6.58663298948257e-18 | 0.380633839390481 | 0.367 | 0.308 | 2.37625958361563e-13 | M2 macrophages | PER3 |
| 7.73140744367749e-18 | -0.373863468 | 0.226 | 0.272 | 2.78925986345553e-13 | M2 macrophages | DOP1B |
| 9.81836774266898e-18 | 0.323158609897043 | 0.31 | 0.249 | 3.54217253052269e-13 | M2 macrophages | NORAD |
| 9.85170002484199e-18 | -0.35087635 | 0.384 | 0.418 | 3.55419781796224e-13 | M2 macrophages | RSRC1 |
| 9.94664572268492e-18 | 0.321675763627069 | 0.334 | 0.269 | 3.58845137737304e-13 | M2 macrophages | ATP6V1D |
| 1.67501563576121e-17 | 0.405966687307286 | 0.28 | 0.225 | 6.04295390913574e-13 | M2 macrophages | KLF10 |
| 2.12432186102867e-17 | -0.370094848 | 0.236 | 0.279 | 7.66391597803314e-13 | M2 macrophages | GGA1 |
| 2.68243449159448e-17 | -0.31836988 | 0.239 | 0.285 | 9.67741891532539e-13 | M2 macrophages | PPP4R3A |
| 3.85964138179076e-17 | -0.524833876 | 0.238 | 0.278 | 1.39244282130865e-12 | M2 macrophages | SLC8B1 |
| 3.95832834486682e-17 | 0.847919875445132 | 0.334 | 0.291 | 1.4280461169776e-12 | M2 macrophages | SEMA4A |
| 5.58275250738647e-17 | -0.417196773 | 0.393 | 0.424 | 2.01408962208982e-12 | M2 macrophages | TGFBR1 |
| 5.66101463583637e-17 | 0.347781865350511 | 0.774 | 0.836 | 2.04232425017069e-12 | M2 macrophages | HLA-DRB1 |
| 5.83805092970256e-17 | -0.311709641 | 0.489 | 0.521 | 2.10619363390879e-12 | M2 macrophages | FKBP5 |
| 6.29372149041058e-17 | 0.47003564118234 | 0.301 | 0.251 | 2.27058590209542e-12 | M2 macrophages | IVNS1ABP |
| 6.95894225802084e-17 | -0.487665906 | 0.23 | 0.269 | 2.51057759842618e-12 | M2 macrophages | GTF2I |
| 7.05805134448267e-17 | -0.307600227 | 0.373 | 0.41 | 2.54633318354901e-12 | M2 macrophages | UBL3 |
| 7.22035362703283e-17 | -0.331484507 | 0.21 | 0.257 | 2.60488697802464e-12 | M2 macrophages | BICD1 |
| 1.01136320285614e-16 | -0.379531627 | 0.307 | 0.343 | 3.64869502694408e-12 | M2 macrophages | RIOK3 |
| 1.05800372144584e-16 | -0.35124123 | 0.223 | 0.268 | 3.81696002586014e-12 | M2 macrophages | SEC24D |
| 1.0817214992579e-16 | -0.343522283 | 0.244 | 0.29 | 3.90252665287273e-12 | M2 macrophages | N4BP1 |
| 1.13654022450723e-16 | -0.34124541 | 0.242 | 0.287 | 4.10029616795474e-12 | M2 macrophages | ALPK1 |
| 1.33811691981196e-16 | -0.326120902 | 0.513 | 0.531 | 4.8275244116056e-12 | M2 macrophages | LITAF |
| 1.6464273134326e-16 | -0.537135948 | 0.382 | 0.425 | 5.93981581867079e-12 | M2 macrophages | CCL4 |
| 2.00443337599036e-16 | -0.303219345 | 0.242 | 0.286 | 7.23139429056042e-12 | M2 macrophages | CDC42BPB |
| 2.01545465534764e-16 | 0.362820807628237 | 0.292 | 0.237 | 7.27115576009768e-12 | M2 macrophages | RELL1 |
| 3.18931659227696e-16 | -0.315220893 | 0.249 | 0.293 | 1.15060974699576e-11 | M2 macrophages | MAP2K3 |
| 3.68321982631295e-16 | -0.315825622 | 0.374 | 0.408 | 1.32879521673892e-11 | M2 macrophages | ADAM10 |
| 4.69263451883544e-16 | -0.313042852 | 0.307 | 0.35 | 1.69296175536026e-11 | M2 macrophages | IL6R |
| 6.62219787606655e-16 | 0.526866437358349 | 0.288 | 0.241 | 2.38909032774853e-11 | M2 macrophages | ARHGAP5 |
| 8.93717635349263e-16 | -0.349475988 | 0.25 | 0.292 | 3.22426511304954e-11 | M2 macrophages | AZIN1 |
| 9.24121938342743e-16 | 0.384212611437767 | 0.483 | 0.429 | 3.33395471695911e-11 | M2 macrophages | TRA2B |
| 9.7406310218075e-16 | 0.331761559552626 | 0.33 | 0.272 | 3.51412745373749e-11 | M2 macrophages | RESF1 |
| 1.06698089193917e-15 | 0.438275162566188 | 0.31 | 0.261 | 3.84934696384895e-11 | M2 macrophages | MS4A4E |
| 1.18116157504197e-15 | -0.301436828 | 0.236 | 0.28 | 4.26127661427893e-11 | M2 macrophages | ECPAS |
| 1.6664324212472e-15 | -0.301502711 | 0.276 | 0.32 | 6.01198824613351e-11 | M2 macrophages | SYNE1 |
| 1.8622962916699e-15 | -0.548072977 | 0.276 | 0.312 | 6.71860633145751e-11 | M2 macrophages | RND3 |
| 2.11787808849962e-15 | 0.583088685689705 | 0.44 | 0.403 | 7.64066877988008e-11 | M2 macrophages | DIP2B |
| 2.20264780927255e-15 | -0.361421458 | 0.21 | 0.253 | 7.94649250151256e-11 | M2 macrophages | SREBF2 |
| 2.40004576157179e-15 | -0.375458913 | 0.39 | 0.416 | 8.65864509402255e-11 | M2 macrophages | HAVCR2 |
| 2.40938048464832e-15 | 0.327023397958898 | 0.353 | 0.298 | 8.69232197446575e-11 | M2 macrophages | TTYH3 |
| 2.61749557458573e-15 | 0.408178146295041 | 0.308 | 0.258 | 9.44313878443292e-11 | M2 macrophages | STK17B |
| 2.82214967358256e-15 | -0.621606393 | 0.456 | 0.467 | 1.01814693773838e-10 | M2 macrophages | SMS |
| 3.02653656156539e-15 | -0.310219207 | 0.268 | 0.31 | 1.09188359531595e-10 | M2 macrophages | RAB3GAP1 |
| 4.02803201999428e-15 | 0.39797799436286 | 0.316 | 0.261 | 1.45319311185334e-10 | M2 macrophages | VAV1 |
| 4.08081834020285e-15 | -0.339804009 | 0.279 | 0.318 | 1.47223683259498e-10 | M2 macrophages | HGSNAT |
| 5.33046118168086e-15 | 0.302991474492711 | 0.696 | 0.648 | 1.923070480515e-10 | M2 macrophages | JUNB |
| 5.91918964416441e-15 | -0.350179275 | 0.506 | 0.516 | 2.13546604792519e-10 | M2 macrophages | PPP1R15A |
| 7.20986142435203e-15 | -0.463925595 | 0.254 | 0.289 | 2.60110170606348e-10 | M2 macrophages | LILRB1 |
| 7.31811727840606e-15 | -0.451665448 | 0.213 | 0.251 | 2.64015717053056e-10 | M2 macrophages | PAPSS1 |
| 1.9185164894164e-14 | -0.313633051 | 0.312 | 0.348 | 6.92143193886756e-10 | M2 macrophages | DDX21 |
| 2.2443940262465e-14 | -0.524840327 | 0.22 | 0.256 | 8.0971003284895e-10 | M2 macrophages | FPR1 |
| 2.82373842671382e-14 | 0.395031661449644 | 0.474 | 0.423 | 1.01872011220554e-09 | M2 macrophages | DDX3X |
| 3.07529477795931e-14 | -0.442903371 | 0.26 | 0.295 | 1.10947409704438e-09 | M2 macrophages | IGF2R |
| 3.95726144914924e-14 | -0.352787977 | 0.22 | 0.264 | 1.42766121300957e-09 | M2 macrophages | HMBOX1 |
| 4.07733053946644e-14 | -0.35709956 | 0.259 | 0.301 | 1.47097853872331e-09 | M2 macrophages | AGPAT4 |
| 4.67638067111065e-14 | -0.392158698 | 0.328 | 0.358 | 1.68709785471659e-09 | M2 macrophages | MSRA |
| 5.0190319087562e-14 | -0.345066813 | 0.278 | 0.313 | 1.81071614172197e-09 | M2 macrophages | VPS41 |
| 5.86649524332199e-14 | -0.303033231 | 0.265 | 0.308 | 2.11645548893327e-09 | M2 macrophages | USP47 |
| 6.12044157835445e-14 | -0.300417942 | 0.437 | 0.459 | 2.20807170822293e-09 | M2 macrophages | SNX10 |
| 7.22863828199884e-14 | -0.64858006 | 0.281 | 0.311 | 2.60787583299672e-09 | M2 macrophages | INSIG1 |
| 1.0531408397923e-13 | -0.307782597 | 0.6 | 0.607 | 3.79941620771867e-09 | M2 macrophages | PTPRC |
| 1.22008772913792e-13 | -0.329282149 | 0.23 | 0.27 | 4.40171050041089e-09 | M2 macrophages | STK24 |
| 1.22274926855131e-13 | 0.366260114178372 | 0.268 | 0.223 | 4.41131253615256e-09 | M2 macrophages | RFX2 |
| 1.87284413461799e-13 | -0.34631357 | 0.356 | 0.38 | 6.75665978446134e-09 | M2 macrophages | SQOR |
| 1.90620383345011e-13 | -0.378109035 | 0.232 | 0.27 | 6.87701156993798e-09 | M2 macrophages | LRRC8C |
| 2.34169894790337e-13 | -0.571367634 | 0.31 | 0.336 | 8.44814729435098e-09 | M2 macrophages | ST8SIA4 |
| 4.84453882318095e-13 | -0.380937177 | 0.279 | 0.315 | 1.74776427123899e-08 | M2 macrophages | PHF21A |
| 4.91857863967729e-13 | 0.313009851606406 | 0.453 | 0.399 | 1.77447561583637e-08 | M2 macrophages | GLUD1 |
| 5.40051085134183e-13 | -0.326335181 | 0.259 | 0.293 | 1.94834229983859e-08 | M2 macrophages | PPHLN1 |
| 6.73701815538294e-13 | -0.424880741 | 0.327 | 0.359 | 2.4305140399175e-08 | M2 macrophages | CD9 |
| 9.1522269885262e-13 | -0.343495212 | 0.282 | 0.32 | 3.3018489306506e-08 | M2 macrophages | SAMD4A |
| 9.42264395900145e-13 | -0.441940771 | 0.268 | 0.298 | 3.39940726108895e-08 | M2 macrophages | OLA1 |
| 1.09405992910985e-12 | -0.439668158 | 0.249 | 0.282 | 3.94704000624959e-08 | M2 macrophages | GTF2F2 |
| 1.1040926390127e-12 | 0.314736787085721 | 0.552 | 0.493 | 3.98323501376612e-08 | M2 macrophages | SGMS1 |
| 1.44051534363713e-12 | -0.377658165 | 0.417 | 0.433 | 5.19694720523968e-08 | M2 macrophages | FLOT1 |
| 1.62131153764244e-12 | -0.309086857 | 0.374 | 0.407 | 5.84920563435262e-08 | M2 macrophages | ENSG00000226149 |
| 1.83616963335333e-12 | -0.353164017 | 0.253 | 0.29 | 6.62434918624881e-08 | M2 macrophages | DEFB1 |
| 2.27754327625808e-12 | -0.310290749 | 0.321 | 0.354 | 8.21669287775627e-08 | M2 macrophages | CBL |
| 2.53450079635788e-12 | -0.609923343 | 0.466 | 0.475 | 9.14371852302031e-08 | M2 macrophages | ME2 |
| 2.89395662219474e-12 | 0.364515385955949 | 0.31 | 0.264 | 1.04405273058919e-07 | M2 macrophages | MAPRE2 |
| 3.63282228548229e-12 | -0.322378869 | 0.304 | 0.336 | 1.31061329593345e-07 | M2 macrophages | CDKN1A |
| 4.5738465492175e-12 | 0.334915996152939 | 0.297 | 0.25 | 1.6501066195612e-07 | M2 macrophages | GTF2B |
| 5.96505588234626e-12 | -0.316651631 | 0.322 | 0.35 | 2.15201321067406e-07 | M2 macrophages | RXRA |
| 7.25633694816792e-12 | -0.388078256 | 0.306 | 0.337 | 2.61786868079054e-07 | M2 macrophages | RGCC |
| 7.31511775750866e-12 | -0.318094326 | 0.26 | 0.296 | 2.6390750333764e-07 | M2 macrophages | TRPM7 |
| 8.9803394311568e-12 | -0.410165142 | 0.228 | 0.26 | 3.23983705657844e-07 | M2 macrophages | IL17RA |
| 2.02783819875346e-11 | -0.517289194 | 0.484 | 0.491 | 7.31583186964285e-07 | M2 macrophages | MERTK |
| 2.09085987722473e-11 | -0.441795171 | 0.365 | 0.384 | 7.54319517906367e-07 | M2 macrophages | ENTPD1 |
| 2.93205575201602e-11 | 0.373315847329764 | 0.267 | 0.225 | 1.05779775365482e-06 | M2 macrophages | GCNT1 |
| 7.47068997996992e-11 | -0.402574562 | 0.463 | 0.469 | 2.69520082407375e-06 | M2 macrophages | ASPH |
| 1.12705410713648e-10 | -0.340759167 | 0.548 | 0.549 | 4.06607310231629e-06 | M2 macrophages | MARCHF1 |
| 1.89893801855002e-10 | -0.444215823 | 0.303 | 0.331 | 6.8507986895229e-06 | M2 macrophages | TNFAIP8 |
| 2.11801199780983e-10 | -0.397830186 | 0.29 | 0.315 | 7.64115188449852e-06 | M2 macrophages | ATP6V1C1 |
| 2.35459641979652e-10 | 0.340695075690388 | 0.267 | 0.226 | 8.49467750369992e-06 | M2 macrophages | MAT2A |
| 4.29727745042846e-10 | -0.336024011 | 0.42 | 0.431 | 1.55032878579107e-05 | M2 macrophages | CD47 |
| 5.38135738949534e-10 | -0.326614285 | 0.235 | 0.266 | 1.94143230540824e-05 | M2 macrophages | MBOAT1 |
| 6.45753654082757e-10 | -0.353159762 | 0.386 | 0.399 | 2.32968545783436e-05 | M2 macrophages | SLC38A6 |
| 6.70769120513619e-10 | -0.307056234 | 0.24 | 0.269 | 2.41993375607698e-05 | M2 macrophages | RDX |
| 6.97220633710588e-10 | 0.398382031558336 | 0.605 | 0.568 | 2.51536288023769e-05 | M2 macrophages | EMP1 |
| 1.06844365088902e-09 | 0.379888138554016 | 0.484 | 0.444 | 3.85462415931231e-05 | M2 macrophages | MGAT5 |
| 1.24438181819649e-09 | -0.335862266 | 0.434 | 0.447 | 4.48935628550746e-05 | M2 macrophages | PILRA |
| 1.30080481687332e-09 | -0.303883357 | 0.232 | 0.262 | 4.69291353783386e-05 | M2 macrophages | EXOC6 |
| 1.97605606793182e-09 | 0.430384950469936 | 0.366 | 0.331 | 7.12901747627761e-05 | M2 macrophages | ANKS1A |
| 3.56850205891136e-09 | -0.328077762 | 0.511 | 0.512 | 0.000128740848779345 | M2 macrophages | FCHO2 |
| 6.1583952079782e-09 | -0.321160108 | 0.252 | 0.275 | 0.00022217642391823 | M2 macrophages | TOM1 |
| 9.55706343100206e-09 | -0.340507519 | 0.243 | 0.27 | 0.000344790177400261 | M2 macrophages | TIPARP |
| 1.02974607752381e-08 | -0.387236162 | 0.23 | 0.262 | 0.000371501492388266 | M2 macrophages | GLIS3 |
| 1.56722103557662e-08 | 0.329935404843532 | 0.431 | 0.416 | 0.000565406333004976 | M2 macrophages | TREM2 |
| 1.5902735047163e-08 | 0.376739420191438 | 0.267 | 0.233 | 0.000573722972296501 | M2 macrophages | TFRC |
| 1.67592608253487e-08 | 0.313079521946016 | 0.318 | 0.279 | 0.000604623852796105 | M2 macrophages | SH3PXD2A |
| 1.72029801104924e-08 | -0.324109931 | 0.229 | 0.256 | 0.000620631913446234 | M2 macrophages | DLGAP4 |
| 2.68385356638755e-08 | -0.302760775 | 0.233 | 0.259 | 0.000968253851145636 | M2 macrophages | MICU2 |
| 3.60146801501044e-08 | -0.474695259 | 0.395 | 0.405 | 0.00129930161577532 | M2 macrophages | MT2A |
| 3.68726382856824e-08 | -0.324912677 | 0.379 | 0.405 | 0.00133025417143256 | M2 macrophages | MIR99AHG |
| 5.1151821679029e-08 | -0.498922302 | 0.275 | 0.29 | 0.00184540427071433 | M2 macrophages | TBC1D2 |
| 1.04532696607989e-07 | -0.338141523 | 0.298 | 0.314 | 0.00377122609552643 | M2 macrophages | SIL1 |
| 1.59546297748784e-07 | 0.304198107603672 | 0.344 | 0.307 | 0.00575595178388287 | M2 macrophages | AMD1 |
| 1.68949813085412e-07 | 0.431070291273827 | 0.317 | 0.289 | 0.00609520240668241 | M2 macrophages | USP36 |
| 3.36476744915394e-07 | -0.367389171 | 0.277 | 0.296 | 0.0121390715263127 | M2 macrophages | ACSL3 |
| 5.52989714927609e-07 | -0.335296 | 0.398 | 0.404 | 0.0199502099454434 | M2 macrophages | TSPAN15 |
| 6.03882128957863e-07 | -0.321557453 | 0.337 | 0.348 | 0.0217862555664128 | M2 macrophages | SWAP70 |
| 6.44151593617891e-07 | -0.324176829 | 0.252 | 0.271 | 0.0232390570429526 | M2 macrophages | ITPK1 |
| 7.83901023478881e-07 | -0.350103962 | 0.292 | 0.315 | 0.0282807972240476 | M2 macrophages | ENSG00000289901 |
| 1.00539962074601e-06 | -0.359001562 | 0.274 | 0.288 | 0.0362718021176539 | M2 macrophages | IFNGR1 |
| 1.0771846094831e-06 | -0.333407356 | 0.284 | 0.3 | 0.0388615891563216 | M2 macrophages | SLC43A3 |
| 1.15262881706264e-06 | 0.306186082294379 | 0.252 | 0.223 | 0.0415833898331689 | M2 macrophages | ITGA9 |
| 1.60546727125838e-06 | -0.327927984 | 0.351 | 0.359 | 0.0579204427451885 | M2 macrophages | SLC25A24 |
| 2.45291520384318e-06 | 0.366066091307989 | 0.254 | 0.226 | 0.0884938218090505 | M2 macrophages | ELL |
| 1.0586292146871e-05 | 0.318262866686333 | 0.34 | 0.31 | 0.381921661782667 | M2 macrophages | FCGR2C |
| 1.07441970383511e-05 | 0.451168650341191 | 0.251 | 0.226 | 0.387618396552593 | M2 macrophages | NFATC1 |
| 1.63817020990215e-05 | -0.331100976 | 0.235 | 0.252 | 0.591002666626399 | M2 macrophages | CSF3R |

## Supplementary Table S3. Genes with kME＞0.7 derived from hdWGCNA

| gene | module | kME |
| --- | --- | --- |
| RPLP1 | M1 macrophages1 | 0.864845822115756 |
| RPS12 | M1 macrophages1 | 0.858136004794706 |
| TYROBP | M1 macrophages1 | 0.850512939593621 |
| FTH1 | M1 macrophages1 | 0.85023396048819 |
| PFN1 | M1 macrophages1 | 0.840692245364208 |
| RPS23 | M1 macrophages1 | 0.83288161548541 |
| RPL10 | M1 macrophages1 | 0.830359213678001 |
| TMSB10 | M1 macrophages1 | 0.830093621095251 |
| OAZ1 | M1 macrophages1 | 0.823034351195642 |
| RPL30 | M1 macrophages1 | 0.821149493812531 |
| RPL32 | M1 macrophages1 | 0.819911005441848 |
| RPS13 | M1 macrophages1 | 0.819206581865575 |
| RPL19 | M1 macrophages1 | 0.819011230961438 |
| RPL28 | M1 macrophages1 | 0.81817995503579 |
| RPS15A | M1 macrophages1 | 0.816233973459311 |
| RPL26 | M1 macrophages1 | 0.815092108403683 |
| RPS14 | M1 macrophages1 | 0.812935432792429 |
| GAPDH | M1 macrophages1 | 0.812235364803686 |
| RPS8 | M1 macrophages1 | 0.808809866107255 |
| RPL12 | M1 macrophages1 | 0.807489278792976 |
| RPS28 | M1 macrophages1 | 0.805759750081165 |
| RPS27A | M1 macrophages1 | 0.804903884913043 |
| GPX1 | M1 macrophages1 | 0.804594775954863 |
| IFI30 | M1 macrophages1 | 0.80402506323655 |
| MYL6 | M1 macrophages1 | 0.800504079805791 |
| RPL8 | M1 macrophages1 | 0.800122172890146 |
| RPS15 | M1 macrophages1 | 0.794508909342514 |
| RPL18A | M1 macrophages1 | 0.791444092744739 |
| TMSB4X | M1 macrophages1 | 0.78979426124774 |
| SERF2 | M1 macrophages1 | 0.789759716579968 |
| RPS19 | M1 macrophages1 | 0.788842900355125 |
| RPL11 | M1 macrophages1 | 0.78842282594947 |
| GABARAP | M1 macrophages1 | 0.78716117889868 |
| FAU | M1 macrophages1 | 0.785360544487848 |
| RPL13 | M1 macrophages1 | 0.783188294021326 |
| FTL | M1 macrophages1 | 0.78276341148312 |
| RPS3A | M1 macrophages1 | 0.78247460368763 |
| ATP5F1E | M1 macrophages1 | 0.781350765050582 |
| S100A10 | M1 macrophages1 | 0.778055461988423 |
| ACTB | M1 macrophages1 | 0.776387629382978 |
| EEF1A1 | M1 macrophages1 | 0.775692105091496 |
| RPS24 | M1 macrophages1 | 0.775639764973338 |
| RPL29 | M1 macrophages1 | 0.773941910382617 |
| RPL7A | M1 macrophages1 | 0.773599966422336 |
| YBX1 | M1 macrophages1 | 0.773413791936301 |
| RPL34 | M1 macrophages1 | 0.772076227792282 |
| IER3 | M1 macrophages3 | 0.771996091369207 |
| AIF1 | M1 macrophages1 | 0.770364853123449 |
| SH3BGRL3 | M1 macrophages1 | 0.768943864826207 |
| RPS3 | M1 macrophages1 | 0.768366300153755 |
| S100A11 | M1 macrophages1 | 0.767220670536658 |
| RPS27 | M1 macrophages1 | 0.76705762064111 |
| VIM | M1 macrophages1 | 0.762947258965143 |
| RPL15 | M1 macrophages1 | 0.762026830609429 |
| RPS7 | M1 macrophages1 | 0.761070796630746 |
| CD68 | M1 macrophages1 | 0.759676057851229 |
| RPS10 | M1 macrophages1 | 0.759623625442429 |
| RPS25 | M1 macrophages1 | 0.759283536266311 |
| RPS4X | M1 macrophages1 | 0.758736696587664 |
| RBM47 | M1 macrophages2 | 0.758614414592318 |
| CXCL8 | M1 macrophages3 | 0.758308604602166 |
| LGALS1 | M1 macrophages1 | 0.756508454090114 |
| S100A4 | M1 macrophages1 | 0.755510380293296 |
| CST3 | M1 macrophages1 | 0.755509569682935 |
| S100A6 | M1 macrophages1 | 0.755144764996916 |
| CFL1 | M1 macrophages1 | 0.753493049298842 |
| MT-ND4 | M1 macrophages5 | 0.74716797826524 |
| NME2 | M1 macrophages1 | 0.74703039385401 |
| RPL35A | M1 macrophages1 | 0.746849482098679 |
| RPL36 | M1 macrophages1 | 0.745653816385627 |
| RPS18 | M1 macrophages1 | 0.745319263085479 |
| NACA | M1 macrophages1 | 0.743975794998663 |
| TSPO | M1 macrophages1 | 0.740672108454054 |
| RPS9 | M1 macrophages1 | 0.740517937882531 |
| PFDN5 | M1 macrophages1 | 0.73872469488495 |
| CRIP1 | M1 macrophages1 | 0.737996276796683 |
| FNDC3B | M1 macrophages2 | 0.737214325375043 |
| KYNU | M1 macrophages2 | 0.73550990811453 |
| EIF1 | M1 macrophages1 | 0.735339339052769 |
| RPS6 | M1 macrophages1 | 0.733238165725592 |
| ZFAND3 | M1 macrophages2 | 0.733213300345845 |
| VAMP8 | M1 macrophages1 | 0.733203275135439 |
| RPL37 | M1 macrophages1 | 0.733033877770266 |
| RPLP0 | M1 macrophages1 | 0.732070957723952 |
| ACTG1 | M1 macrophages1 | 0.732004968778672 |
| RPL18 | M1 macrophages1 | 0.731074459731538 |
| COX4I1 | M1 macrophages1 | 0.730152057882301 |
| RPL14 | M1 macrophages1 | 0.727463255508998 |
| RPS21 | M1 macrophages1 | 0.726654163679956 |
| RPS5 | M1 macrophages1 | 0.72508196748058 |
| MT-ND3 | M1 macrophages5 | 0.725049140131035 |
| B2M | M1 macrophages1 | 0.7238523129678 |
| PTMA | M1 macrophages1 | 0.722692130668755 |
| JUN | M1 macrophages5 | 0.722109490952493 |
| GSTP1 | M1 macrophages1 | 0.721778148717313 |
| RPL35 | M1 macrophages1 | 0.719244544848349 |
| MT-ND2 | M1 macrophages5 | 0.717575218278764 |
| TPT1 | M1 macrophages1 | 0.715903616166245 |
| RPL39 | M1 macrophages1 | 0.712657421091863 |
| MT-CYB | M1 macrophages5 | 0.712631739870084 |
| RPL6 | M1 macrophages1 | 0.710932624078784 |
| RNASE1 | M1 macrophages1 | 0.70638834382938 |
| RACK1 | M1 macrophages1 | 0.705782284871383 |
| DUSP1 | M1 macrophages5 | 0.705722678413192 |
| RPL3 | M1 macrophages1 | 0.704112602817189 |
| CYBA | M1 macrophages1 | 0.702176582930674 |
| FTH1 | M2 macrophages2 | 0.879246195771397 |
| FTL | M2 macrophages2 | 0.876370167040977 |
| TYROBP | M2 macrophages2 | 0.876203286288515 |
| RPLP1 | M2 macrophages2 | 0.865926106801448 |
| C1QB | M2 macrophages2 | 0.863135324218787 |
| TMSB4X | M2 macrophages2 | 0.86107735187656 |
| PFN1 | M2 macrophages2 | 0.857287079895217 |
| OAZ1 | M2 macrophages2 | 0.854690226222355 |
| TMSB10 | M2 macrophages2 | 0.853727176965753 |
| ACTB | M2 macrophages2 | 0.853675961981129 |
| C1QC | M2 macrophages2 | 0.851286885173799 |
| CD68 | M2 macrophages2 | 0.851029982155316 |
| RPS12 | M2 macrophages2 | 0.847096796510957 |
| GPX1 | M2 macrophages2 | 0.846834278753366 |
| S100A11 | M2 macrophages2 | 0.845852676486218 |
| C1QA | M2 macrophages2 | 0.843924924491628 |
| S100A4 | M2 macrophages2 | 0.843916092120521 |
| S100A6 | M2 macrophages2 | 0.841590723114383 |
| SERF2 | M2 macrophages2 | 0.833738481043975 |
| B2M | M2 macrophages2 | 0.830769723452283 |
| RPL19 | M2 macrophages2 | 0.829502065481846 |
| RPL28 | M2 macrophages2 | 0.826871891479166 |
| RPL30 | M2 macrophages2 | 0.826637818406523 |
| IFI30 | M2 macrophages2 | 0.825817248308257 |
| RNASE1 | M2 macrophages2 | 0.82443828663879 |
| RPS23 | M2 macrophages2 | 0.823789485613312 |
| NPC2 | M2 macrophages2 | 0.822724124560661 |
| S100A10 | M2 macrophages2 | 0.821961830727944 |
| RPS13 | M2 macrophages2 | 0.821447828837221 |
| GABARAP | M2 macrophages2 | 0.81652699354162 |
| CD83 | M2 macrophages3 | 0.811748191049177 |
| RPL10 | M2 macrophages2 | 0.811635763729744 |
| GAPDH | M2 macrophages2 | 0.810686419237994 |
| VIM | M2 macrophages2 | 0.810140533069853 |
| YBX1 | M2 macrophages2 | 0.809957872933857 |
| CFL1 | M2 macrophages2 | 0.808760836725748 |
| AIF1 | M2 macrophages2 | 0.808708246943168 |
| CST3 | M2 macrophages2 | 0.807605383660548 |
| CD74 | M2 macrophages2 | 0.8074939702754 |
| MYL6 | M2 macrophages2 | 0.807352331218929 |
| RPS27A | M2 macrophages2 | 0.806781220007609 |
| RPL26 | M2 macrophages2 | 0.80668444259915 |
| RPL12 | M2 macrophages2 | 0.803063723146771 |
| RPS19 | M2 macrophages2 | 0.802643743765801 |
| RPS15 | M2 macrophages2 | 0.801691663149768 |
| ATP5F1E | M2 macrophages2 | 0.801636118361479 |
| RPL8 | M2 macrophages2 | 0.800895049307325 |
| SH3BGRL3 | M2 macrophages2 | 0.800031347656358 |
| RPS28 | M2 macrophages2 | 0.799277314589364 |
| TSPO | M2 macrophages2 | 0.799080446081134 |
| RPS15A | M2 macrophages2 | 0.799060177732956 |
| FAU | M2 macrophages2 | 0.798870847253439 |
| RPL32 | M2 macrophages2 | 0.797349997644691 |
| RPS8 | M2 macrophages2 | 0.796620596255601 |
| RPL11 | M2 macrophages2 | 0.796132961230729 |
| LGALS1 | M2 macrophages2 | 0.79569921703037 |
| EEF1A1 | M2 macrophages2 | 0.794530245959839 |
| JUN | M2 macrophages3 | 0.792027325092319 |
| CRIP1 | M2 macrophages2 | 0.786307037019539 |
| VAMP8 | M2 macrophages2 | 0.786246776028228 |
| FOSB | M2 macrophages3 | 0.786035903751867 |
| RPS14 | M2 macrophages2 | 0.780447871238294 |
| RPL7A | M2 macrophages2 | 0.780370614786797 |
| RPL15 | M2 macrophages2 | 0.780072081386907 |
| PPIA | M2 macrophages2 | 0.779948653237148 |
| COX4I1 | M2 macrophages2 | 0.773859602460769 |
| RPL29 | M2 macrophages2 | 0.773287592600543 |
| CYBA | M2 macrophages2 | 0.771768283324431 |
| RPS3 | M2 macrophages2 | 0.771673929756438 |
| RPS7 | M2 macrophages2 | 0.771179285239996 |
| CLIC1 | M2 macrophages2 | 0.769676648138631 |
| NME2 | M2 macrophages2 | 0.768310446519078 |
| RPS25 | M2 macrophages2 | 0.767486642643115 |
| RPL18A | M2 macrophages2 | 0.765848143275047 |
| RPS24 | M2 macrophages2 | 0.765624971082494 |
| CSTB | M2 macrophages2 | 0.765014717513153 |
| RNASEK | M2 macrophages2 | 0.763169945013098 |
| RPS18 | M2 macrophages2 | 0.762981944016624 |
| RPL13 | M2 macrophages2 | 0.762271028877284 |
| ACTG1 | M2 macrophages2 | 0.761995441420499 |
| RPS3A | M2 macrophages2 | 0.761590086003965 |
| CTSB | M2 macrophages2 | 0.761508269754054 |
| FCER1G | M2 macrophages2 | 0.761309967491508 |
| ATP6V0C | M2 macrophages2 | 0.758758040416197 |
| EGR1 | M2 macrophages3 | 0.757054065247867 |
| PPDPF | M2 macrophages2 | 0.750851386997262 |
| HLA-DPA1 | M2 macrophages2 | 0.750666371523581 |
| NACA | M2 macrophages2 | 0.75016824271497 |
| RPL34 | M2 macrophages2 | 0.748555767727144 |
| PFDN5 | M2 macrophages2 | 0.748018738496523 |
| EMP3 | M2 macrophages2 | 0.74792667995277 |
| RPL14 | M2 macrophages2 | 0.747719108013562 |
| RPS10 | M2 macrophages2 | 0.74685087686144 |
| RPS4X | M2 macrophages2 | 0.743571800016304 |
| RPS27 | M2 macrophages2 | 0.743249182912003 |
| RPLP0 | M2 macrophages2 | 0.74324597065191 |
| DUSP1 | M2 macrophages3 | 0.740571460700963 |
| LGALS3 | M2 macrophages2 | 0.740005926111054 |
| RPL36 | M2 macrophages2 | 0.739750818609642 |
| HLA-B | M2 macrophages2 | 0.738594535655165 |
| RPL18 | M2 macrophages2 | 0.734727897265581 |
| IER2 | M2 macrophages3 | 0.734094799785479 |
| GNG5 | M2 macrophages2 | 0.733064690656429 |
| ATP6V1F | M2 macrophages2 | 0.729500625820529 |
| RPS5 | M2 macrophages2 | 0.727989467048894 |
| EIF1 | M2 macrophages2 | 0.727653481628635 |
| LRMDA | M2 macrophages1 | 0.72373036547042 |
| JUND | M2 macrophages3 | 0.716446205924814 |
| FOS | M2 macrophages3 | 0.712002100322039 |
| FRMD4B | M2 macrophages1 | 0.711820766917484 |
| IER3 | M2 macrophages3 | 0.709230571163546 |
| ELMO1 | M2 macrophages1 | 0.702288034032136 |
| SEMA4A | M2 macrophages3 | 0.701889453432308 |

## Supplementary Table S4. Shared significantly differential genes across pseudotime state branches

| gene |
| --- |
| FAU |
| RPL15 |
| RPL18A |
| RPS23 |
| RPL39 |
| GPX1 |
| RPL29 |
| RPL19 |
| RPS13 |
| RPS4X |
| SH3BGRL3 |
| RPS15A |
| RPS15 |
| RPL32 |
| UBB |
| UBA52 |
| RPS14 |
| RPL12 |
| RPL18 |
| RPS18 |
| CFL1 |
| RPS21 |
| RPS12 |
| RPL30 |
| RPL8 |
| RPL26 |
| RPL36 |
| RPS27A |
| AIF1 |
| GABARAP |
| RPL35 |
| LGALS1 |
| GAPDH |
| RPS28 |
| RPL7A |
| RPLP2 |
| RPS16 |
| RPL34 |
| RPL35A |
| RPS10 |
| RPL28 |
| RPL14 |
| NACA |
| PFN1 |
| RPS8 |
| RPS3A |
| RPL9 |
| RPS6 |
| ATP5F1E |
| RPL11 |
| RPS3 |
| RPL22 |
| RPS27 |
| TSPO |
| RPS5 |
| ATP6V1F |
| RPS7 |
| NME2 |
| CSTB |
| SRP14 |
| COX4I1 |
| OAZ1 |
| RPL3 |
| RPL24 |
| CLIC1 |
| RPL37 |
| RPL6 |
| RPLP1 |
| SERF2 |
| RPS2 |
| RPL23A |
| RPS19 |
| RPL21 |
| RPL27 |
| RACK1 |
| ATP6V0C |
| CD68 |
| GSTP1 |
| RPS25 |
| VAMP8 |
| YBX1 |
| UBC |
| RPL36A |
| RPS9 |
| TPT1 |
| RPL17 |
| S100A11 |
| RPL4 |
| EIF1 |
| RPL5 |
| FCER1G |
| RPL38 |
| GPX4 |
| PCBP1 |
| LYZ |
| RPS24 |
| BTF3 |
| TMSB10 |
| RPL13 |
| COX6B1 |
| PPIA |
| DUSP1 |
| ELOB |
| RPS26 |
| RPS11 |
| PRDX1 |
| RPL37A |
| PFDN5 |
| TAGLN2 |
| EEF1B2 |
| UQCRB |
| MYL6 |
| COX7C |
| RPL36AL |
| MT-ND4L |
| RPL10A |
| RPL10 |
| NDUFA4 |
| UBL5 |
| RPLP0 |
| FOS |
| NPC2 |
| JUNB |
| MIF |
| C1QC |
| PNRC1 |
| GNG5 |
| COX5B |
| RPL7 |
| MYL12A |
| DBI |
| TXN |
| DYNLL1 |
| HLA-DPA1 |
| SRGN |
| TYROBP |
| H3-3B |
| RPSA |
| PPDPF |
| POLR2L |
| RPL27A |
| PYCARD |
| JUN |
| HLA-B |
| CHCHD2 |
| C1QA |
| RNASE1 |
| RPL13A |
| UQCR11 |
| ATP6V0B |
| BLOC1S1 |
| ATP5MG |
| NDUFB2 |
| RHOB |
| EEF1G |
| CTSD |
| GADD45B |
| EEF2 |
| COX8A |
| ACTG1 |
| SLC25A6 |
| TMA7 |
| H3-3A |
| CD63 |
| TOMM7 |
| COX6A1 |
| OST4 |
| RNASEK |
| COX6C |
| CALM1 |
| RPL23 |
| HSPA8 |
| NDUFA1 |
| S100A9 |
| IFI30 |
| S100A10 |
| SAP18 |
| RPL41 |
| MYL12B |
| FOSB |
| EEF1A1 |
| ATP5MF |
| ATP5MC2 |
| CRIP1 |
| FXYD5 |
| CALM3 |
| PSMA7 |
| HLA-C |
| GNB2 |
| MT-ATP8 |
| UQCRQ |
| HSPB1 |
| NDUFS5 |
| GUK1 |
| SERPINF1 |
| PTMA |
| RPS29 |
| ATP5PD |
| FOLR2 |
| NDUFA13 |
| ZFP36 |
| EEF1D |
| CST3 |
| LAMTOR4 |
| UQCR10 |
| SSR4 |
| C1QB |
| H2AZ1 |
| BBLN |
| NUPR1 |
| LDHA |
| SLC25A5 |
| KRTCAP2 |
| S100A4 |
| MT-ND5 |
| HSP90AB1 |
| NR4A1 |
| BTG1 |
| CAPG |
| GRN |
| SUMO2 |
| NDUFA11 |
| TUBA1B |
| COMMD6 |
| CIB1 |
| S100A8 |
| NFKBIA |
| TIMP1 |
| RPS17 |
| HINT1 |
| RPS20 |
| COX7A2 |
| ATP5MC3 |
| ACP5 |
| TPI1 |
| ATP5PF |
| HLA-A |
| POMP |
| AP2S1 |
| ENSG00000280441 |
| CALR |
| LAPTM5 |
| ATP6V1G1 |
| ANXA5 |
| PEBP1 |
| LGALS3 |
| PPIB |
| EDF1 |
| C4orf48 |
| NPM1 |
| PLTP |
| COX7B |
| C1orf162 |
| SOD1 |
| KLF4 |
| PTMS |
| IER3 |
| HLA-DMA |
| TOMM6 |
| PPP1R15A |
| HIGD2A |
| TREM2 |
| S100A6 |
| TMEM258 |
| TRMT112 |
| NOP10 |
| CYBA |
| WDR83OS |
| ATP5MK |
| SNHG29 |
| YWHAH |
| EGR1 |
| CD14 |
| PRDX5 |
| ARL6IP4 |
| FKBP1A |
| ATP5IF1 |
| BRK1 |
| EMP3 |
| ATP5F1D |
| CD81 |
| TRAPPC5 |
| GSTO1 |
| JUND |
| ATP5PO |
| NDUFB10 |
| IER2 |
| NR4A2 |
| VAT1 |
| ARHGDIB |
| ANAPC11 |
| DNAJB1 |
| AP2M1 |
| CD83 |
| HSBP1 |
| SYNGR2 |
| NDUFB7 |
| NDUFC2 |
| TUBB |
| SEC61B |
| APOC1 |
| CHCHD10 |
| ALDOA |
| NEDD8 |
| PPP1CA |
| SOX5 |
| COX5A |
| ARPC5 |
| SERP1 |
| FCGR3A |
| RPL31 |
| HLA-E |
| TMSB4X |
| SLC25A3 |
| GLUL |
| IFI27L2 |
| PSME1 |
| ALOX5AP |
| LAMTOR1 |
| CSNK2B |
| RAC1 |
| CD59 |
| SNRPD2 |
| NDUFB11 |
| HLA-DRA |
| HLA-DRB5 |
| MICOS10 |
| RPS27L |
| ENO1 |
| YWHAB |
| CEBPB |
| RNH1 |
| ISCU |
| PRR13 |
| SARAF |
| ATP5MJ |
| TALDO1 |
| SELENOW |
| EIF3K |
| ATF3 |
| ACTB |
| NDUFB4 |
| TMBIM6 |
| C4orf3 |
| ANXA1 |
| BSG |
| C19orf53 |
| UQCRH |
| MAP1LC3B |
| ATP5F1B |
| CXCL2 |
| APRT |
| KLF2 |
| HLA-DPB1 |
| CD99 |
| TMEM219 |
| NOP53 |
| GNAS |
| ARHGDIA |
| HSPE1 |
| ARL6IP1 |
| FKBP8 |
| EIF4A1 |
| TRAPPC1 |
| CD151 |
| RABAC1 |
| SEC61G |
| RNF181 |
| DAZAP2 |
| HLA-DQB1 |
| RAN |
| RHOG |
| HLA-DRB1 |
| GCHFR |
| NENF |
| PRNP |
| POLR2J3 |
| CCL3 |
| CD37 |
| ROMO1 |
| ITGB2 |
| ATP5ME |
| SNX3 |
| KCTD12 |
| PKM |
| ARPC3 |
| DDAH2 |
| LMNA |
| FTH1 |
| TRIR |
| RAB13 |
| FIS1 |
| DPP7 |
| BRI3 |
| LAMTOR2 |
| CCL4 |
| MIR99AHG |
| UBXN1 |
| DHRS7 |
| APOE |
| SDCBP |
| HSPA1A |
| SF3B5 |
| NINJ1 |
| CFD |
| CALM2 |
| SELENOH |
| EID1 |
| SPCS1 |
| FBP1 |
| UCP2 |
| BCAP31 |
| CHMP2A |
| LAMTOR5 |
| MGST3 |
| SKP1 |
| DUSP23 |
| C2 |
| PRG4 |
| PSMB6 |
| DSTN |
| DISC1 |
| TNRC6A |
| ITM2B |
| PARK7 |
| RHOA |
| SELENOK |
| EIF3G |
| MT-ND2 |
| AFF1 |
| GMFG |
| GRK3 |
| PSMB3 |
| DNAAF9 |
| CXCL3 |
| JTB |
| TMEM14C |
| ALDH1A1 |
| ATP6V0E1 |
| CTNND1 |
| WDFY3 |
| TXNDC17 |
| TMEM147 |
| SMDT1 |
| ARPC1B |
| MIR3667HG |
| DRAP1 |
| COPE |
| CLU |
| VIM |
| IFI27 |
| PLCL2 |
| SCAND1 |
| COTL1 |
| HMGN2 |
| VPS13B |
| CRTAP |
| KLF6 |
| RREB1 |
| AKR1B1 |
| HMGB1 |
| CXCL16 |
| TSC22D3 |
| RBM6 |
| PCBP2 |
| ZNF710 |
| FLNA |
| PLCG2 |
| FABP3 |
| TTC17 |
| SUMO3 |
| NDUFAF3 |
| CIAO2B |
| TLE5 |
| AURKAIP1 |
| COPS9 |
| PELATON |
| TBC1D22A |
| PSMB1 |
| VPS28 |
| ARF6 |
| FABP5 |
| TNRC18 |
| GARS1-DT |
| COMT |
| CDKN1A |
| DAD1 |
| GABARAPL2 |
| EIF5A |
| RBM3 |
| ERH |
| KDELR1 |
| NKTR |
| GMDS-DT |
| HLA-DQA1 |
| TBC1D14 |
| ATRAID |
| GRINA |
| APH1A |
| NDUFA2 |
| ZYX |
| COX14 |
| XIST |
| ARF1 |
| TREM1 |
| GNAI2 |
| PSD3 |
| RNF7 |
| POLR2J |
| NRP1 |
| DIP2B |
| PAN3 |
| PRDX6 |
| NDUFB8 |
| TET3 |
| CAPNS1 |
| TOMM20 |
| CNBP |
| ASAH1 |
| STK3 |
| RAB5C |
| MRPL57 |
| LAMP1 |
| WDR70 |
| TMEM50A |
| GLRX |
| DYNLRB1 |
| FLI1 |
| PDE4D |
| PRELID1 |
| HLA-DQA2 |
| MON2 |
| NDUFS7 |
| RBX1 |
| MED13L |
| SNRPG |
| MRPL41 |
| B2M |
| DNAJA1 |
| STAG1 |
| HERC4 |
| DNMT3A |
| CCNI |
| SQSTM1 |
| PPT1 |
| CXCL8 |
| TRAPPC9 |
| MAFB |
| TWF2 |
| ATP8B4 |
| REX1BD |
| PLAUR |
| ATP6AP2 |
| INPP4A |
| HNRNPK |
| CTSL |
| ST13 |
| TMEM59 |
| NDUFA3 |
| TMCC1 |
| MRPL51 |
| ARF5 |
| BST2 |
| ARID1B |
| FTL |
| CREG1 |
| LST1 |
| PABPC1 |
| RBM26 |
| PET100 |
| FTX |
| SEM1 |
| RHOC |
| AVL9 |
| KDM2A |
| NDUFB9 |
| LAPTM4A |
| GSAP |
| FCHSD2 |
| KRT10 |
| NDUFA6 |
| CDKAL1 |
| OLR1 |
| HNRNPA1 |
| RPS19BP1 |
| AGO4 |
| GPSM3 |
| DOK2 |
| NAMPT |
| ETV6 |
| POLR2E |
| PSMD8 |
| PHC3 |
| DENND1A |
| PHPT1 |
| CELF1 |
| RTRAF |
| TKT |
| NDUFS6 |
| MGAT1 |
| AKAP10 |
| SPIDR |
| PGLS |
| SFMBT2 |
| VKORC1 |
| GPATCH8 |
| ZFAS1 |
| KANSL1 |
| CYCS |
| H2AJ |
| MIEN1 |
| GTF3A |
| CIAO2A |
| NLK |
| MICOS13 |
| HMOX1 |
| MT-ND6 |
| ZFAND5 |
| TENT2 |
| PLIN2 |
| UXT |
| JAK2 |
| SGK1 |
| NCOA1 |
| HEXB |
| PTPN6 |
| LSM4 |
| MT-ND3 |
| ZFP36L2 |
| PARP8 |
| EHMT1 |
| AAK1 |
| SNHG6 |
| ENY2 |
| TMEM176A |
| PRKAG2 |
| CDC42 |
| MT-ND1 |
| SOS1 |
| NUCB1 |
| FAF1 |
| DEFB1 |
| BIRC6 |
| CLASP1 |
| TMEM160 |
| SLC39A11 |
| GPNMB |
| H2AZ2 |
| NAV2 |
| ANKS1A |
| ATXN7L1 |
| TPM3 |
| P4HB |
| EPS15 |
| NDUFC1 |
| VDAC1 |
| TOMM5 |
| RGCC |
| ZNF706 |
| ZNHIT1 |
| EIF3F |
| ERP29 |
| IGSF6 |
| SNU13 |
| CTSA |
| HERC1 |
| RBP4 |
| BANF1 |
| GLIPR1 |
| PSMB10 |
| NAA38 |
| MGAT5 |
| CNPY3 |
| ATP5PB |
| PNISR |
| ATP9B |
| UQCRC1 |
| TNFAIP3 |
| ATRN |
| SUB1 |
| ANXA2 |
| UFC1 |
| COG5 |
| IFITM2 |
| ENSG00000278996 |
| RASA1 |
| NAALADL2 |
| SCFD2 |
| CUTA |
| EIF4G2 |
| ATM |
| ITPR2 |
| PIP4K2A |
| ZNF638 |
| MS4A4E |
| MRPL34 |
| OSBPL3 |
| NSD1 |
| ASH1L |
| ZNF609 |
| GADD45GIP1 |
| LINC03009 |
| FAM172A |
| PHKB |
| C18orf32 |
| RGL1 |
| SLC9A9 |
| EEF2K |
| AUP1 |
| TUBB4B |
| RNF150 |
| ATR |
| ARMH3 |
| PGAM1 |
| SLCO3A1 |
| ITSN1 |
| MACF1 |
| FARS2 |
| VAV3 |
| SNRPB |
| VPS8 |
| ATP6V0D1 |
| ARL6IP5 |
| COP1 |
| CEBPD |
| C5AR1 |
| DIAPH2 |
| FUCA1 |
| RNASET2 |
| RABGAP1 |
| PCCA |
| ATOX1 |
| AOPEP |
| BLTP1 |
| ATXN2 |
| RAD23A |
| NDUFB3 |
| ARL15 |
| ZCCHC7 |
| IFITM3 |
| SIVA1 |
| CCL4L2 |
| SOD2 |
| GNG10 |
| PRDX3 |
| LRRK1 |
| ENSG00000226149 |
| BAX |
| AKAP9 |
| VTI1A |
| CTSZ |
| SRP9 |
| PPP6R2 |
| JPX |
| NCOA2 |
| HIVEP3 |
| PEA15 |
| FAM193A |
| TSPAN4 |
| TMEM179B |
| ALDH2 |
| PIBF1 |
| SNED1 |
| IKBKB |
| GHITM |
| MRPL54 |
| RGS10 |
| TFCP2 |
| FTO |
| MT-ND4 |
| CDK13 |
| HDAC8 |
| UBR3 |
| NF1 |
| NT5C2 |
| SMURF2 |
| BTG2 |
| INPP5D |
| INSIG1 |
| TPM4 |
| RAB5IF |
| FAM120B |
| COX17 |
| BTBD9 |
| POLD4 |
| TIMM8B |
| SIPA1L3 |
| PHF21A |
| C2CD5 |
| FBXO42 |
| BAZ2B |
| NDUFAB1 |
| CISD3 |
| SERPINB1 |
| HLA-DRB6 |
| SPI1 |
| MLLT10 |
| MARK3 |
| MBD5 |
| CLEC2B |
| PLAAT3 |
| DDT |
| TPK1 |
| MRPS21 |
| ABL1 |
| PLBD1 |
| PKN2 |
| NBAS |
| PDSS2 |
| R3HDM2 |
| SSH2 |
| SLC16A3 |
| FCGR2C |
| DOCK1 |
| CTSH |
| MYADM |
| RBM8A |
| ADIPOR1 |
| RPS6KC1 |
| MPC2 |
| GIGYF2 |
| CHN2 |
| PLD3 |
| TNRC6B |
| TMED9 |
| CHD9 |
| HELZ |
| RELCH |
| BAG1 |
| EXOC6B |
| DIRC3 |
| STUB1 |
| KDM6B |
| PSAP |
| SSR2 |
| MIR29B2CHG |
| SETD2 |
| TNFSF13 |
| ZNF407 |
| SERTAD1 |
| SF3B6 |
| CPEB3 |
| ANKRD17 |
| ARID1A |
| LRBA |
| FNBP4 |
| CBL |
| ATP11A |
| ITCH |
| ARHGAP25 |
| ANKRD44 |
| SENP6 |
| UBE3C |
| WWP1 |
| PDS5A |
| PRKN |
| KDM7A |
| MIR34AHG |
| NEU1 |
| SHPRH |
| BBS9 |
| ENTPD1 |
| CRTAC1 |
| LINGO1 |
| TMEM256 |
| MIRLET7BHG |
| ATAD2B |
| ARFGEF1 |
| NDUFA12 |
| EIF1B |
| TRIO |
| FER |
| SNX29 |
| SDF4 |
| VPS13D |
| DOCK11 |
| MT-CYB |
| ST6GAL1 |
| MLF2 |
| PUM2 |
| INVS |
| SCAPER |
| ALPK1 |
| IMMP2L |
| ATP5MC1 |
| KDM4C |
| TCN2 |
| FRMD4A |
| RERE |
| VPS54 |
| DMXL2 |
| CCDC18-AS1 |
| RUFY3 |
| CD74 |
| ATP5F1A |
| DNASE2 |
| ADRM1 |
| ARHGAP22 |
| WWOX |
| PSMC5 |
| ZZZ3 |
| WDPCP |
| RICTOR |
| SLC25A13 |
| PTPRJ |
| SCFD1 |
| ABCC1 |
| ALKBH7 |
| FHIT |
| TNXB |
| MOB3B |
| TBCB |
| USP34 |
| DLG1 |
| NR4A3 |
| TMED2 |
| L3MBTL4 |
| SUMF1 |
| ARHGAP31 |
| PHIP |
| SNX13 |
| EIF4EBP1 |
| BNC2 |
| LSM3 |
| CLASP2 |
| CTBP2 |
| SPG11 |
| TNF |
| CORO1B |
| CTSS |
| GPATCH2 |
| SCN9A |
| FRYL |
| PDGFC |
| MZT2B |
| MDH2 |
| ZNF767P |
| BCAS3 |
| DYM |
| HERC3 |
| HECTD4 |
| MYO9A |
| TIMP3 |
| IL1B |
| PARP14 |
| VPS50 |
| DOCK8 |
| METTL26 |
| LSM7 |
| ZNF148 |
| TBC1D5 |
| SLC36A1 |
| HERC2 |
| SMIM26 |
| SMYD3 |
| ARL2 |
| TCF4 |
| MARCHF1 |
| SLC38A9 |
| MKLN1 |
| KAT6A |
| FNBP1 |
| ARID2 |
| TUFM |
| GAB2 |
| STARD13 |
| KIAA0319L |
| N4BP2L2 |
| CEP192 |
| SLC38A6 |
| MTLN |
| CSNK1G1 |
| CMIP |
| VPS53 |
| RPTOR |
| MAP3K1 |
| KIF13A |
| ZNF292 |
| SETD5 |
| EBF1 |
| ABRACL |
| SCAF8 |
| RBM33 |
| STIM1 |
| ERC1 |
| PSME2 |
| SPRED1 |
| DYRK1A |
| AHCYL2 |
| FAM168A |
| HCFC1R1 |
| ENSG00000290928 |
| UQCRFS1 |
| USP25 |
| KDELR2 |
| MDFIC |
| DMXL1 |
| STAU2 |
| WDFY2 |
| GTF2A2 |
| DISP1 |
| LYST |
| THADA |
| FAM13A |
| RNF5 |
| FUOM |
| PHB2 |
| TMEM117 |
| ATF7 |
| KDM2B |
| SNX17 |
| MYCBP2 |
| ENSG00000291015 |
| UBR2 |
| MVB12B |
| WDFY4 |
| TTC28 |
| USP24 |
| RASA2 |
| CITED2 |
| SSNA1 |
| SH3PXD2A |
| CWF19L2 |
| CLEC16A |
| DOP1B |
| MEA1 |
| SGMS1 |
| ATXN1 |
| ANKRD36C |
| LRCH3 |
| AP3B1 |
| DCTN3 |
| HECTD2 |
| FRS2 |
| RNASE6 |
| RTN1 |
| RFX7 |
| ADCY7 |
| RNF213 |
| WDR7 |
| SETX |
| MYO1F |
| KDM6A |
| ITGA9 |
| ESR1 |
| ANKIB1 |
| ARRB1 |
| FBXL20 |
| PBRM1 |
| HDAC9 |
| METTL15 |
| AMBRA1 |
| LRCH1 |
| DGLUCY |
| SPECC1L |
| RNF38 |
| ENTPD1-AS1 |
| TCF12 |
| WWP2 |
| BRWD1 |
| PHACTR2 |
| ZRANB2 |
| ASXL2 |
| GTDC1 |
| DIS3L2 |
| COMMD10 |
| FAM133B |
| KIAA2026 |
| RALGAPA1 |
| RAD51B |
| CBR1 |
| RASAL2 |
| PPP3CA |
| NIPBL |
| VPS13C |
| ZBTB20 |
| CDYL |
| ASCC3 |
| UTRN |
| ENSG00000283674 |
| UGGT2 |
| NCOA3 |
| NFIA |
| CPNE8 |
| FOXJ3 |
| RABGAP1L |
| TMEM131 |
| RNF216 |
| MFSD14CP |
| CCNY |
| UVRAG |
| PHB1 |
| SUPT4H1 |
| FBXL17 |
| MRTFA |
| USP32 |
| SRGAP2 |
| PTPN12 |
| MRPS34 |
| ATXN7 |
| ITFG1 |
| C1QBP |
| CCDC124 |
| PRR14L |
| ROCK2 |
| AGAP1 |
| THRB |
| TRPS1 |
| CNTLN |
| GPR183 |
| SOS2 |
| ZNF280D |
| TRPC4AP |
| LUC7L3 |
| UBE4B |
| ZMYM4 |
| RGS1 |
| ANKRD36 |
| HRH1 |
| ATG7 |
| GCNT1 |
| ECHS1 |
| NEMF |
| DTNA |
| PTBP2 |
| CAMKMT |
| REV1 |
| TBL1XR1 |
| SRPK2 |
| CTSB |
| FCGRT |
| MDM4 |
| SLC2A9 |
| TBCK |
| PHF14 |
| ASAP1 |
| ARHGAP15 |
| FCHO2 |
| PLD1 |
| CCL3L3 |
| PTK2 |
| RIN2 |
| HDAC4 |
| SLC4A7 |
| XRN1 |
| MAML3 |
| DOCK2 |
| FAM135A |
| TNKS |
| RFX3-DT |
| ERCC6L2 |
| APPL2 |
| NPIPB13 |
| DDX17 |
| DNAJC5 |
| DOP1A |
| TASOR2 |
| PTEN |
| SBF2 |
| RNF111 |
| TANGO6 |
| ZZEF1 |
| MAP3K14 |
| DBNDD2 |
| SDCCAG8 |
| RC3H1 |
| CEP170 |
| NHP2 |
| HMBOX1 |
| DAPK1 |
| ARFGEF2 |
| NFATC3 |
| CENPX |
| BMPR2 |
| NSMCE2 |
| GET3 |
| ULK4 |
| APC |
| EIF4G3 |
| RNPC3 |
| CARMIL1 |
| RUBCNL |
| NEK7 |
| ABHD18 |
| TRPM7 |
| ANKFY1 |
| DIP2A |
| AKT3 |
| SLC8A1 |
| ENSG00000291203 |
| EXOC4 |
| TBC1D2B |
| TSHZ3 |
| SUSD6 |
| LINC02785 |
| FAM13B |
| UBE2K |
| PIAS1 |
| TTC14 |
| ASAP2 |
| ARPC2 |
| R3HDM1 |
| POLK |
| EYA2 |
| TIMM23B |
| FKBP2 |
| AGFG1 |
| CAB39 |
| DLEU2 |
| POLR2K |
| ZNF33A |
| PHACTR4 |
| SENP7 |
| CDK2AP2 |
| KMT2C |
| TDRD3 |
| MAPK14 |
| ARHGEF10L |
| KLHDC10 |
| PCBD1 |
| CARD16 |
| LINC00342 |
| ASPH |
| CUL5 |
| TUBGCP3 |
| TRAPPC10 |
| TRIM33 |
| PIKFYVE |
| TBC1D12 |
| GANC |
| ENSG00000262879 |
| ERICH1 |
| EPC1 |
| C17orf49 |
| SLC23A2 |
| GPBP1L1 |
| DOCK7 |
| VPS45 |
| BMP2K |
| MAML2 |
| ENSG00000257398 |
| PEAK1 |
| SV2B |
| APPBP2 |
| MT-CO2 |
| TEC |
| PAM |
| H4C3 |
| LCORL |
| FNIP1 |
| ZNF518A |
| ATF7IP |
| PCED1B |
| OPHN1 |
| RALGAPA2 |
| SUFU |
| BICD1 |
| RAPGEF1 |
| EPC2 |
| VAV1 |
| SIPA1L2 |
| MAPKAP1 |
| TTC7B |
| CBLB |
| SMIM29 |
| OGA |
| SLC16A1-AS1 |
| CERS6 |
| ARHGAP24 |
| RAP1GDS1 |
| YTHDC2 |
| RNF217 |
| ELMO1 |
| CMKLR1 |
| NCOA6 |
| SUCLG2 |
| DNPH1 |
| SMG6 |
| ZMYND8 |
| ZDHHC21 |
| MTAP |
| DGKH |
| CUX1 |
| CHFR |
| TTBK2 |
| DENND1B |
| USP33 |
| ENSG00000253496 |
| ATRX |
| MAMDC2 |
| GATAD2B |
| NAV1 |
| ENSG00000291136 |
| NUTM2A-AS1 |
| UBR1 |
| HGSNAT |
| HEATR5A |
| MERTK |
| ZNRF2 |
| POU2F1 |
| PPP1R21 |
| NFX1 |
| EHBP1 |
| MBOAT1 |
| AUTS2 |
| FRY |
| NFATC2 |
| ENSG00000273748 |
| SPIRE1 |
| SNX14 |
| HTT |
| HECTD1 |
| CIITA |
| TRAK1 |
| DTNB |
| ZNF385B |
| LIMD1 |
| CCM2 |
| FAM222B |
| CEPT1 |
| PCNX2 |
| ITSN2 |
| IKZF1 |
| PPP3CC |
| MANBA |
| FMN1 |
| BCL2A1 |
| CBFA2T2 |
| LRRC8D |
| BDP1 |
| FBXW4 |
| PACS1 |
| GLG1 |
| RUNX1 |
| SUSD1 |
| ENSG00000284977 |
| PTPRM |
| APBA1 |
| SPG7 |
| FAM53B |
| TMEM135 |
| DPH6 |
| PI4KA |
| RHOT1 |
| MARCHF7 |
| PPP3CB |
| GOLGA4 |
| LARP4B |
| BLNK |
| RBM25 |
| MAP2K5 |
| NPEPPS |
| PCM1 |
| PLEKHA2 |
| PREX1 |
| SRSF11 |
| RBMS1 |
| OXR1 |
| WDR37 |
| ZNF438 |
| ZFHX3 |
| YEATS2 |
| ST8SIA4 |
| BTRC |
| CUEDC1 |
| CEP350 |
| ARHGAP21 |
| KLC1 |
| FAR1 |
| TMEM106A |
| TLK2 |
| CTIF |
| MGAT4A |
| GTF2I |
| SVIL-AS1 |
| DENND4C |
| PHYKPL |
| DPYD |
| ARHGEF11 |
| WIPF1 |
| LATS1 |
| PARP4 |
| ARAP2 |
| MFSD4B |
| WDR20 |
| NCOR1 |
| PIK3CB |
| MTHFD1L |
| ICA1 |
| FAR2 |
| XPO4 |
| EXOC6 |
| SLCO2B1 |
| ANKHD1 |
| EXOC2 |
| TAOK1 |
| POU2F2 |
| RNGTT |
| ZDHHC14 |
| CLEC2D |
| LDAH |
| TNS3 |
| LDLRAD4 |
| MEF2C |
| HEATR5B |
| SLC22A23 |
| CCDC91 |
| ACACA |
| BABAM2 |
| FOXP1 |
| ZKSCAN1 |
| NRF1 |
| MYO5A |
| ABR |
| SH3RF3 |
| GRAMD1B |
| RSRC1 |
| MICU2 |
| ARHGAP10 |
| DLC1 |
| NUP160 |
| ANKAR |
| AP2A2 |
| PIP5K1A |
| FIG4 |
| COL1A2 |
| SLF2 |
| MCPH1 |
| TPCN1 |
| AP3S2 |
| KAZN |
| BPTF |
| PIK3C3 |
| MSH3 |
| NEK6 |
| COL3A1 |
| ANKRD11 |
| HLCS |
| ZNF532 |
| PAXBP1 |
| CCDC14 |
| CCDC66 |
| PVT1 |
| WDR11 |
| CDK8 |
| EYA3 |
| GUSBP1 |
| CPLANE1 |
| MYO1D |
| RCOR3 |
| PCNX1 |
| LINC02328 |
| VMP1 |
| EVI5 |
| UBE2E2 |
| ELF2 |
| SMAD4 |
| FOXK1 |
| RNF169 |
| DMTF1 |
| DOCK10 |
| LPP |
| ZFAND3 |
| FOXN3 |
| GAS7 |
| CBR4 |
| ENSG00000290993 |
| PPARGC1B |
| COX10-DT |
| GAB3 |
| MCM9 |
| TTN |
| ENSG00000227486 |
| SGK3 |
| PARD3B |
| ENSG00000228434 |
| HMGA1P4 |
| UBN2 |
| MINDY3 |
| ENSG00000290921 |
| ARHGAP6 |
| CHST11 |
| PIGL |
| DNM1 |
| NR3C1 |
| TMEM260 |
| ARMCX4 |
| CAMK1D |
| SCMH1 |
| XKR6 |
| ENSG00000287100 |
| FAM118A |
| ST3GAL3 |
| SP100 |
| JAZF1 |
| SLC7A6 |
| FBXO28 |
| HYCC2 |
| ST3GAL1 |
| PBX3 |
| SNHG14 |
| RPAP2 |
| CPQ |
| NUDT16-DT |
| NUDT3 |
| ENSG00000267787 |
| ZNF75D |
| TTC7A |
| TFEC |
| ADAP2 |
| MEF2A |
| INPP5B |
| ZMIZ1 |
| RIN3 |
| SCAF11 |
| PCGF3-AS1 |
| XRRA1 |
| NCOR2 |
| JMJD1C |
| ITGB8 |
| BMS1P1 |
| NHLRC2 |
| LINC01684 |
| SYK |
| LINC00486 |
| SND1 |
| NAA25 |
| STX7 |
| ENSG00000291175 |
| MTSS1 |
| TTC13 |
| SORCS2 |
| TMEM168 |
| CCDC7 |
| ATP8B1 |
| COL6A2 |
| RAB39A |
| USP15 |
| ME1 |
| MT-ATP6 |
| PIK3R5 |
| MYO9B |
| SDHAP4 |
| SNX24 |
| MT-CO1 |
| MAGI2 |
| ANKRD12 |
| LRMDA |
| PICALM |
| FNDC3B |
| APBB2 |
| ZSWIM6 |
| ELMOD3 |
| TANC2 |
| FRMD4B |
| MT-CO3 |
| SIGLEC1 |
| LRP1B |
| GNAQ |
| RB1 |
| C8orf44 |
| DOCK4 |
| ABI3BP |
| CYTH1 |
| ENSG00000271533 |
| DST |
| CELF2 |
| TBXAS1 |
| PTPRC |
| LINC00923 |
| HEXIM2-AS1 |
| COLEC12 |
| LAMTOR5-AS1 |
| RBM47 |
| ENSG00000265987 |
| OVCH1-AS1 |
| ACAP2 |
| ENSG00000289474 |
| EFNA5 |
| FMNL2 |
| SEMA3A |
| RBPJ |
| CYRIB |
| APBB1IP |
| ARHGAP18 |
| ZEB2 |
| NEAT1 |
| TNS1 |
| PLXDC2 |
| FYB1 |
| DAB2 |
| MALAT1 |
| UIMC1 |
| MRPS15 |
| PSMA2 |
| PABPC4 |
| F13A1 |
| VOPP1 |
| SYNRG |
| ELOC |
| PLP2 |
| PGK1 |
| RAB3GAP2 |
| ABCC5 |
| ZFR |
| PDCD5 |
| NSUN3 |
| UHRF2 |
| MRPL52 |
| SPIN1 |
| TOGARAM1 |
| EVI2B |
| SENP5 |
| FUNDC2 |
| MAP3K5 |
| DHX57 |
| LINC01374 |
| KDM5A |
| MAF1 |
| NOL10 |
| LARGE1 |
| TRMT11 |
| KIF16B |
| CTSK |
| DHRSX |
| HNRNPA0 |
| MRPL43 |
| PPP2R5E |
| MAP4 |
| PUM1 |
| DCAF5 |
| EPB41L1 |
| UPF2 |
| TSHZ2 |
| SLC2A13 |
| PDS5B |
| PSENEN |
| EZR |
| REPS1 |
| SSH1 |
| GRK5 |
| ENSG00000258168 |
| ACVR2A |
| SLMAP |
| RALGAPB |
| IL15 |
| DNAJC13 |
| USP47 |
| UBE2Q2P1 |
| LRRC8C |
| NTAN1 |
| PRPF4B |
| TMLHE |
| TOM1L2 |
| BICRAL |
| CREBBP |
| GTF3C6 |
| ROCK1 |
| ADARB1 |
| PIK3AP1 |
| TOMM22 |
| PIGN |
| KCNQ3 |
| VDAC2 |
| MARK2 |
| TMEM161B-DT |
| RIC1 |
| SMURF1 |
| RWDD1 |
| DNM2 |
| ARPC4 |
| PUDP |
| CARD8 |
| COA3 |
| PPP1R12B |
| SPATA5 |
| R3HCC1L |
| PIK3C2A |
| TMEM230 |
| KMT5B |
| SMC5 |
| ANK2 |
| DCAF10 |
| SLX4IP |
| SEC24B |
| PSMA3-AS1 |
| MRPS24 |
| VPS41 |
| FGD5 |
| NDUFS8 |
| UBE2W |
| FBXL4 |
| FBXW7 |
| UBR5 |
| STX8 |
| ACOT7 |
| RPN1 |
| PKD2 |
| VAMP3 |
| SEMA4A |
| ATE1 |
| IKZF2 |
| USP6NL |
| GLMP |
| ATXN3 |
| FBXW8 |
| SEC22A |
| LSM10 |
| DDX10 |
| ZNF91 |
| WWC2 |
| PIGX |
| FGF13 |
| MBTD1 |
| CDC42BPA |
| NDUFB1 |
| EPSTI1 |
| CYC1 |
| EPS8 |
| CLYBL |
| BCLAF3 |
| SSR3 |
| CTDSPL2 |
| FOXK2 |
| ZNF611 |
| NLN |
| MRFAP1 |
| SLC10A7 |
| EDA |
| ZNF586 |
| RPS4Y1 |
| SEMA3C |
| TRIP4 |
| CCDC88A |
| SDHAP1 |
| SFMBT1 |
| XAF1 |
| VWA8 |
| ENSG00000274422 |
| ACYP2 |
| FAAP20 |
| DDX60L |
| RPRD1A |
| DUSP2 |
| GASK1B-AS1 |
| ZXDC |
| CRTC3 |
| SELENOP |
| SLC20A2 |
| SRSF3 |
| ITLN1 |
| USP40 |
| CCNT2 |
| PSMB8 |
| ZNF721 |
| CDC42SE2 |
| RC3H2 |
| ADI1 |
| MPP1 |
| IQCB1 |
| ENSG00000259972 |
| FGD2 |
| ZNF385A |
| LINC01278 |
| LCOR |
| ERGIC3 |
| RBMS3 |
| MIATNB |
| REEP5 |
| SLC25A39 |
| SLC8A1-AS1 |
| ANKRD26 |
| TCEAL4 |
| AOAH |
| ZNF431 |
| ABLIM3 |
| NHSL1 |
| BTBD7 |
| HOXB6 |
| POLR2I |
| CDH23 |
| ATXN10 |
| IBTK |
| CENATAC |
| TRAPPC12 |
| AGO3 |
| SREBF2 |
| RAB28 |
| MLXIP |
| UBE2L6 |
| LINC01504 |
| ATP1A1 |
| SMG1 |
| LYN |
| FUT8 |
| IMP3 |
| RAB1B |
| VMA21 |
| TIMM13 |
| SPATA6 |
| HIP1 |
| CCDC85B |
| FAM174C |
| SNX30 |
| MNAT1 |
| NSA2 |
| CLTB |
| NOL7 |
| ZDHHC17 |
| CHD7 |
| RAB3GAP1 |
| WASHC4 |
| SP3 |
| TUBA1C |
| RANBP1 |
| TMEM35B |
| KMT2A |
| EMC4 |
| KLHL5 |
| MIR181A1HG |
| SDF2L1 |
| DIDO1 |
| SCLT1 |
| GIT2 |
| GAPVD1 |
| TCERG1 |
| GSE1 |
| PTPRA |
| DENND4B |
| SURF1 |
| PRIM2 |
| TIMM10 |
| CEP162 |
| CD9 |
| GMDS |
| BUD31 |
| NUBPL |
| PPP1R12A |
| CSRNP1 |
| PPP6R3 |
| THOC1 |
| JOSD2 |
| STAT5B |
| RPRD2 |
| PTPN9 |
| ID2 |
| RPL22L1 |
| SPPL3 |
| ZNF780B |
| NCKAP5 |
| TYW1 |
| LINC01473 |
| EGR2 |
| SETDB1 |
| TAX1BP3 |
| FIBP |
| SUMO1 |
| CSGALNACT1 |
| GON4L |
| EIF4EBP3 |
| EEFSEC |
| SBDS |
| ENSG00000234147 |
| NAXE |
| PFKFB3 |
| KSR1 |
| ARID4B |
| PTPMT1 |
| ECM1 |
| TTLL5 |
| GALNT10 |
| RAB11B |
| RYR1 |
| ARHGEF7 |
| ARGLU1 |
| DENND6A |
| EMD |
| RBIS |
| N4BP1 |
| STARD9 |
| KDM3B |
| KIDINS220 |
| GPD2 |
| RUFY2 |
| SUGP2 |
| MED27 |
| COL5A2 |
| IRF1-AS1 |
| RAPGEF6 |
| ZNF169 |
| ARFIP1 |
| ENSG00000284685 |
| LINC02649 |
| GPR107 |
| EXOC1 |
| DGCR6L |
| DYNC2I1 |
| SPTLC2 |
| GARRE1 |
| STRADA |
| PLEKHM3 |
| ACBD6 |
| RPS6KA2 |
| IREB2 |
| KATNBL1 |
| ZEB1 |
| NCEH1 |
| SLTM |
| SKAP2 |
| RNASEH2C |
| NCL |
| PCNX4 |
| MRTFB |
| RHEB |
| EEIG2 |
| RAB10 |
| NDUFA7 |
| TRA2A |
| RPS6KA3 |
| GCNT2 |
| TUT4 |
| AIP |
| ZFC3H1 |
| SYNE3 |
| ZMYM6 |
| CCSER2 |
| EIF4A3 |
| TRAPPC2L |
| IER5 |
| UQCC1 |
| HLA-DMB |
| PRPF18 |
| COA1 |
| KLHL20 |
| RBM5 |
| THOC2 |
| TUBA1A |
| BACH1 |
| TMEM176B |
| PPP4C |
| CLOCK |
| NUTM2B-AS1 |
| TRRAP |
| MRPL18 |
| ADGRB3 |
| ABCA6 |
| MKNK1 |
| PSMB4 |
| FAM228B |
| ABCC4 |
| HIBCH |
| TAF2 |
| ZNF708 |
| ARHGAP26 |
| CNTRL |
| NPIPB5 |
| MTREX |
| FAM50A |
| AGAP6 |
| SRI |
| PRRC2C |
| WDR33 |
| TAF3 |
| NPAS3 |
| HERPUD1 |
| UQCC2 |
| RAB32 |
| DMD |
| CTTNBP2 |
| TECR |
| MSR1 |
| ANKMY1 |
| ARMC8 |
| FARP1 |
| STOX2 |
| FOCAD |
| PCED1B-AS1 |
| MTMR3 |
| MCRIP1 |
| DAAM1 |
| MPPED2 |
| HSPBAP1 |
| AHI1 |
| UACA |
| ARHGAP35 |
| LARP1 |
| PECAM1 |
| RSL24D1 |
| STAM2 |
| RHBDD1 |
| SYF2 |
| CD163L1 |
| STX10 |
| USP9X |
| CXCR4 |
| CRLF3 |
| CHCHD1 |
| MICAL3 |
| PLA2G4C |
| STK38 |
| ETFB |
| SHTN1 |
| MTM1 |
| TRPM2 |
| TMEM141 |
| ZBTB7C |
| LONP2 |
| HINT2 |
| SNAPIN |
| MRPL40 |
| LINC02256 |
| BMPR1A |
| MAPK8IP3 |
| DGKD |
| CNOT4 |
| MTR |
| ZNF37A |
| PNP |
| PSPC1 |
| NSD2 |
| ENSG00000290968 |
| PLEKHG5 |
| SEC11C |
| RCBTB2 |
| ZNF428 |
| BRAF |
| CEBPA |
| IPO9 |
| CHD6 |
| SPARCL1 |
| CCDC141 |
| NR3C2 |
| PLAGL1 |
| UBASH3B |
| MYDGF |
| RABEP1 |
| TCP11L1 |
| SUPT3H |
| PPCS |
| DEPDC5 |
| LINC01376 |
| TMEM51 |
| BOD1L1 |
| CC2D2B |
| PPM1L |
| RAPH1 |
| CMTM6 |
| MAP2K4 |
| NMT1 |
| HSD17B10 |
| LAMA4 |
| AUH |
| WRN |
| TRAF3IP2-AS1 |
| CCDC170 |
| TAB2 |
| ZNF131 |
| SELENOT |
| AP4E1 |
| HNRNPAB |
| PARG |
| LRPAP1 |
| ARSB |
| MCU |
| TMEM208 |
| TTC23 |
| KXD1 |
| MIB1 |
| SRBD1 |
| FAM20A |
| FAM193B |
| BCAR3 |
| ATP6AP1 |
| TNFSF12 |
| NCF1 |
| CHCHD5 |
| MAP3K3 |
| ZSCAN5A |
| NCOA7 |
| CERT1 |
| UMAD1 |
| HMGN1 |
| RHBDF2 |
| ECPAS |
| SOCS3 |
| KATNIP |
| ZNF236 |
| P2RY6 |
| STX16 |
| TRAPPC8 |
| EIF2B3 |
| DCP2 |
| GUSB |
| TMEM196 |
| LINC00106 |
| FMNL3 |
| ORC2 |
| SYNE1 |
| SSBP2 |
| CDC27 |
| ATP10D |
| MAGI1 |
| LRRK2 |
| MRPL11 |
| TLK1 |
| RASSF4 |
| MRPL17 |
| SMAD2 |
| RORA |
| ZNF26 |
| EIF6 |
| SELENOS |
| GPHN |
| OSBPL9 |
| EMSY |
| PEX14 |
| GSK3B |
| MED13 |
| IFNAR2 |
| XBP1 |
| MOSMO |
| CDK19 |
| TMEM205 |
| ITGBL1 |
| TRIP12 |
| HOXB7 |
| LTBP2 |
| GHR |
| GPC6 |
| MIGA1 |
| CACYBP |
| TTLL11 |
| ENSG00000273149 |
| KDM4B |
| EOLA2-DT |
| LIMK2 |
| XRCC4 |
| CAPN3 |
| POLR2G |
| SANBR |
| SFSWAP |
| PRRC2B |
| CCDC149 |
| TRAM1 |
| GTPBP10 |
| CSRP1 |
| PIK3CA |
| SAMD4A |
| TAF15 |
| C1GALT1 |
| ANO10 |
| ABHD3 |
| TRIM13 |
| LRRFIP2 |
| LINC03006 |
| PTPRG |
| NDUFV2 |
| TRIM56 |
| CROCCP3 |
| TAMM41 |
| CHMP4B |
| SAMD9L |
| RGS19 |
| SOGA1 |
| TRANK1 |
| MED23 |
| BTAF1 |
| GBF1 |
| TASP1 |
| NLRP1 |
| EP400 |
| SREK1 |
| ENSG00000291188 |
| ARHGEF18 |
| DPM3 |
| PTRHD1 |
| SLC25A11 |
| BCL2 |
| RFX3 |
| ECH1 |
| NDUFB5 |
| EMC6 |
| ENSG00000257322 |
| PSMC3 |
| NAGA |
| AGPAT3 |
| LARP4 |
| INTS9 |
| CSTF3 |
| ENSG00000251034 |
| SLC35A3 |
| PRDM2 |
| IDH3G |
| NUDT14 |
| NBEA |
| LINC02391 |
| CDR2 |
| PRKX |
| STXBP5 |
| PSTPIP2 |
| CSTPP1 |
| ME3 |
| ABAT |
| RAB34 |
| PTAR1 |
| SDK1 |
| MT2A |
| ZNF83 |
| SET |
| CAPN7 |
| CBFB |
| PALD1 |
| STRN3 |
| PFDN2 |
| CDON |
| CYB5A |
| XPO6 |
| ANO6 |
| CCAR1 |
| ZNF248 |
| KRIT1 |
| POGZ |
| METTL16 |
| TAF1B |
| PAFAH1B1 |
| TDRD10 |
| RYK |
| ZCCHC2 |
| PTPN2 |
| CUL3 |
| XPO1 |
| SLC3A2 |
| KIAA1328 |
| HCST |
| TMBIM1 |
| UBE3D |
| NRIP1 |
| PPIP5K2 |
| PCBD2 |
| SMS |
| PRKRIP1 |
| TMEM72-AS1 |
| PRPF40B |
| PA2G4 |
| FGL2 |
| NDUFA8 |
| ENSG00000290832 |
| CRAMP1 |
| GALK2 |
| EPG5 |
| RAD54L2 |
| TIAM1 |
| EIF3I |
| TMEM161B |
| FOXN2 |
| ACP2 |
| FANCL |
| MYSM1 |
| SMG7 |
| QSER1 |
| SELENOI |
| ZNF251 |
| TIAM2 |
| STIM2 |
| EPS15L1 |
| GYPC |
| DOCK5 |
| ZNF516 |
| ZNF644 |
| CLASRP |
| MT1X |
| FASTKD1 |
| MAFF |
| MOB1B |
| MMADHC |
| ZMYM2 |
| MDN1 |
| TCEAL3 |
| LCLAT1 |
| COMMD1 |
| CEP295 |
| FBXW11 |
| MICAL2 |
| SYNJ1 |
| MFHAS1 |
| ARHGEF3 |
| PLCB1 |
| SNRPC |
| ORMDL2 |
| ARNT |
| NUP50-DT |
| BRWD3 |
| SLC39A1 |
| NOD1 |
| ENSG00000237356 |
| ATP5F1C |
| TRAF3 |
| SEPTIN9 |
| ARL4A |
| YIF1A |
| MGA |
| CSPP1 |
| CYB5R3 |
| VPS35L |
| ANKRD13C |
| TNPO3 |
| TPT1-AS1 |
| GSTK1 |
| ENSG00000286162 |
| PSMD7 |
| ZNHIT6 |
| GPATCH2L |
| EFHD2 |
| LSM5 |
| NUDT1 |
| TRIM66 |
| AEBP2 |
| ENHO |
| SMARCAD1 |
| TDRD7 |
| NR1H2 |
| XPO7 |
| RSL1D1 |
| DNASE1 |
| AGTRAP |
| S100A13 |
| GDAP2 |
| ASB3 |
| SEMA6A-AS1 |
| HIGD1A |
| TENM4 |
| ANP32B |
| BTBD10 |
| SRSF9 |
| DEPTOR |
| ARHGAP17 |
| TUSC2 |
| NRDC |
| NVL |
| RER1 |
| NRP2 |
| NDUFB6 |
| LAGE3 |
| PLA2G4A |
| ENSG00000234961 |
| ENSG00000253557 |
| MRPL23 |
| SNRPF |
| TECPR2 |
| MTOR |
| CEP112 |
| PRKDC |
| CNOT2 |
| ZDHHC12 |
| MICU3 |
| PNN |
| CORO1A |
| SSBP1 |
| CABLES1 |
| SMIM15 |
| DENND5B |
| KANSL1-AS1 |
| ATF4 |
| SELENOM |
| DCXR |
| BLTP3A |
| SSU72 |
| SLIRP |
| WDR44 |
| MCCC1 |
| ZRANB3 |
| MICU1 |
| ARHGAP5 |
| ZNF562 |
| SZT2 |
| ZMAT2 |
| DDOST |
| PIGT |
| SNRPN |
| ZNF718 |
| SPAST |
| CAPS2 |
| STX2 |
| IFI44L |
| SMCHD1 |
| ZNF133 |
| ARID3A |
| PNRC2 |
| VRK2 |
| IPO11 |
| INO80 |
| MORC3 |
| HEBP1 |
| JPT1 |
| LARS2 |
| ATP10A |
| MTF2 |
| WDR49 |
| RUSC2 |
| S100PBP |
| RNF187 |
| KANSL1L-AS1 |
| NPHP3 |
| LDHB |
| CDYL2 |
| GDPD1 |
| AGPS |
| TSEN2 |
| IER3IP1 |
| PMS2P4 |
| CEP128 |
| CABIN1 |
| OTUB1 |
| TCF20 |
| ZDHHC1 |
| SNRPD3 |
| ZNF512 |
| AGPAT2 |
| UNK |
| PARP11 |
| NUP107 |
| HTATIP2 |
| DZIP1L |
| TLN2 |
| ELP3 |
| VGLL4 |
| SHLD2 |
| USP3 |
| YLPM1 |
| YPEL5 |
| PARN |
| PUM3 |
| PPFIA1 |
| RTL8C |
| ENSG00000287195 |
| SPINT2 |
| SNX19 |
| MED15 |
| POLA1 |
| FKTN |
| ENSG00000289013 |
| ZNF700 |
| ENOX2 |
| ST3GAL2 |
| ARHGEF12 |
| ENSG00000290737 |
| ZNF678 |
| C14orf119 |
| TMEM70 |
| POLE4 |
| PDLIM1 |
| ABCA9 |
| DTNBP1 |
| SPATS2 |
| AGTPBP1 |
| TGFBR1 |
| ZFYVE28 |
| ENSG00000290548 |
| PARP15 |
| MZF1 |
| CLIP1 |
| NUDCD3 |
| METTL25 |
| RRAGA |
| WAC |
| IWS1 |
| TUBB2A |
| CENPP |
| CDK12 |
| COPZ1 |
| ARL17A |
| SLC11A2 |
| CREB1 |
| PILRB |
| ENSG00000291075 |
| ALMS1 |
| EXT1 |
| VAMP5 |
| KPNA5 |
| ENSG00000254420 |
| GPAA1 |
| NAA35 |
| TMEM65 |
| SHOC1 |
| SNHG8 |
| TRAPPC11 |
| ZNF680 |
| SELPLG |
| LSM14A |
| KIFC3 |
| NSRP1 |
| RPH3AL |
| FUCA2 |
| MTX1 |
| AQR |
| PHTF2 |
| ABCC3 |
| KLHL22 |
| CHCHD3 |
| SVIL |
| TTC27 |
| CD52 |
| MAU2 |
| MYOF |
| KIAA0232 |
| ADK |
| RAB12 |
| SMARCC1 |
| OGT |
| OPA1 |
| TMEM134 |
| PDE7A |
| ENSG00000291117 |
| USP31 |
| CHD8 |
| RNF149 |
| ITFG2 |
| NOVA1 |
| ACAD10 |
| BEX4 |
| DHRS4 |
| HSPA1B |
| SRGAP1 |
| EEA1 |
| PLEKHA5 |
| C2CD3 |
| CRTC1 |
| EMC7 |
| ABI3 |
| MRPL14 |
| ENSG00000285692 |
| TTC3 |
| ENSG00000291067 |
| IFT88 |
| MRPS12 |
| TSTD3 |
| ENSG00000272368 |
| NDUFS3 |
| WARS2-AS1 |
| KIAA0825 |
| CLCN5 |
| SECISBP2 |
| DYNLT3 |
| YIPF3 |
| MITF |
| LIMCH1 |
| MTA3 |
| ERBIN |
| CDK4 |
| SNRPD1 |
| PPP2R5C |
| TTC21B |
| KLF12 |
| CHSY1 |
| NBPF11 |
| MFSD8 |
| NUTF2 |
| BHLHE40 |
| MYO15B |
| TYW1B |
| ZMAT1 |
| ZNF846 |
| NR6A1 |
| DENND3 |
| HIPK3 |
| ZNF524 |
| DPP8 |
| SVBP |
| UNC13B |
| BCKDK |
| PDAP1 |
| ZNF577 |
| ADAM10 |
| DDRGK1 |
| TMEM37 |
| LINC00174 |
| ACACB |
| CDK5RAP2 |
| FUT10 |
| ARHGAP12 |
| AAGAB |
| TLR2 |
| RAF1 |
| C1orf122 |
| AFTPH |
| COX7A2L |
| LRIG2 |
| FAM32A |
| TAPT1-AS1 |
| CARF |
| GTF2F2 |
| ZNF511 |
| RIF1 |
| ANAPC13 |
| SON |
| METTL8 |
| ANAPC15 |
| ZNF654 |
| NBPF19 |
| AP1S1 |
| LAMA2 |
| VPS13A |
| ZNF587B |
| SPEN |
| TBC1D9 |
| GPBAR1 |
| NEU3 |
| GM2A |
| PTPRD |
| EPN2 |
| CEP85L |
| EFL1 |
| RUBCN |
| SF3B4 |
| MRPL28 |
| SAT2 |
| CSTA |
| SH3RF1 |
| BLOC1S2 |
| DGKG |
| KLHDC1 |
| PSME4 |
| CNOT10 |
| RBM42 |
| TSTD1 |
| VAV2 |
| ST3GAL6 |
| TRIM44 |
| TFPT |
| SIMC1 |
| PACRGL |
| ZCRB1 |
| ZNF81 |
| LINC00662 |
| LUC7L |
| CDK6 |
| ZBTB47-AS1 |
| OGDH |
| C9orf78 |
| ZNF160 |
| ZNF451 |
| PDC-AS1 |
| MCOLN3 |
| CALCR |
| TBC1D19 |
| PIAS2 |
| ZNF44 |
| TARBP1 |
| PGS1 |
| MRPS36 |
| CHMP2B |
| MRPL12 |
| STON2 |
| NBR2 |
| MAP4K5 |
| SEC24D |
| USP48 |
| ZNF736 |
| GAK |
| ENSG00000274767 |
| ORC5 |
| GARNL3 |
| XYLT1 |
| PDLIM5 |
| LSM2 |
| ENSG00000213963 |
| ANTXR2 |
| ARF3 |
| LRRC69 |
| PRMT3 |
| CNIH1 |
| HACD4 |
| TRMT13 |
| ENSG00000291283 |
| ZNF785 |
| DNAJC11 |
| ZFP14 |
| LINC02100 |
| TMEM109 |
| ENSG00000291177 |
| ENSG00000226824 |
| DENND11 |
| STOML2 |
| ZNF652 |
| ACIN1 |
| WDR59 |
| ANKUB1 |
| SPCS3 |
| NBEAL1 |
| PDE3B |
| CD300C |
| LINC01409 |
| CCDC115 |
| MLH3 |
| OSTC |
| MAN1A2 |
| PDE8A |
| TCF12-DT |
| TAPT1 |
| MRPL36 |
| SIK2 |
| PIN1 |
| PCSK7 |
| MSL2 |
| GABARAPL1 |
| FSD1L |
| COG6 |
| PLEKHA1 |
| TEX264 |
| RNF121 |
| HMGA1 |
| E2F3 |
| EZH1 |
| MIR100HG |
| ST7L |
| POLR3GL |
| ATG10 |
| GLI3 |
| BRD1 |
| SEPHS2 |
| ZNF254 |
| STRN |
| FGD3 |
| IQGAP2 |
| ACER3 |
| CDK17 |
| RSF1 |
| FAM149B1 |
| NRBF2 |
| CAMK2D |
| SLC12A6 |
| CYB5D1 |
| PSMB9 |
| PLXNA2 |
| SLC38A4-AS1 |
| DCAF6 |
| ERCC1 |
| ENSG00000290453 |
| MDH1 |
| SLC25A1 |
| NLRC5 |
| PRDX4 |
| DCLRE1C |
| PDPR |
| TMCO4 |
| MID1IP1 |
| GNG11 |
| KLF10 |
| NPL |
| TMX3 |
| CRIM1 |
| VPS39 |
| LYSET |
| DCAKD |
| ENSG00000259363 |
| POLR2F |
| EVA1C |
| UBA6 |
| URI1 |
| SPOPL |
| SERGEF |
| ALOX5 |
| KANTR |
| ACP1 |
| CTDNEP1 |
| TAF1 |
| ZNF782 |
| BAD |
| NUDT16 |
| STX12 |
| CHMP1B |
| MRPL27 |
| LINC02987 |
| RIGI |
| AGK |
| SMIM19 |
| STX18-AS1 |
| KANSL3 |
| FAM219A |
| IGFLR1 |
| MAPK10 |
| LTC4S |
| FOXP2 |
| RPP21 |
| GATAD2A |
| DNHD1 |
| LRSAM1 |
| POM121 |
| ISOC2 |
| TMEM181 |
| GIMAP4 |
| MIA2 |
| GALNT2 |
| ALKBH1 |
| MAP3K20 |
| NAPB |
| PIK3CD |
| NFKBIZ |
| ARL4D |
| MRPS7 |
| MAN1C1 |
| CHSY3 |
| SETBP1 |
| CCDC146 |
| SEMA5A |
| CCRL2 |
| VCAM1 |
| PHC2 |
| SLC30A6 |
| EDRF1 |
| ENSG00000251314 |
| CCNY-AS1 |
| PIK3R1 |
| PACSIN2 |
| SNF8 |
| ENSG00000291171 |
| NUDCD2 |
| GPS1 |
| ENSG00000286608 |
| GCC2 |
| UROD |
| TMIGD3 |
| GNG2 |
| ACAD11 |
| XYLB |
| TBL1X |
| SLC30A9 |
| PPIC |
| CDC42BPB |
| ENSG00000287117 |
| ZNF385D |
| RAB30-DT |
| ANTKMT |
| HEATR3 |
| CACNA2D1 |
| RTTN |
| SLC31A2 |
| PSMB2 |
| COPRS |
| PPP2R2D |
| TMEM144 |
| METRNL |
| SERPINA1 |
| UPP1 |
| BBX |
| GTF2H5 |
| TMEM131L |
| ATG2B |
| GDE1 |
| GLMN |
| SPATA7 |
| ENSG00000274265 |
| THUMPD2 |
| DCTPP1 |
| TMEM87A |
| PPP1R11 |
| MAD1L1 |
| ERVK13-1 |
| CRYBG3 |
| KLHL2 |
| USP4 |
| SKIC3 |
| TMEM241 |
| SH2B3 |
| ENSG00000280434 |
| CKS2 |
| ZMYND11 |
| NNT |
| PREP |
| PPFIBP2 |
| SPTSSA |
| NCKAP1L |
| DYNC2H1 |
| OTUD1 |
| NGRN |
| MAP2K3 |
| ATOSA |
| PLEKHG1 |
| CCDC107 |
| ESCO1 |
| CDK14 |
| HEATR1 |
| ELL |
| CAMLG |
| ADISSP |
| FNTB |
| DPYD-AS1 |
| ARMH4 |
| ENSG00000289581 |
| FAM162A |
| MCRIP2 |
| IGF1R |
| HNRNPH1 |
| PRKG1 |
| SLC35D2 |
| TBC1D4 |
| ELP4 |
| PIGB |
| P2RY14 |
| NME1 |
| STAT1 |
| KAT2B |
| ZNF141 |
| RFFL |
| FLVCR2 |
| ARMC2 |
| GABBR1 |
| H1-10 |
| GFRA2 |
| FBXL7 |
| SMARCA2 |
| NAIP |
| ENSG00000291003 |
| RAB33B-AS1 |
| ZNF398 |
| SNRPE |
| C12orf4 |
| PRORP |
| SBNO2 |
| EML4 |
| APLF |
| PRXL2C |
| NPLOC4 |
| PDE7B |
| KDM2B-DT |
| STAG3L5P |
| DNAJB14 |
| STAG3L4 |
| ANXA4 |
| PRMT2 |
| TPP2 |
| NR2C2 |
| ARHGEF35-AS1 |
| RCSD1 |
| ZNF714 |
| SLC49A4 |
| ARRDC3-AS1 |
| RAD52 |
| TBCE |
| ELOVL1 |
| KPNA1 |
| ARC |
| NSD3 |
| SCAI |
| POT1 |
| SH3D19 |
| RNPEPL1 |
| CLN8-AS1 |
| C9orf85 |
| SNN |
| SLC9A8 |
| PUF60 |
| TMEM164 |
| SH3KBP1 |
| RPL26L1 |
| LINC00630 |
| KCNK13 |
| UBE2A |
| SSBP3 |
| PARD3 |
| COL6A3 |
| RAD50 |
| NBPF9 |
| THBD |
| ZNF585B |
| ENSG00000288755 |
| CASK |
| GLA |
| EMBP1 |
| DDI2 |
| NFIC |
| RBM41 |
| DNAJC19 |
| PARP9 |
| AFG1L |
| LRP10 |
| ZSCAN30 |
| CD99P1 |
| ANXA2R-AS1 |
| TRIB1 |
| OSBPL1A |
| NFIB |
| HCG18 |
| AP2B1 |
| SMIM30 |
| CWC27 |
| DHX35 |
| ANTXR1 |
| PRDX6-AS1 |
| CAVIN3 |
| ATG4C |
| URGCP |
| HAX1 |
| ENSG00000289007 |
| NDUFAF8 |
| SRA1 |
| MROH1 |
| CWC15 |
| VAC14 |
| KCNQ1 |
| ALS2 |
| SMIM20 |
| LMF1 |
| AMPD3 |
| PMS1 |
| ST3GAL5 |
| KMT2D |
| MMS22L |
| TCF25 |
| BRMS1 |
| MAST2 |
| POLE3 |
| CTSF |
| ENSG00000244055 |
| NIPAL2 |
| ENSG00000213600 |
| CCDC93 |
| TEP1 |
| CRIP2 |
| DNAJC24 |
| RAB8A |
| SNAPC3 |
| PER3 |
| ENOSF1 |
| SAP30L-AS1 |
| CCT6P3 |
| SHQ1 |
| COL14A1 |
| TMEM63A |
| ATP2B1-AS1 |
| ZC3H7A |
| PRANCR |
| STX11 |
| ZER1 |
| ZNF593 |
| PMM2 |
| C12orf57 |
| MAGOH |
| C7orf50 |
| ENSG00000291147 |
| FKBP15 |
| B3GALNT2 |
| FARP2 |
| CPT1A |
| PLCL1 |
| SRGAP3 |
| NCK1 |
| RUNX1T1 |
| ENSG00000256948 |
| TXN2 |
| DICER1 |
| SEC31A |
| LNPEP |
| MAPK1 |
| GRAMD4 |
| COL6A1 |
| TPRA1 |
| DYNLT1 |
| ADAMTS6 |
| TCEAL8 |
| CKMT2-AS1 |
| MED28-DT |
| RTL8A |
| NR2C1 |
| STX18 |
| SRSF4 |
| WBP2 |
| RAB11FIP2 |
| TRIM14 |
| ZNF333 |
| DROSHA |
| WAPL |
| FBXO38 |
| NPIPB15 |
| BMT2 |
| ZNF573 |
| DCP1B |
| TADA2A |
| ZNF235 |
| ZNF804A |
| RRAS |
| TIMM17B |
| SCN1A-AS1 |
| TXK |
| IL4R |
| OTULIN |
| KIAA0753 |
| LMBR1 |
| TUBGCP5 |
| HLA-DQB2 |
| NPHS1 |
| G2E3 |
| TNC |
| RNF217-AS1 |
| HTR1F |
| ENSG00000250195 |
| UVSSA |
| USO1 |
| UEVLD |
| DPM2 |
| TMEM62 |
| H1-0 |
| IL7 |
| COA6 |
| CNST |
| CALHM6 |
| FGGY |
| MRPL55 |
| LAMTOR3 |
| DIP2C |
| ABCB7 |
| PTPRS |
| MYG1 |
| ACOT11 |
| EP300 |
| TPPP3 |
| LYPLAL1-AS1 |
| CASD1 |
| EXO5-DT |
| GABRB2 |
| SPOPL-DT |
| ANAPC4 |
| SHLD1 |
| ADNP |
| CYTH4 |
| SIRPB2 |
| ITPKB |
| ABI2 |
| INSR |
| IGSF21 |
| PLIN1 |
| CCDC191 |
| VMO1 |
| CCDC28A |
| ISG15 |
| PLEKHG2 |
| DMAC1 |
| MTMR1 |
| MIR646HG |
| ARSG |
| MRPL21 |
| PI4K2B |
| CHMP5 |
| GATD3 |
| MOB3A |
| TRMT10B |
| ZBED4 |
| GFPT1 |
| SIGIRR |
| PGLS-DT |
| BCL2L1 |
| ENSG00000284959 |
| PDHX |
| FBXO34 |
| FBL |
| KAT6B |
| GLIS3 |
| NAA15 |
| PTPDC1 |
| BSCL2 |
| MAPRE1 |
| ENSG00000249738 |
| BOLA3 |
| HNRNPH2 |
| TFEB |
| ENSG00000240401 |
| NAA16 |
| PKD1L3 |
| SLC25A26 |
| NEK11 |
| PPP1R15B |
| FBXL15 |
| PER1 |
| RPL23AP7 |
| EXOG |
| PTPRE |
| CCT7 |
| CSNK1G3 |
| RNASE2 |
| PGRMC1 |
| TP53TG1 |
| COMMD9 |
| KHDC4 |
| COA4 |
| PTCD3 |
| IDE |
| POP7 |
| AXL |
| ENSG00000269940 |
| SPATA1 |
| MIPEP |
| CDIPT |
| TMEM9B |
| C11orf98 |
| VKORC1L1 |
| CEP95 |
| ENSG00000289564 |
| UBL7 |
| CD38 |
| ANKRD17-DT |
| PMVK |
| CADPS2 |
| NEK1 |
| ENSG00000287306 |
| ENSG00000290683 |
| SRGAP2C |
| TGIF1 |
| LMCD1-AS1 |
| GOLM2 |
| MED14 |
| ZNF521 |
| AFDN |
| BANP |
| SLC12A2-DT |
| NIN |
| EFR3A |
| FGD5-AS1 |
| CAMSAP1 |
| RAP1GAP2 |
| PRRG1 |
| NUP205 |
| ILF2 |
| ZNF33B |
| GAB1 |
| LUC7L2 |
| PTBP3 |
| MPG |
| MBNL2 |
| CRYZL1 |
| TGFBR2 |
| STXBP4 |
| DDX31 |
| CLK4 |
| HLA-DOA |
| TAF4 |
| PSMA4 |
| GPC5 |
| ENSG00000289156 |
| TNFAIP8L2 |
| GABPB2 |
| VEZT |
| MAP4K3 |
| COMMD8 |
| CCT5 |
| PRKCA |
| ORAI3 |
| MSANTD2 |
| ADD1 |
| ADCY9 |
| RARS2 |
| UQCC3 |
| FANCM |
| ATP6V0D1-DT |
| ACAA2 |
| SLC4A8 |
| SP140L |
| CD36 |
| ATP6V0A1 |
| ZNF559 |
| TRIT1 |
| PNPT1 |
| MMADHC-DT |
| XNDC1N |
| C2CD5-AS1 |
| ERCC6L2-AS1 |
| RAB11FIP3 |
| SMIM8 |
| MARCHF6 |
| TSC1 |
| TOB1 |
| SLC35G2 |
| DIAPH2-AS1 |
| ENSG00000238142 |
| SP110 |
| TALAM1 |
| GLCCI1 |
| MYO19 |
| SS18 |
| CUL1 |
| UGGT1 |
| USP54 |
| TMED4 |
| ENSG00000289228 |
| TCP1 |
| EPM2A |
| CEP250-AS1 |
| DUT |
| PSMD1 |
| PITPNC1 |
| IL10 |
| TPRG1 |
| SPICE1 |
| ENSG00000290931 |
| RAB6A |
| ARHGEF6 |
| FGF10 |
| NGLY1 |
| INSYN2B |
| LRRC37B |
| MMS19 |
| UBE3A |
| ENSG00000237188 |
| LINC00910 |
| ASXL1 |
| SLC25A12 |
| CD2AP |
| CENPC |
| MAPKBP1 |
| SLC25A24 |
| IK |
| CDC26 |
| PRKCB |
| ZCCHC14 |
| BICC1 |
| MARS1 |
| PPHLN1 |
| CA5B |
| RNF115 |
| TSR2 |
| CD200R1 |
| MRPL24 |
| DHX32 |
| HSPG2 |
| FAM200B |
| ZNF430 |
| DDIT4 |
| RERG |
| DSE |
| AREL1 |
| BCL7B |
| ATP2B4 |
| CARS1 |
| CSAD |
| NCOA5 |
| STK4 |
| INTS7 |
| NOTCH2 |
| GPR155 |
| FTCDNL1 |
| PBX1 |
| ME2 |
| CACUL1 |
| RFWD3 |
| MRPL1 |
| NUP88 |
| NEDD4 |
| PCGF3 |
| ACSF3 |
| NDUFA5 |
| THYN1 |
| C2orf27A |
| RFTN1 |
| RNF170 |
| SDCBP2-AS1 |
| IL1A |
| HACE1 |
| WDR27 |
| IFI44 |
| CTCF |
| CLCN6 |
| PARP12 |
| MEF2C-AS1 |
| RILP |
| LRPPRC |
| SLC39A13-AS1 |
| FKBP4 |
| SYT17 |
| ARL2BP |
| USP45 |
| SGPP1 |
| ZNF621 |
| SLC25A17 |
| MRPS18C |
| MPHOSPH8 |
| ZFYVE16 |
| AKR7A2 |
| PALS1 |
| SKI |
| HEIH |
| SERPINI2 |
| EIPR1 |
| DDX60 |
| AVEN |
| ZNF346 |
| MPST |
| CLEC3B |
| ZKSCAN7-AS1 |
| RANBP10 |
| AGAP9 |
| ZNF627 |
| SUCLA2 |
| CTPS2 |
| GALK1 |
| OSBPL11 |
| GFER |
| ABHD14B |
| LRRC37A2 |
| TSIX |
| DCUN1D2 |
| GPR141 |
| TNPO1 |
| L3MBTL1 |
| CEP152 |
| ENSG00000230333 |
| P2RX7 |
| PHF3 |
| ZNF655 |
| NCK1-DT |
| SLC35F6 |
| GTF2I-AS1 |
| NBPF12 |
| ENSG00000290427 |
| RELL1 |
| CCDC80 |
| ZBTB37 |
| IMP4 |
| ENSG00000254288 |
| ENSG00000228655 |
| CPED1 |
| PPP4R3B |
| INTS8 |
| FANCC |
| HS2ST1 |
| GPRIN3 |
| RBL1 |
| ZNF197 |
| HEXIM1 |
| GLYR1 |
| LSM6 |
| TFB1M |
| ASB7 |
| ENSG00000251652 |
| TNK2 |
| ANAPC7 |
| ZFHX2-AS1 |
| GTF3C2 |
| RHOBTB3 |
| PAX8-AS1 |
| TBCEL |
| ENSG00000273118 |
| MS4A14 |
| RHOXF1-AS1 |
| PHYH |
| GATM |
| WAS |
| PTK2B |
| LDB2 |
| CAMK2G |
| GALNT1 |
| ENSG00000250075 |
| NALF1 |
| SLC35F1 |
| URB1 |
| MCOLN2 |
| VPS13B-DT |
| GALNT7 |
| MRPS27 |
| MIRLET7A1HG |
| SDSL |
| WLS |
| PRDX2 |
| MEF2B |
| RANGRF |
| NME7 |
| TAF9 |
| USP42 |
| ENSG00000235979 |
| APEX1 |
| TMLHE-AS1 |
| CDC14A |
| IFT80 |
| SERINC2 |
| COX10 |
| SNRK |
| SLC25A16 |
| TRNT1 |
| CEP135 |
| LPGAT1 |
| LINC02615 |
| SRRM2 |
| PLXND1 |
| ICE2 |
| ZNF66 |
| ZNF506 |
| NUP43 |
| CSF2RA |
| RIC8B |
| A1BG |
| PHLDA2 |
| MIDN |
| PGM5 |
| GNG12-AS1 |
| CDIN1 |
| UBP1 |
| MSRA |
| CEP290 |
| MAPK8 |
| PCDH9 |
| MAZ |
| LINC00685 |
| PPCDC |
| WDR41 |
| METTL5 |
| CZIB |
| TTN-AS1 |
| FAM185A |
| NCK2 |
| DSTYK |
| RILPL1 |
| TEFM |
| GOLGA1 |
| DAPK1-IT1 |
| CFAP44 |
| DNAJC16 |
| STAT2 |
| ZNF875 |
| TMEM87B |
| TENT4A |
| LILRA2 |
| C22orf39 |
| WBP1L |
| TRAPPC13 |
| MRPS26 |
| LRRC59 |
| PRPF3 |
| COL1A1 |
| HCG17 |
| DGCR2 |
| ENSG00000285667 |
| HOXB3 |
| DGCR8 |
| CLPP |
| LINC01176 |
| CEP43 |
| TULP4 |
| SNHG22 |
| AKAP8L |
| EPB41L2 |
| HNMT |
| LINC02908 |
| WBP2NL |
| FAM91A1 |
| PGGT1B |
| LINC02978 |
| ENSG00000227531 |
| SENP1 |
| BTN2A1 |
| MRPS16 |
| RHOXF1 |
| GSTCD |
| JAGN1 |
| ICE1 |
| NOX4 |
| TMEM60 |
| LRRC25 |
| ENSG00000291299 |
| ZNF565 |
| EGLN2 |
| MAMDC2-AS1 |
| PRKD3 |
| MAD2L1-DT |
| ENSG00000291207 |
| ADPGK |
| COA8 |
| MCC |
| SPR |
| DLG2 |
| PITPNA |
| FCGR2B |
| ENSG00000286787 |
| EPB41 |
| MAP3K7 |
| ENSG00000249207 |
| CCL18 |
| TAF10 |
| KHDRBS2 |
| RHOBTB1 |
| ORAI1 |
| DNAJA2 |
| CASP8AP2 |
| PRDM10 |
| DCUN1D4 |
| RSL1D1-DT |
| TNFRSF11A |
| TBC1D2 |
| MCM3AP |
| WDR43 |
| PHLDA1 |
| ENSG00000291214 |
| CEP89 |
| GLCE |
| CEP164 |
| ENSG00000260971 |
| HYAL2 |
| ZFAT |
| COPG2 |
| RPS6KA5 |
| PHF12 |
| GCSH |
| ZHX3 |
| PHF20 |
| ENSG00000287124 |
| TPGS1 |
| DNAJC4 |
| CUEDC2 |
| DSCAML1 |
| TVP23C |
| HCK |
| ZNF43 |
| CAMSAP2 |
| ZNF592 |
| CLIP4 |
| TANGO2 |
| FBN1 |
| PRKCE |
| MTMR9 |
| CCNL2 |
| NAA10 |
| AKAP11 |
| ULK2 |
| PDE1A |
| HOOK3 |
| CHM |
| LINC03076 |
| TMEM126A |
| TTI1 |
| BCL2L1-AS1 |
| ARHGEF40 |
| CREBZF |
| CMTM3 |
| ENSG00000236449 |
| RGS2 |
| ENSG00000290833 |
| FUBP3 |
| ENSG00000232063 |
| PITHD1 |
| IP6K1 |
| DNAH14 |
| USP49 |
| ERCC8 |
| KATNAL1 |
| SMARCA4 |
| MIR155HG |
| KIAA0586 |
| PSMD6 |
| CYB5R1 |
| TAGAP-AS1 |
| MEG3 |
| ABHD15-AS1 |
| LINC02762 |
| RNF144B |
| SEMA4B |
| APTX |
| ABCD3 |
| ZFPM2 |
| ALG13 |
| SCGB2B2 |
| ASMTL-AS1 |
| ENSG00000291054 |
| MRPS33 |
| TTL |
| DDX46 |
| GTF2H2 |
| CFDP1 |
| MIRLET7IHG |
| SMAP2 |
| ENSG00000291201 |
| PSMC4 |
| ENSG00000286288 |
| SLC22A15 |
| DCLK1 |
| SNCAIP |
| ENSG00000289298 |
| SEC31B |
| PLIN4 |
| MTMR2 |
| DHDDS |
| MTFMT |
| GOLGB1 |
| ATP11B |
| RASA3 |
| ZNF41 |
| NARS2 |
| AKAP7 |
| PRELID3A |
| AGA-DT |
| MPHOSPH9 |
| NUP133 |
| LOXL3 |
| VAPB |
| TRIM37 |
| GDAP1 |
| NELFE |
| ENSG00000290999 |
| RAP2C-AS1 |
| STAT3 |
| ENSG00000253983 |
| C18orf25 |
| SPATS2L |
| SNRPB2 |
| NDUFAF6 |
| XPR1 |
| ZBTB1 |
| GOLGA7 |
| ZNF124 |
| LYPLAL1 |
| CNPPD1 |
| TBCD |
| PRMT7 |
| ABCC10 |
| CDKN2B-AS1 |
| MRRF |
| BORCS7 |
| ANAPC10 |
| ZBTB46 |
| DDIT3 |
| FHAD1 |
| MILR1 |
| MIR181A2HG |
| ARID4A |
| DCAF1 |
| PUS7 |
| UBE2S |
| FAM89B |
| ENSG00000291174 |
| ADGRG6 |
| ENSG00000258526 |
| TPRKB |
| ANKS3 |
| GSDMA |
| DCTN4 |
| RCL1 |
| FBXO22 |
| CHRAC1 |
| ANK3 |
| ENSG00000226471 |
| PRPF39 |
| IL17RA |
| PFDN6 |
| CD2BP2 |
| CUL4A |
| SWT1 |
| POLR3A |
| SACM1L |
| SCD |
| MAP3K4 |
| MAVS |
| PPM1M |
| ENSG00000288018 |
| GNA12 |
| TENM3 |
| LIX1-AS1 |
| PPP4R1 |
| USP10 |
| ENSG00000286423 |
| TECPR1 |
| C1R |
| FCGR1A |
| ZFYVE26 |
| ELOF1 |
| ZFP64 |
| PPFIBP1 |
| USP8 |
| UTP20 |
| MMP19 |
| DCAF17 |
| ZNF449 |
| ENSG00000290924 |
| RBM12B |
| TYW5 |
| PLXDC1 |
| KLRK1 |
| SMIM10L1 |
| NMB |
| ARHGEF2 |
| ZNRD2 |
| GUSBP2 |
| COMP |
| DAAM2 |
| ZDHHC13 |
| PAXX |
| SLC16A7 |
| SULT1A1 |
| PIGU |
| COMMD3 |
| ZC3H3 |
| CEBPG |
| WDFY1 |
| LPAR1 |
| TMTC3 |
| GPR135 |
| SERTAD3 |
| UNKL |
| EFCAB13 |
| PIGG |
| ANKRD27 |
| ENSG00000227598 |
| D2HGDH |
| NFATC2IP |
| GAS6-AS1 |
| SLC39A3 |
| ATG101 |
| MPRIP |
| IFI16 |
| RUNX2 |
| FAM120C |
| ZMYM1 |
| OR2A1-AS1 |
| CSNK2A1 |
| CATSPER2 |
| FPGT |
| MRE11 |
| SLC45A4 |
| POP4 |
| MRPL4 |
| ACVR1 |
| NEK3 |
| ZNF266 |
| C2CD2 |
| PQBP1 |
| HUWE1 |
| KPNA3 |
| PDCL3 |
| CFH |
| GADD45G |
| PTPN4 |
| NLRP3 |
| TTLL3 |
| SS18L2 |
| ENSG00000290709 |
| SASH1 |
| TRIM4 |
| EXD3 |
| CDK11A |
| DPY19L4 |
| CROCCP2 |
| TADA3 |
| ENSG00000287778 |
| SLC1A3 |
| DPY19L3 |
| BCKDHB |
| GPALPP1 |
| YIPF4 |
| RPP25 |
| ENSG00000290073 |
| RALGPS1 |
| EBNA1BP2 |
| LINC02798 |
| ENSG00000288891 |
| CNOT6 |
| FAF2 |
| USPL1 |
| CMTM7 |
| NCBP2AS2 |
| ZNF117 |
| CCNQ |
| ENSG00000290876 |
| TCAIM |
| ENSG00000286194 |
| SPOP |
| ETNK1 |
| ISCA2 |
| PLAAT4 |
| ENSG00000267523 |
| ZC3H7B |
| MMP16 |
| NRDE2 |
| XXYLT1 |
| NOMO3 |
| LINC02981 |
| H2AX |
| TBC1D32 |
| EIF4E |
| EIF2AK2 |
| MINK1 |
| WBP1 |
| RPA3 |
| ZC3H6 |
| MECOM |
| CFP |
| PCBP3 |
| HDAC7 |
| QRICH1 |
| GLOD4 |
| ENSG00000243176 |
| DPH3 |
| COMMD4 |
| COMMD5 |
| LYSMD2 |
| ARMCX5-GPRASP2 |
| PPME1 |
| MTMR10 |
| SCYL3 |
| SLC25A43 |
| CMKLR2 |
| TGFBR3 |
| NSMAF |
| UBA6-DT |
| MARVELD1 |
| TMTC4 |
| TM2D2 |
| TAB3 |
| IRAK1BP1 |
| MKKS |
| MCTP2 |
| DCP1A |
| C1S |
| ABCB10 |
| GFUS |
| RNF213-AS1 |
| NUBP2 |
| TBC1D16 |
| CTDSPL |
| ANAPC5 |
| RSKR |
| RNF135 |
| DET1 |
| COG3 |
| TSSC4 |
| MIOS |
| ATP6AP1-DT |
| PRDM4 |
| POLR1A |
| DDHD1 |
| TINF2 |
| ENSG00000267136 |
| SUPT20H |
| PSMG3 |
| YIF1B |
| TBK1 |
| MTHFR |
| RAD51D |
| MED29 |
| PISD |
| TRIM28 |
| BIVM |
| GOSR1 |
| PRMT1 |
| MGRN1 |
| SAP130 |
| CCDC171 |
| AHSA1 |
| FLCN |
| ATF2 |
| SURF2 |
| RNASEH2B |
| ANKRD13D |
| SNRNP25 |
| NT5C |
| MROH7 |
| MBNL3 |
| TRADD |
| MARK4 |
| CLIP2 |
| GFM2 |
| COBLL1 |
| CACNA2D3 |
| ZNF561-AS1 |
| LINC01145 |
| HCG27 |
| SYT1 |
| POC1B |
| UBE2O |
| CHIC1 |
| NT5DC1 |
| PMAIP1 |
| ENSG00000230649 |
| PRIMPOL |
| SYPL1 |
| RASSF3 |
| ZNF354B |
| ECM2 |
| MTERF1 |
| HMG20B |
| CMSS1 |
| GPRC5A |
| SLC26A3 |
| PPM1D |
| SOCS7 |
| SLC35E2B |
| PRICKLE2 |
| MIR223HG |
| ENSG00000289421 |
| CCDC125 |
| FIP1L1 |
| EVI2A |
| ZMIZ2 |
| ZNF808 |
| SEC63 |
| LPCAT2 |
| ZNF69 |
| DAB1 |
| FMNL1-DT |
| TMEM120A |
| BRD4 |
| DENND2B |
| LSM1 |
| ARHGEF9 |
| GCFC2 |
| IMPDH2 |
| VEGFC |
| FBH1 |
| MCUR1 |
| DNAH1 |
| ENSG00000262265 |
| XPO5 |
| IFI6 |
| FGD6 |
| FRG1-DT |
| TTC39C |
| SS18L1 |
| MIF4GD |
| ENSG00000290989 |
| ENSG00000286145 |
| DHRS7B |
| MFSD10 |
| ENSG00000223727 |
| ANGEL1 |
| POLR3B |
| RRP7A |
| NAPEPLD |
| SIGLEC11 |
| DTWD2 |
| ZNF566 |
| ENSG00000259678 |
| LINC01004 |
| FRMD3 |
| ASCC1 |
| PDZD2 |
| EXOSC4 |
| EXT2 |
| HMGB2 |
| ATAD5 |
| CYP20A1 |
| ENSG00000253194 |
| ENSG00000253720 |
| AFF3 |
| THBS4 |
| STARD7-AS1 |
| PLPP1 |
| BDNF-AS |
| ABHD14A |
| PHAF1 |
| NHERF1 |
| COA5 |
| AKIP1 |
| MRPS28 |
| GASK1A |
| HCFC2 |
| THOC7 |
| ZC3H12D |
| WDSUB1 |
| LEMD3 |
| ANKRD42-DT |
| MRPL37 |
| EPHA3 |
| SIL1 |
| RCN3 |
| ITGAL |
| SMYD4 |
| SCOC |
| MAML1 |
| ATG14 |
| SH3PXD2B |
| SLC37A1 |
| SLC35F5 |
| RESF1 |
| ZNF253 |
| TSNARE1 |
| TMUB1 |
| ZNF14 |
| LINC03051 |
| LEPR |
| OTUD6B |
| CREB3L2 |
| C15orf61 |
| TWF1 |
| MANF |
| RAB27A |
| ENSG00000270792 |
| MAGED2 |
| NSUN6 |
| ENSG00000290476 |
| APEH |
| PRELID3B |
| TMEM120B |
| SNHG32 |
| CERS2 |
| KCNT2 |
| GK5 |
| ARPC5L |
| P3H2 |
| NAPRT |
| RETREG2 |
| TENT4B |
| PDE5A |
| ZNF704 |
| CCZ1B |
| AGAP5 |
| LINC01422 |
| FAXDC2 |
| ANKRD6 |
| SEC23A |
| FMC1 |
| DNAJB9 |
| RAI1 |
| ENSG00000268081 |
| GADD45A |
| ENSG00000264895 |
| SNAPC5 |
| IARS1 |
| SDS |
| UBE2G2 |
| AAMP |
| DAGLB |
| OSBPL10 |
| ENSG00000240291 |
| KLHL8 |
| ITGAV |
| TNFRSF25 |
| GGA3 |
| C1orf112 |
| G6PC3 |
| ENSG00000285679 |
| UBE2M |
| C1orf159 |
| MOCS2 |
| ZC3H12A |
| MISFA |
| SAMD4B |
| POLR1H |
| ENSG00000257764 |
| TRIP11 |
| BMERB1 |
| GOLPH3L |
| TBC1D22B |
| IGF2R |
| ENSG00000288942 |
| MAN2A1 |
| PLSCR3 |
| RASA4B |
| NRAD1 |
| DCK |
| ZNF765 |
| ECHDC2 |
| DCBLD1 |
| BLTP2 |
| DPH6-DT |
| COPB2-DT |
| LRRC28 |
| RHBDD2 |
| SEC23IP |
| FBXL5 |
| ENSG00000229618 |
| ZNF581 |
| ERI3 |
| TSR3 |
| SAV1 |
| TMEM245 |
| QRSL1 |
| OXNAD1 |
| CREB3 |
| ZNF106 |
| FAM13A-AS1 |
| GATAD1 |
| C5 |
| MKNK2 |
| SORBS2 |
| CCDC18 |
| REPS2 |
| ZBTB26 |
| SIRPD |
| ZNF107 |
| TBX15 |
| PRELID2 |
| PYCR2 |
| NUP42 |
| AMMECR1 |
| GIMAP1 |
| ENSG00000273691 |
| HTATSF1 |
| CRYBB2P1 |
| ITGA9-AS1 |
| YPEL2 |
| COLQ |
| ENSG00000279277 |
| PPA2 |
| LIPE-AS1 |
| ZNF32 |
| GEN1 |
| BLTP3B |
| DBF4B |
| PPP1R14B |
| PDZD8 |
| KPNA6 |
| RGL3 |
| UNC119 |
| PIGP |
| FRMD4A-AS1 |
| CHCHD7 |
| LYPLA2 |
| ATG5 |
| ODF2L |
| FGF14 |
| CCL2 |
| PLXNC1 |
| ZNF493 |
| CNIH3 |
| PPIL2 |
| AGBL3 |
| ENSG00000291159 |
| RGS12 |
| ENSG00000285744 |
| AVPI1 |
| ARHGEF10 |
| RNF19A |
| IL15RA |
| TERF2 |
| CCL20 |
| AKR1C3 |
| DNMBP |
| ENSG00000254538 |
| NRIR |
| POLR3K |
| CEMIP |
| MAP7 |
| PTPN21 |
| PHLDB2 |
| XPNPEP3 |
| ENSG00000286264 |
| ZYG11B |
| SYMPK |
| NPRL3 |
| ZNF439 |
| OOEP |
| FAM151B |
| GALNT11 |
| PRR12 |
| SLC38A7 |
| CPPED1 |
| MSI2 |
| ZNF789 |
| MRPL47 |
| NDUFS1 |
| MAT2B |
| PAQR3 |
| HMG20A |
| TAF1D |
| SLC15A2 |
| FNBP1L |
| TRMT2B |
| TUBD1 |
| ENSG00000254604 |
| ZNF528-AS1 |
| RPRD1B |
| ENSG00000264853 |
| VPS25 |
| GPR173 |
| CEP126 |
| EVL |
| PTGS2 |
| ALG3 |
| KCMF1 |
| RNF216P1 |
| TRIAP1 |
| PIK3C2B |
| BEST1 |
| C1RL |
| ELP1 |
| EIF1B-AS1 |
| ENSG00000288526 |
| DDA1 |
| PRR5 |
| ACSF2 |
| ENSG00000250777 |
| ZNF75A |
| MLKL |
| CASC15 |
| PCSK6 |
| PLEKHH2 |
| GTF2IRD1 |
| EPHB2 |
| CACNA1C |
| RBM28 |
| IDH3B |
| WDR35 |
| NPAT |
| CCDC57 |
| PRH1 |
| TMEM267 |
| DNAJA4 |
| PRLR |
| EPHA1-AS1 |
| BAZ1B |
| ENSG00000265334 |
| FAM210B |
| SPECC1 |
| SAMD8 |
| DIPK1A |
| CTTNBP2NL |
| PTPN14 |
| MIIP |
| UBE4A |
| ENSG00000290046 |
| PDZD11 |
| KLHL28 |
| STXBP6 |
| INTS4 |
| NLGN1 |
| OFD1 |
| DNAJC1 |
| CACNB2 |
| MLLT6 |
| STAC3 |
| RAD9A |
| ZFAND4 |
| HEG1 |
| PLEKHA8 |
| TEX14 |
| CEP120 |
| ENSG00000290796 |
| ENSG00000285572 |
| APOO |
| IGF1 |
| MECP2 |
| PDCD1LG2 |
| ZBTB40 |
| CDC14B |
| HNRNPK-AS1 |
| IL6R-AS1 |
| LTN1 |
| ALDH9A1 |
| TMEM132C |
| WWTR1 |
| ENSG00000233635 |
| GMPR2 |
| SGO1-AS1 |
| ORC3 |
| HDAC2-AS2 |
| GNPTAB |
| TRAF5 |
| PACS2 |
| MRPS23 |
| IFT20 |
| CR1 |
| POLDIP2 |
| DPH5-DT |
| RRAGC-DT |
| LINC01094 |
| KDM5B |
| PIGBOS1 |
| TRAPPC6A |
| MTG2 |
| WIPF2 |
| SLC39A4 |
| YAF2 |
| PCMTD1 |
| ENSG00000258425 |
| ENSG00000288792 |
| SENP3 |
| ARRDC5 |
| LINC01909 |
| ADGRG2 |
| MSRB1 |
| RNF215 |
| EPM2A-DT |
| ADCK1 |
| LINC02542 |
| LPAR6 |
| LRRC40 |
| MRPL16 |
| RFC3 |
| RGS6 |
| ERMAP |
| LINC02899 |
| CEACAM16-AS1 |
| AMY2B |
| TASOR |
| SMPDL3A |
| FDX2 |
| RSBN1L |
| ENSG00000289691 |
| TRIM38 |
| AGAP12P |
| SIGMAR1 |
| REEP4 |
| FSTL1 |
| TTC12 |
| CENPB |
| NCBP3 |
| CACNB4 |
| IL12RB2 |
| ZNF445 |
| ENSG00000286153 |
| PCIF1 |
| C1QTNF7-AS1 |
| CHIC2 |
| SMC5-DT |
| ENSG00000257178 |
| ZNF717 |
| ABCA5 |
| MGMT |
| CLHC1 |
| ENSG00000291272 |
| U2SURP |
| GRPEL1 |
| MTERF4 |
| DPP9 |
| PTPN13 |
| H1-4 |
| DOK1 |
| NEB |
| LINC00680 |
| MLST8 |
| RFK |
| ENSG00000255476 |
| ZNF277 |
| ADGRA3 |
| ENSG00000235111 |
| NME4 |
| LIN52 |
| BLZF1 |
| AQP7 |
| RAB40C |
| CRK |
| GOLGA8A |
| SYNE2 |
| DDB2 |
| DAPP1 |
| XIAP |
| ENSG00000285921 |
| ENSG00000257239 |
| HTR7 |
| TMEM52B |
| ZBTB44 |
| MARCHF8 |
| CUL2 |
| NXT1 |
| SLC24A1 |
| GNL3L |
| RAI14 |
| EEF1AKMT2 |
| SFI1 |
| ENSG00000253184 |
| VIRMA |
| PEX3 |
| CLMP |
| UGCG |
| PSMD10 |
| AASDH |
| TAGAP |
| ENSG00000289053 |
| TTF2 |
| LINC01772 |
| ENSG00000263709 |
| ENSG00000176593 |
| CRYBG1 |
| SLC35B1 |
| ATL2 |
| FBXO10 |
| TPST1 |
| PSMG1 |
| SLC10A3 |
| ENSG00000254733 |
| SULF1 |
| ABLIM1 |
| ENSG00000266846 |
| NDUFAF5 |
| CELF6 |
| ZNF790 |
| MFSD5 |
| LMTK2 |
| SUN1 |
| CNOT1 |
| CALD1 |
| ZNF90 |
| MAD2L2 |
| ARID5A |
| ARHGAP28 |
| NHLRC3 |
| ZNHIT3 |
| GLRX5 |
| BRD9 |
| FAM215B |
| TRG-AS1 |
| PPP4R3A |
| GLUD1P3 |
| ARL5C |
| LDLRAD3 |
| PIWIL4 |
| ADCY6 |
| NHERF2 |
| OSER1 |
| MORN2 |
| NF2 |
| PDK3 |
| LIMA1 |
| TNKS2 |
| POLN |
| CPSF6 |
| TSGA10 |
| PRR5L |
| WEE2-AS1 |
| ENSG00000286724 |
| GNE |
| MTHFD2 |
| CDK5RAP1 |
| CDCP1 |
| ZNF618 |
| PPP2R2A |
| ZNF594 |
| ENSG00000236986 |
| SNHG7 |
| POC5 |
| ADAMTSL3 |
| PDCD11 |
| ENSG00000291121 |
| PGM2L1 |
| SEC14L1 |
| RYR2 |
| KCTD13 |
| MTFR1 |
| ABCD2 |
| TFDP2 |
| BTBD1 |
| PDGFRA |
| EAF2 |
| PGM5P2 |
| GULP1 |
| ZNF552 |
| PRKAA1 |
| PHF20L1 |
| NUP35 |
| ZNF462 |
| TTF1 |
| FAM118B |
| MYO18A |
| ZDHHC4 |
| NSUN2 |
| ACSS1 |
| SEPSECS |
| ENSG00000266957 |
| STK32B |
| STK39 |
| TBC1D31 |
| MIER1 |
| NFKBIB |
| ACVR1B |
| CCDC102B |
| ADAMTS9-AS2 |
| MRPL15 |
| ZNF345 |
| SNTB2 |
| ANAPC1 |
| KIZ |
| ZNF224 |
| ZNF440 |
| BTBD8 |
| BIRC6-AS2 |
| ANKDD1A |
| ZBTB20-AS5 |
| DERA |
| SMC6 |
| OLA1 |
| ENSG00000287555 |
| LINC01266 |
| VCL |
| PTPRO |
| ZC2HC1A |
| DDX55 |
| ENSG00000257176 |
| UFL1-AS1 |
| EIF4ENIF1 |
| MBTPS1 |
| PHF5A |
| IMMP1L |
| PDGFD |
| ROBO1 |
| STX17 |
| ENSG00000268659 |
| MYLK-AS1 |
| MMP24 |
| TSPYL1 |
| RAMAC |
| MBTPS2 |
| EHD4 |
| NHS |
| ANGPTL2 |
| SLC1A1 |
| SELENOO |
| AHCY |
| MRPL45P2 |
| PFDN4 |
| ANKS1B |
| RBM19 |
| CDC73 |
| ATAD1 |
| CASP10 |
| INTU |
| ENSG00000262580 |
| IPO9-AS1 |
| IFT172 |
| C9 |
| ENSG00000259564 |
| LRRC27 |
| PLXNA4 |
| LAMB1 |
| SIN3B |
| SBNO1 |
| PRPF38B |
| COG7 |
| HERC2P7 |
| TP53BP1 |
| SEMA4D |
| DHFR |
| MALT1 |
| ENSG00000284707 |
| HHEX |
| PPWD1 |
| RNLS |
| ZNF337-AS1 |
| PTPRK |
| SLC35B2 |
| ZNF429 |
| LPIN2 |
| FAM117B |
| MSTO1 |
| PCNT |
| ITGB5 |
| ADM |
| KIF2A |
| ELOA-AS1 |
| ERCC3 |
| ANGEL2 |
| NAV3 |
| ORC4 |
| ODAD3 |
| IER5L |
| AHCTF1 |
| INO80D |
| ANGPT2 |
| ENSG00000234428 |
| DENND6A-AS1 |
| HNRNPLL |
| KCTD3 |
| SLC25A44 |
| LRP5 |
| CSRP1-AS1 |
| ENSG00000274441 |
| WASHC5 |
| SHMT2 |
| HOMER2 |
| FBXL2 |
| MYC |
| STAT6 |
| SLC41A2 |
| MROCKI |
| COL4A1 |
| GALM |
| EGR3 |
| PALB2 |
| HEXD |
| LINC00598 |
| ZNF766 |
| GIMAP6 |
| FARSB |
| SLF1 |
| MLLT3 |
| SLC27A1 |
| ASTN2 |
| CCDC30 |
| TMEM94 |
| ATP11C |
| CEP57L1 |
| RRP7BP |
| SYS1 |
| ZNF519 |
| SEC24B-AS1 |
| ZNF461 |
| SCARB1 |
| NDC1 |
| ENSG00000265477 |
| PCYT1A |
| CCDC200 |
| ZNF587 |
| SNAPC2 |
| RNF34 |
| TRAF3IP1 |
| LINC01239 |
| RRAGB |
| ENSG00000270055 |
| ZC3H14 |
| KCTD17 |
| BBS4 |
| CCDC167 |
| TMEM86A |
| ETFDH |
| PAF1 |
| DCN |
| KIAA1217 |
| ENSG00000285534 |
| DZIP3 |
| PPM1B |
| ANG |
| CMTR1 |
| CRYBB1 |
| EPHA6 |
| ENSG00000259336 |
| NEO1 |
| SFXN5 |
| ETFA |
| OCEL1 |
| LINC00886 |
| ZMAT5 |
| PRELP |
| ALKBH8 |
| VTA1 |
| MTMR8 |
| METAP1D |
| EMG1 |
| CCDC186 |
| ERV3-1 |
| ENSG00000289130 |
| EHBP1L1 |
| MACROD2 |
| DCAF7 |
| LINC01010 |
| CRACR2A |
| MGAT2 |
| CYB5R4 |
| NUP153 |
| DLG5 |
| ENSG00000280383 |
| ZNF862 |
| IFI35 |
| MPV17L2 |
| STPG4 |
| DTWD1 |
| RAD51-AS1 |
| SOCS6 |
| ENSG00000229839 |
| LGR4 |
| UNC5C |
| MRPS17 |
| CCDC59 |
| DCBLD2 |
| ENSG00000290459 |
| FUNDC1 |
| CLCN3 |
| RMDN2 |
| DNAI4 |
| MARF1 |
| NABP2 |
| SMAD5 |
| ZNF276 |
| TM9SF4 |
| SLC25A4 |
| FAM136A |
| NPIPB2 |
| NBPF1 |
| ADAM28 |
| DCAF8 |
| ENSG00000286533 |
| FXYD2 |
| ICMT |
| ARL4C |
| C2orf42 |
| TENM1 |
| FAM110B |
| NCAPD3 |
| NSUN5P1 |
| TMED1 |
| RHOQ-AS1 |
| MEG8 |
| AJAP1 |
| KIF9-AS1 |
| ENSG00000291259 |
| MDP1 |
| THSD7A |
| ID3 |
| RNASE4 |
| ERMP1 |
| HMGXB3 |
| C11orf80 |
| LINC02974 |
| ENSG00000183308 |
| ENSG00000250057 |
| NEK9 |
| UBAP2 |
| TSEN34 |
| PARP6 |
| POMC |
| KRBOX4 |
| ENSG00000289516 |
| UBXN7 |
| ARHGAP42 |
| IFIT2 |
| CES1 |
| IGFBP5 |
| THOC6 |
| CCR5AS |
| ENSG00000272800 |
| PDSS1 |
| MAST4 |
| CDK5 |
| CTC-338M12.4 |
| PCGF1 |
| DIS3L |
| STMN1 |
| TMEM9B-AS1 |
| RMND1 |
| INPP5A |
| STK16 |
| SLC50A1 |
| GET4 |
| CEMIP2 |
| POLR2B |
| MFNG |
| ZC4H2 |
| SLC25A46 |
| ITIH5 |
| MLYCD |
| ENTPD4 |
| RBL2 |
| LINC01482 |
| LHFPL2 |
| TMEM39A |
| TSC2 |
| IRAK4 |
| TIMM50 |
| TBC1D1 |
| NFXL1 |
| PPP2R3A |
| ENSG00000291063 |
| TAMALIN |
| ENSG00000224950 |
| RFXANK |
| RBSN |
| SECISBP2L |
| ADORA3 |
| LRRC63 |
| GPN3 |
| MZT2A |
| ENSG00000285658 |
| ENSG00000226465 |
| PRECSIT |
| ASGR1 |
| POLD3 |
| DNM1L |
| ENSG00000290854 |
| ITGB3BP |
| LRIG2-DT |
| CDKL1 |
| TOPBP1 |
| ENSG00000287510 |
| ADCY2 |
| EXOC5 |
| GSTM1 |
| MCCC2 |
| BOLA2 |
| DMXL1-DT |
| KMT2B |
| TRMT44 |
| ETFRF1 |
| ENSG00000285755 |
| CCDC106 |
| POLH |
| CWF19L1 |
| ZNF326 |
| PDE10A |
| SNED1-AS1 |
| MSH2 |
| ZNF329 |
| TSTD2 |
| MGAT4B |
| ENSG00000285909 |
| TMEM115 |
| APAF1 |
| PUS10 |
| ASH2L |
| ZNF763 |
| ZNF433-AS1 |
| TRIM24 |
| AHI1-DT |
| ENSG00000279175 |
| PLPBP |
| HBEGF |
| ACVR2B |
| PALMD |
| RBMS2 |
| GNGT2 |
| RCBTB1 |
| SLC9A7 |
| ACTR3B |
| KALRN |
| ZNF701 |
| MTBP |
| ILKAP |
| NUMA1 |
| MUC6 |
| IPO8 |
| ACOX3 |
| LANCL2 |
| ALG9 |
| NFKBIE |
| PEF1 |
| CCNT1 |
| ENSG00000261573 |
| OSM |
| ZNF397 |
| EPCAM-DT |
| LETMD1 |
| LYRM4 |
| ZNRF1 |
| SPEF2 |
| CNTN4 |
| ZNF420 |
| ZNF529-AS1 |
| CRY2 |
| TNPO2 |
| PLCB4 |
| SRSF8 |
| IGKV1-5 |
| MAP2K6 |
| ZSWIM8 |
| PPP1R26-AS1 |
| CASP8 |
| R3HCC1 |
| ENSG00000272140 |
| ENSG00000287503 |
| C6orf226 |
| PCBP1-AS1 |
| ZMYM5 |
| LINC00265 |
| SNHG25 |
| MTMR12 |
| PRRX1 |
| ATP6V0E2 |
| SMARCAL1 |
| MRPS31 |
| UBALD2 |
| ENSG00000279738 |
| PPARA |
| JHY |
| CCDC180 |
| FYN |
| CTC1 |
| SLC15A4 |
| GATD1-DT |
| ENSG00000289702 |
| OSBPL6 |
| PMS2P3 |
| LINC02237 |
| ENSG00000291124 |
| ANKRD18DP |
| EPB41L4A-AS1 |
| ISLR |
| SESN1 |
| GLRX2 |
| ZNF484 |
| MRPL50 |
| ZNF234 |
| NUP155 |
| RB1CC1 |
| DLEU7 |
| KIAA1671 |
| IFT122 |
| ERBB4 |
| MTF1 |
| MOSPD3 |
| GOLGA6L10 |
| SCYL2 |
| MICA |
| TTLL4 |
| MUC12 |
| TAB1 |
| POP5 |
| ELP2 |
| PRSS23-AS1 |
| PAAF1 |
| ADAT2 |
| SLC7A2 |
| BAZ2A |
| NIPA1 |
| ZNF737 |
| HHAT |
| SUZ12 |
| GXYLT1 |
| LINC01138 |
| ENSG00000289439 |
| B3GNT5 |
| STEAP4 |
| NBPF14 |
| FAM151B-DT |
| TNNI3K |
| CDC16 |
| ENSG00000290680 |
| ZNF337 |
| TMEM106C |
| PRKAR2A |
| ENO4 |
| DYNC2LI1 |
| RUFY1 |
| ASCC2 |
| TMEM150B |
| YAP1 |
| ZFHX4 |
| PART1 |
| SCART1 |
| TMEM91 |
| SLC52A2 |
| NFYC |
| MIR3142HG |
| ENSG00000267632 |
| LINC02422 |
| RBM44 |
| PLCE1 |
| NUP54 |
| LENG8 |
| SPTBN1 |
| ENSG00000261997 |
| CNTNAP2 |
| CFAP61-AS1 |
| AXIN1 |
| ENSG00000226266 |
| ZNF675 |
| ZRANB2-DT |
| PHF8 |
| SLC9B1 |
| CHTF8 |
| MFAP3 |
| COG4 |
| NUP93 |
| WASH6P |
| MTFP1 |
| MYCBP2-AS1 |
| LHFPL6 |
| MALRD1 |
| NT5C3A |
| COMTD1 |
| DDX50 |
| ZNF761 |
| AVIL |
| STPG2 |
| G6PD |
| INTS10 |
| ZBTB34 |
| CHROMR |
| LINC00571 |
| B4GALT7 |
| METTL2B |
| CEP83 |
| PAXIP1 |
| ENSG00000259118 |
| GRIK2 |
| WDR18 |
| TULP3 |
| ICAM4 |
| LARP1B |
| PWWP2A |
| PCNA |
| MAGED1 |
| TEX10 |
| BMAL1 |
| ODR4 |
| RINL |
| CKAP5 |
| GEM |
| EDEM3 |
| PSMD5 |
| ENPP1 |
| ZNF267 |
| TEX41 |
| MRPS30-DT |
| NOL8 |
| UGDH-AS1 |
| ENSG00000232855 |
| USP3-AS1 |
| EFCAB7 |
| APOBEC3C |
| CDKN1C |
| USP13 |
| HCCS |
| TSPAN31 |
| GOSR2 |
| ZNF175 |
| C3orf14 |
| ACSM6 |
| LRRC2 |
| COL4A2 |
| ZFP30 |
| SLC39A14 |
| TSPAN14 |
| TBX1 |
| MGAT4C |
| BRCA1 |
| LINC01605 |
| ENSG00000266049 |
| GON7 |
| PPIF |
| SCARA5 |
| ALG11 |
| N4BP2L1 |
| CEP295NL |
| ZGRF1 |
| PKP4 |
| GPSM2 |
| ELN |
| ZNF641 |
| SPTLC3 |
| TP53RK |
| PTRH1 |
| SLC13A3 |
| LINC00907 |
| SDHAF1 |
| ENSG00000268112 |
| ZCWPW2 |
| LINC01355 |
| SLC33A1 |
| SLC25A36 |
| INTS3 |
| AMZ2P1 |
| TRAPPC2B |
| MREG |
| FOSL1 |
| PAG1 |
| HIBADH |
| ENSG00000286803 |
| ENSG00000248752 |
| ARHGAP27 |
| POC1B-AS1 |
| ACOT2 |
| PINK1 |
| ANKRD10-IT1 |
| ZC3HC1 |
| TMEM101 |
| EGFR |
| PRADC1 |
| WDR36 |
| PLPP3 |
| EFTUD2 |
| OSBPL2 |
| FRRS1 |
| RBM45 |
| PRDM15 |
| ROR1 |
| GFPT2 |
| ATP6V1A |
| RPL34-DT |
| CASTOR3P |
| DNAI3 |
| SGPL1 |
| NPHP4 |
| NSF |
| SMAD3 |
| TMEM67 |
| WDR12 |
| ZNF496 |
| CDH13 |
| LANCL1 |
| ZNF84 |
| SP2-AS1 |
| ENSG00000223732 |
| NOL9 |
| MRC2 |
| UBA3 |
| EOLA1-DT |
| GTF3C3 |
| DBT |
| ENSG00000230773 |
| PPM1H |
| ENSG00000232611 |
| ENSG00000280145 |
| TMEM14A |
| RNF214 |
| PLSCR4 |
| ENSG00000253853 |
| MFSD11 |
| ASTL |
| ENSG00000268362 |
| ENSG00000286681 |
| SCARA3 |
| KCNIP2 |
| ZDHHC7 |
| PTPN22 |
| MPDZ |
| NTN1 |
| KIN |
| ENSG00000291066 |
| CCDC65 |
| PARL |
| ANKRD37 |
| CFAP410 |
| GGA2 |
| SMPD1 |
| LIN54 |
| IL10RB-DT |
| NDE1 |
| GEMIN7-AS1 |
| YARS2 |
| GTF2IRD2 |
| ENSG00000249001 |
| ACP3 |
| POP1 |
| SERAC1 |
| RAC2 |
| MX2 |
| FAM114A1 |
| KIFAP3 |
| DNAAF4 |
| ZNF500 |
| THAP11 |
| CDKL5 |
| NCAM2 |
| POLR2C |
| SOX6 |
| AK9 |
| NACC2 |
| YJU2B |
| TRAK2 |
| HERPUD2 |
| ARMCX6 |
| ISCA1 |
| ADD3 |
| ENSG00000290183 |
| TST |
| LINC01088 |
| SMPD4 |
| PREPL |
| HOXB2 |
| CHST15 |
| ENSG00000289070 |
| CC2D2A |
| ENSG00000286481 |
| ENSG00000289463 |
| NAT10 |
| AGO2 |
| ACCS |
| ZNF790-AS1 |
| MCPH1-AS1 |
| EML1 |
| LINC01237 |
| ENSG00000287684 |
| KCNQ5 |
| ENSG00000187229 |
| THAP7 |
| RNF32-DT |
| HBS1L |
| ROR2 |
| IGLV3-21 |
| TSHZ3-AS1 |
| DOCK6 |
| PTGFR |
| ENSG00000251680 |
| NBPF10 |
| LIPE |
| PAK4 |
| NDUFAF7 |
| PDIA5 |
| MRPS2 |
| ZNF622 |
| CCDC85A |
| ENSG00000234185 |
| MYO5B |
| KYAT3 |
| CDC7 |
| PSMB8-AS1 |
| ENSG00000291055 |
| MRPL58 |
| LTBP1 |
| AGPAT1 |
| CCDC6 |
| SARM1 |
| PDZRN3 |
| GLE1 |
| THAP6 |
| ENSG00000289405 |
| FYCO1 |
| DNAJB4 |
| DPH1 |
| TNRC6C |
| TBX19 |
| GAN |
| OSBPL5 |
| ZFYVE9 |
| NKAIN2 |
| APP |
| KLHL29 |
| ING2 |
| RENO1 |
| N4BP2L2-IT2 |
| ENSG00000249456 |
| NOM1 |
| UPF3A |
| ENSG00000260517 |
| HS1BP3 |
| INPP4B |
| GALC |
| PEX7 |
| ZCCHC4 |
| LINC00513 |
| AKAP17A |
| EYS |
| ENSG00000288969 |
| CLIC5 |
| VRK3 |
| LPAR5 |
| GATB |
| RIMKLB |
| ADTRP |
| PLEKHH1 |
| ALG13-AS1 |
| ZNF100 |
| PAXBP1-AS1 |
| FUBP1 |
| DPF3 |
| VRK1 |
| SLC35A4 |
| TOP2B |
| MSH5 |
| MEGF9 |
| ZNF341 |
| IQCH-AS1 |
| CNNM2 |
| IQCK |
| ENSG00000290018 |
| FREM1 |
| TRPT1 |
| KPNB1-DT |
| ZC3H8 |
| ENSG00000225300 |
| INPP5F |
| CTDP1 |
| LMAN2L |
| STAMBPL1 |
| ENSG00000236540 |
| ZNF302 |
| BRF1 |
| ENSG00000291042 |
| SETDB2 |
| ELK3 |
| ENSG00000289504 |
| ENSG00000225311 |
| COL24A1 |
| SGCD |
| LMO7 |
| ZNF16 |
| LRRC4C |
| ENSG00000286071 |
| TXNRD3 |
| ENSG00000231563 |
| ENSG00000289341 |
| RSU1 |
| SLIT3 |
| PAPOLG |
| PHF2 |
| RARS1 |
| MAGEF1 |
| ENSG00000224356 |
| ZNF320 |
| STXBP3 |
| FBXO46 |
| MTG1 |
| CRYGS |
| ENSG00000258181 |
| PEX26 |
| CTNNBL1 |
| ZNF70 |
| SNCA |
| TSC22D1 |
| RIPK1 |
| CDH11 |
| FLG-AS1 |
| HK1 |
| MADD |
| ENSG00000291132 |
| RBFOX1 |
| DHX8 |
| RNF2 |
| SRFBP1 |
| USP37 |
| MYZAP |
| SLC37A3 |
| SLIT2 |
| FAT4 |
| DAP3 |
| ENSG00000273486 |
| ENSG00000289340 |
| PID1 |
| PEX13 |
| ST6GALNAC4 |
| TMEM107 |
| ATG16L1 |
| ENSG00000288025 |
| APOOL |
| HSD17B7P2 |
| MRPL9 |
| ENSG00000286125 |
| TEX2 |
| RAB24 |
| DUSP5 |
| ZPR1 |
| HISLA |
| POLA2 |
| OTOA |
| SINHCAF |
| CEP104 |
| ENSG00000286271 |
| ZNF626 |
| AK6 |
| ANKRD23 |
| OCRL |
| TUBGCP4 |
| UBL4A |
| GNB4 |
| CSE1L |
| SIK1 |
| RNF113A |
| SEZ6L |
| CASS4 |
| ENSG00000226526 |
| NOTCH2NLA |
| ENSG00000289528 |
| YY1AP1 |
| WDR17 |
| ENSG00000267383 |
| ANKLE2 |
| UBOX5 |
| SECTM1 |
| CARMN |
| TTC21A |
| SNX25 |
| SLC19A3 |
| DTYMK |
| ODC1 |
| SUGCT |
| FKBP1B |
| TUG1 |
| ENSG00000227355 |
| ADAR |
| TAF5L |
| SP2-DT |
| RTN4RL1 |
| FIGN |
| FBRSL1 |
| LY6E |
| NRSN2-AS1 |
| ARID3B |
| ZNF138 |
| ENTPD5 |
| SLC35D1 |
| ENSG00000287077 |
| STIMATE |
| PELI2 |
| GTF2H1 |
| TSPOAP1-AS1 |
| ENSG00000274213 |
| CD247 |
| LINC01762 |
| ENSG00000227227 |
| PFKFB4 |
| MRPS31P5 |
| BORCS6 |
| RNF4 |
| ZNF529 |
| USP46 |
| COL5A1 |
| FADD |
| ENSG00000224848 |
| GRIPAP1 |
| SLX9 |
| ADGRL2 |
| PLAA |
| UBXN2B |
| MMP2-AS1 |
| ARHGAP30 |
| ENSG00000228010 |
| TACC2 |
| TCEANC2 |
| OSMR |
| SENP2 |
| DNM3OS |
| MKNK1-AS1 |
| S1PR1 |
| PRPS1 |
| AKAP8 |
| ATG16L2 |
| FIRRE |
| INSL6 |
| SUCO |
| HDHD2 |
| ENSG00000286277 |
| EFCAB2 |
| SCLY |
| HIPK2 |
| XPC |
| EARS2 |
| NTN4 |
| ING1 |
| RPGR |
| RNF168 |
| KCNMB3 |
| FLVCR1 |
| LINC02340 |
| HDDC3 |
| APOL3 |
| DNAJC17 |
| SPRY3 |
| LINC01572 |
| STS |
| ARL17B |
| L2HGDH |
| LRP6 |
| HERC6 |
| MIR3945HG |
| TM7SF3 |
| FHIP2A |
| ENSG00000273674 |
| CENPJ |
| GBP2 |
| ENSG00000286121 |
| CALN1 |
| BLOC1S4 |
| CDC34 |
| ESF1 |
| ENSG00000275119 |
| TPRG1L |
| OXLD1 |
| BTN2A3P |
| TCP10L |
| PRICKLE1 |
| ST6GALNAC3 |
| TRAF7 |
| EFHC1 |
| BEAN1 |
| SEPTIN11 |
| NOTCH1 |
| LSP1 |
| HRH2 |
| IL1RN |
| EFCAB6 |
| ENSG00000289698 |
| SF3B3 |
| IRAG2 |
| ADRB2 |
| GRAMD2B |
| EFCAB11 |
| KDM4A |
| WDR89 |
| UBN1 |
| ADAMTS10 |
| LENG8-AS1 |
| AGBL1 |
| PITPNM2 |
| WDR75 |
| TRIM25 |
| ENSG00000273507 |
| KLHDC4 |
| ENSG00000273295 |
| DHDH |
| ENSG00000276649 |
| CCDC136 |
| GIN1 |
| BSDC1 |
| ZUP1 |
| ZNF564 |
| P2RY12 |
| ZNF282 |
| MIDEAS |
| ENSG00000272807 |
| ZNF594-DT |
| AATF |
| LRP12 |
| ENSG00000254242 |
| RAD18 |
| ENSG00000288075 |
| MSC-AS1 |
| PRKAR2B |
| SH3GL1 |
| NBPF15 |
| TEAD1 |
| LINS1 |
| RAB2B |
| KCTD1 |
| SFXN2 |
| FAM111A |
| RMND5B |
| TTC8 |
| PDXP-DT |
| JMJD8 |
| RPS6KB1 |
| ENSG00000271009 |
| ZRSR2 |
| MLIP |
| EFEMP1 |
| NBPF20 |
| PPP1R3E |
| ENSG00000288302 |
| ENSG00000290007 |
| ADAMTS5 |
| MAX |
| RBPMS |
| ENSG00000290396 |
| ENSG00000233367 |
| PTPRB |
| LINC02705 |
| DDX3Y |
| LMNB2 |
| ZNF569 |
| UBOX5-AS1 |
| EBP |
| ENSG00000285664 |
| LUCAT1 |
| RECK |
| NMNAT1 |
| TTC28-AS1 |
| AHDC1 |
| CORO7 |
| ENSG00000286458 |
| RAB42 |
| SAFB2 |
| LSAMP |
| GBA2 |
| MRPL35 |
| WDR25 |
| DLGAP1 |
| FAT2 |
| B3GNTL1 |
| WDR73 |
| KATNAL2 |
| BORCS5 |
| ARHGEF37 |
| ENSG00000291073 |
| ENSG00000258561 |
| F8A1 |
| ABT1 |
| ENSG00000237813 |
| AP4S1 |
| ZNF791 |
| SLC30A5 |
| VPS33A |
| XPNPEP1 |
| ZNF839 |
| HMCN1 |
| ENSG00000247228 |
| CLPB |
| BTBD3 |
| ENSG00000261684 |
| ADCY10P1 |
| ENSG00000267199 |
| HDX |
| TATDN3 |
| MTX2 |
| DPY19L1 |
| PROSER3 |
| NTM |
| CAMSAP1-DT |
| POU6F1 |
| CP |
| TTC19 |
| ENSG00000253392 |
| STK35 |
| ABCA8 |
| ENSG00000228427 |
| ZBTB49 |
| CXCL1 |
| WARS2 |
| ZNF7 |
| UBE3B |
| RBM27 |
| MAPK9 |
| ZW10 |
| GFM1 |
| AQP1 |
| SPRY4-AS1 |
| TNFRSF10A |
| GIGYF1 |
| PRKD1 |
| ENSG00000288016 |
| MFSD14A |
| HHIP |
| OVOL2 |
| NYAP2 |
| TBC1D23 |
| TESK2 |
| DHX9-AS1 |
| NCBP1 |
| G2E3-AS1 |
| IMMT |
| MDM2 |
| SNHG15 |
| ENSG00000289376 |
| LRP8 |
| PAPLN |
| NSMCE3 |
| CDKN2AIP |
| ZNF460-AS1 |
| PXDN |
| ENSG00000283103 |
| SHISA4 |
| ZNF589 |
| ZNF264 |
| ENSG00000244459 |
| EMID1 |
| RASSF1 |
| CDK2AP1 |
| DNAJC27 |
| NAB1 |
| PXK |
| FAM156A |
| FGF10-AS1 |
| TRMU |
| LTO1 |
| SAFB |
| GPATCH1 |
| CLDN14 |
| ARMC9 |
| GSTT2 |
| ARL6IP6 |
| DELEC1 |
| ZC3H12B |
| ENSG00000291130 |
| HOXB5 |
| NIPAL3 |
| ATF7IP2 |
| PRDM11 |
| WDR5B-DT |
| NUDT16L1 |
| FAM168B |
| MIR222HG |
| NIFK-AS1 |
| PKD2L2-DT |
| ENSG00000267002 |
| SETD3 |
| ARHGAP11B-DT |
| CMC1 |
| MAGI3 |
| SLAIN2 |
| LINC01645 |
| KDM1B |
| TRIM23 |
| MYPN |
| ENSG00000291065 |
| ZNF546 |
| SETD4 |
| RSAD2 |
| MACO1 |
| CNOT6L |
| GRIK1 |
| LTBP3 |
| EXOSC5 |
| POLE2 |
| ZNF432 |
| ENSG00000290039 |
| TCF7L1 |
| TLNRD1 |
| ENSG00000272316 |
| SULT1A3 |
| ZNF146 |
| PEX1 |
| ESRRA |
| CYTH3 |
| NDUFAF4 |
| ARHGAP8 |
| ADGRL1-AS1 |
| JAK3 |
| NOB1 |
| PIK3IP1-DT |
| ZNF608 |
| OTUD7A |
| MEIS2 |
| LINC00506 |
| TRIM22 |
| MEF2C-AS2 |
| ENSG00000254363 |
| ANKRD52 |
| ZNF142 |
| CAPN15 |
| ZC3H18 |
| ENSG00000227033 |
| ADGRE2 |
| ENSG00000278384 |
| ENSG00000287720 |
| ATP6V0A2 |
| GART |
| AARS2 |
| FMO4 |
| MAST3 |
| MT1G |
| TMEM9 |
| RINT1 |
| POU5F2 |
| TUBG1 |
| ZNF783 |
| ENSG00000225751 |
| GIMAP8 |
| ENSG00000291220 |
| CDKN2AIPNL |
| KDM5C |
| NBPF8 |
| ANXA2R-OT1 |
| ENSG00000267764 |
| PTGIR |
| CYP1B1-AS1 |
| ENSG00000274383 |
| SFXN4 |
| QTRT2 |
| USP38-DT |
| NAV2-IT1 |
| ALDH18A1 |
| BANK1 |
| ADAMTS17 |
| ZNF544 |
| TOR1A |
| TMEM203 |
| RHNO1 |
| GEMIN6 |
| ENSG00000259616 |
| VSIG10 |
| CCNT2-AS1 |
| SPATA9 |
| SYCP2L |
| HRAS |
| NAP1L4 |
| ENSG00000290067 |
| TEX261 |
| SKAP1 |
| HSD17B8 |
| RASGEF1C |
| CEACAM19 |
| ENSG00000259865 |
| QPCT |
| IPPK |
| TAFA2 |
| STEAP2 |
| PTPRF |
| PPP4R4 |
| IL21R |
| POLI |
| FASTKD2 |
| ENSG00000258891 |
| RALGPS2 |
| LINC00472 |
| CTNNA3 |
| NSUN7 |
| RTL8B |
| PNMA1 |
| SLC35C1 |
| RPP25L |
| CEACAM22P |
| TAS2R4 |
| CKS1B |
| ZNF670 |
| CPVL-AS2 |
| DIRC3-AS1 |
| ENSG00000257252 |
| MIPOL1 |
| GTF2IRD2B |
| ETV5 |
| APOC2 |
| CLCC1 |
| AFAP1 |
| SNHG20 |
| NUP85 |
| PTCD2 |
| VWF |
| LNX2 |
| MYO10 |
| WDR4 |
| CFAP68 |
| NOP16 |
| ADAMTS2 |
| RBM15B |
| HOXB-AS1 |
| PWWP3A |
| NEGR1 |
| PLEKHG3 |
| ENSG00000228107 |
| PIK3R4 |
| ZBTB41 |
| ZNF25 |
| ARMCX1 |
| ANK2-AS1 |
| ENSG00000263990 |
| ENSG00000261267 |
| FBXO17 |
| ACAT2 |
| ZNF567 |
| BBS2 |
| MR1 |
| ENSG00000271789 |
| NDOR1 |
| SMG5 |
| ZNF624 |
| CFAP418-AS1 |
| ZC3H13 |
| ACBD3 |
| UQCC4 |
| MRPL10 |
| ENSG00000291099 |
| SGCE |
| ENSG00000259589 |
| KCNMA1 |
| FLYWCH1 |
| TEX9 |
| ENSG00000288923 |
| PHKA2 |
| ATP9A |
| TCP11L2 |
| ZNF280C |
| RAMP2 |
| NINL |
| ENSG00000233178 |
| NRM |
| ENSG00000286074 |
| SLC35A1 |
| DDAH1 |
| ENSG00000225313 |
| ENSG00000289557 |
| ZNF682 |
| CCNO-DT |
| UBR4 |
| PYROXD1 |
| TCHP |
| LINC01118 |
| DUS2 |
| RNF185 |
| RTN4IP1 |
| ISM1 |
| IL6 |
| C1QTNF7 |
| KNTC1 |
| LINC02766 |
| MED12L |
| GALNT15 |
| LMBRD2 |
| TCTN3 |
| CATSPERE |
| FILIP1 |
| MT1F |
| ITGB5-AS1 |
| RARRES1 |
| ENSG00000225689 |
| RBM14 |
| ZNF451-AS1 |
| SGIP1 |
| ENSG00000268205 |
| EYA4 |
| DCLK2 |
| ZDHHC12-DT |
| ENSG00000272831 |
| SATB2 |
| TAFAZZIN |
| B3GLCT |
| LEMD2 |
| LIFR |
| ENSG00000239415 |
| EPC1-AS1 |
| MAK |
| ATP8A1 |
| MMGT1 |
| BORA |
| FAM76B |
| MARK1 |
| GOPC |
| ENSG00000230826 |
| TOP3A |
| ENSG00000229321 |
| FBXO38-DT |
| PMM1 |
| FKBP14-AS1 |
| ENSG00000253988 |
| SLC25A40 |
| LINC01924 |
| ZSCAN25 |
| ENPP2 |
| LINC01965 |
| INTS2 |
| ENSG00000290856 |
| ENSG00000228061 |
| ZNF738 |
| NUDCD1 |
| TTC39B |
| CIDEA |
| MYO6 |
| SLC24A3 |
| TP53INP2 |
| THTPA |
| DNM3 |
| HSD17B3 |
| NFRKB |
| WNT5B |
| MTRF1 |
| SLC35A2 |
| ENSG00000270871 |
| OSMR-DT |
| TMEM119 |
| ENSG00000289315 |
| LIPN |
| LAMC1 |
| ENSG00000261669 |
| CERS5 |
| ARHGAP19 |
| DBP |
| PHLPP2 |
| ENSG00000205740 |
| SH3BP5-AS1 |
| ENSG00000263708 |
| SLC2A6 |
| DYNC1I1 |
| AP3D1 |
| CES1P1 |
| RGPD8 |
| PRKACB |
| TMOD2 |
| ADIPOQ |
| ENSG00000287292 |
| URB2 |
| CFI |
| ZNF212 |
| CDKN2C |
| SH3YL1 |
| GFOD2 |
| ATG4B |
| LBX2-AS1 |
| ENSG00000230732 |
| MMP1 |
| ENSG00000253347 |
| ENSG00000272219 |
| ENSG00000283118 |
| COL15A1 |
| CELSR1 |
| ING2-DT |
| ENSG00000242861 |
| NRXN3 |
| ZBTB16 |
| NEDD4L |
| ENSG00000226310 |
| TOR4A |
| LNX1 |
| BCR |
| KLHL12 |
| ENSG00000289956 |
| C4orf19 |
| KDM4A-AS1 |
| SNX4 |
| FBXO41 |
| ARHGAP20 |
| PNPLA7 |
| CCNJ |
| ZNF347 |
| ZNF425 |
| ZNF703 |
| MRPL46 |
| GCN1 |
| LNCAROD |
| ENSG00000288700 |
| AEBP1 |
| CCN2 |
| CYP2U1-AS1 |
| ZNF383 |
| TMEM143 |
| ENSG00000254263 |
| SCAMP5 |
| MSS51 |
| TSHZ1 |
| SFXN1 |
| VIPAS39 |
| APPBP2-DT |
| USF3 |
| LINC00639 |
| ENSG00000276345 |
| TBC1D22A-DT |
| ZNF180 |
| VPS26C |
| KYAT1 |
| MITD1 |
| PCOLCE |
| CBR3-AS1 |
| ACO1 |
| ACSS2 |
| ATXN1L |
| FAN1 |
| WDR19 |
| USP20 |
| TRPC1 |
| GOT1-DT |
| FBXO36 |
| ENSG00000259124 |
| CCDC15 |
| THSD4 |
| ENSG00000279159 |
| ENSG00000287937 |
| PPP2R1B |
| NRGN |
| ENSG00000290886 |
| LRRC37A |
| WWC3 |
| ENSG00000278390 |
| FRA10AC1 |
| ZNF136 |
| SCAP |
| ALYREF |
| ITGA1 |
| SOWAHD |
| CD80 |
| ENSG00000262151 |
| FAP |
| HORMAD1 |
| PLOD2 |
| IGKV3-11 |
| TULP2 |
| MAILR |
| LINC01060 |
| ANKRD39 |
| TNFRSF12A |
| CCDC142 |
| ERAP2 |
| MORC2 |
| UBXN2A |
| CPTP |
| C21orf62-AS1 |
| GOLGA8B |
| STXBP1 |
| CLDN14-AS1 |
| ESRRB |
| ENSG00000272356 |
| RARA-AS1 |
| TIA1 |
| TSPAN5 |
| LINC02246 |
| COPG2IT1 |
| NEK4 |
| ASAP3 |
| AP3M2 |
| BGN |
| ENSG00000268056 |
| ADH1B |
| KCTD7 |
| C15orf48 |
| ZSWIM5 |
| SH2D3C |
| MYBPC3 |
| MCM3AP-AS1 |
| ZFP82 |
| WDR48 |
| STAM |
| ENSG00000249771 |
| THSD4-AS1 |
| ADAM7-AS1 |
| PPP2R5A |
| ZNG1C |
| NEK10 |
| ZNF814 |
| SOCS1 |
| ZBTB8A |
| ERCC6 |
| MTMR4 |
| SGSM2 |
| DNAL4 |
| AGAP4 |
| GTF2E2 |
| FOXP1-IT1 |
| APRG1 |
| SLC12A7 |
| ENSG00000267461 |
| HTRA4 |
| SPIN3 |
| JRK |
| TRIM16 |
| ENSG00000284738 |
| ULK1 |
| PTH2R |
| RSPH3 |
| MGME1 |
| PGAP1 |
| SACS |
| COX15 |
| CLTCL1 |
| SLC39A9 |
| IGHMBP2 |
| MFN1 |
| CPSF7 |
| B3GALT1 |
| DENND2A |
| LINC01358 |
| PRKCI |
| ENSG00000255240 |
| ARHGAP29 |
| RNF125 |
| CHI3L1 |
| ZNF630 |
| CCDC71 |
| ARL10 |
| STXBP5-AS1 |
| INSYN2A |
| GYS1 |
| ENSG00000287665 |
| MINDY4 |
| ZNF273 |
| ENSG00000262370 |
| TLL1 |
| CEP85 |
| ACOD1 |
| SAC3D1 |
| BCL2L13 |
| DFFB |
| SPIRE2 |
| NUDT18 |
| ASPHD1 |
| ACD |
| VPS33B |
| TRIM5 |
| RCHY1 |
| ENSG00000257452 |
| COL8A1 |
| ZNF850 |
| SLC25A42 |
| GABPB1-AS1 |
| CLK2 |
| COPS7B |
| LINC02930 |
| TMEM232 |
| IL1RAPL1 |
| ZNF121 |
| PINX1 |
| C17orf58 |
| GABRE |
| IL16 |
| NMT2 |
| SORCS1 |
| RPUSD1 |
| BVES-AS1 |
| TEX22 |
| GPAM |
| SPRYD3 |
| ICA1L |
| GTF2H2C |
| TOM1L1 |
| PRX |
| ENSG00000230490 |
| ZNF202 |
| TPTEP1 |
| ZNF709 |
| ENSG00000231731 |
| ENSG00000291143 |
| HACD2 |
| EIF3J-DT |
| RASGRF2 |
| ENSG00000290951 |
| TMEM51-AS2 |
| SEC61A2 |
| CCN3 |
| ENSG00000243004 |
| RNASEH2B-AS1 |
| ENSG00000246851 |
| NDRG2 |
| HPS4 |
| TNS2-AS1 |
| UBAP1L |
| CMTM4 |
| ZFP41 |
| NPIPB4 |
| NMNAT3 |
| TRPV4 |
| ZNF76 |
| CYSLTR2 |
| ZCCHC8 |
| CENPW |
| EIF1AY |
| SLC26A5 |
| LINC02240 |
| ENSG00000286808 |
| GKAP1 |
| EVA1A-AS |
| C10orf88 |
| LINC02976 |
| CARD11 |
| TMTC1 |
| SLFNL1-AS1 |
| WDHD1 |
| LINC01500 |
| PRKD2 |
| UCK1 |
| ENSG00000287085 |
| ENSG00000257027 |
| PPIP5K1 |
| EPM2AIP1 |
| C11orf71 |
| CAD |
| BCO2 |
| LIMK1 |
| KLHL23 |
| CETN2 |
| MSL1 |
| POMK |
| OTUD3 |
| PNISR-AS1 |
| ENSG00000253607 |
| EBF2 |
| ENSG00000260167 |
| LINC02193 |
| ZNF551 |
| OTUD7B |
| GHRLOS |
| KCND3 |
| EDDM13 |
| UTP11 |
| PRKAG2-AS1 |
| HMGA2 |
| C6orf47 |
| TRHDE-AS1 |
| TIAL1 |
| ENSG00000287807 |
| FHIP1B |
| GSDMB |
| ZNF3 |
| ZNF112 |
| TRAPPC6B |
| GNA14 |
| GSTM4 |
| LINC00862 |
| GYG2 |
| LINC00968 |
| C12orf40 |
| ACY1 |
| SEL1L3 |
| ZNF514 |
| DZANK1 |
| KLHL24 |
| CD44-AS1 |
| SLC4A5 |
| TMEM170B |
| BAHCC1 |
| DOCK4-AS1 |
| MAP3K20-AS1 |
| ENSG00000286512 |
| HEXD-IT1 |
| ENSG00000286480 |
| ETS1 |
| MEIS1 |
| ABCA10 |
| BBS1 |
| ASCL2 |
| RMDN1 |
| ECD |
| ZNF283 |
| NUAK1 |
| YDJC |
| PRUNE2 |
| PNPO |
| ERI1 |
| SHANK2 |
| SND1-IT1 |
| DCAF16 |
| CUL9 |
| RORA-AS1 |
| NLRC4 |
| IGKV1-9 |
| ENSG00000279342 |
| LRRC7 |
| BMAL2 |
| ZCCHC24 |
| LINC01411 |
| IFT81 |
| TRIM26 |
| ENSG00000241220 |
| TRIM36 |
| ENSG00000287216 |
| CCL13 |
| NR2F2-AS1 |
| BRAP |
| CHN1 |
| GFOD1 |
| ITGA4 |
| SLC7A5 |
| FZD1 |
| LINC01013 |
| ECT2 |
| CD7 |
| PPP2R3C |
| PLIN5 |
| RDH5 |
| MRPL44 |
| ENSG00000289437 |
| ZNF45 |
| ENSG00000288899 |
| TBX18 |
| ALG8 |
| TRIM16L |
| CH25H |
| ZNF417 |
| MAN2C1 |
| SLC1A5 |
| MLH1 |
| ENSG00000271945 |
| SYTL3 |
| ZEB1-AS1 |
| VEGFA |
| CARNMT1 |
| DDHD2 |
| ENSG00000287421 |
| PROCA1 |
| ENSG00000260877 |
| MTPAP |
| LINC02356 |
| ITIH4 |
| ENSG00000232828 |
| KLHL18 |
| CEP44 |
| SPATA5L1 |
| IFT22 |
| METTL14 |
| ZNF423 |
| TRIOBP |
| PKD1L2 |
| ANGPT1 |
| LY6G5B |
| KCTD9 |
| AKNA |
| TYK2 |
| NTHL1 |
| TSPAN9 |
| MIR193BHG |
| CPNE4 |
| OPCML |
| SLC30A4 |
| RAB43 |
| ARHGEF28 |
| DNAAF10 |
| AADACL2-AS1 |
| SEMA3E |
| MOCOS |
| LINC02018 |
| RBP7 |
| LINC01719 |
| AGL |
| DLGAP4-AS1 |
| SLC16A4 |
| ENSG00000234915 |
| SHROOM4 |
| LINC02384 |
| PTTG1 |
| ENSG00000289600 |
| NIBAN1 |
| KLF8 |
| PVR |
| ENSG00000231128 |
| LPCAT3 |
| PLEKHG6 |
| XACT |
| TMEM250 |
| ABHD6 |
| ACAD9 |
| MID1 |
| FBXL13 |
| OXR1-AS1 |
| STARD13-AS |
| CMC4 |
| MEX3A |
| TP53 |
| ENSG00000289180 |
| MYH11 |
| N4BP2 |
| GABRB1 |
| EMC9 |
| RCE1 |
| ENSG00000261098 |
| TTI2 |
| GABRA4 |
| PPM1F |
| TRERF1 |
| PCF11-AS1 |
| KCNIP4 |
| FAM131B-AS2 |
| LIN9 |
| TRPM3 |
| FBXW2 |
| HELB |
| FBXL18 |
| BCL2L12 |
| ZNF713 |
| FAM20B |
| ANKRD9 |
| DLG2-AS2 |
| GABPB1 |
| ENSG00000261472 |
| PML |
| ENSG00000257279 |
| HSDL2 |
| LINC01285 |
| NISCH |
| ANKMY2 |
| ENSG00000282917 |
| SMIM27 |
| IFIH1 |
| RETREG3 |
| BRD8 |
| ABCA3 |
| TGFBRAP1 |
| ENSG00000255968 |
| NID2 |
| PDE1C |
| LPIN3 |
| MYH10 |
| CPB2-AS1 |
| ZNF215 |
| DNAJC14 |
| CLSTN2 |
| WDTC1 |
| ENSG00000241577 |
| ILRUN-AS1 |
| ST7 |
| PLS3 |
| PGBD1 |
| PLK2 |
| STYXL2 |
| CCDC3 |
| PUSL1 |
| GMPPB |
| ENSG00000286503 |
| KLHL42 |
| ZNF768 |
| PRKCG |
| TMEM139-AS1 |
| RPS6KL1 |
| SPAG17 |
| ENSG00000271554 |
| ENSG00000226849 |
| ZDHHC11B |
| ENSG00000285669 |
| ENSG00000286887 |
| HEATR6 |
| ENSG00000250764 |
| AMN1 |
| BOC |
| PSD4 |
| MRI1 |
| ENSG00000229751 |
| CLDN23 |
| ENSG00000270072 |
| MINDY2 |
| ZNF30 |
| GEMIN5 |
| CXorf38 |
| LINC00205 |
| C5orf34 |
| ANKRD46 |
| BIRC3 |
| FSIP2-AS1 |
| LINC01876 |
| SOBP |
| ENSG00000283415 |
| ENSG00000258378 |
| H1-2 |
| SPINT1 |
| TMEM220-AS1 |
| LINC01814 |
| C6 |
| EVC |
| TMEM163 |
| ERG28 |
| C1RL-AS1 |
| HYMAI |
| TRAM2 |
| DELE1 |
| LINC00899 |
| ENSG00000259201 |
| HTRA2 |
| MAGEH1 |
| NHSL2 |
| LNC-LBCS |
| ZNF343 |
| AMOTL1 |
| ZNF549 |
| AREG |
| ENSG00000291046 |
| FANCA |
| PCMTD1-DT |
| VWA2 |
| ADAMTS16 |
| ENSG00000248758 |
| RFLNA |
| LINC02133 |
| KRBA2 |
| C8orf82 |
| MTO1 |
| SAMD5 |
| F5 |
| USP50 |
| CACNA1D |
| OMG |
| ENSG00000225092 |
| C19orf12 |
| SMIM15-AS1 |
| ENSG00000250397 |
| SCN11A |
| ENSG00000261632 |
| ENSG00000267474 |
| PATL2 |
| BTN3A1 |
| IL20RB |
| ENSG00000291224 |
| DDX19A-DT |
| ZNF778 |
| SREBF1 |
| ENSG00000260378 |
| UBA2 |
| CACNA1A |
| HPCAL1 |
| CTPS1 |
| RTEL1 |
| CRB1 |
| NOL4L |
| LINC01036 |
| ENSG00000288107 |
| ITPR3 |
| CATSPER1 |
| PCMTD2 |
| NFKB1 |
| KIF26B |
| CAAP1 |
| AMMECR1L |
| VIPR1 |
| KLC1-AS1 |
| VAV3-AS1 |
| ENSG00000287149 |
| PABIR1 |
| HDGFL3 |
| BIN3 |
| CFAP91 |
| DAGLA |
| KCND2 |
| NSUN4 |
| CCDC77 |
| ZNF561 |
| EDC3 |
| PNPLA4 |
| SGSM1 |
| ENSG00000286872 |
| AIG1 |
| ERP27 |
| DOHH |
| ENSG00000271254 |
| ANKRD50 |
| ENSG00000264112 |
| PXDNL |
| POGLUT1 |
| AMT |
| PSKH1 |
| ACSS3 |
| BOLA1 |
| DNLZ |
| CARNMT1-AS1 |
| ENSG00000237705 |
| DAPK2 |
| MOAP1 |
| ENSG00000273797 |
| CAMKK1 |
| MED26 |
| ENSG00000240963 |
| SVEP1 |
| GUF1 |
| SEC16B |
| PI4KAP1 |
| ZNF540 |
| PROCR |
| DHCR24 |
| NEDD1 |
| TTC26 |
| RPE |
| EVC2 |
| DDR2 |
| TAS2R30 |
| ERMARD |
| ZNF140 |
| HCG25 |
| TGS1 |
| ENSG00000290399 |
| LNPK |
| ZFYVE27 |
| ENSG00000231201 |
| ATP2A3 |
| DCTN5 |
| MYLK4 |
| AKAP6 |
| SNAPC4 |
| CHD3 |
| ADSS1 |
| TSHR |
| MED17 |
| KRBOX5 |
| TMEM51-AS1 |
| ENSG00000287264 |
| FBXO3 |
| AP5M1 |
| CEP250 |
| TMEM132D |
| IFT140 |
| DIS3 |
| ENSG00000233478 |
| SLK |
| TMTC2 |
| CLPX |
| GTSF1 |
| ZNRF3 |
| AP1G2-AS1 |
| ENSG00000281181 |
| CD99L2 |
| ENSG00000259805 |
| OLFML3 |
| LINC02614 |
| PIP5K1C |
| TIGD1 |
| ENSG00000287299 |
| TMEM11-DT |
| RGPD2 |
| RFXAP |
| SYTL2 |
| HESX1 |
| ENSG00000288992 |
| ST18 |
| ENSG00000224905 |
| ENSG00000267248 |
| DCUN1D1 |
| ENSG00000267476 |
| ENSG00000279110 |
| RASSF8 |
| LRGUK |
| ADAMTS19 |
| ENSG00000284633 |
| SLC51A |
| RBFADN |
| HECW2 |
| SOX2-OT |
| SEPTIN7-DT |
| ENSG00000270071 |
| LINC02006 |
| ENSG00000288012 |
| ENSG00000283839 |
| HPS6 |
| ZNF195 |
| HMGB3 |
| RNF144A |
| ZNF891 |
| RHBDL2 |
| PTCD1 |
| CEP70 |
| CCDC150 |
| CRCP |
| CAPRIN2 |
| C11orf65 |
| CCDC32 |
| SPDYE5 |
| LINC02580 |
| ANO3 |
| IKBKE-AS1 |
| STRBP |
| SLC44A1 |
| LINC01515 |
| ENSG00000291209 |
| NAA40 |
| FBXO33 |
| MYL6B |
| ZNF786 |
| SLC38A10 |
| ATPSCKMT |
| LINC02316 |
| SRPX2 |
| TNS2 |
| ENOX1 |
| LDHD |
| KIAA1522 |
| FANCD2 |
| SHB |
| RANBP3 |
| CA3 |
| PHLDB1 |
| CHCHD4 |
| NOXRED1 |
| NR2C2AP |
| ENSG00000289469 |
| ZNF486 |
| WDR81 |
| PGA5 |
| ENSG00000286652 |
| LVRN |
| SPPL2B |
| KIAA1755 |
| IKBKE |
| GRID2IP |
| CHUK |
| CCSER1 |
| ZDBF2 |
| PALM2AKAP2 |
| TMEM268 |
| ZNF616 |
| ENSG00000290059 |
| APTR |
| ENSG00000278903 |
| SLC16A10 |
| CFAP69 |
| MYRIP |
| SLC2A12 |
| EHHADH |
| CFAP54 |
| TMEM266 |
| PIGZ |
| ASF1A |
| DNAH9 |
| CCSAP |
| ENSG00000236065 |
| MYO1B |
| CARD8-AS1 |
| ZSCAN26 |
| SPTLC1 |
| ZSCAN12 |
| FLNB |
| UBE2H-DT |
| GALNT17 |
| LINC00115 |
| NMD3 |
| ENSG00000246090 |
| TMED8 |
| TATDN1 |
| ENSG00000285731 |
| SP1 |
| KLHL7 |
| SPATC1L |
| ENSG00000290537 |
| CERS4 |
| MTHFSD |
| ZFYVE1 |
| ALG6 |
| RFTN2 |
| ZKSCAN7 |
| EVI5L |
| TMEM209 |
| ENSG00000233461 |
| ENSG00000223598 |
| ENSG00000230325 |
| CRPPA |
| DHX29 |
| ZKSCAN8 |
| ENSG00000259986 |
| PRAG1 |
| PENK |
| UPP2 |
| CWC22 |
| MFF-DT |
| CYP27C1 |
| GMEB1 |
| UCK2 |
| ENSG00000258666 |
| TMEM229B |
| SEC14L2 |
| TM2D1 |
| ZNG1F |
| ERCC5 |
| OMD |
| MSRB3 |
| CDKN2D |
| PAQR7 |
| ROBO2 |
| ZNF490 |
| LINC01389 |
| LINC02656 |
| POT1-AS1 |
| ENSG00000280614 |
| TANC1 |
| SYNPO2L-AS1 |
| DLEC1 |
| CCDC134 |
| TPM1 |
| AOX1 |
| MIR762HG |
| ENSG00000263300 |
| ENSG00000264456 |
| TJP1 |
| VPS37C |
| TRMT9B |
| UTP6 |
| PRKCQ |
| DUBR |
| PRDM6 |
| ENSG00000289884 |
| CTNNA2 |
| ZNF354A |
| LINC00882 |
| ENSG00000267672 |
| PAQR4 |
| NELFA |
| ITGA11 |
| ENSG00000270207 |
| ENSG00000261596 |
| LINC00603 |
| EVA1B |
| ENSG00000284602 |
| ROGDI |
| OTOAP1 |
| MYH3 |
| ENSG00000277855 |
| PIEZO1 |
| ENSG00000225889 |
| PEX6 |
| FRMD6 |
| ENSG00000250240 |
| PCDHGA10 |
| ARL6 |
| ALDH1L2 |
| CFAP61 |
| SEMA6D |
| LINC02516 |
| ZNF605 |
| ZNF827 |
| CRLS1 |
| RAPGEF4 |
| RSPRY1 |
| LGALSL-DT |
| ENSG00000267108 |
| MPZL2 |
| CHD1-DT |
| CEP78 |
| CAMTA2 |
| HMGXB4 |
| CCR5 |
| ENSG00000226281 |
| IGKV3-20 |
| DDX52 |
| WASL-DT |
| LSG1 |
| ALDH1A2 |
| TTTY14 |
| ENSG00000272234 |
| ADGRL3 |
| ESRRG |
| FANCI |
| UNC5B |
| SLC13A4 |
| ERG |
| KCNC1 |
| ENSG00000291182 |
| ENSG00000256568 |
| RNF26 |
| PABIR3 |
| VPS9D1 |
| ENSG00000246331 |
| DMGDH |
| VIRMA-DT |
| ENSG00000258454 |
| ENSG00000267598 |
| ZNF746 |
| ENSG00000286068 |
| ZNF12 |
| TRAF6 |
| ENTPD3-AS1 |
| CCDC92 |
| TMEM54 |
| ENSG00000249476 |
| CAND1 |
| NUDT13 |
| CDC42EP1 |
| KBTBD12 |
| INKA2 |
| RRS1 |
| SFR1 |
| CRADD-AS1 |
| ERMN |
| ENSG00000286828 |
| CLMN |
| ENSG00000290397 |
| SAMD4A-AS1 |
| DNAH6 |
| FSD2 |
| ENSG00000257298 |
| TBCC |
| KSR2 |
| ENSG00000232053 |
| SLC16A4-AS1 |
| ENSG00000247925 |
| PTGER3 |
| ZNF600 |
| KIAA0513 |
| EYA1 |
| BCOR |
| TIGAR |
| ENSG00000289727 |
| CBFA2T3 |
| SH2B1 |
| PAQR8 |
| ENSG00000286904 |
| RUFY1-AS1 |
| CPSF2 |
| PHRF1 |
| NCAPG2 |
| L3MBTL2-AS1 |
| KLF7-IT1 |
| CDADC1 |
| APCDD1L-DT |
| PRDM5 |
| STYXL1 |
| ENSG00000253636 |
| LRFN5 |
| ENSG00000271795 |
| STK31 |
| ENSG00000250126 |
| DEUP1 |
| SGCZ |
| TICAM2 |
| SUPV3L1 |
| SPHK2 |
| ABCC2 |
| B4GALNT3 |
| MRPS9 |
| CTTN-DT |
| KCNAB2 |
| GRM7 |
| SGSM3-AS1 |
| LTB |
| CEP63 |
| HRG-AS1 |
| PAXIP1-AS2 |
| ANKRD13A |
| NCALD |
| SART3 |
| HPSE2 |
| RAB15 |
| DOK6 |
| CASP2 |
| GGACT |
| OSBP |
| MROH8 |
| ENSG00000271860 |
| OXA1L-DT |
| SCAT2 |
| RYR3 |
| ADGRL1 |
| ADAMTS3 |
| NIPSNAP3B |
| LINC01948 |
| ADAM12 |
| MCM8 |
| LINC01290 |
| GPRASP1 |
| ENSG00000291144 |
| PALS2 |
| STEAP3-AS1 |
| SRPK1 |
| PRKX-AS1 |
| PUS7L |
| ZNF441 |
| AGPAT5 |
| NRG1 |
| SLC7A6OS |
| MXRA8 |
| PANK3 |
| KAT7 |
| ATP8B1-AS1 |
| LINC02202 |
| ENSG00000248367 |
| BMS1 |
| YY2 |
| ENSG00000284930 |
| MAP2K7 |
| AGMO |
| CENPI |
| CHN2-AS1 |
| NEXMIF |
| GALNT8 |
| LRRC17 |
| TGFA |
| ENSG00000285336 |
| TET1 |
| LINC01003 |
| LINC01435 |
| SRD5A1 |
| NECTIN3-AS1 |
| LINC01877 |
| KLHL6 |
| MAGI2-AS3 |
| GOLGA8R |
| SCARF1 |
| ENSG00000259536 |
| NRXN1 |
| ENSG00000240571 |
| IGF2BP2 |
| ENSG00000267694 |
| POM121C |
| MRPL49 |
| FRAT2 |
| ENSG00000254186 |
| STON1 |
| PTGR3 |
| ABLIM2 |
| POLE |
| NCF4-AS1 |
| ENSG00000276337 |
| LINC02828 |
| SYT15B |
| CAB39L |
| KIF6 |
| GORAB |
| RNF175 |
| NUDT15 |
| EVA1A |
| ENSG00000227681 |
| SLC18B1 |
| TRIM62 |
| HES1 |
| COG1 |
| COL12A1 |
| SETD7 |
| HACL1 |
| ZDHHC2 |
| DUSP28 |
| NR1D2 |
| ENSG00000260510 |
| IL10RB |
| ENSG00000257759 |
| ENSG00000286739 |
| ZNF595 |
| ENSG00000258312 |
| TAF1A |
| SNORC |
| ARHGAP44 |
| CAPN10-DT |
| MPV17L |
| ENSG00000291101 |
| HSDL2-AS1 |
| GINS1 |
| DHTKD1 |
| PFKFB1 |
| UPF3B |
| GPR89B |
| BMPR1B |
| CELF4 |
| EEPD1 |
| LINC01839 |
| ADGRE1 |
| FKBP11 |
| NEPRO-AS1 |
| FAM3B |
| CCDC86 |
| ENSG00000261096 |
| TLE2 |
| CSF3R |
| ENSG00000281195 |
| SLC25A37 |
| ZNF528 |
| ADGRL4 |
| ADAMTSL1 |
| MTRR |
| ABRAXAS2 |
| AP3M1 |
| LRIG1 |
| ZNF507 |
| ENSG00000233848 |
| TVP23B |
| AASS |
| ENSG00000227388 |
| ENSG00000289082 |
| GGH |
| NADK2 |
| MYO16 |
| ENSG00000246225 |
| FAM216A |
| LIAS |
| GOLGA3 |
| TBC1D8 |
| ZNF252P |
| MOV10 |
| ENSG00000226647 |
| ZSCAN9 |
| ZNF341-AS1 |
| ENSG00000289551 |
| PANK2-AS1 |
| TRAP1 |
| COL22A1 |
| SMIM35 |
| ZNF623 |
| ENSG00000235100 |
| C7orf25 |
| ENDOG |
| TDP1 |
| ELMO1-AS1 |
| EED |
| PIWIL2 |
| LINC02913 |
| HSPA12A |
| ENSG00000253197 |
| ENSG00000290095 |
| RNFT1 |
| SLC39A10 |
| ENSG00000248636 |
| ENSG00000286401 |
| DIAPH1-AS1 |
| FAM13C |
| TMEM102 |
| ENSG00000231104 |
| RAD17 |
| TMEM104 |
| PKNOX2 |
| ENSG00000290692 |
| BIN2 |
| RPL39L |
| DLGAP2 |
| ENSG00000287965 |
| ENSG00000259177 |
| CALCRL |
| ENSG00000262020 |
| ENSG00000287608 |
| ZNF189 |
| LINC02646 |
| ENSG00000226699 |
| COL18A1 |
| IQCH |
| ENSG00000225963 |
| PARD6A |
| ZSCAN32 |
| KIF21A |
| TTC33 |
| NAA60 |
| SLC35B3 |
| MACROH2A2 |
| RBM15 |
| TMEM108 |
| ENSG00000234426 |
| PACC1 |
| ZC2HC1C |
| ENSG00000227579 |
| NME9 |
| CHAD |
| ZKSCAN2-DT |
| ENSG00000285530 |
| LINC00624 |
| ENSG00000286861 |
| GSG1L |
| TPD52 |
| ENSG00000290958 |
| GPR180 |
| ENSG00000258603 |
| PPP3CB-AS1 |
| THAP1 |
| SLAMF7 |
| CYB561D1 |
| HELQ |
| HPS3 |
| NDUFAF2 |
| PPP1R9A |
| ASPN |
| ZNF213-AS1 |
| PCGF6 |
| ZNF660 |
| SDK2 |
| ENSG00000280164 |
| ENSG00000257221 |
| ENSG00000249988 |
| COL5A3 |
| RAB40A |
| ENSG00000228541 |
| AGO1 |
| GREB1L |
| TRIM52-AS1 |
| ENSG00000261889 |
| DDX42 |
| DPH5 |
| CRLF1 |
| LINC02456 |
| SKAP1-AS1 |
| ENSG00000233783 |
| ENSG00000279529 |
| TRIM2 |
| E2F3-IT1 |
| A2ML1-AS1 |
| ST7-AS2 |
| ANKRD24 |
| DHX37 |
| CCP110 |
| SLC35F3 |
| ZNF19 |
| KCNH1 |
| ENSG00000290588 |
| NRIP3 |
| TLE1-DT |
| ENSG00000285803 |
| ZFHX3-AS1 |
| ZSCAN2-AS1 |
| GRID2 |
| METAP1 |
| VCPKMT |
| PCDH15 |
| C5orf22 |
| TMCC1-DT |
| MARCKSL1 |
| SETD1B |
| SYNGAP1 |
| C2orf49 |
| ENSG00000260999 |
| ACTR5 |
| EOLA2 |
| SPESP1 |
| ENSG00000285994 |
| PKD1 |
| ALDH7A1 |
| VENTX |
| SUGP1 |
| FRMD5 |
| LY75 |
| LINC02511 |
| CAP2 |
| CD69 |
| COG2 |
| PTPRN2-AS1 |
| ENSG00000261474 |
| ZNF568 |
| CDC40 |
| NXT2 |
| GUCY2C-AS1 |
| STRIP1 |
| ABTB3 |
| ENSG00000258337 |
| ENSG00000272754 |
| BBS5 |
| KHDC1-AS1 |
| NME8 |
| GNPAT |
| IGKV1-12 |
| ENSG00000287269 |
| ASAP1-IT2 |
| OSGIN1 |
| ENSG00000274667 |
| TCAF1 |
| GALNT16-AS1 |
| GRIK4 |
| ENSG00000232748 |
| SLC25A27 |
| ENSG00000279833 |
| SENCR |
| LINC00504 |
| CHST14 |
| HIRA |
| ENSG00000259649 |
| MPZL1 |
| ENSG00000249175 |
| SCML2 |
| ZBTB25 |
| ITGA3 |
| ENSG00000289380 |
| C1orf21 |
| PHEX |
| ENSG00000267222 |
| TMEM182 |
| KIAA1549 |
| RETN |
| ZNF777 |
| ENSG00000263847 |
| ZNF892 |
| ACP6 |
| RRP36 |
| ENSG00000267904 |
| ENSG00000290835 |
| DHX33 |
| ENSG00000251556 |
| ENSG00000286924 |
| FKBP10 |
| PLBD1-AS1 |
| IP6K2 |
| THBS2 |
| USHBP1 |
| TDRD1 |
| ZNF93 |
| ENSG00000250602 |
| NTRK2 |
| ENSG00000244701 |
| GALNTL6 |
| KHK |
| FABP4 |
| COL25A1 |
| ATP13A2 |
| PKN2-AS1 |
| ESR2 |
| ADAM22 |
| ENSG00000268015 |
| ENSG00000273448 |
| ENSG00000239922 |
| SMAD1 |
| ENSG00000267274 |
| HECW1 |
| SUMO4 |
| GAS2 |
| MYO1H |
| PDGFRB |
| PTGFRN |
| NFATC2IP-AS1 |
| PLAG1 |
| C2orf88 |
| ENSG00000213279 |
| MORN1 |
| TMEM41B |
| COLGALT2 |
| ENSG00000222017 |
| ENSG00000289353 |
| EBF3 |
| ANKS6 |
| KCTD21-AS1 |
| PIWIL4-AS1 |
| POFUT1 |
| PRMT5-DT |
| SCRN3 |
| ENSG00000285184 |
| ITFG2-AS1 |
| PRDM16 |
| ENSG00000286431 |
| CHEK2 |
| ENSG00000253238 |
| TNFSF9 |
| ZNF491 |
| CHST13 |
| ENGASE |
| GALNT18 |
| GPD1 |
| CCDC73 |
| RARB |
| KIAA1958 |
| TPTE2 |
| ENSG00000243960 |
| EHBP1-AS1 |
| ENSG00000272054 |
| MAEA |
| GTF3C2-AS2 |
| CUBN |
| MUC12-AS1 |
| ZNF483 |
| TBCCD1 |
| ENSG00000228352 |
| TXLNGY |
| NRXN2 |
| CPT1B |
| DNMT1 |
| ENSG00000265739 |
| EIF5A2 |
| CHKB |
| AKT3-IT1 |
| SPOCK1 |
| CHRM5 |
| ENSG00000253645 |
| LINC02196 |
| GGT7 |
| MFSD2A |
| ACSM5 |
| ASB16-AS1 |
| CTDSPL2-DT |
| ZNF350-AS1 |
| TAC4 |
| POLR1B |
| RABL2B |
| PPFIA2 |
| ZNF625 |
| EREG |
| ZNF155 |
| GNB1L |
| ZNF250 |
| ENSG00000283045 |
| MTHFD2L |
| ZNF362 |
| LINC02934 |
| ENSG00000290812 |
| ZFP90 |
| LINC01366 |
| SLC22A16 |
| H2AC25 |
| CSNK2A2 |
| RSBN1 |
| SPDYE1 |
| ENSG00000232934 |
| CFAP70 |
| ZFHX2 |
| PAFAH1B3 |
| ENSG00000204971 |
| SLC25A53 |
| PRKAG2-AS2 |
| ENSG00000259767 |
| ENSG00000289443 |
| IFT43 |
| LINC02884 |
| MBOAT2 |
| ENSG00000287291 |
| PACRG |
| PPP2R3B |
| H2BC12 |
| CDRT4 |
| ZSCAN20 |
| ENSG00000290790 |
| UPK2 |
| SCAMP1 |
| MACC1 |
| ZNF79 |
| TPCN2 |
| SNTG2 |
| KIF24 |
| ARHGAP15-AS1 |
| MCAM |
| WWC1 |
| FAM184B |
| ENSG00000276248 |
| ENSG00000289043 |
| SYTL4 |
| ENSG00000205959 |
| DGKZ |
| CNTNAP3 |
| ENSG00000237938 |
| ENSG00000288737 |
| PIK3R6 |
| ZNF274 |
| RABL2A |
| SYT12 |
| BAZ2B-AS1 |
| PARVA |
| CDH26 |
| CISH |
| LRRC8C-DT |
| OGN |
| ENSG00000259635 |
| PYROXD2 |
| LINC02026 |
| ST8SIA1 |
| BTD |
| FAM50B |
| ZNNT1 |
| LINC01550 |
| SLC9A6 |
| ENSG00000227240 |
| RHD |
| NPIPB3 |
| LINC01340 |
| ANKRD44-IT1 |
| ENSG00000287024 |
| ANKZF1 |
| LHCGR |
| AADACP1 |
| SNRNP48 |
| ENSG00000232721 |
| MAPKAPK5 |
| ITGA7 |
| ENSG00000267698 |
| ITGB3 |
| PTCSC2 |
| ENSG00000276564 |
| ENSG00000264290 |
| L3MBTL4-AS1 |
| RBAK |
| ENSG00000248664 |
| SYNGAP1-AS1 |
| FRMD6-AS2 |
| KCNE4 |
| LINC02609 |
| CDK11B |
| URB1-AS1 |
| SEC23B |
| OSBP2 |
| ZBED5 |
| ZNF665 |
| LINC02889 |
| DUSP8 |
| ENSG00000285622 |
| MIR3936HG |
| THAP2 |
| BARD1 |
| SERPINI1 |
| ANKHD1-DT |
| ZNF707 |
| HOMER1 |
| PAN2 |
| DPP6 |
| MFSD6 |
| PTS |
| DHDDS-AS1 |
| LUM |
| OAZ3 |
| TACC3 |
| ENSG00000266368 |
| ANO2 |
| HDHD5-AS1 |
| ENSG00000287042 |
| LINC00299 |
| DISC1FP1 |
| HGF |
| COL10A1 |
| ENDOV |
| OR1B1 |
| EPCAM |
| ENSG00000287972 |
| TMEM169 |
| SRXN1 |
| LINC00222 |
| MMP28 |
| HHIPL1 |
| ZNF823 |
| RGS16 |
| HES6 |
| ENSG00000288853 |
| ENSG00000260816 |
| TOGARAM2 |
| PIEZO2 |
| ENSG00000290888 |
| PHC2-AS1 |
| ENSG00000287180 |
| ENSG00000287286 |
| CHRNA3 |
| AOC1 |
| ENSG00000257496 |
| ZNF177 |
| KREMEN1 |
| CMTR2 |
| AFAP1L1 |
| TIPARP-AS1 |
| AGBL4 |
| CYP2R1 |
| ZIC1 |
| SLC25A45 |
| KCNIP1 |
| ENSG00000248994 |
| LDB1 |
| ENSG00000265316 |
| EHD4-AS1 |
| NIM1K |
| NUTM2E |
| INTS5 |
| RNF31 |
| LYG1 |
| ENSG00000261766 |
| H3-3A-DT |
| NRG3 |
| PABPC1L |
| PRXL2B |
| ENSG00000285783 |
| MAD2L1BP |
| C7 |
| ENSG00000287978 |
| CADM2 |
| PELO |
| ZNF606 |
| CIDEC |
| ZKSCAN5 |
| ENSG00000234425 |
| ENSG00000288744 |
| DIXDC1 |
| ZNHIT2 |
| KLHL36 |
| POMT1 |
| ENSG00000247121 |
| HULC |
| DOC2A |
| ENSG00000276672 |
| LINC01505 |
| ENSG00000290830 |
| KCNIP2-AS1 |
| GDPD5 |
| ENSG00000255672 |
| IRF3 |
| VSTM4 |
| ENSG00000276724 |
| PRR3 |
| PTPRT |
| INTS4P1 |
| TSPOAP1 |
| PHACTR2-AS1 |
| CCDC71L |
| FMO5 |
| FAM86DP |
| THRB-IT1 |
| ENSG00000257735 |
| DUSP7 |
| MATK |
| ENSG00000288714 |
| NSUN5P2 |
| BEND7 |
| TLR6 |
| RARG |
| CFTR |
| ENSG00000285080 |
| COQ8B |
| SCIN |
| TFAP2E |
| ENSG00000286755 |
| MUC20 |
| LMLN |
| ADAMTS12 |
| EDEM2 |
| HSPA2 |
| ENSG00000290762 |
| ENSG00000261369 |
| ENSG00000285651 |
| KLHL3 |
| FGF7 |
| SHPK |
| CNOT11 |
| ENSG00000238246 |
| PPP5D1P |
| LINC00540 |
| PHKA1 |
| DIO3OS |
| CFLAR-AS1 |
| ENSG00000274421 |
| FRMPD4 |
| DOCK8-AS2 |
| CBLL1-AS1 |
| ST7-OT4 |
| ATAD3B |
| APOLD1 |
| RABEP2 |
| BCL10-AS1 |
| TMEM191A |
| ENSG00000256116 |
| ENSG00000269189 |
| ENSG00000267009 |
| RMI1 |
| KCNMA1-AS1 |
| QSOX2 |
| TTC4 |
| ENSG00000261218 |
| IGIP |
| ENSG00000284634 |
| LINC00996 |
| LINC01089 |
| DUOX1 |
| MTARC1 |
| IFT74 |
| PRPSAP2 |
| NDST1 |
| MRPL53 |
| PARPBP |
| EMCN |
| WDR53 |
| ZNF876P |
| TARS3 |
| KNOP1 |
| ENSG00000273828 |
| SYN3 |
| ENSG00000236266 |
| ZNF257 |
| ENSG00000287839 |
| MAP2 |
| DCLRE1B |
| RPGRIP1L |
| ENSG00000260773 |
| ENSG00000285939 |
| RIC3 |
| CASC2 |
| PREX2 |
| MSH4 |
| OBI1 |
| EXD2 |
| ENSG00000231024 |
| SV2C |
| ENSG00000288919 |
| LINC02284 |
| ENSG00000236234 |
| GOLGA5 |
| LINC02917 |
| KLHL13 |
| EGLN1 |
| ENSG00000241772 |
| ENSG00000259899 |
| CLIC6 |
| PJA1 |
| DRC3 |
| KIF27 |
| ENSG00000285801 |
| PCCA-AS1 |
| ZNF615 |
| POLR3F |
| SLC35E3 |
| AATBC |
| TENM2 |
| H2BC4 |
| ARHGAP11B |
| SLC39A8 |
| DOCK3 |
| ENSG00000287553 |
| FMOD |
| FAM184A |
| BLCAP |
| ENSG00000290784 |
| ENSG00000257342 |
| NSL1 |
| ENSG00000291208 |
| ZNF2 |
| LCMT1-AS1 |
| LINC02642 |
| LINC00534 |
| DEPDC4 |
| WASH2P |
| ZNF317 |
| ENSG00000285796 |
| ENSG00000277701 |
| ENSG00000287202 |
| TWIST2 |
| MVP-DT |
| CD226 |
| EPS15-AS1 |
| ARHGEF26 |
| DUSP18 |
| ENSG00000285621 |
| A1BG-AS1 |
| ENSG00000290450 |
| LINC02993 |
| GUCY1A1 |
| LRRC74A |
| GUCA1B |
| SDC2 |
| TTC9C |
| CDKN2B |
| PNMA8B |
| ROBO4 |
| FGD1 |
| COP1-DT |
| KCNK10 |
| BICRA-AS1 |
| SLFN12 |
| ANO5 |
| ENSG00000275202 |
| CDS2 |
| THEM6 |
| ENSG00000264520 |
| ENSG00000258740 |
| EFNA2 |
| ENSG00000240093 |
| TMEM88 |
| PPL |
| TSBP1-AS1 |
| PSMD6-AS2 |
| KANSL2 |
| TH2LCRR |
| FNDC10 |
| CEP97 |
| NPIPA1 |
| CCDC40 |
| EXTL3 |
| KIF3B |
| FZD4-DT |
| STX17-DT |
| ZNF8 |
| ENSG00000260805 |
| ENSG00000285090 |
| ENSG00000286292 |
| CCL5 |
| LANCL1-AS1 |
| ENSG00000255139 |
| MAP3K21 |
| TENT5A |
| ATP8B2 |
| RANBP17 |
| PITX1-AS1 |
| DCC |
| ZNF571 |
| ENSG00000289788 |
| ENTPD7 |
| ENSG00000279168 |
| BRIP1 |
| CAV1 |
| IGSF10 |
| SPACA6 |
| LINC00467 |
| PFKFB2 |
| L3MBTL3 |
| KCNK6 |
| DANT2 |
| HSPA2-AS1 |
| GADL1 |
| ZNF787 |
| ENSG00000257277 |
| SLCO5A1 |
| EOLA1 |
| CCZ1 |
| ENSG00000290767 |
| TACR1 |
| TG |
| ENSG00000277969 |
| GRB10 |
| SPDYE2 |
| JADE3 |
| NFIX |
| CYP7B1 |
| EML6 |
| SPTBN5 |
| THEM4 |
| LINC02250 |
| ENSG00000290285 |
| DNAAF11 |
| RTCA-AS1 |
| PKD1L1 |
| CYB5RL |
| CGNL1 |
| ENSG00000260955 |
| ENSG00000254394 |
| CDH12 |
| KAT14 |
| CCDC28A-AS1 |
| ENSG00000289259 |
| LINC02245 |
| FGF2 |
| ENSG00000266538 |
| ENSG00000273165 |
| LINC01322 |
| GSTO2 |
| ABCB1 |
| FZD6 |
| ING4 |
| POLR3C |
| NLRX1 |
| SLC35E1 |
| GNG7 |
| DST-AS1 |
| GYPE |
| CNKSR3 |
| NPM3 |
| SLC22A1 |
| XYLT2 |
| ENSG00000291079 |
| DIABLO |
| PRKAB2 |
| PANK1 |
| ALG14 |
| DNAH7 |
| NMRAL2P |
| PCYT2 |
| MOGS |
| CUZD1 |
| VASH1-DT |
| ZNF525 |
| TOX2 |
| LINC00623 |
| CEP68 |
| LINC01301 |
| FKBP14 |
| CALCB |
| FBLN1 |
| ENSG00000230105 |
| POLR1C |
| COPS8-DT |
| TMA16 |
| TTC36-AS1 |
| ZNF557 |
| ENSG00000262979 |
| ENSG00000253288 |
| EDIL3 |
| TDG |
| HMCN2 |
| SLC9B2 |
| PIP5K1B |
| KCNMB4 |
| GMPS |
| FHOD3 |
| ENSG00000289306 |
| PPFIA4 |
| KLF3-AS1 |
| TIMM44 |
| DNAAF5 |
| ENSG00000276728 |
| ENSG00000238039 |
| CDHR3 |
| CCDC13-AS2 |
| ENSG00000265656 |
| ENSG00000240207 |
| ENSG00000287127 |
| ENSG00000278576 |
| ENSG00000261386 |
| ORMDL3 |
| SPAG1 |
| TRIM52 |
| C16orf54 |
| ZNF384 |
| ENSG00000258824 |
| ENSG00000250592 |
| SLC25A28 |
| CLDN12 |
| THSD7B |
| ADAM19 |
| STK36 |
| ENSG00000242628 |
| COL16A1 |
| LINC01091 |
| PCOLCE2 |
| ENSG00000236069 |
| CRTC3-AS1 |
| S1PR4 |
| ENSG00000289839 |
| NUS1 |
| ENSG00000286136 |
| TRDMT1 |
| VASN |
| ABCC9 |
| ENSG00000249593 |
| PPP1R12C |
| CLSTN3 |
| TREX1 |
| REXO1 |
| ALDH1A3-AS1 |
| ENSG00000242798 |
| LINC03016 |
| COL21A1 |
| GTF2E1 |
| GALNT7-DT |
| ZNF674 |
| ENSG00000290646 |
| CLEC4E |
| BRF2 |
| ENSG00000262877 |
| ENSG00000254129 |
| F3 |
| TRIM35 |
| NUP210 |
| TMEM68 |
| GNAI1 |
| CSRNP2 |
| MMAA |
| ENSG00000249258 |
| POLL |
| MCAT |
| ENSG00000289294 |
| SAP30 |
| HOXD3 |
| ZNF236-DT |
| ENSG00000260793 |
| ZNF563 |
| ZNF225 |
| ENSG00000287463 |
| CDS1 |
| ENSG00000287168 |
| RAB3A |
| DEAF1 |
| RFX1 |
| HINFP |
| CERK |
| HELLPAR |
| SHOX2 |
| CECR2 |
| LINC00987 |
| RSPO2 |
| MED9 |
| TUFT1 |
| TTC31 |
| IGLV2-14 |
| DPF2 |
| ENSG00000229019 |
| AQP3 |
| ENSG00000285979 |
| ENSG00000254936 |
| SKP2 |
| H2AC20 |
| RRAS2 |
| AR |
| ENSG00000286242 |
| ENSG00000259617 |
| ENSG00000273320 |
| ENSG00000260949 |
| TIGD7 |
| PORCN |
| ASB14 |
| RGMA |
| ENSG00000261838 |
| JAM3 |
| ANGPTL4 |
| NEBL |
| TMEM252-DT |
| ENSG00000243155 |
| LMBR1L |
| ZNF510 |
| SLPI |
| NEURL1 |
| ENSG00000228679 |
| ENSG00000286895 |
| ENSG00000250751 |
| PDF |
| EML2 |
| LRRC37A3 |
| INAFM2 |
| TRUB1 |
| SLC35A5 |
| ENSG00000289586 |
| ELAPOR2 |
| LNCRNA-IUR |
| ENSG00000291202 |
| ENSG00000235085 |
| ENSG00000241860 |
| CHRM3 |
| ENSG00000290091 |
| ENSG00000287621 |
| ENSG00000242880 |
| PC |
| VGLL3 |
| ENSG00000287175 |
| ENSG00000289269 |
| CIP2A |
| GAMT |
| IGKV3-15 |
| ENSG00000272953 |
| GVQW3 |
| ANKRD16 |
| SLFN12L |
| POSTN |
| OSGIN2 |
| SLC17A9 |
| ENSG00000258695 |
| ENSG00000289039 |
| PDCD4-AS1 |
| ENSG00000265254 |
| EEF1AKMT4 |
| PRDM4-AS1 |
| ENSG00000267379 |
| TOX |
| TCF3 |
| GRIP2 |
| ENSG00000233739 |
| IL2RG |
| KTN1-AS1 |
| EFNA4 |
| ENSG00000285103 |
| ENSG00000230010 |
| MXRA5 |
| ENSG00000287925 |
| ZBTB48 |
| ENSG00000285043 |
| GCNT7 |
| ENSG00000225718 |
| ENSG00000262898 |
| MECR |
| ENSG00000256789 |
| CPSF3 |
| WASF3 |
| KIAA1614 |
| RNF180 |
| PPP1CB-DT |
| WHAMM |
| EDARADD |
| POLB |
| ENSG00000231329 |
| LIMD2 |
| ENSG00000230107 |
| KCTD11 |
| KLRG1 |
| TTLL7 |
| MFSD3 |
| REC114 |
| SMAD9 |
| ZNF596 |
| GPT2 |
| MKRN2OS |
| RIPOR3 |
| NTRK3 |
| CRISPLD1 |
| HS6ST1 |
| EOGT-DT |
| CHD1L |
| CCDC26 |
| ADAM32 |
| ENSG00000229195 |
| LINC01320 |
| OGG1 |
| LTBP4 |
| CEP76 |
| ENSG00000277959 |
| ACOT4 |
| ENSG00000263657 |
| KCNJ1 |
| ENSG00000273319 |
| FCSK |
| S100A3 |
| SNAI3-AS1 |
| LINC01002 |
| SEMA6A |
| ZNF692 |
| NEMP2 |
| PLS1 |
| ENSG00000269937 |
| MAPRE3 |
| NSMCE1-DT |
| ZNF799 |
| CCDC51 |
| ENSG00000289470 |
| ZNF684 |
| ENSG00000259539 |
| C19orf25 |
| C19orf38 |
| CEACAM1 |
| ARSJ |
| TNFAIP6 |
| ENSG00000228280 |
| GSPT2 |
| LINC01117 |
| ZNF512B |
| SMAD6 |
| CCL3-AS1 |
| CRACD |
| MAFTRR |
| ENSG00000261094 |
| GRID1 |
| ENSG00000266896 |
| ENSG00000257831 |
| ENSG00000291228 |
| SMC1B |
| ENSG00000276115 |
| LRP2BP-AS1 |
| SLC25A51 |
| ENSG00000222043 |
| TMCC2 |
| ZNF470-DT |
| GPR89A |
| CD274 |
| ENSG00000286076 |
| NDNF |
| WDR31 |
| SLC25A25-AS1 |
| PRIM1 |
| NRL |
| PIGM |
| SNHG4 |
| LINC01135 |
| TMEM44 |
| ENSG00000286471 |
| ENSG00000257732 |
| ADAMTS7 |
| ENSG00000258380 |
| RNPC3-DT |
| ENSG00000272211 |
| ENSG00000289161 |
| LINC00159 |
| ARHGAP1 |
| ID2-AS1 |
| ENSG00000286511 |
| ENSG00000254631 |
| ZFP37 |
| ENSG00000255886 |
| PLPP7 |
| EBLN2 |
| ENSG00000259103 |
| ENSG00000261560 |
| CPXM2 |
| IQSEC2 |
| ODF2 |
| ENSG00000286863 |
| ZNF727 |
| IPP |
| USP28 |
| TEKTIP1 |
| ENSG00000286864 |
| ENSG00000290590 |
| ZNF318 |
| KCNMB2-AS1 |
| BCAR3-AS1 |
| FLT1 |
| SLIT1 |
| EME2 |
| UHRF1 |
| IMPG2 |
| ENSG00000261124 |
| RPGRIP1 |
| CDR1 |
| ANKDD1B |
| INTS12 |
| C4orf45 |
| FANK1 |
| ENSG00000269688 |
| ENSG00000239407 |
| ZNF316 |
| RNF139-DT |
| MOK |
| TMEM192 |
| MET |
| HSD17B1-AS1 |
| ENSG00000234902 |
| ARHGEF17 |
| S100A1 |
| TLCD2 |
| VIPR1-AS1 |
| BMPER |
| ADAT1 |
| ENSG00000258101 |
| CADPS |
| ENSG00000288863 |
| IRF2BP1 |
| PABIR2 |
| ABCC13 |
| ENSG00000261451 |
| ZNF845 |
| CHODL |
| LY86-AS1 |
| ZNF813 |
| ENSG00000276517 |
| GCNA |
| ENSG00000289526 |
| NCKAP5-AS2 |
| USP30 |
| PPP2R2B |
| FAM227B |
| PAWR |
| LINC02955 |
| FBXO21 |
| CRTAM |
| CHCHD6 |
| SRR |
| ATAT1 |
| SPATA17 |
| PDXP |
| BAHD1 |
| ENAH |
| ENSG00000249492 |
| ENSG00000273338 |
| MARCHF5 |
| TJAP1 |
| PALM |
| TLR5 |
| SLC35G1 |
| MEOX2 |
| DTD1 |
| FEN1 |
| WDR11-DT |
| LBHD2 |
| TARS2 |
| SMOC1 |
| DACH1 |
| DOCK9 |
| ZNF888 |
| ZNF354C |
| PIM2 |
| POLM |
| ACADSB |
| CALCOCO1 |
| SEPSECS-AS1 |
| SSC5D |
| DENND2C |
| NEMP2-DT |
| MTUS2 |
| PCA3 |
| ELOVL6 |
| LINC00663 |
| ENSG00000267571 |
| LINC02201 |
| PDE9A |
| ENSG00000286715 |
| PIGF |
| ENSG00000248734 |
| ENSG00000264513 |
| TTC38 |
| FOLR3 |
| RRP1B |
| OCLN |
| ENSG00000289574 |
| SMN2 |
| TSSK3 |
| TNNI2 |
| COL27A1 |
| FAM24B |
| SPACA9 |
| LINC02175 |
| HCCS-DT |
| CYP3A5 |
| KIF3C |
| ENSG00000286581 |
| PDE2A-AS2 |
| MEGF8 |
| JADE2 |
| PAFAH2 |
| MYEF2 |
| ENSG00000287979 |
| MAP3K10 |
| ENSG00000288555 |
| LINC00865 |
| EEF1AKMT1 |
| ING5 |
| PLA2R1 |
| POLR3E |
| TACR2 |
| SIRPB1 |
| CDK7 |
| ENSG00000290088 |
| ZNF649 |
| ABCC5-AS1 |
| ARSD |
| SLFNL1 |
| VWC2 |
| IQCJ |
| HEATR4 |
| LINC02763 |
| PPP1R12A-AS1 |
| ABCA9-AS1 |
| ENSG00000267436 |
| ENSG00000282022 |
| ZNF555 |
| TUBA4A |
| OCIAD2 |
| RPA4 |
| TMSB15B-AS1 |
| CD96 |
| COX19 |
| ZNF223 |
| IGHA2 |
| ENSG00000235450 |
| FKBPL |
| GTF3C1 |
| UTP25 |
| CASP7 |
| ERLIN1 |
| IL2RA |
| ENSG00000290127 |
| ENSG00000281016 |
| CDR2-DT |
| C10orf105 |
| ENSG00000247134 |
| ENSG00000251600 |
| C17orf80 |
| ZKSCAN4 |
| GHRL |
| MAOB |
| DDX19A |
| LINC01356 |
| MCEE |
| ENSG00000290560 |
| C14orf93 |
| DYNLT2 |
| PPEF1 |
| NECAP1 |
| ADAMTSL2 |
| ENSG00000286599 |
| CHST3 |
| LINC01736 |
| ZNF85 |
| MPP3 |
| ENSG00000227803 |
| TMEFF1 |
| SBF2-AS1 |
| RASGRP1 |
| CYYR1 |
| LINC02910 |
| ACSM3 |
| ENSG00000288772 |
| FLJ46284 |
| ENSG00000235749 |
| TMPRSS13 |
| NBPF3 |
| ENSG00000265943 |
| ENSG00000287242 |
| ENSG00000266371 |
| SPATA21 |
| ENSG00000253214 |
| AOC3 |
| SLC35B4 |
| MCEMP1 |
| C1orf53 |
| FBXO16 |
| ENSG00000273341 |
| NHEJ1 |
| ZNF324B |
| VXN |
| ATP6V1C2 |
| ENSG00000289242 |
| LRRC15 |
| PITPNM3 |
| TMED6 |
| ENSG00000259437 |
| ALS2CL |
| ENSG00000276334 |
| LRIG3 |
| ADGRF5 |
| ZNF324 |
| ENSG00000287855 |
| ENSG00000290048 |
| ZNF227 |
| MED14OS |
| ENSG00000214803 |
| NPIPB11 |
| NUDT8 |
| VIT |
| LINC02055 |
| RERE-AS1 |
| ENSG00000269514 |
| ATAD2 |
| CILP |
| ALG1L9P |
| SPEG |
| ENSG00000249236 |
| ZNF433 |
| ARHGAP32 |
| SORBS1 |
| CHRNA7 |
| LINC02008 |
| ENSG00000289534 |
| TMEM30A-DT |
| LZTR1 |
| RIOX1 |
| POLG |
| ENSG00000226601 |
| HPN |
| POMT2 |
| LINC02236 |
| ENSG00000286786 |
| ENSG00000287625 |
| SIRT5 |
| NDUFA6-DT |
| ENSG00000270050 |
| EEF2KMT |
| ENSG00000288067 |
| SP140 |
| MMP21 |
| TUBA1B-AS1 |
| ENSG00000287188 |
| TAF8 |
| HLTF |
| SMIM10 |
| CCL23 |
| METTL4 |
| LRRN3 |
| ENSG00000270571 |
| FAM228A |
| ENSG00000286147 |
| NAPSB |
| ENSG00000260086 |
| FAM78A |
| DDX51 |
| LINC00173 |
| IGFBP3 |
| ENSG00000290870 |
| GPR161 |
| GJA1 |
| RECQL5 |
| PIP4K2B |
| IGHGP |
| TJP2 |
| ERC2 |
| ENSG00000286809 |
| ENSG00000285877 |
| ENSG00000286319 |
| TMEM184B |
| HYAL1 |
| CATIP-AS2 |
| BACH2 |
| GTPBP4 |
| CACNB3 |
| CDCA4 |
| PRKCA-AS1 |
| ENSG00000281383 |
| MDM1 |
| ENSG00000241073 |
| METTL3 |
| LINC00309 |
| ENSG00000259891 |
| ZDHHC23 |
| MIGA2 |
| USP30-AS1 |
| ECT2L |
| STARD8 |
| ELMO2 |
| RGPD5 |
| DPH1-AS1 |
| KCTD21 |
| TTYH2 |
| SPTBN2 |
| ENSG00000255462 |
| ENSG00000270277 |
| LRRC1 |
| SETMAR |
| HNRNPA1L3 |
| BRCA2 |
| LEFTY1 |
| ATG13 |
| NTAQ1 |
| RABL3 |
| ZFP3 |
| LINC03022 |
| ICAM5 |
| METTL6 |
| YJEFN3 |
| CASTOR2 |
| BACE1 |
| TMEM45A |
| DNAJA3 |
| FFAR2 |
| ENSG00000260536 |
| ZNF260 |
| GUCY1A2 |
| TMEM26 |
| FAM222A |
| C19orf18 |
| POLQ |
| MS4A6E |
| ENSG00000291081 |
| CREG2 |
| PXMP2 |
| NECTIN3 |
| ZP3 |
| ENSG00000283384 |
| CAVIN1 |
| ENSG00000288029 |
| NEFH |
| LURAP1 |
| BDKRB2 |
| CENPE |
| C21orf58 |
| DDX11-AS1 |
| SGK2 |
| KCNC4 |
| ENSG00000291219 |
| ZBTB5 |
| FAM86B3P |
| ENSG00000289260 |
| ADHFE1 |
| NPHP1 |
| ENSG00000263033 |
| PLET1 |
| ENSG00000260111 |
| ANKRA2 |
| TBC1D30 |
| RAB37 |
| MIER3 |
| NLRP12 |
| KDM8 |
| ENSG00000286994 |
| TMEM254-AS1 |
| NOMO2 |
| SFRP1 |
| NAALAD2 |
| LINC00482 |
| ENSG00000289035 |
| ENSG00000285940 |
| KDM7A-DT |
| LRRC3 |
| TNIK |
| KDM5D |
| TRAFD1 |
| DTX4 |
| LINC01353 |
| KLC4 |
| PRTFDC1 |
| CDC20-DT |
| LINC02145 |
| HIGD1C |
| RRP15 |
| THSD1 |
| ENSG00000289276 |
| ADRA2B |
| SLAMF6 |
| CADM1 |
| REEP6 |
| ENSG00000231401 |
| FAM174B |
| ATP1A1-AS1 |
| ENSG00000223576 |
| HAS2 |
| MATCAP1 |
| PM20D2 |
| AQP11 |
| MELK |
| ENSG00000290535 |
| AKR1C8 |
| ENSG00000227627 |
| LINC02757 |
| ZNF487 |
| TP63 |
| ENSG00000232884 |
| FAM209A |
| SLC1A3-AS1 |
| H4C6 |
| CLDN7 |
| ZNF311 |
| TRIQK |
| MRO |
| LINC01357 |
| RNASEH2A |
| ENSG00000272630 |
| SLX4 |
| SLC16A13 |
| FLACC1 |
| TRMT61B |
| TAS2R14 |
| LINC00937 |
| LINC02234 |
| ENSG00000258455 |
| DNAH3 |
| ARHGAP23 |
| ENSG00000258302 |
| UBE2Q2P2 |
| OAS3 |
| SLC25A30 |
| RFX5-AS1 |
| ENSG00000250604 |
| ENSG00000260392 |
| ENSG00000287092 |
| ENSG00000260651 |
| KLKB1 |
| BLMH |
| GPR160 |
| ZNF92 |
| MTARC2 |
| RANBP3L |
| TMEM236 |
| ZNF860 |
| ENSG00000274215 |
| LDHAL6A |
| MICB-DT |
| VAT1L |
| ENSG00000287637 |
| UBR5-DT |
| PRKCH |
| ANKFN1 |
| KIF3A |
| SMO |
| AK4 |
| ENSG00000290843 |
| LY6G5C |
| SAP30L |
| SPATA13 |
| ENSG00000255670 |
| DPP10 |
| CLCN4 |
| ENSG00000286489 |
| CLEC18B |
| ENSG00000291068 |
| CIT |
| ETV1 |
| CHKB-DT |
| DYNLT5 |
| MEGF6 |
| ENSG00000269967 |
| B4GALT1-AS1 |
| ACADM |
| RARRES2 |
| RHOJ |
| DICER1-AS1 |
| SMARCA1 |
| RPAP3-DT |
| ENSG00000280800 |
| ENSG00000289868 |
| ENSG00000266385 |
| PMS2 |
| FAT1 |
| ENSG00000239300 |
| UBTD1 |
| ENSG00000251023 |
| IGLV6-57 |
| FAM43A |
| PTGIS |
| ZNF214 |
| ENSG00000254367 |
| BMP1 |
| ENSG00000271774 |
| LINC01134 |
| ENSG00000288879 |
| SLC44A2 |
| WAKMAR2 |
| ENSG00000288066 |
| FAM20C |
| ENSG00000284968 |
| SLC7A1 |
| EPB41L4A |
| SLC16A2 |
| SPDYE18 |
| NNMT |
| MTURN |
| ADAT3 |
| TMCO1-AS1 |
| GTPBP2 |
| GOLM1 |
| SCO1 |
| LINC00665 |
| DIO2 |
| UMPS |
| DDHD1-DT |
| TBC1D8B |
| FAM238A |
| LINC01954 |
| RABGAP1L-AS1 |
| MTUS1-DT |
| CBX7 |
| CCR3 |
| GPD1L |
| ENSG00000243944 |
| ULK4P2 |
| ZNF468 |
| HELZ2 |
| PCAT1 |
| DZIP1 |
| RAPGEFL1 |
| ENSG00000236723 |
| PIR |
| MAP3K9 |
| JAM2 |
| LINC01931 |
| DRICH1 |
| LINC02381 |
| ENSG00000267546 |
| ZDHHC18 |
| LINC02666 |
| ENSG00000259704 |
| TMC8 |
| PLCG1 |
| GAS1RR |
| ZNF620 |
| PODN |
| TAF1C |
| PLA2G2D |
| IGHM |
| CD2BP2-DT |
| RGS5 |
| ENSG00000255182 |
| ENSG00000249635 |
| CNTNAP1 |
| IKZF5 |
| ALDH1B1 |
| TMED2-DT |
| SIX2 |
| WASF1 |
| STARD4-AS1 |
| ZNF23 |
| DLGAP1-AS2 |
| ZWINT |
| SLC25A29 |
| LASP1NB |
| ENSG00000278834 |
| MATN2 |
| MUC1 |
| SLC25A20 |
| BIRC7 |
| ENSG00000233223 |
| SPNS2 |
| ENSG00000258344 |
| NOS1AP |
| ENSG00000273145 |
| LINGO2 |
| ENSG00000225218 |
| PROSER1 |
| ADAL |
| ENSG00000255158 |
| TMPRSS5 |
| FBXL19 |
| PKIA-AS1 |
| MIR497HG |
| RGS13 |
| FAM135B |
| C9orf153 |
| ENSG00000253200 |
| UPRT |
| ZNF18 |
| WTIP |
| IGLV2-23 |
| ENSG00000288745 |
| RRN3P3 |
| BHMT2 |
| LINC02207 |
| NPTN-IT1 |
| ENSG00000288925 |
| DGUOK-AS1 |
| AFTPH-DT |
| BRCC3 |
| GOLGA8H |
| TRIM72 |
| ENSG00000289995 |
| IGLV3-25 |
| ENSG00000269825 |
| RNFT2 |
| TUBE1 |
| BAZ1A-AS1 |
| ENSG00000287255 |
| CCDC126 |
| ENSG00000272449 |
| USP34-DT |
| FHAD1-AS1 |
| ADPRM |
| PELI3 |
| C1QTNF1 |
| CEP72 |
| ZFP2 |
| FKRP |
| TRAPPC2 |
| TIFA |
| HAMP |
| UNC79 |
| RAD54B |
| PDIK1L |
| ENSG00000290032 |
| KIF20B |
| RCC1L |
| SULT1C2 |
| DGKE |
| ENSG00000286688 |
| A2MP1 |
| ENSG00000273064 |
| LINC03072 |
| RAET1E-AS1 |
| ENSG00000228566 |
| ENSG00000273175 |
| ENSG00000287655 |
| HCAR2 |
| LINC02256.1 |
| ENSG00000286656 |
| ENSG00000287875 |
| ENSG00000285728 |
| IGHV4-39 |
| RTP4 |
| ENSG00000224789 |
| AP1S3 |
| ENSG00000273284 |
| ZNF350 |
| ENSG00000228509 |
| RTL10 |
| TK1 |
| CLUAP1 |
| ENSG00000273272 |
| ENSG00000230303 |
| DSTNP2 |
| FAAH2 |
| CAV2 |
| RFPL1S |
| IGHV1-3 |
| SPP1 |
| ENSG00000288061 |
| S100A2 |
| MMP9 |
| AKR1E2 |
| KITLG |
| CSF1 |
| CRACDL |
| BMP5 |
| TPH1 |
| ENSG00000255910 |
| FBXO31 |
| EIF4E3 |
| PCDHGB6 |
| GEMIN8 |
| BRD3OS |
| KLF5 |
| ENSG00000290385 |
| MAFIP |
| FBF1 |
| HIVEP2-DT |
| MCPH1-DT |
| ENSG00000286024 |
| CMYA5 |
| ENSG00000284052 |
| AMOTL2 |
| RHPN2 |
| PVALB |
| ENSG00000269578 |
| ZNF558 |
| SGCG |
| ENSG00000291215 |
| ZKSCAN3 |
| ROBO3 |
| CHI3L2 |
| OSBPL7 |
| EXOSC3 |
| GPSM1 |
| ZNF574 |
| ENSG00000238221 |
| FAM177B |
| MTX3 |
| ENSG00000289152 |
| ENSG00000254409 |
| XCR1 |
| PLB1 |
| EXPH5 |
| BCL11A |
| LMOD1 |
| ENSG00000288818 |
| OCM |
| FOXP1-DT |
| SMN1 |
| S100Z |
| LINC02985 |
| DCUN1D3 |
| ENSG00000254865 |
| DARS2 |
| TRPV1 |
| METTL18 |
| CNTNAP3B |
| TECTA |
| NEK5 |
| ANOS1 |
| ENSG00000250820 |
| LINC02080 |
| POLR2J2 |
| LMO7-AS1 |
| FANCB |
| RRAD |
| PATJ |
| ZNF776 |
| ZNF805 |
| ENSG00000260997 |
| TMEM116 |
| PBX4 |
| PRRX2 |
| MORN3 |
| CCDC88C |
| MTA1 |
| ENSG00000263089 |
| ZBTB24 |
| ENSG00000259081 |
| LINC00607 |
| PDE6D |
| ZBED3-AS1 |
| BCORL1 |
| PLEKHH3 |
| CADM3 |
| ENSG00000290040 |
| ZNF182 |
| MYG1-AS1 |
| ZNF471 |
| TRIM73 |
| L3HYPDH |
| THBS2-AS1 |
| ENSG00000289397 |
| ENSG00000259408 |
| ENSG00000261114 |
| TGDS |
| ENSG00000289396 |
| H2BC21 |
| ENSG00000288059 |
| WDR5 |
| ENSG00000275764 |
| FZD4 |
| HSD17B7 |
| CR1L |
| TIRAP |
| CUX2 |
| HFE |
| ENSG00000260316 |
| ENSG00000225885 |
| ENSG00000289318 |
| STAMBP |
| HIF1AN |
| RPAP1 |
| SCRN1 |
| CDK10 |
| INE1 |
| ENSG00000287632 |
| ENSG00000256706 |
| GCOM1 |
| TRIM50 |
| LINC00921 |
| ASB11 |
| ENSG00000271011 |
| ENSG00000290793 |
| ENSG00000254556 |
| KCNG2 |
| GMEB2 |
| ENSG00000267082 |
| LETM1 |
| ENSG00000234139 |
| ABCA7 |
| CLDN15 |
| LINC00926 |
| ENSG00000289854 |
| CABP4 |
| YTHDF3-DT |
| LINC02803 |
| ENSG00000289948 |
| IGHV1-18 |
| ZFPM2-AS1 |
| ENSG00000289425 |
| ATL1 |
| ADCY4 |
| HS3ST1 |
| ENSG00000270022 |
| ENSG00000258860 |
| AJM1 |
| PDE7B-AS1 |
| LGI4 |
| ENSG00000286488 |
| FLRT2 |
| FOXRED1 |
| MCMDC2 |
| CHCT1 |
| FAM53B-AS1 |
| MLLT1 |
| ACKR1 |
| CDK18 |
| FAM81A |
| JAKMIP3 |
| ARSK |
| ENSG00000273151 |
| PLVAP |
| PGM3 |
| BAMBI |
| PPP5C |
| GDPD3 |
| CSNK1G2 |
| E4F1 |
| ENSG00000271797 |
| PEBP4 |
| RAB38 |
| ENSG00000264666 |
| NOTCH3 |
| EPOP |
| ENSG00000257715 |
| NATD1 |
| FCRLB |
| OPA1-AS1 |
| HSF2 |
| MRNIP-DT |
| C1orf220 |
| CDKN3 |
| RANBP3-DT |
| ZNF469 |
| MAN2A2 |
| SCUBE3-AS1 |
| ENSG00000270504 |
| ZNF77 |
| ENSG00000285639 |
| ENSG00000288743 |
| SYCP3 |
| DNAH12 |
| LERFS |
| SMOC2 |
| VEGFD |
| AKR1C1 |
| P3H3 |
| MIR2052HG |
| CDK1 |
| TCF4-AS1 |
| ALG10B |
| RASD1 |
| SCTR |
| ATP13A4 |
| ENSG00000278668 |
| ACAP3 |
| LY6G6C |
| DNM1P35 |
| INTS6-AS1 |
| IGHV3-23 |
| CCN5 |
| TRIM65 |
| ENSG00000217702 |
| PGAP2 |
| NEIL1 |
| RACGAP1 |
| ENSG00000286416 |
| FAM78B |
| PLD4 |
| FAM171A1 |
| SEPTIN7P9 |
| C5orf64 |
| MYPOP |
| ENSG00000285696 |
| PSMD14-DT |
| ENTPD6 |
| PATL1-DT |
| ENSG00000287226 |
| WDFY3-AS2 |
| P2RY8 |
| ANGPTL1 |
| EHD2 |
| ZNF699 |
| C1QTNF3 |
| ENSG00000203325 |
| PARM1 |
| GRK4 |
| OXCT1 |
| ENSG00000231918 |
| RDH13 |
| IGHV1-69D |
| VASH1 |
| C14orf132 |
| MTERF3 |
| ZNF653 |
| ENSG00000286035 |
| ZDHHC11 |
| CFAP20DC |
| AS3MT |
| PET117 |
| FGF12 |
| ENSG00000257181 |
| SLC26A7 |
| WDR3 |
| CPAMD8 |
| GPAT2 |
| S1PR3 |
| PSMD7-DT |
| ZNF28 |
| MAP1B |
| FBXO25 |
| ENSG00000240710 |
| ENSG00000289339 |
| ENSG00000250354 |
| ENSG00000286705 |
| NPEPPSP1 |
| TSPAN33 |
| MICALL1 |
| MPP7 |
| RUNDC1 |
| ENSG00000272667 |
| IGKV4-1 |
| RNF24 |
| NEURL2 |
| ENSG00000270210 |
| HFM1 |
| LINC02966 |
| NUF2 |
| SGO2 |
| SHMT1 |
| LLGL2 |
| ENSG00000268650 |
| TDRKH |
| CBX5 |
| PPP1R1A |
| ENSG00000272970 |
| JAG2 |
| PIK3CD-AS2 |
| ENSG00000270087 |
| NAGS |
| CLIP3 |
| FOXN3-AS1 |
| LYSMD4 |
| P3H4 |
| ENSG00000289138 |
| CCDC62 |
| TXNL4B |
| ENSG00000284428 |
| ENSG00000274341 |
| SCOC-AS1 |
| ST3GAL4 |
| TMPRSS9 |
| C6orf163 |
| EFHC2 |
| SLC22A4 |
| SYCP2 |
| PRRT3-AS1 |
| FAM66C |
| CMTM8 |
| MSANTD4 |
| SH2B2 |
| FAM131A |
| GPR157 |
| ENSG00000289227 |
| VASH1-AS1 |
| ENSG00000259659 |
| HLX-AS1 |
| LINC01426 |
| MORC4 |
| LINC01252 |
| IMPA2 |
| LINC02427 |
| TMEM147-AS1 |
| IL1R2 |
| ENSG00000291032 |
| ENSG00000267364 |
| GIPC2 |
| ENSG00000262810 |
| ENSG00000229628 |
| SYDE2 |
| LINC03033 |
| ADGRA2 |
| SIGLEC16 |
| LINC02541 |
| ZNF613 |
| EXTL2 |
| ZMYM4-AS1 |
| SELP |
| AK8 |
| LINC00520 |
| SUSD5 |
| TFR2 |
| ETAA1 |
| PPP1R3B-DT |
| PPP1R3F |
| ZNF286A |
| LINC02021 |
| LINC02997 |
| ENSG00000256433 |
| TMEM191C |
| ENSG00000286254 |
| HCG20 |
| ENSG00000284675 |
| PLCD4 |
| ZNF852 |
| B3GAT2 |
| ENSG00000289056 |
| ENSG00000288907 |
| DGKI |
| YPEL1 |
| CCDC90B-AS1 |
| C4orf46 |
| MEDAG |
| ZNF20 |
| ENSG00000286883 |
| REXO4 |
| LINC02688 |
| TRAF2 |
| ENSG00000249171 |
| ENSG00000286753 |
| MEAK7 |
| AZIN2 |
| ENSG00000258082 |
| ENSG00000269968 |
| ENSG00000259138 |
| GOLGA8N |
| RIOX2 |
| ENSG00000289283 |
| LHFPL5 |
| WASHC1 |
| DHCR7 |
| ENSG00000267096 |
| IDO1 |
| ENSG00000275494 |
| C9orf24 |
| ENSG00000272668 |
| ATP7A |
| ENSG00000289052 |
| STX1A |
| LBX2 |
| KIF15 |
| ENSG00000259735 |
| ALDH8A1 |
| DIAPH3 |
| RAVER2 |
| PDK1 |
| LIG1 |
| DNAL1 |
| ENSG00000272518 |
| CLDN11 |
| LRP11 |
| DNMBP-AS1 |
| ENSG00000285730 |
| TADA1 |
| ENSG00000259269 |
| PPP1R3G |
| LOXL1 |
| ENSG00000289689 |
| ENSG00000236924 |
| C8orf76 |
| APOL2 |
| WDCP |
| IGHG1 |
| SNHG19 |
| ENSG00000290916 |
| AIM2 |
| KMT5A |
| ZNF681 |
| ENSG00000234584 |
| ZNF879 |
| MLXIPL |
| ZNF687-AS1 |
| RAB30 |
| ZSCAN21 |
| CD28 |
| TMOD1 |
| ENSG00000285634 |
| FCER2 |
| SRGAP2-AS1 |
| EGFL8 |
| ENSG00000289562 |
| PKD2L2 |
| GNA15-DT |
| C2CD2L |
| IHO1 |
| FSIP1 |
| ENSG00000287093 |
| DPH7 |
| C1QTNF6 |
| SETD6 |
| ENSG00000286813 |
| CTNNBIP1 |
| DYSF |
| NNT-AS1 |
| AARSD1 |
| FAHD2B |
| PBLD |
| ZNF322 |
| ENSG00000262050 |
| ENSG00000289523 |
| RAPGEF3 |
| AKAP1 |
| GALNS |
| GPAT4-AS1 |
| MPZL3 |
| BMP6 |
| ENSG00000251675 |
| ZNF382 |
| ENSG00000267042 |
| HSCB |
| ENSG00000204850 |
| PSPH |
| SF1-DT |
| TBC1D13 |
| SOCS2-AS1 |
| SLFN13 |
| DNA2 |
| ENSG00000260563 |
| MIOS-DT |
| CPS1 |
| ENSG00000226816 |
| DEPP1 |
| MICOS10-DT |
| USP18 |
| ENSG00000289084 |
| ENSG00000289486 |
| IGLV1-51 |
| C4BPB |
| ENSG00000288999 |
| ENSG00000277767 |
| TSEN15 |
| KDM4D |
| FLT3 |
| ALDOC |
| DNAJB5 |
| ENSG00000260917 |
| MYBBP1A |
| FAM234B |
| MX1 |
| PRICKLE4 |
| ZNF526 |
| MIR924HG |
| LINC02285 |
| LINC02723 |
| TRIM8-DT |
| GNPDA2 |
| KIRREL1 |
| ENSG00000289401 |
| ENSG00000258572 |
| CCNB3 |
| ENSG00000243305 |
| TSPAN7 |
| ENSG00000270127 |
| ZNF696 |
| ENSG00000259515 |
| ENSG00000261140 |
| SLC7A9 |
| ENSG00000283696 |
| ERBB2 |
| ENSG00000225806 |
| C3orf70 |
| PKN3 |
| ZSCAN29 |
| ENSG00000257475 |
| ENSG00000289005 |
| CDC42-IT1 |
| LAMA3 |
| APOM |
| HARS2 |
| SMG1P7 |
| ENSG00000260274 |
| STPG1 |
| ENSG00000269918 |
| RNF8 |
| HERC2P9 |
| ENSG00000289883 |
| PDK4-AS1 |
| SPATA6L |
| ENSG00000283078 |
| HMGN3-AS1 |
| TRIM3 |
| ENSG00000290995 |
| HOXB8 |
| DNER |
| DLX4 |
| CCDC81 |
| TTC5 |
| ENSG00000272688 |
| PLCH1 |
| MACROD1 |
| LOXL2 |
| ENSG00000287721 |
| ZNF502 |
| ENSG00000291232 |
| ARHGAP11A-DT |
| MOCS3 |
| IBA57 |
| TM4SF19-AS1 |
| FAM83H |
| KIF11 |
| ZNF607 |
| ENSG00000291258 |
| CPE |
| LNCTAM34A |
| TLDC2 |
| CNGA1 |
| PJVK |
| NXN |
| LGALS2 |
| JPT2 |
| DCAF4 |
| SYT9 |
| LRRC20 |
| CCDC122 |
| GOLGA6L9 |
| EID3 |
| CPEB1 |
| PODXL |
| C10orf95-AS1 |
| TMEM231 |
| NHLRC1 |
| GABRA3 |
| TMEM185A |
| KCNN3 |
| CLDN5 |
| SH3BGRL2 |
| HLA-F-AS1 |
| ENSG00000278376 |
| PKD2L1 |
| ENSG00000255410 |
| GCC2-AS1 |
| GOLPH3-DT |
| CCDC194 |
| ENSG00000289928 |
| HCN2 |
| MC1R |
| CENPO |
| ENSG00000287562 |
| ST20 |
| LCAT |
| FITM2 |
| MINPP1 |
| CILK1 |
| IGSF22 |
| DCDC1 |
| MEX3D |
| WDR7-OT1 |
| IL11RA |
| MINDY1 |
| C1QTNF4 |
| TMEM202-AS1 |
| ZNF208 |
| ENSG00000287691 |
| C17orf97 |
| MAP3K12 |
| LINC00519 |
| ZNF114 |
| PCNX4-DT |
| METTL17 |
| SASS6 |
| ZNF836 |
| ENSG00000257526 |
| ENSG00000273017 |
| KIF4A |
| RAMP3 |
| ENSG00000268584 |
| ENSG00000286707 |
| C13orf46 |
| UBL7-DT |
| AKAP12 |
| KCNAB3 |
| ARHGAP5-AS1 |
| JDP2-AS1 |
| PASK |
| ZNF726 |
| DHRS12 |
| ABHD10 |
| PTK7 |
| GNRH1 |
| FHIP2B |
| HABP4 |
| DIO1 |
| ZNF554 |
| KANK4 |
| DLL1 |
| GALNT14 |
| BACE1-AS |
| ENSG00000285517 |
| RASAL3 |
| LINC01934 |
| MTCP1 |
| ENSG00000255031 |
| GARIN1A |
| LIG3 |
| PGBD4 |
| GGT5 |
| ENSG00000258646 |
| REXO5 |
| LINC02975 |
| DNAJC27-AS1 |
| ZSWIM9 |
| RAB44 |
| FBLN2 |
| SRSF12 |
| ENSG00000259345 |
| ZNF865 |
| LEKR1 |
| DNAJB5-DT |
| PLA2G7 |
| ENSG00000266877 |
| EIF3C |
| ADCK5 |
| KCNH4 |
| ENSG00000288880 |
| PCCB |
| BVES |
| ENSG00000291185 |
| LINC02680 |
| RC3H1-DT |
| ENSG00000226571 |
| ENSG00000272335 |
| PDLIM3 |
| ENSG00000288886 |
| LINC02904 |
| GRIN2C |
| BPNT1 |
| ENSG00000258982 |
| ZNF284 |
| LINC02641 |
| ENSG00000278932 |
| SLC1A2 |
| RSPO4 |
| TMC7 |
| KIAA0408 |
| HOXC4 |
| PYY |
| ENSG00000287733 |
| SLC9A3-AS1 |
| BACE2 |
| PLA2G4B |
| LIPC |
| ARAP3 |
| KAT2A |
| SUV39H1 |
| ENSG00000281021 |
| NUP37 |
| ENSG00000260409 |
| ENSG00000248774 |
| CROT |
| INCENP |
| SHF |
| ENSG00000283360 |
| PDK1-AS1 |
| SIAE |
| ENSG00000286366 |
| MIR3677HG |
| POFUT2 |
| RAB11FIP4 |
| SHE |
| ENSG00000260927 |
| TAF5 |
| ENSG00000291047 |
| CLEC12B |
| HELLS |
| HARBI1 |
| LRRC49 |
| PPP2R5D |
| SIGLEC5 |
| VILL |
| LINC01414 |
| MYO5C |
| LCA5 |
| ACTN2 |
| DKK2 |
| CDAN1 |
| CPOX |
| ENSG00000238009 |
| ZNF444 |
| CENATAC-DT |
| LRRK2-DT |
| ATP6V1FNB |
| ENSG00000290758 |
| NINJ2 |
| OLFML2B |
| ZNF101 |
| ERCC4 |
| RPUSD4 |
| OIP5 |
| ENSG00000267283 |
| ATRNL1 |
| RBM26-AS1 |
| P4HA3 |
| LINC01169 |
| ZNF710-AS1 |
| TIMELESS |
| LINC02698 |
| ENSG00000290937 |
| LIMD1-AS1 |
| CYP46A1 |
| LINC02916 |
| MUSK |
| HPS1-AS1 |
| KMT5C |
| GALNT3 |
| MHENCR |
| CCL8 |
| BEND3 |
| INSIG1-DT |
| TREML1 |
| RBM33-DT |
| CACNB1 |
| ENSG00000288111 |
| CCNB1 |
| DDO |
| PPP1R32 |
| ACE |
| TPD52L1 |
| LINC03036 |
| CHAF1A |
| TIPIN |
| NUMBL |
| ZNF550 |
| FCER1A |
| ALOX12P2 |
| FAM85B |
| LAMB2 |
| SCARF2 |
| CCDC175 |
| ENSG00000267765 |
| H2AZ1-DT |
| ENSG00000286525 |
| RASSF8-AS1 |
| ENSG00000291225 |
| COL23A1 |
| SEPHS1 |
| MYL6B-AS1 |
| C18orf54 |
| TC2N |
| LIX1L-AS1 |
| POMZP3 |
| ENSG00000288983 |
| LTB4R |
| SYP |
| NECTIN4 |
| FAM53A |
| LINC00310 |
| PKDCC |
| ACBD4 |
| NT5E |
| KNL1 |
| AP4B1 |
| ENSG00000287865 |
| ENSG00000275180 |
| DSCAS |
| MIX23 |
| SH2D4B |
| PSMG3-AS1 |
| BTC |
| NQO1-DT |
| LRRC34 |
| TCTN2 |
| PHF7 |
| G0S2 |
| ENSG00000289272 |
| SCD5 |
| C10orf143 |
| CAPS |
| PPM1F-AS1 |
| RUSC1-AS1 |
| ABCB9 |
| CHMP3-AS1 |
| CD248 |
| SHROOM3 |
| ENSG00000288093 |
| ZNF470 |
| LINC02306 |
| DKK3 |
| PFKP |
| DDN-AS1 |
| FAAH |
| C12orf60 |
| TUBA8 |
| CNTN5 |
| ENTR1 |
| MATN1 |
| PLAC8L1 |
| HAUS5 |
| ENSG00000289058 |
| NEK8 |
| ZNF829 |
| REC8 |
| ENSG00000247679 |
| ADPRHL1 |
| TYRO3 |
| RIMS3 |
| GPR156 |
| RAMACL |
| MANEA-DT |
| NBPF25P |
| ENSG00000269044 |
| CNTNAP4 |
| SNHG10 |
| ENSG00000272501 |
| GLIPR1L2 |
| FLVCR1-DT |
| ENSG00000291011 |
| UBA5 |
| ZBTB9 |
| SLAIN1 |
| MSANTD2-AS1 |
| NPW |
| ARHGEF35 |
| ABL2 |
| INTS6 |
| HIVEP2 |
| HSPH1 |
| SPAG9 |
| MIR23AHG |
| SIK3 |
| FNIP2 |
| B4GALT1 |
| ADAM17 |
| HIVEP1 |
| CLIC4 |
| ARIH1 |
| DENND4A |
| TSC22D2 |
| ICAM1 |
| SESTD1 |
| DNAJB6 |
| ATP13A3 |
| IRAK2 |
| TANK |
| BAZ1A |
| MCL1 |
| NFAT5 |
| DENND5A |
| AFF4 |
| ELL2 |
| GPBP1 |
| FOXO3 |
| RYBP |
| CRY1 |
| MAPK6 |
| CPEB4 |
| MCTP1 |
| MAN1A1 |
| ETS2 |
| ALCAM |
| LINC01619 |
| EIF2AK3 |
| ZC3H12C |
| IGKC |
| SLC38A2 |
| PDE4B |
| KIF1B |
| TIPARP |
| NOTCH2NLC |
| PPP1R10 |
| UBAP1 |
| FAM107B |
| IGHG4 |
| LINC-PINT |
| EMP1 |
| FBXO11 |
| GCH1 |
| LCP2 |
| USP12 |
| REL |
| NFE2L2 |
| RASGEF1B |
| WTAP |
| TLE4 |
| PHLPP1 |
| JARID2 |
| MAPKAPK2 |
| KMT2E |
| GLS |
| LPXN |
| ATF6 |
| HNRNPC |
| NFKBID |
| STK40 |
| TCF7L2 |
| ARL8B |
| DDX3X |
| STK10 |
| RND3 |
| ETF1 |
| SIPA1L1 |
| PTPN1 |
| ESYT2 |
| NABP1 |
| PPARG |
| PSEN1 |
| ATP2B1 |
| KYNU |
| QKI |
| P4HA1 |
| NUFIP2 |
| CHD2 |
| CLK1 |
| BAG3 |
| ATP1B3 |
| PCF11 |
| PELI1 |
| ITGA5 |
| SFPQ |
| GPR132 |
| HNRNPU |
| OXSR1 |
| FGD4 |
| ELOVL5 |
| YME1L1 |
| ELF1 |
| RAB7A |
| TP53BP2 |
| IFRD1 |
| RIPK2 |
| SLC27A4 |
| HSPD1 |
| HSP90AA1 |
| AKAP13 |
| PAPSS2 |
| RTN4 |
| MB21D2 |
| TNFRSF10B |
| ARID5B |
| PDE4C |
| EHD1 |
| ACKR3 |
| DOT1L |
| LITAF |
| APLP2 |
| TAOK3 |
| DHX34 |
| EZH2 |
| KCNQ1OT1 |
| ENSG00000260911 |
| ENSG00000286062 |
| CD44 |
| LIMS1 |
| JDP2 |
| CTNNB1 |
| SPRED2 |
| CRYM |
| YTHDF3 |
| SNX9 |
| ZHX2 |
| RCOR1 |
| ACSL1 |
| PNPLA8 |
| LONRF3 |
| CLN8 |
| ACTR3-AS1 |
| ANKRD28 |
| NUMB |
| IGLC2 |
| CFAP161 |
| CCNL1 |
| FNDC3A |
| CSGALNACT2 |
| TGFB1 |
| ERN1 |
| CFLAR |
| ABTB2 |
| SERPINB9 |
| KIF13B |
| KANK1 |
| KMO |
| AZIN1 |
| MBNL1 |
| RAPGEF2 |
| C5AR2 |
| ANPEP |
| HECA |
| PITPNB |
| EIF5 |
| GNA13 |
| LRRC23 |
| WNT2B |
| UBE2E1 |
| PLEK |
| MYO1E |
| AHCYL1 |
| REV3L |
| ENSG00000249870 |
| MCF2L2 |
| CD300E |
| NCF1B |
| PPP1R13B |
| METTL7A |
| NEDD9 |
| KPNA4 |
| KANSL1L |
| MYLIP |
| CPEB2 |
| CREB5 |
| SGMS2 |
| PPARD |
| SLC25A33 |
| ACSL5 |
| SERTAD2 |
| ZFX |
| ENSG00000253736 |
| RLF |
| ENSG00000284797 |
| ERF |
| OSBPL8 |
| CYLD |
| FOXO1 |
| RAB21 |
| EIF1AX |
| RASGRP3 |
| PPIEL |
| GAS2L3 |
| B4GALT5 |
| FOSL2 |
| RELB |
| IL3RA |
| CCNH |
| RIPK2-DT |
| IQCN |
| AQP9 |
| ENSG00000223725 |
| CDC42EP3 |
| AGPAT4 |
| PDGFB |
| TET2 |
| UBE2H |
| ZC3HAV1 |
| AGAP3 |
| UBAC2 |
| UST |
| ENSG00000256448 |
| GAREM1 |
| MPDU1 |
| CHORDC1 |
| RNF19B |
| IRF2BP2 |
| ARL5B |
| FLOT1 |
| SCML1 |
| MARCHF3 |
| CD109 |
| BTG3 |
| KLF9 |
| LINC00877 |
| SLC16A1 |
| DUSP6 |
| HIPK4 |
| LINC01033 |
| ENSG00000258216 |
| UBTD2 |
| ADNP2 |
| HSPA9 |
| ARRDC3 |
| SLC12A2 |
| CCNYL1 |
| SAMSN1 |
| TAF4B |
| ENSG00000289085 |
| BID |
| FGFR1 |
| SLC30A4-AS1 |
| FCGR2A |
| MAP2K1 |
| AMD1 |
| ENSG00000285693 |
| GTF2B |
| SEC24A |
| GNPTG |
| LINC02296 |
| MORF4L2 |
| ENSG00000235978 |
| CLIC2 |
| RENBP |
| IRAK3 |
| NUP98 |
| PLK3 |
| GK |
| LUZP1 |
| THUMPD3-AS1 |
| ENSG00000290823 |
| RFX2 |
| BZW1 |
| TSPYL2 |
| VPS37B |
| STIP1 |
| PKNOX1 |
| SERPINE1 |
| IRS2 |
| ABHD5 |
| NFATC1 |
| ENSG00000251194 |
| ERO1B |
| MIR22HG |
| HAPSTR1 |
| ZBTB10 |
| IRF1 |
| NFKB2 |
| FAM241A |
| SP4 |
| GSK3A |
| BTBD19 |
| TSPAN15 |
| ATP2C1 |
| NOCT |
| DUSP16 |
| LIMASI |
| FAM53C |
| SIRT1 |
| KLHL21 |
| CYP1B1 |
| FAM210A |
| ITGAX |
| GBE1 |
| ATP1B1 |
| PIM3 |
| LONRF1 |
| PRDM1 |
| VPS36 |
| ENSG00000288827 |
| SLC35F2 |
| LAP3 |
| LINC01588 |
| CRADD |
| MN1 |
| CHML |
| LRRC8A |
| ZBTB43 |
| ITPRIP |
| MRAS |
| FEM1C |
| ATP13A3-DT |
| IGHG3 |
| ENSG00000289472 |
| TGFB2 |
| MXD1 |
| EPAS1 |
| CA13 |
| LINC00641 |
| SLC9A1 |
| RCC1 |
| ENSG00000253295 |
| PMF1 |
| ETV3 |
| MAMLD1 |
| MAFK |
| C11orf96 |
| NUP210L |
| ELOVL7 |
| SH3BP5 |
| OLMALINC |
| RP2 |
| SLC20A1 |
| OPN3 |
| TRIM69 |
| ERLEC1 |
| USP53 |
| SARNP |
| TICAM1 |
| ENSG00000188897 |
| NCR3LG1 |
| STK38L |
| RAB26 |
| TXNDC16 |
| PLAU |
| ZNF697 |
| MAFG |
| ITPR1 |
| RNF103 |
| IFITM10 |
| ENSG00000262202 |
| SMIM7 |
| MAP4K4 |
| PLA2G6 |
| APIP |
| SYNPO2 |
| SYNJ2 |
| RDH10 |
| IL1RAP |
| NFE2L3 |
| CBX4 |
| SLC5A3 |
| CKLF |
| ADA |
| ARFGAP3 |
| INTS6L |
| TWSG1 |
| ENSG00000286248 |
| ENSG00000266313 |
| RBKS |
| ZFP91 |
| NDUFV3 |
| PCSK5 |
| TENT5C |
| ENSG00000255847 |
| DECR1 |
| EDEM1 |
| CLEC1A |
| ZFAND2A |
| ZMIZ1-AS1 |
| PIK3R3 |
| ZSWIM7 |
| KBTBD8 |
| IFFO1 |
| HMGCS1 |
| XPA |
| CNPY2 |
| MPV17 |
| DENR |
| ZSWIM4 |
| C2orf92 |
| ALPK3 |
| ENSG00000265975 |
| JMJD6 |
| LINC00243 |
| TMEM243 |
| RBBP8 |
| ENSG00000285091 |
| LINC00964 |
| SPDYA |
| ANKRD33B |
| GPR176 |
| RUVBL2 |
| PIGA |
| SMCO4 |
| KLHL15 |
| IGLC3 |
| HSPA1L |
| AGGF1 |
| ZNF503 |
| CATSPERG |
| SLC39A12 |
| PURB |
| FBXO9 |
| LRRC8B |
| ENSG00000250519 |
| ENSG00000273221 |
| WHRN |
| SCIMP |
| KIRREL2 |
| EMC1-AS1 |
| TIMM23 |
| ENSG00000229044 |
| MLX |
| ZC3H4 |
| UROS |
| KHDRBS3 |
| CPNE9 |
| SLC19A2 |
| USP38 |
| ENSG00000289254 |
| SMARCB1 |
| LINC02611 |
| ENSG00000240553 |
| ENSG00000228037 |
| ENSG00000291233 |
| CARD14 |
| ARHGAP26-AS1 |
| PLEKHA6 |
| ZBTB21 |
| ODF3B |
| SAMD12 |
| SERPINE3 |
| ENSG00000290441 |
| LINC01283 |
| ACOT13 |
| KLHDC3 |
| TINCR |
| MFAP5 |
| MIR9-1HG |
| PCP4L1 |
| LINC02150 |
| TCHH |
| DYNLL2 |
| BCCIP |
| FLJ40194 |
| ENSG00000269902 |
| LINC02458 |
| ENSG00000225450 |
| ARL11 |
| TBC1D7 |
| EPB41L5 |
| PDE2A |
| HK2 |
| ENSG00000290241 |
| CACNG7 |
| TCOF1 |
| KLF2-DT |
| DPEP2 |
| NFU1 |
| SDHAF2 |
| PDZRN4 |
| ARG2 |
| TANK-AS1 |
| METTL22 |
| VIL1 |
| BHLHE41 |
| TMEM220 |
| AMZ1 |
| ZNF580 |
| ENSG00000225339 |
| ARPIN |
| ENSG00000289017 |
| PLGRKT |
| C4orf47 |
| MRPS35 |
| MIR17HG |
| SEMA6B |
| TLE1 |
| KRTAP5-AS1 |
| RPP38 |
| JAG1 |
| LINC02345 |
| ENSG00000287044 |
| NT5DC3 |
| ENSG00000250917 |
| MLF1 |
| ARMC10 |
| KLRD1 |
| RASSF5 |
| HDAC3 |
| HAUS8 |
| USF1 |
| ENSG00000290051 |
| PDCD2 |
| ENSG00000263923 |
| TAF11 |
| TMEM63B |
| ENSG00000271204 |
| CTNNAL1 |
| GBA1 |
| IFT52 |
| ENSG00000254459 |
| ENSG00000233912 |
| SNHG9 |
| RNF122 |
| H2BC18 |
| PSMG4 |
| ENSG00000278095 |
| FN3K |
| ENSG00000261544 |
| ENSG00000261888 |
| LDHC |
| SIX3 |
| LSMEM2 |
| SESN2 |
| ALKBH3 |
| CORO2A |
| LSMEM1 |
| APOD |
| ENSG00000257258 |
| CD300A |
| CD44-DT |
| PNLDC1 |
| ENSG00000288928 |
| AKAP13-AS1 |
| LINC01637 |
| L1TD1 |
| SUPT5H |
| CCDC97 |
| ENSG00000269906 |
| NRROS |
| PROX1 |
| SLC8A2 |
| PTGER2 |
| SDC4 |
| NTPCR |
| MEX3C |
| CCDC112 |
| HDHD5 |
| DYRK4 |
| FLNC-AS1 |
| ENSG00000236990 |
| ADAMTSL4-AS2 |
| TNFRSF9 |
| HSBP1L1 |
| CCL14 |
| APLP1 |
| NUDT4 |
| ENSG00000288612 |
| LDLR |
| GAPLINC |
| RP9 |
| LINC00339 |
| NANOS3 |
| IGHA1 |
| TPM2 |
| PREB |
| ENSG00000270019 |
| ENSG00000229021 |
| PKP2 |
| ENSG00000286314 |
| LHPP |
| LINC02137 |
| LINC01678 |
| ENSG00000288704 |
| DALRD3 |
| ARSI |
| ZNF780A |
| DHX16 |
| RNASEH1 |
| ENSG00000288033 |
| ENSG00000260196 |
| TTLL1-AS1 |
| HS3ST3A1 |
| LINC02901 |
| GPR137C |
| NT5C3B |
| ENSG00000235189 |
| SEMA3B |
| ENSG00000251598 |
| CDC42EP4 |
| ENSG00000287593 |
| PANX1 |
| ZNF585A |
| ENSG00000251393 |
| CADM4 |
| IRGQ |
| DYRK3 |
| ENSG00000255299 |
| ENSG00000290846 |
| RABEPK |
| C1orf131 |
| ZFPM1 |
| ENSG00000269807 |
| GSK3B-DT |
| LINC01602 |
| NDUFV2-AS1 |
| ENSG00000223711 |
| BORCS8 |
| AIMP2 |
| PLCXD1 |
| NFIL3 |
| ENSG00000287123 |
| ARMC12 |
| LINC00847 |
| SLC16A6 |
| HS3ST2 |
| ARL16 |
| C1orf50 |
| UBE2D3-AS1 |
| RMDN2-AS1 |
| ADCY3 |
| MIR4300HG |
| BTG3-AS1 |
| MRS2 |
| SNAPC1 |
| ZNG1E |
| DAND5 |
| RPUSD3 |
| RWDD3 |
| LINC02605 |
| CFAP58-DT |
| ENSG00000260257 |
| LINC01168 |
| PEMT |
| FAHD2A |
| NUDT2 |
| ENSG00000250069 |
| LINC02895 |
| SPSB1 |
| ENSG00000286850 |
| RP9P |
| C2orf74-DT |
| RRP12 |
| NBPF26 |
| CCN1 |
| ADGRV1 |
| ENSG00000286622 |
| NOP2 |
| LINC01344 |
| ENSG00000231873 |
| ENSG00000280195 |
| RASA4 |
| DHH |
| LINC01031 |
| ENSG00000289997 |
| ENSG00000290100 |
| CXCL5 |
| ENSG00000290735 |
| NR1D1 |
| SPATC1 |
| EIF2B1 |
| C2orf68 |
| ENSG00000290609 |
| NOPCHAP1 |
| SNHG26 |
| EHMT2 |
| LYRM1 |
| THOC3 |
| FXN |
| FADS3 |
| TMEM138 |
| CCDC137 |
| HSPA6 |
| TP53I3 |
| TBP |
| LINC00891 |
| COQ9 |
| TNFAIP8L3 |
| ENSG00000285646 |
| PPP1R27 |
| RDUR |
| BTN3A2 |
| CHRNE |
| TACO1 |
| DNAH17 |
| ENSG00000261460 |
| ENSG00000277482 |
| SLAMF8 |
| HDHD3 |
| RGPD1 |
| CENPT |
| ELP6 |
| HTT-AS |
| ENSG00000228401 |
| ANAPC2 |
| SIGLEC14 |
| ACER2 |
| SCIRT |
| ENSG00000287791 |
| GS1-24F4.2 |
| PARP16 |
| BATF |
| ENSG00000283573 |
| IDI2-AS1 |
| NEU4 |
| TIMM21 |
| ERAL1 |
| ENSG00000291178 |
| LINC01852 |
| TARBP2 |
| DCSTAMP |
| USP9Y |
| ENSG00000272625 |
| CT69 |
| KLRC4 |
| ENSG00000290008 |
| SCUBE2 |
| SLC2A5 |
| LINC00592 |
| CIAPIN1 |
| ABHD11 |
| BPHL |
| ENSG00000289726 |
| STARD10 |
| SPACDR |
| TOR2A |
| ENSG00000286256 |
| LAG3 |
| ITM2A |
| CSMD1 |
| USP12-DT |
| ENSG00000258077 |
| ENSG00000288995 |
| CAMK2N1 |
| CCDC163 |
| PTCH2 |
| MSANTD1 |
| MLLT11 |
| NYAP1 |
| GP6-AS1 |
| CIAO3 |
| ENSG00000287507 |
| JAML |
| ENSG00000274528 |
| GRIP1 |
| EAF1 |
| NME6 |
| EPHA4 |
| MYOC |
| PHKG1 |
| ENSG00000289977 |
| CHRNA5 |
| BASP1-AS1 |
| TMEM26-AS1 |
| PYGM |
| THAP8 |
| NOTCH2NLB |
| RND1 |
| TRAF3IP3 |
| ENSG00000248559 |
| HSD11B2 |
| CFAP45 |
| LINC02812 |
| SORD |
| IGLV1-40 |
| TRIM21 |
| IGSF22-AS1 |
| TCTN1 |
| ESPL1 |
| ZNF691 |
| PARD6G-AS1 |
| ENSG00000291107 |
| ENSG00000260177 |
| ENSG00000290019 |
| LINC01115.1 |
| ENSG00000286782 |
| CACHD1 |
| YAE1 |
| CYP27A1 |
| CRISPLD2 |
| NRARP |
| ENSG00000289198 |
| H2BC6 |
| ENSG00000285424 |
| ENSG00000235066 |
| B3GALNT1 |
| CABYR |
| ENSG00000289845 |
| AGMAT |
| ENSG00000289430 |
| SMIM47 |
| ENSG00000279317 |
| AATK |
| GSTM3 |
| EBPL |
| IZUMO1 |
| ZDHHC16 |
| TAS1R1 |
| ARFIP2 |
| ENSG00000265907 |
| TRAF1 |
| ZFAND3-DT |
| PPP1R13L |
| FCN1 |
| AGBL5 |
| PPP1R35-AS1 |
| DDX11 |
| ENSG00000286177 |
| HNRNPD-DT |
| ENSG00000287420 |
| NKRF |
| ZNF496-DT |
| LINC02728 |
| EXOC3-AS1 |
| ASB13 |
| MAB21L3 |
| GRAMD2A |
| ENSG00000273210 |
| CDC42BPG |
| ENSG00000251259 |
| SPAG16 |
| PI16 |
| AZU1 |
| LINC00880 |
| HCAR3 |
| ENSG00000270640 |
| ARMC5 |
| ENSG00000249309 |
| SARS2 |
| SNAI1 |
| FGF18 |
| LY6K |
| CSRP2 |
| ITGA10 |
| MLF1-DT |
| LINC02470 |
| LIF |
| YPEL3-DT |
| SIRT3 |
| TCF7 |
| LINC02482 |
| FOSL2-AS1 |
| ENSG00000286536 |
| DNAI2 |
| GPR84 |
| ENSG00000236754 |
| PPP1R15B-AS1 |
| LINC02330 |
| ARHGAP39 |
| ENSG00000285966 |
| ITPR2-AS1 |
| SNX32 |
| METRN |
| ENSG00000223969 |
| ENSG00000289988 |
| TM4SF1 |
| ARHGAP31-AS1 |
| TUBB3 |
| ENSG00000255946 |
| LINC01393 |
| PGA3 |
| LINC01841 |
| ENSG00000289312 |
| MAP3K5-AS2 |
| PPP1R36 |
| ADAMTS1 |
| H1-3 |
| SPART-AS1 |
| GRK7 |
| ATP8B5P |
| NDUFAF1 |
| MYADM-AS1 |
| ENSG00000289456 |
| ENSG00000253181 |
| ENSG00000253256 |
| NIBAN3 |
| PRR7 |
| MZF1-AS1 |
| LINC00324 |
| ENSG00000286186 |
| ITPKC |
| ENSG00000276256 |
| CD300LB |
| ENSG00000290791 |
| DDX43 |
| LINC01410 |
| ENSG00000289061 |
| CCDC152 |
| NOP14 |
| PTOV1-AS1 |
| YIPF7 |
| SLC10A1 |
| ISG20 |
| ENSG00000229337 |
| C20orf96 |
| CLCF1 |
| LILRA5 |
| FGFBP2 |
| THAP9 |
| BNIP1 |
| SLC41A1 |
| LINC02988 |
| IDO2 |
| SPATA2L |
| MCF2 |
| ENSG00000287738 |
| ENSG00000278000 |
| ENSG00000261026 |
| ENSG00000259033 |
| ENSG00000267737 |
| TKTL1 |
| GAST |
| LSP1P5.1 |
| LNCATV |
| KCNA3 |
| SFRP2 |
| SCAT1 |
| ENSG00000203644 |
| TMEM216 |
| ENSG00000287200 |
| ENSG00000251139 |
| ENSG00000288703 |
| UNC5D |
| ENSG00000189229 |
| ELAC1 |
| ENSG00000287078 |
| ZNF71 |
| ENSG00000214719 |
| PECR |
| ENSG00000285688 |
| CTRC |
| PDE6B |
| ENSG00000271882 |
| CENPS |
| ZFX-AS1 |
| MBIP |
| SPTBN4 |
| LINC01226 |
| HERC2P3 |
| SPOUT1 |
| CENPL |
| AHNAK2 |
| TPSB2 |
| C19orf47 |
| TRAF4 |
| ENSG00000232807 |
| SDHAF4 |
| ENSG00000253593 |
| GIRGL |
| STIL |
| ZNF407-AS1 |
| LINC02019 |
| ENSG00000287070 |
| ENSG00000272030 |
| SFRP4 |
| ITGB1-DT |
| EIF5AL1 |
| PRR19 |
| C5orf63 |
| RAD51AP1 |
| ENSG00000287682 |
| STRADB |
| IL23A |
| ENSG00000274508 |
| IL18BP |
| ENSG00000290057 |
| ZNF419 |
| DUSP5-DT |
| SPRED3 |
| BTG2-DT |
| GDF11 |
| RGS18 |
| CD48 |
| UTP14A |
| AHRR |
| ENSG00000285710 |
| ENSG00000225032 |
| ENSG00000276651 |
| ENSG00000289865 |
| DSCAM |
| DUS4L |
| ASRGL1 |
| ENSG00000242282 |
| MAP7D3 |
| FAM200A |
| MILIP |
| CRABP2 |
| CMPK2 |
| ZNF181 |
| ENSG00000291131 |
| GDF9 |
| ENSG00000280206 |
| ENSG00000230532 |
| COQ6 |
| KCNK7 |
| IL1RL2 |
| MIDEAS-AS1 |
| ENSG00000224794 |
| RASIP1 |
| FOXC1 |
| RIPOR2 |
| IDH2-DT |
| CTSV |
| TIMM29 |
| TUBGCP6 |
| BATF2 |
| HEXIM2 |
| GBP5 |
| GAS8 |
| TMEM25 |
| SLC25A14 |
| MYOSLID |
| CD22 |
| PXN-AS1 |
| ANKRD29 |
| CCL24 |
| PCYOX1L |
| ITGB2-AS1 |
| PTPRG-AS1 |
| SMG1-DT |
| GK-AS1 |
| ENSG00000288794 |
| TTLL1 |
| ENSG00000289949 |
| ADPGK-AS1 |
| TMEM177 |
| SNHG11 |
| BFSP1 |
| ENSG00000289424 |
| ATRIP |
| IRF2-DT |
| DNAI7 |
| ALKBH6 |
| ENSG00000213062 |
| CD1D |
| SYT15-AS1 |
| PSD |
| SPATA12 |
| SOCS3-DT |
| VMAC |
| EPHB3 |
| MAP1LC3C |
| ENSG00000286138 |
| ENSG00000289589 |
| LMTK3 |
| SREBF2-AS1 |
| ENSG00000278133 |
| GLIDR |
| GEMIN2 |
| CDPF1 |
| TMEM238 |
| CHRFAM7A |
| AKAP1-DT |
| ENSG00000273329 |
| ENSG00000286549 |
| SIAH2-AS1 |
| DTD2 |
| LMNB1-DT |
| ENSG00000291262 |
| METTL2A |
| C3orf20 |
| ECSCR |
| OPRM1 |
| GIHCG |
| TNFSF15 |
| LINC02551 |
| APC2 |
| ENSG00000234810 |
| ENSG00000284615 |
| FAM186B |
| BMP2K-DT |
| ZNF671 |
| NCS1 |
| ZNF841 |
| NUDT7 |
| IGHV3-21 |
| ENSG00000263272 |
| GDF15 |
| SAXO1 |
| ENSG00000290074 |
| GSTM2 |
| ADM5 |
| SLC17A7 |
| EID2B |
| C17orf114 |
| ENSG00000288009 |
| SCAMP1-AS1 |
| DNAAF1 |
| ENSG00000290122 |
| ELF3-AS1 |
| ENSG00000291149 |
| ZNF232 |
| ZSCAN31 |
| MAP7D2 |
| ENSG00000258820 |
| OASL |
| NID1 |
| CFAP251 |
| PAOX |
| ENSG00000290872 |
| NLGN3 |
| TUT1 |
| MYH7B |
| LIN7B |
| BSG-AS1 |
| ENSG00000237531 |
| ENSG00000233230 |
| C16orf86 |
| LINC02869 |
| ZNF749 |
| ENSG00000287351 |
| GTF2IP1 |
| ENSG00000268798 |
| TOMM20L-DT |
| C17orf75 |
| LAMB3 |
| ENSG00000245025 |
| CT70 |
| RNASEH1-DT |
| CYLD-AS1 |
| DRAIC |
| N6AMT1 |
| ECHDC3 |
| ICAM3 |
| ENSG00000259945 |
| LINC01703 |
| NUDT6 |
| FCHSD1 |
| ZNF816 |
| SLC2A11 |
| MAPT |
| RBM34 |
| GPRC5D-AS1 |
| XRCC3 |
| LINC02940 |
| NKD1 |
| PARP3 |
| ENSG00000260136 |
| RWDD2B |
| TFAP4 |
| ENSG00000274922 |
| PTX3 |
| DNAJC5B |
| LGALS4 |
| ENSG00000289050 |
| ENSG00000261324 |
| ENSG00000259760 |
| CDA |
| ASF1B |
| SPRY2 |
| ZNF658 |
| C16orf95 |
| ENSG00000273226 |
| LINC00824 |
| REL-DT |
| ENO3 |
| ENSG00000291231 |
| UPK3BL2 |
| DNAJC28 |
| ENSG00000260912 |
| ELL3 |
| HTRA3 |
| CHST7 |
| ENSG00000286952 |
| ZNF287 |
| ENSG00000291180 |
| SLC6A8 |
| NECAB3 |
| HILPDA |
| ENSG00000250994 |
| ENSG00000284999 |
| B9D1 |
| ZNF473 |
| MIR4713HG |
| FALEC |
| USP46-DT |
| HDAC4-AS1 |
| ZNF583 |
| ZNF485 |
| DUXAP8 |
| ZSCAN2 |
| SPAG5-AS1 |
| SLC22A18AS |
| SNX9-AS1 |
| CLCN2 |
| ENSG00000261512 |
| TNFRSF10C |
| MIEF2 |
| ZNF517 |
| GSTT2B |
| ZNF764 |
| FAAP24 |
| SLC9A5 |
| ENSG00000287878 |
| BCDIN3D |
| ULK4P3 |
| ZNF582 |
| ENSG00000290853 |
| SIDT1 |
| PEX11A |
| ENSG00000285650 |
| LINC01554 |
| SCX |
| CHEK1 |
| ENSG00000228395 |
| PHOSPHO2 |
| ENSG00000278002 |
| MINCR |
| DIRAS1 |
| LBHD1 |
| ENSG00000260267 |
| BAALC |
| ENPP6 |
| FAM86EP |
| KLHL25 |
| ENSG00000291186 |
| SGPP2 |
| RWDD2A |
| SLC38A4 |
| ENSG00000286964 |
| ENSG00000290457 |
| ACAP1 |
| LOXL2-AS1 |
| ZNF530 |
| GPAT3 |
| ENSG00000247853 |
| PCCA-DT |
| SDCBP2 |
| ZNF542P |
| PLEKHF1 |
| EDNRB-AS1 |
| MNS1 |
| ENSG00000236507 |
| LNCARSR |
| ENSG00000286391 |
| DNAJC22 |
| TEX33 |
| TXNIP |
| ENSG00000253125 |
| RSPH10B2 |
| FBXO40 |
| ENSG00000289267 |
| TTLL10 |
| ENSG00000275719 |
| ENSG00000288531 |
| ENSG00000228033 |
| LINC01821 |
| ENSG00000285190 |
| SLC25A31 |
| COL9A3 |
| H2BC5 |
| ENSG00000238280 |
| EGFL7 |
| POLG2 |
| MYL11 |
| LINC02344 |
| MPEG1 |
| ENSG00000288724 |
| ENSG00000273133 |
| MS4A1 |
| CDC20B |
| PCDHAC2 |
| POLR2A |
| ENSG00000289041 |
| SERPING1 |
| HLX |
| ENSG00000288524 |
| FZD9 |
| GSEC |
| PNCK |
| ENSG00000288976 |
| MTRFR |
| ENSG00000288939 |
| FAM25G |
| ENSG00000282440 |
| NXT1-AS1 |
| LYVE1 |
| SLC2A14 |
| ENSG00000225057 |
| RFESD |
| PDK4 |
| MRC1 |
| WWP1-AS1 |
| CTR9 |
| H4C16 |
| MSLN |
| ENSG00000288929 |
| ENSG00000290123 |
| IDI1 |
| ENSG00000289010 |
| IL11 |
| ENSG00000288956 |
| ENSG00000272558 |
| GNAL |
| GARIN4 |
| CD209 |
| BZW1-AS1 |
| LINC02810 |
| HYKK |
| RTKN2 |
| ENSG00000285668 |
| ENSG00000290097 |
| ENSG00000291006 |
| TOB1-AS1 |
| CAVIN2 |
| H2BC15 |
| ENSG00000288996 |
| ENSG00000286315 |
| GHET1 |
| DNAJC3-DT |
| ENSG00000287005 |
| DUSP3 |
| KLF7 |
| CDC37 |
| ARRB2 |
| AKR1A1 |
| STMP1 |
| PSMA6 |
| C1orf43 |
| LILRB4 |
| DNAJC15 |
| TCEA1 |
| PPP3R1 |
| WSB1 |
| VTI1B |
| EPB41L3 |
| WDR26 |
| IL6R |
| RHOQ |
| ABCA1 |
| RABGEF1 |
| ABI1 |
| GNB1 |
| ZNF207 |
| TUT7 |
| WNK1 |
| RAB1A |
| AHR |
| TMEM165 |
| GPCPD1 |
| CSNK1A1 |
| FPR3 |
| IQGAP1 |
| TNFAIP2 |
| HYCC1 |
| COX16 |
| UBE2R2 |
| STAG2 |
| MGP |
| BCAT1 |
| RAB8B |
| JAK1 |
| PNPLA2 |
| SRGAP2B |
| PHACTR1 |
| FKBP5 |
| MAP3K2 |
| TOP1 |
| ANKH |
| ATP6V1H |
| RSRP1 |
| STXBP2 |
| CD58 |
| TNFAIP8 |
| SLC25A19 |
| SERINC5 |
| SLC66A3 |
| USP36 |
| ILRUN |
| SLC30A7 |
| STT3B |
| GNPDA1 |
| CISD2 |
| COPA |
| CTNNA1 |
| CHKA |
| CHD1 |
| PLEKHM2 |
| NPC1 |
| CALHM2 |
| DYNC1H1 |
| SHARPIN |
| PSMD9 |
| SDHC |
| COMMD7 |
| IQSEC1 |
| SKIL |
| TRAPPC3 |
| BABAM1 |
| CPM |
| UBL3 |
| RANBP9 |
| BECN1 |
| IL6ST |
| C6orf62 |
| LATS2 |
| ANKRD36B |
| SCNM1 |
| ATP2A2 |
| DRAM1 |
| RBM39 |
| C9orf72 |
| AP1G1 |
| COPS5 |
| CORO1C |
| RNF141 |
| MAP3K8 |
| NPTN |
| REXO2 |
| CREBRF |
| IPMK |
| MBP |
| ZFAND6 |
| ATF5 |
| MRPS18B |
| ZDHHC24 |
| CYRIA |
| SNTB1 |
| MBD2 |
| HDDC2 |
| CCDC25 |
| EBAG9 |
| TMEM183A |
| TBC1D15 |
| TMEM18 |
| TACC1 |
| PDCD6IP |
| PTGES2 |
| MAPRE2 |
| CRELD2 |
| SCCPDH |
| STK24 |
| TM9SF3 |
| SIN3A |
| PPTC7 |
| DHX15 |
| HNRNPD |
| SLC6A6 |
| PDE3A |
| ZFPL1 |
| NUP58 |
| KLHDC8B |
| ALOX12-AS1 |
| SMIM3 |
| ABCG1 |
| SRD5A3 |
| ACSL4 |
| MFSD14B |
| SBDSP1 |
| CINP |
| UBE2D4 |
| FLYWCH2 |
| FAM120A |
| SCAF4 |
| MED8 |
| ACSL3 |
| TMEM150A |
| ZNF688 |
| MYCBP |
| PLEKHJ1 |
| OTUD4 |
| MRPS18A |
| MMP3 |
| ZNF800 |
| TGIF2 |
| MEF2D |
| SIAH1 |
| MED11 |
| ZBTB7A |
| HIF1A |
| BCL6 |
| HIF1A-AS3 |
| PI4KB |
| NFKBIL1 |
| MED30 |
| RBBP6 |
| NAA50 |
| THRAP3 |
| GEMIN7 |
| SHOC2 |
| FTSJ1 |
| DHX9 |
| SLC36A4 |
| STK17B |
| PDPK1 |
| SRP54 |
| ADIPOR2 |
| ZDHHC20 |
| FBXO6 |
| IQCG |
| NCKAP5L |
| ZRANB1 |
| ZBTB11 |
| BAK1 |
| CHASERR |
| DLGAP4 |
| MTUS1 |
| NDEL1 |
| ATP6V1C1 |
| G3BP1 |
| IPO7 |
| SLC25A25 |
| TXNDC11 |
| GNAI3 |
| CLEC11A |
| PDRG1 |
| PER2 |
| ABHD17B |
| SESN3 |
| PPP2CB |
| ARL13B |
| CARS2 |
| LSS |
| IST1 |
| C1orf174 |
| GCLC |
| OPA3 |
| SAYSD1 |
| USP7 |
| SLC8B1 |
| CXCL12 |
| ZNFX1 |
| TMEM42 |
| ZBTB38 |
| UBAP2L |
| CAMKK2 |
| RDX |
| CGAS |
| ERRFI1 |
| VDR |
| YES1 |
| SOAT1 |
| KDM3A |
| STX3 |
| RCAN1 |
| LINC01150 |
| LPIN1 |
| CNNM4 |
| KDM1A |
| BEX3 |
| DUSP10 |
| VCAN |
| TIMM22 |
| KBTBD2 |
| DNAJC30 |
| NOD2 |
| ZNF10 |
| GRWD1 |
| SMAD7 |
| PLEKHM1 |
| RNF138 |
| NEMP1 |
| ZCCHC10 |
| PATL1 |
| TNFRSF10D |
| TFRC |
| TXLNG |
| NCKAP1 |
| HAVCR1 |
| ENSG00000234261 |
| C6orf136 |
| ENSG00000275964 |
| ZNF143 |
| WDR47 |
| IGFBP6 |
| UBE2G1 |
| TRHDE |
| MYOM1 |
| RNMT |
| TFCP2L1 |
| PTER |
| PHTF1 |
| PLAC9 |
| RASSF7 |
| SLC66A2 |
| UBE2Z |
| SLC17A5 |
| ZBTB4 |
| JMY |
| NFYA |
| SNX8 |
| CCDC138 |
| CDKL3 |
| MRNIP |
| TLR1 |
| UAP1 |
| PPRC1 |
| CSNK1E |
| PURA |
| C3orf62 |
| WASL |
| GTF2A1 |
| DIPK2A |
| DPM1 |
| BIRC2 |
| GTPBP1 |
| STX5 |
| MDC1 |
| ZSWIM3 |
| KRT10-AS1 |
| NOC3L |
| RRN3 |
| DERPC |
| ZNF410 |
| ZNF335 |
| APPAT |
| IFFO2 |
| GAPT |
| PCID2 |
| UPK3BL1 |
| BOLA2B |
| RETREG1 |
| ENSG00000267469 |
| HTD2 |
| NOP58 |
| ENSG00000289100 |
| MIR142HG |
| FBXL14 |
| ENSG00000285280 |
| NUP188 |
| ENSG00000265496 |
| FMR1 |
| SMG1P5 |
| IPCEF1 |
| SUCLG2-DT |
| LPCAT1 |
| SMARCA5-AS1 |
| ELF4 |
| MOB2 |
| GRAMD1A |
| RBM10 |
| SNX16 |
| ENSG00000265401 |
| H2AC17 |
| TRGV3 |
| FAM241B |
| ENSG00000272742 |
| ENSG00000285976 |
| ZNF844 |
| YARS1 |
| TFPI |
| SPINDOC |
| IGKV1-6 |
| CROCC |
| PDE8B |
| CENPA |
| BRPF3 |
| TMEM97 |
| ENSG00000230113 |
| JADE1 |
| IKZF4 |
| ZCWPW1 |
| NUCB1-AS1 |
| ZBTB18 |
| HAS1 |
| H2AC21 |
| PDE1B |
| FBXO30 |
| ENSG00000288936 |
| FGFBP3 |
| DYRK2 |
| ITGB7 |
| XG |
| RPUSD2 |
| ENSG00000288839 |
| ENSG00000260879 |
| MYL9 |
| ENSG00000289261 |
| FRMD8 |
| DMPK |
| DAG1 |
| MASTL |
| ENSG00000290859 |
| PLXNA3 |
| ENSG00000265737 |
| TRGV5 |
| ENSG00000228793 |
| VWCE |
| LINC02610 |
| ELK4 |
| ENSG00000254682 |
| CD79B |
| CCDC89 |
| PHF10 |
| LINC01191 |
| FAM9B |
| ATF1 |
| FAM117A |
| TRGV7 |
| BTG1-DT |
| PIK3R5-DT |
| WRNIP1 |
| KCNJ14 |
| LINC01914 |
| PPM1K |
| ING3 |
| LRP2BP |
| GBP3 |
| PAQR5 |
| ENSG00000235820 |
| IGHV1-24 |
| C7orf31 |
| PDGFRL |
| CD34 |
| XPC-AS1 |
| ENSG00000273275 |
| ENSG00000235288 |
| EFNB2 |
| C17orf67 |
| SLC35E4 |
| LINC00900 |
| MEIKIN |
| IGLV2-8 |
| ENSG00000289588 |
| UBE2T |
| ADGRF3 |
| HAUS6 |
| ENSG00000259238 |
| VNN1 |
| IGLV3-1 |
| LINC02977 |
| PITPNA-AS1 |
| SNHG3 |
| ENSG00000291111 |
| ENSG00000269072 |
| CD1C |
| LINC01116 |
| ENSG00000255491 |
| TMCC3 |
| FOXO6 |
| CXCL14 |
| SLX1A |
| ENSG00000285999 |
| CEBPA-DT |
| GPR153 |
| SLC25A34 |
| SNHG31 |
| TFAP2E-AS1 |
| PSRC1 |
| SRC |
| CENPM |
| LEAP2 |
| AACS |
| LIPT2 |
| KCNE1 |
| LINC02947 |
| SCGB3A2 |
| ENSG00000230454 |
| KIZ-AS1 |
| PCDH12 |
| ENSG00000290792 |
| IQCE |
| ENSG00000290549 |
| LINC00511 |
| CRYAB |
| ENSG00000289189 |
| LNCTSI |
| ACTA2 |
| ECRG4 |
| RAB7B |
| SYNPO |
| ZNF416 |
| LINGO3 |
| LINC02035 |
| CBR1-AS1 |
| OSR2 |
| SHC3 |
| ENSG00000274015 |
| QPCTL |
| MFAP4 |
| ENSG00000267011 |
| ENSG00000258744 |
| ZBTB2 |
| TMEM38A |
| ENSG00000243389 |
| LINC01506 |
| PLEKHA4 |
| SLCO4A1 |
| E2F6 |
| FZD8 |
| VAMP1 |
| SPAG6 |
| FCGBP |
| SSPN |
| C16orf46 |
| TBC1D3L |
| ENSG00000272092 |
| KL |
| LINC02577 |
| TMPO-AS1 |
| ENSG00000287032 |
| SYNDIG1L |
| SLAMF1 |
| ENSG00000273080 |
| SYCE1L |
| ENSG00000286677 |
| CPEB2-DT |
| ENSG00000233005 |
| DLG4 |
| ENSG00000236255 |
| PXYLP1 |
| SEPTIN4-AS1 |
| LINC00654 |
| SLC20A1-DT |
| LRRC75A |
| GALNT4 |
| ERI2 |
| DONSON |
| CALCRL-AS1 |
| ENSG00000249631 |
| LAT |
| CNFN |
| ENSG00000289952 |
| ENSG00000261845 |
| ADIRF-AS1 |
| TRAF3IP2 |
| MDGA1 |
| LINC02361 |
| HSPA4L |
| ENSG00000289633 |
| CDO1 |
| MYBL1 |
| CATIP-AS1 |
| ZNF821 |
| RASSF1-AS1 |
| USP2-AS1 |
| NETO2 |
| PRC1-AS1 |
| ENSG00000289987 |
| MAP3K14-AS1 |
| NUP153-AS1 |
| SLC5A11 |
| DMWD |
| PCLAF |
| UCKL1-AS1 |
| ENSG00000261799 |
| LINC00528 |
| IRF4 |
| YOD1 |
| CEBPE |
| ENSG00000289226 |
| LMCD1 |
| CFAP53 |
| C1orf56 |
| ENSG00000287494 |
| STX5-DT |
| ENSG00000288785 |
| LINC02613 |
| PLAGL2 |
| LINC02362 |
| ENSG00000258525 |
| STAT4 |
| IL1R1 |
| PHC1 |
| CDC42-AS1 |
| SOD3 |
| GPR146 |
| DNAJC18 |
| HS3ST3B1 |
| SGMS1-AS1 |
| PPIL6 |
| PLGLB1 |
| THY1 |
| TFAP2C |
| ADCY10 |
| LPL |
| INHBA |
| SOX13 |
| ARL9 |
| NECTIN1 |
| ENSG00000272908 |
| ENSG00000274104 |
| TPBG |
| ST3GAL1-DT |
| ENSG00000272669 |
| CNN3 |
| LINC01474 |
| ENSG00000257060 |
| LINC01270 |
| CFAP141 |
| PLCG1-AS1 |
| TBC1D25 |
| DAB2IP |
| ENSG00000289410 |
| ENSG00000273216 |
| FHL1 |
| ENSG00000243797 |
| DERL3 |
| HIC2 |
| IL32 |
| CCND2 |
| PDYN-AS1 |
| SNTA1 |
| FOXRED2 |
| ID1 |
| LINC02631 |
| TMEM64 |
| GNB5 |
| TYMS |
| LRRC4 |
| CDC42EP5 |
| MFGE8 |
| ALDH1A3 |
| POPDC2 |
| CLDN4 |
| PYGO1 |
| MMP17 |
| PDZD7 |
| AGFG2 |
| IFNLR1 |
| EFEMP2 |
| PPAT |
| STK26 |
| SLC40A1 |
| CLEC12A |
| LINC01068 |
| ENSG00000289583 |
| LINC00278 |
| UTY |
| ENSG00000282885 |
| NR1I2 |
| GIMAP7 |
| H2BC11 |
| ZNF331 |
| ZNF256 |
| ENSG00000288957 |
| VAX1 |
| ENSG00000229983 |
| ZNF285 |
| RAB20 |
| SCO2 |
| FAM106A |
| ENSG00000214708 |
| SIGLECL1 |
| ENSG00000283445 |

## Supplementary Table S5. Genes derived from L-R pairs from cell communication

| source | target | ligand | receptor | prob | pval | interaction_name | interaction_name_2 | pathway_name | annotation | evidence |
| --- | --- | --- | --- | --- | --- | --- | --- | --- | --- | --- |
| M2 macrophages | M2 macrophages | TGFB1 | TGFbR1_R2 | 0.00588349757811723 | 0.01 | TGFB1_TGFBR1_TGFBR2 | TGFB1 - (TGFBR1+TGFBR2) | TGFb | Secreted Signaling | KEGG: hsa04350 |
| M1 macrophages | M2 macrophages | TGFB2 | TGFbR1_R2 | 0.00121408849343539 | 0 | TGFB2_TGFBR1_TGFBR2 | TGFB2 - (TGFBR1+TGFBR2) | TGFb | Secreted Signaling | KEGG: hsa04350 |
| M1 macrophages | M1 macrophages | TGFB1 | ACVR1B_TGFbR2 | 0.00137269506552793 | 0.03 | TGFB1_ACVR1B_TGFBR2 | TGFB1 - (ACVR1B+TGFBR2) | TGFb | Secreted Signaling | PMID: 27449815 |
| M2 macrophages | M1 macrophages | TGFB1 | ACVR1B_TGFbR2 | 0.00142494764077553 | 0 | TGFB1_ACVR1B_TGFBR2 | TGFB1 - (ACVR1B+TGFBR2) | TGFb | Secreted Signaling | PMID: 27449815 |
| M1 macrophages | M1 macrophages | TGFB2 | ACVR1B_TGFbR2 | 0.000293005573651664 | 0 | TGFB2_ACVR1B_TGFBR2 | TGFB2 - (ACVR1B+TGFBR2) | TGFb | Secreted Signaling | PMID: 27449815 |
| M2 macrophages | M1 macrophages | TGFB2 | ACVR1B_TGFbR2 | 0.000274074160051802 | 0 | TGFB2_ACVR1B_TGFBR2 | TGFB2 - (ACVR1B+TGFBR2) | TGFb | Secreted Signaling | PMID: 27449815 |
| M2 macrophages | M1 macrophages | TGFB1 | ACVR1_TGFbR | 0.00153575993818477 | 0 | TGFB1_ACVR1_TGFBR1 | TGFB1 - (ACVR1+TGFBR1) | TGFb | Secreted Signaling | PMID: 29376829 |
| M1 macrophages | M1 macrophages | TGFB2 | ACVR1_TGFbR | 0.000318830616957828 | 0 | TGFB2_ACVR1_TGFBR1 | TGFB2 - (ACVR1+TGFBR1) | TGFb | Secreted Signaling | PMID: 29376829 |
| M1 macrophages | M2 macrophages | TGFB2 | ACVR1_TGFbR | 0.00030786288941518 | 0.02 | TGFB2_ACVR1_TGFBR1 | TGFB2 - (ACVR1+TGFBR1) | TGFb | Secreted Signaling | PMID: 29376829 |
| M1 macrophages | M1 macrophages | BMP2 | BMPR1B_ACVR2A | 4.16635809960829e-05 | 0 | BMP2_BMPR1B_ACVR2A | BMP2 - (BMPR1B+ACVR2A) | BMP | Secreted Signaling | KEGG: hsa04350; PMID:26893264 |
| M2 macrophages | M1 macrophages | BMP2 | BMPR1B_ACVR2A | 3.747457780762e-05 | 0 | BMP2_BMPR1B_ACVR2A | BMP2 - (BMPR1B+ACVR2A) | BMP | Secreted Signaling | KEGG: hsa04350; PMID:26893264 |
| M1 macrophages | M1 macrophages | BMP2 | BMPR1B_ACVR2B | 1.65416323484121e-05 | 0 | BMP2_BMPR1B_ACVR2B | BMP2 - (BMPR1B+ACVR2B) | BMP | Secreted Signaling | KEGG: hsa04350; PMID:26893264 |
| M1 macrophages | M1 macrophages | BMP2 | BMPR1B_BMPR2 | 4.66975303963875e-05 | 0 | BMP2_BMPR1B_BMPR2 | BMP2 - (BMPR1B+BMPR2) | BMP | Secreted Signaling | KEGG: hsa04350; PMID:26893264 |
| M2 macrophages | M1 macrophages | BMP2 | BMPR1B_BMPR2 | 4.20024174633284e-05 | 0.02 | BMP2_BMPR1B_BMPR2 | BMP2 - (BMPR1B+BMPR2) | BMP | Secreted Signaling | KEGG: hsa04350; PMID:26893264 |
| M1 macrophages | M1 macrophages | INHBA | ACVR1B_ACVR2A | 2.68362673162782e-05 | 0 | INHBA_ACVR1B_ACVR2A | INHBA - (ACVR1B+ACVR2A) | ACTIVIN | Secreted Signaling | KEGG: hsa04350 |
| M2 macrophages | M1 macrophages | INHBA | ACVR1B_ACVR2A | 6.09838501017252e-06 | 0 | INHBA_ACVR1B_ACVR2A | INHBA - (ACVR1B+ACVR2A) | ACTIVIN | Secreted Signaling | KEGG: hsa04350 |
| M1 macrophages | M1 macrophages | INHBA | ACVR1B_ACVR2B | 1.06546706514592e-05 | 0 | INHBA_ACVR1B_ACVR2B | INHBA - (ACVR1B+ACVR2B) | ACTIVIN | Secreted Signaling | KEGG: hsa04351 |
| M1 macrophages | M2 macrophages | INHBA | ACVR1B_ACVR2B | 8.50829834669834e-06 | 0 | INHBA_ACVR1B_ACVR2B | INHBA - (ACVR1B+ACVR2B) | ACTIVIN | Secreted Signaling | KEGG: hsa04351 |
| M1 macrophages | M1 macrophages | WNT2B | FZD8_LRP5 | 1.83457334363448e-06 | 0 | WNT2B_FZD8_LRP5 | WNT2B - (FZD8+LRP5) | WNT | Secreted Signaling | KEGG: hsa04310; PMID: 23209147 |
| M1 macrophages | M2 macrophages | WNT2B | FZD8_LRP5 | 1.59799705214343e-06 | 0 | WNT2B_FZD8_LRP5 | WNT2B - (FZD8+LRP5) | WNT | Secreted Signaling | KEGG: hsa04310; PMID: 23209147 |
| M1 macrophages | M1 macrophages | WNT2B | FZD8_LRP6 | 2.56285965006843e-06 | 0 | WNT2B_FZD8_LRP6 | WNT2B - (FZD8+LRP6) | WNT | Secreted Signaling | KEGG: hsa04310; PMID: 23209147 |
| M1 macrophages | M2 macrophages | WNT2B | FZD8_LRP6 | 2.13261890185249e-06 | 0 | WNT2B_FZD8_LRP6 | WNT2B - (FZD8+LRP6) | WNT | Secreted Signaling | KEGG: hsa04310; PMID: 23209147 |
| M1 macrophages | M1 macrophages | TGFA | EGFR | 5.31426965840143e-06 | 0 | TGFA_EGFR | TGFA - EGFR | EGF | Secreted Signaling | KEGG: hsa04012 |
| M1 macrophages | M2 macrophages | TGFA | EGFR | 4.93119278468443e-06 | 0 | TGFA_EGFR | TGFA - EGFR | EGF | Secreted Signaling | KEGG: hsa04012 |
| M1 macrophages | M1 macrophages | TGFA | EGFR_ERBB2 | 5.50394184145273e-06 | 0 | TGFA_EGFR_ERBB2 | TGFA - (EGFR+ERBB2) | EGF | Secreted Signaling | KEGG: hsa04012 |
| M1 macrophages | M2 macrophages | TGFA | EGFR_ERBB2 | 5.30155037224093e-06 | 0 | TGFA_EGFR_ERBB2 | TGFA - (EGFR+ERBB2) | EGF | Secreted Signaling | KEGG: hsa04012 |
| M2 macrophages | M2 macrophages | PDGFA | PDGFRA | 2.87876478868914e-05 | 0.02 | PDGFA_PDGFRA | PDGFA - PDGFRA | PDGF | Secreted Signaling | PMID: 15207812 |
| M1 macrophages | M1 macrophages | PDGFB | PDGFRA | 0.000169755228718354 | 0 | PDGFB_PDGFRA | PDGFB - PDGFRA | PDGF | Secreted Signaling | PMID: 15207812 |
| M1 macrophages | M2 macrophages | PDGFB | PDGFRA | 0.000181442007824761 | 0 | PDGFB_PDGFRA | PDGFB - PDGFRA | PDGF | Secreted Signaling | PMID: 15207812 |
| M2 macrophages | M1 macrophages | PDGFC | PDGFRA | 0.000598697380785255 | 0 | PDGFC_PDGFRA | PDGFC - PDGFRA | PDGF | Secreted Signaling | PMID: 15207812 |
| M2 macrophages | M2 macrophages | PDGFC | PDGFRA | 0.000639895733155809 | 0 | PDGFC_PDGFRA | PDGFC - PDGFRA | PDGF | Secreted Signaling | PMID: 15207812 |
| M2 macrophages | M1 macrophages | VEGFA | FLT1 | 5.03232990289846e-05 | 0 | VEGFA_VEGFR1 | VEGFA - VEGFR1 | VEGF | Secreted Signaling | KEGG: hsa04370; PMID: 16633338 |
| M2 macrophages | M1 macrophages | VEGFA | KDR | 3.1938615773647e-05 | 0.02 | VEGFA_VEGFR2 | VEGFA - VEGFR2 | VEGF | Secreted Signaling | KEGG: hsa04370; PMID: 16633338 |
| M1 macrophages | M2 macrophages | VEGFA | KDR | 3.93385301020851e-05 | 0 | VEGFA_VEGFR2 | VEGFA - VEGFR2 | VEGF | Secreted Signaling | KEGG: hsa04370; PMID: 16633338 |
| M2 macrophages | M2 macrophages | VEGFA | KDR | 5.74011664748076e-05 | 0 | VEGFA_VEGFR2 | VEGFA - VEGFR2 | VEGF | Secreted Signaling | KEGG: hsa04370; PMID: 16633338 |
| M1 macrophages | M1 macrophages | VEGFB | FLT1 | 3.0524285275792e-05 | 0 | VEGFB_VEGFR1 | VEGFB - VEGFR1 | VEGF | Secreted Signaling | KEGG: hsa04370; PMID: 16633338 |
| M2 macrophages | M1 macrophages | VEGFC | KDR | 7.306443166742e-06 | 0 | VEGFC_VEGFR2 | VEGFC - VEGFR2 | VEGF | Secreted Signaling | KEGG: hsa04370; PMID: 16633338 |
| M1 macrophages | M2 macrophages | VEGFC | KDR | 8.42746091037927e-06 | 0 | VEGFC_VEGFR2 | VEGFC - VEGFR2 | VEGF | Secreted Signaling | KEGG: hsa04370; PMID: 16633338 |
| M2 macrophages | M2 macrophages | VEGFC | KDR | 1.31316460277964e-05 | 0 | VEGFC_VEGFR2 | VEGFC - VEGFR2 | VEGF | Secreted Signaling | KEGG: hsa04370; PMID: 16633338 |
| M2 macrophages | M1 macrophages | VEGFA | FLT1_KDR | 4.20896383889982e-05 | 0 | VEGFA_VEGFR1R2 | VEGFA - VEGFR1R2 | VEGF | Secreted Signaling | PMID: 16633338 |
| M2 macrophages | M2 macrophages | VEGFA | FLT1_KDR | 4.63174306948562e-05 | 0 | VEGFA_VEGFR1R2 | VEGFA - VEGFR1R2 | VEGF | Secreted Signaling | PMID: 16633338 |
| M2 macrophages | M1 macrophages | VEGFC | FLT4_KDR | 5.68706280193272e-06 | 0 | VEGFC_VEGFR2R3 | VEGFC - VEGFR2R3 | VEGF | Secreted Signaling | PMID: 16633338 |
| M1 macrophages | M2 macrophages | VEGFC | FLT4_KDR | 5.54733812817064e-06 | 0 | VEGFC_VEGFR2R3 | VEGFC - VEGFR2R3 | VEGF | Secreted Signaling | PMID: 16633338 |
| M2 macrophages | M2 macrophages | VEGFC | FLT4_KDR | 8.64386065788588e-06 | 0 | VEGFC_VEGFR2R3 | VEGFC - VEGFR2R3 | VEGF | Secreted Signaling | PMID: 16633338 |
| M2 macrophages | M1 macrophages | IGF1 | IGF1R | 2.59622408905219e-05 | 0 | IGF1_IGF1R | IGF1 - IGF1R | IGF | Secreted Signaling | PMID: 14604834 |
| M2 macrophages | M2 macrophages | IGF1 | IGF1R | 5.79747410640883e-05 | 0 | IGF1_IGF1R | IGF1 - IGF1R | IGF | Secreted Signaling | PMID: 14604834 |
| M1 macrophages | M1 macrophages | CCL3 | CCR1 | 0.00760534691103706 | 0 | CCL3_CCR1 | CCL3 - CCR1 | CCL | Secreted Signaling | KEGG: hsa04060 |
| M1 macrophages | M2 macrophages | CCL3 | CCR1 | 0.00434615046392967 | 0 | CCL3_CCR1 | CCL3 - CCR1 | CCL | Secreted Signaling | KEGG: hsa04060 |
| M1 macrophages | M1 macrophages | CCL3L3 | CCR1 | 0.00699929657345124 | 0 | CCL3L3_CCR1 | CCL3L3 - CCR1 | CCL | Secreted Signaling | KEGG: hsa04060 |
| M1 macrophages | M2 macrophages | CCL3L3 | CCR1 | 0.00399877051689759 | 0 | CCL3L3_CCR1 | CCL3L3 - CCR1 | CCL | Secreted Signaling | KEGG: hsa04060 |
| M2 macrophages | M2 macrophages | CCL2 | CCR2 | 0.000949226087307123 | 0 | CCL2_CCR2 | CCL2 - CCR2 | CCL | Secreted Signaling | KEGG: hsa04060 |
| M1 macrophages | M1 macrophages | CCL4 | CCR5 | 0.0158655204791495 | 0 | CCL4_CCR5 | CCL4 - CCR5 | CCL | Secreted Signaling | KEGG: hsa04060 |
| M1 macrophages | M1 macrophages | CCL3 | CCR5 | 0.0167255401602048 | 0 | CCL3_CCR5 | CCL3 - CCR5 | CCL | Secreted Signaling | KEGG: hsa04060 |
| M1 macrophages | M1 macrophages | CXCL12 | CXCR4 | 0.000857702301845656 | 0 | CXCL12_CXCR4 | CXCL12 - CXCR4 | CXCL | Secreted Signaling | KEGG: hsa04060 |
| M2 macrophages | M1 macrophages | CXCL12 | CXCR4 | 0.000496825888911524 | 0 | CXCL12_CXCR4 | CXCL12 - CXCR4 | CXCL | Secreted Signaling | KEGG: hsa04060 |
| M1 macrophages | M1 macrophages | CXCL12 | ACKR3 | 0.00102688374816486 | 0 | CXCL12_ACKR3 | CXCL12 - ACKR3 | CXCL | Secreted Signaling | KEGG: hsa04060 |
| M2 macrophages | M1 macrophages | CXCL12 | ACKR3 | 0.00059486698337791 | 0 | CXCL12_ACKR3 | CXCL12 - ACKR3 | CXCL | Secreted Signaling | KEGG: hsa04060 |
| M1 macrophages | M1 macrophages | MIF | CD74_CXCR4 | 0.0127193237665039 | 0 | MIF_CD74_CXCR4 | MIF - (CD74+CXCR4) | MIF | Secreted Signaling | PMID: 29637711; PMID: 24760155 |
| M2 macrophages | M1 macrophages | MIF | CD74_CXCR4 | 0.0122719741553288 | 0 | MIF_CD74_CXCR4 | MIF - (CD74+CXCR4) | MIF | Secreted Signaling | PMID: 29637711; PMID: 24760155 |
| M1 macrophages | M1 macrophages | MIF | CD74_CD44 | 0.0327392865385092 | 0 | MIF_CD74_CD44 | MIF - (CD74+CD44) | MIF | Secreted Signaling | PMID: 29637711; PMID: 26175090 |
| M2 macrophages | M1 macrophages | MIF | CD74_CD44 | 0.0316103617478098 | 0 | MIF_CD74_CD44 | MIF - (CD74+CD44) | MIF | Secreted Signaling | PMID: 29637711; PMID: 26175090 |
| M1 macrophages | M2 macrophages | MIF | CD74_CXCR2 | 0.00034774602628238 | 0 | MIF_CD74_CXCR2 | MIF - (CD74+CXCR2) | MIF | Secreted Signaling | PMID: 29637711; PMID: 26175090 |
| M1 macrophages | M1 macrophages | MIF | ACKR3 | 0.00439821062124251 | 0 | MIF_ACKR3 | MIF - ACKR3 | MIF | Secreted Signaling | PMID: 26175090 |
| M2 macrophages | M1 macrophages | MIF | ACKR3 | 0.00424226421273507 | 0 | MIF_ACKR3 | MIF - ACKR3 | MIF | Secreted Signaling | PMID: 26175090 |
| M1 macrophages | M1 macrophages | IL7 | IL7R_IL2RG | 5.63952157917767e-06 | 0 | IL7_IL7R_IL2RG | IL7 - (IL7R+IL2RG) | IL2 | Secreted Signaling | KEGG: hsa04060 |
| M2 macrophages | M1 macrophages | IL7 | IL7R_IL2RG | 4.78494509996597e-06 | 0 | IL7_IL7R_IL2RG | IL7 - (IL7R+IL2RG) | IL2 | Secreted Signaling | KEGG: hsa04060 |
| M1 macrophages | M2 macrophages | IL7 | IL7R_IL2RG | 4.46335336272419e-06 | 0 | IL7_IL7R_IL2RG | IL7 - (IL7R+IL2RG) | IL2 | Secreted Signaling | KEGG: hsa04060 |
| M1 macrophages | M1 macrophages | IL15 | IL15RA_IL2RB | 2.88626434573211e-05 | 0 | IL15_IL15RA_IL2RB | IL15 - (IL15RA+IL2RB) | IL2 | Secreted Signaling | KEGG: hsa04060 |
| M2 macrophages | M1 macrophages | IL15 | IL15RA_IL2RB | 8.13569352758995e-06 | 0 | IL15_IL15RA_IL2RB | IL15 - (IL15RA+IL2RB) | IL2 | Secreted Signaling | KEGG: hsa04060 |
| M1 macrophages | M2 macrophages | IL15 | IL15RA_IL2RB | 1.48904417898201e-05 | 0 | IL15_IL15RA_IL2RB | IL15 - (IL15RA+IL2RB) | IL2 | Secreted Signaling | KEGG: hsa04060 |
| M1 macrophages | M1 macrophages | IL6 | IL6R_IL6ST | 0.00209220028889628 | 0 | IL6_IL6R_IL6ST | IL6 - (IL6R+IL6ST) | IL6 | Secreted Signaling | KEGG: hsa04060 |
| M1 macrophages | M1 macrophages | CNTF | CNTFR_LIFR | 2.29233304043693e-06 | 0 | CNTF_CNTFR_LIFR | CNTF - (CNTFR+LIFR) | LIFR | Secreted Signaling | KEGG: hsa04060 |
| M1 macrophages | M1 macrophages | LIF | LIFR_IL6ST | 9.28091729640615e-05 | 0 | LIF_LIFR_IL6ST | LIF - (LIFR+IL6ST) | LIFR | Secreted Signaling | KEGG: hsa04060 |
| M1 macrophages | M1 macrophages | OSM | LIFR_IL6ST | 8.17643890005929e-06 | 0 | OSM_LIFR_IL6ST | OSM - (LIFR+IL6ST) | OSM | Secreted Signaling | KEGG: hsa04060 |
| M1 macrophages | M1 macrophages | IL1A | IL1R1_IL1RAP | 2.94233779201509e-05 | 0 | IL1A_IL1R1_IL1RAP | IL1A - (IL1R1+IL1RAP) | IL1 | Secreted Signaling | KEGG: hsa04060 |
| M2 macrophages | M1 macrophages | IL1A | IL1R1_IL1RAP | 6.00925622398969e-06 | 0 | IL1A_IL1R1_IL1RAP | IL1A - (IL1R1+IL1RAP) | IL1 | Secreted Signaling | KEGG: hsa04060 |
| M1 macrophages | M1 macrophages | IL1B | IL1R1_IL1RAP | 0.00533110483011076 | 0 | IL1B_IL1R1_IL1RAP | IL1B - (IL1R1+IL1RAP) | IL1 | Secreted Signaling | KEGG: hsa04060 |
| M1 macrophages | M1 macrophages | IL1A | IL1R2 | 5.22452775730837e-05 | 0 | IL1A_IL1R2 | IL1A - IL1R2 | IL1 | Secreted Signaling | KEGG: hsa04060 |
| M2 macrophages | M1 macrophages | IL1A | IL1R2 | 1.05024479744207e-05 | 0 | IL1A_IL1R2 | IL1A - IL1R2 | IL1 | Secreted Signaling | KEGG: hsa04060 |
| M1 macrophages | M1 macrophages | IL1B | IL1R2 | 0.00942902629380977 | 0 | IL1B_IL1R2 | IL1B - IL1R2 | IL1 | Secreted Signaling | KEGG: hsa04060 |
| M2 macrophages | M2 macrophages | CSF1 | CSF1R | 0.00549005439270678 | 0 | CSF1_CSF1R | CSF1 - CSF1R | CSF | Secreted Signaling | KEGG: hsa04060 |
| M1 macrophages | M1 macrophages | CSF3 | CSF3R | 0.000120190290805209 | 0 | CSF3_CSF3R | CSF3 - CSF3R | CSF3 | Secreted Signaling | KEGG: hsa04060 |
| M2 macrophages | M1 macrophages | CSF3 | CSF3R | 1.88713150678692e-06 | 0 | CSF3_CSF3R | CSF3 - CSF3R | CSF3 | Secreted Signaling | KEGG: hsa04060 |
| M1 macrophages | M2 macrophages | CSF3 | CSF3R | 1.89428411699305e-06 | 0 | CSF3_CSF3R | CSF3 - CSF3R | CSF3 | Secreted Signaling | KEGG: hsa04060 |
| M1 macrophages | M1 macrophages | TNF | TNFRSF1A | 0.00492027379621344 | 0 | TNF_TNFRSF1A | TNF - TNFRSF1A | TNF | Secreted Signaling | KEGG: hsa04060 |
| M1 macrophages | M2 macrophages | TNF | TNFRSF1A | 0.0116894775798588 | 0 | TNF_TNFRSF1A | TNF - TNFRSF1A | TNF | Secreted Signaling | KEGG: hsa04060 |
| M1 macrophages | M1 macrophages | TNF | TNFRSF1B | 0.00441341448007211 | 0 | TNF_TNFRSF1B | TNF - TNFRSF1B | TNF | Secreted Signaling | KEGG: hsa04060 |
| M1 macrophages | M2 macrophages | TNF | TNFRSF1B | 0.00513275920906194 | 0 | TNF_TNFRSF1B | TNF - TNFRSF1B | TNF | Secreted Signaling | KEGG: hsa04060 |
| M2 macrophages | M1 macrophages | LTA | TNFRSF1A | 0.000439690418272287 | 0 | LTA_TNFRSF1A | LTA - TNFRSF1A | LT | Secreted Signaling | KEGG: hsa04060 |
| M1 macrophages | M2 macrophages | LTA | TNFRSF1A | 0.000437836508655373 | 0 | LTA_TNFRSF1A | LTA - TNFRSF1A | LT | Secreted Signaling | KEGG: hsa04060 |
| M2 macrophages | M2 macrophages | LTA | TNFRSF1A | 0.00105111819636325 | 0 | LTA_TNFRSF1A | LTA - TNFRSF1A | LT | Secreted Signaling | KEGG: hsa04060 |
| M2 macrophages | M1 macrophages | LTA | TNFRSF1B | 0.000394213095526665 | 0 | LTA_TNFRSF1B | LTA - TNFRSF1B | LT | Secreted Signaling | KEGG: hsa04060 |
| M2 macrophages | M2 macrophages | LTA | TNFRSF1B | 0.000458767961027104 | 0 | LTA_TNFRSF1B | LTA - TNFRSF1B | LT | Secreted Signaling | KEGG: hsa04060 |
| M2 macrophages | M2 macrophages | LTA | TNFRSF14 | 0.000317160417145747 | 0 | LTA_TNFRSF14 | LTA - TNFRSF14 | LT | Secreted Signaling | KEGG: hsa04060 |
| M2 macrophages | M2 macrophages | LTa1b2 | LTBR | 0.000260543925281963 | 0 | LTA_LTB_LTBR | LTA - (LTB+LTBR) | LT | Secreted Signaling | PMID: 24248355 |
| M2 macrophages | M2 macrophages | TNFSF14 | LTBR | 9.70190508709008e-05 | 0 | TNFSF14_LTBR | TNFSF14 - LTBR | LIGHT | Secreted Signaling | KEGG: hsa04060 |
| M2 macrophages | M2 macrophages | TNFSF14 | TNFRSF14 | 8.40223683937009e-05 | 0 | TNFSF14_TNFRSF14 | TNFSF14 - TNFRSF14 | LIGHT | Secreted Signaling | KEGG: hsa04060 |
| M2 macrophages | M2 macrophages | TNFSF10 | TNFRSF10A | 8.89394744355764e-07 | 0 | TNFSF10_TNFRSF10B | TNFSF10 - TNFRSF10B | TRAIL | Secreted Signaling | KEGG: hsa04060 |
| M1 macrophages | M1 macrophages | NAMPT | INSR | 0.000581931629422833 | 0 | NAMPT_INSR | NAMPT - INSR | VISFATIN | Secreted Signaling | PMID: 28490838 |
| M1 macrophages | M2 macrophages | NAMPT | INSR | 0.000572243410857047 | 0 | NAMPT_INSR | NAMPT - INSR | VISFATIN | Secreted Signaling | PMID: 28490838 |
| M1 macrophages | M1 macrophages | NAMPT | ITGA5_ITGB1 | 0.0350522132642187 | 0 | NAMPT_ITGA5_ITGB1 | NAMPT - (ITGA5+ITGB1) | VISFATIN | Secreted Signaling | PMID: 28490838 |
| M1 macrophages | M2 macrophages | NAMPT | ITGA5_ITGB1 | 0.0223919290108967 | 0 | NAMPT_ITGA5_ITGB1 | NAMPT - (ITGA5+ITGB1) | VISFATIN | Secreted Signaling | PMID: 28490838 |
| M1 macrophages | M1 macrophages | C3 | C3AR1 | 0.000467102820932766 | 0 | C3_C3AR1 | C3 - C3AR1 | COMPLEMENT | Secreted Signaling | KEGG: hsa04080 |
| M1 macrophages | M2 macrophages | C3 | C3AR1 | 0.000510413733049623 | 0 | C3_C3AR1 | C3 - C3AR1 | COMPLEMENT | Secreted Signaling | KEGG: hsa04080 |
| M1 macrophages | M1 macrophages | C3 | ITGAM_ITGB2 | 0.000903306960510122 | 0 | C3_ITGAM_ITGB2 | C3 - (ITGAM+ITGB2) | COMPLEMENT | Secreted Signaling | PMID: 16234578 |
| M1 macrophages | M1 macrophages | C3 | ITGAX_ITGB2 | 0.000769457830980088 | 0 | C3_ITGAX_ITGB2 | C3 - (ITGAX+ITGB2) | COMPLEMENT | Secreted Signaling | PMID: 16234578 |
| M2 macrophages | M1 macrophages | C3 | ITGAX_ITGB2 | 0.000584886234619932 | 0 | C3_ITGAX_ITGB2 | C3 - (ITGAX+ITGB2) | COMPLEMENT | Secreted Signaling | PMID: 16234578 |
| M1 macrophages | M1 macrophages | C5 | C5AR1 | 7.6091284419801e-07 | 0 | HC_C5AR1 | HC - C5AR1 | COMPLEMENT | Secreted Signaling | KEGG: hsa04080 |
| M2 macrophages | M1 macrophages | C5 | C5AR1 | 6.8256404768472e-07 | 0 | HC_C5AR1 | HC - C5AR1 | COMPLEMENT | Secreted Signaling | KEGG: hsa04080 |
| M1 macrophages | M1 macrophages | FLT3LG | FLT3 | 3.0241410475942e-08 | 0 | FLT3L_FLT3 | FLT3L - FLT3 | FLT3 | Secreted Signaling | PMID: 25992210 |
| M2 macrophages | M1 macrophages | FLT3LG | FLT3 | 2.69096888962467e-08 | 0 | FLT3L_FLT3 | FLT3L - FLT3 | FLT3 | Secreted Signaling | PMID: 25992210 |
| M1 macrophages | M2 macrophages | FLT3LG | FLT3 | 2.69096888962467e-08 | 0 | FLT3L_FLT3 | FLT3L - FLT3 | FLT3 | Secreted Signaling | PMID: 25992210 |
| M2 macrophages | M2 macrophages | HGF | MET | 4.59612716695677e-07 | 0 | HGF_MET | HGF - MET | HGF | Secreted Signaling | PMID: 28475121 |
| M1 macrophages | M1 macrophages | ANXA1 | FPR1 | 0.00780486795218312 | 0 | ANXA1_FPR1 | ANXA1 - FPR1 | ANNEXIN | Secreted Signaling | PMID: 23230437 |
| M2 macrophages | M1 macrophages | ANXA1 | FPR1 | 0.00742428766472911 | 0 | ANXA1_FPR1 | ANXA1 - FPR1 | ANNEXIN | Secreted Signaling | PMID: 23230437 |
| M2 macrophages | M1 macrophages | GAS6 | AXL | 0.000622038320847861 | 0 | GAS6_AXL | GAS6 - AXL | GAS | Secreted Signaling | PMID: 27801848 |
| M1 macrophages | M2 macrophages | GAS6 | AXL | 0.000475063967070887 | 0 | GAS6_AXL | GAS6 - AXL | GAS | Secreted Signaling | PMID: 27801848 |
| M2 macrophages | M2 macrophages | GAS6 | AXL | 0.00217906351776086 | 0 | GAS6_AXL | GAS6 - AXL | GAS | Secreted Signaling | PMID: 27801848 |
| M1 macrophages | M1 macrophages | GRN | SORT1 | 0.00200287924169463 | 0 | GRN_SORT1 | GRN - SORT1 | GRN | Secreted Signaling | PMID: 29555433 |
| M2 macrophages | M1 macrophages | GRN | SORT1 | 0.00202766847433951 | 0 | GRN_SORT1 | GRN - SORT1 | GRN | Secreted Signaling | PMID: 29555433 |
| M1 macrophages | M1 macrophages | LGALS9 | PTPRC | 0.0088730844331902 | 0.01 | LGALS9_CD45 | LGALS9 - CD45 | GALECTIN | Secreted Signaling | PMID: 30120235 |
| M2 macrophages | M1 macrophages | LGALS9 | PTPRC | 0.00927992277934385 | 0 | LGALS9_CD45 | LGALS9 - CD45 | GALECTIN | Secreted Signaling | PMID: 30120235 |
| M2 macrophages | M1 macrophages | PROS1 | AXL | 0.000179708420840918 | 0 | PROS1_AXL | PROS1 - AXL | PROS | Secreted Signaling | PMID: 29531161 |
| M2 macrophages | M2 macrophages | PROS1 | AXL | 0.000630235108487659 | 0 | PROS1_AXL | PROS1 - AXL | PROS | Secreted Signaling | PMID: 29531161 |
| M1 macrophages | M1 macrophages | LGALS9 | HAVCR2 | 0.00248277577704248 | 0 | LGALS9_HAVCR2 | LGALS9 - HAVCR2 | GALECTIN | Secreted Signaling | PMID: 27192565 |
| M2 macrophages | M1 macrophages | LGALS9 | HAVCR2 | 0.0025973809723812 | 0 | LGALS9_HAVCR2 | LGALS9 - HAVCR2 | GALECTIN | Secreted Signaling | PMID: 27192565 |
| M1 macrophages | M1 macrophages | LGALS9 | CD44 | 0.0122212785262443 | 0 | LGALS9_CD44 | LGALS9 - CD44 | GALECTIN | Secreted Signaling | PMID: 25065622 |
| M2 macrophages | M1 macrophages | LGALS9 | CD44 | 0.0127796549086529 | 0 | LGALS9_CD44 | LGALS9 - CD44 | GALECTIN | Secreted Signaling | PMID: 25065622 |
| M1 macrophages | M1 macrophages | COL1A1 | ITGA1_ITGB1 | 0.00864210611708491 | 0 | COL1A1_ITGA1_ITGB1 | COL1A1 - (ITGA1+ITGB1) | COLLAGEN | ECM-Receptor | KEGG: hsa04512 |
| M1 macrophages | M2 macrophages | COL1A1 | ITGA1_ITGB1 | 0.00592827567454331 | 0.01 | COL1A1_ITGA1_ITGB1 | COL1A1 - (ITGA1+ITGB1) | COLLAGEN | ECM-Receptor | KEGG: hsa04512 |
| M1 macrophages | M1 macrophages | COL1A1 | ITGA2_ITGB1 | 0.00771340577639639 | 0 | COL1A1_ITGA2_ITGB1 | COL1A1 - (ITGA2+ITGB1) | COLLAGEN | ECM-Receptor | KEGG: hsa04512 |
| M1 macrophages | M2 macrophages | COL1A1 | ITGA2_ITGB1 | 0.00586612333475804 | 0 | COL1A1_ITGA2_ITGB1 | COL1A1 - (ITGA2+ITGB1) | COLLAGEN | ECM-Receptor | KEGG: hsa04512 |
| M1 macrophages | M1 macrophages | FN1 | ITGA4_ITGB1 | 0.0102509152879066 | 0 | FN1_ITGA4_ITGB1 | FN1 - (ITGA4+ITGB1) | FN1 | ECM-Receptor | KEGG: hsa04512 |
| M1 macrophages | M1 macrophages | FN1 | ITGA5_ITGB1 | 0.0310713272804087 | 0 | FN1_ITGA5_ITGB1 | FN1 - (ITGA5+ITGB1) | FN1 | ECM-Receptor | KEGG: hsa04512 |
| M1 macrophages | M1 macrophages | FN1 | ITGA8_ITGB1 | 0.0163635866558191 | 0 | FN1_ITGA8_ITGB1 | FN1 - (ITGA8+ITGB1) | FN1 | ECM-Receptor | KEGG: hsa04512 |
| M1 macrophages | M2 macrophages | FN1 | ITGA8_ITGB1 | 0.0115181437447461 | 0 | FN1_ITGA8_ITGB1 | FN1 - (ITGA8+ITGB1) | FN1 | ECM-Receptor | KEGG: hsa04512 |
| M1 macrophages | M2 macrophages | COL1A1 | ITGA9_ITGB1 | 0.00586209366280104 | 0 | COL1A1_ITGA9_ITGB1 | COL1A1 - (ITGA9+ITGB1) | COLLAGEN | ECM-Receptor | KEGG: hsa04512 |
| M2 macrophages | M2 macrophages | COL1A1 | ITGA9_ITGB1 | 0.00366722438846196 | 0 | COL1A1_ITGA9_ITGB1 | COL1A1 - (ITGA9+ITGB1) | COLLAGEN | ECM-Receptor | KEGG: hsa04512 |
| M1 macrophages | M1 macrophages | FN1 | ITGAV_ITGB1 | 0.033439671029211 | 0 | FN1_ITGAV_ITGB1 | FN1 - (ITGAV+ITGB1) | FN1 | ECM-Receptor | KEGG: hsa04512 |
| M1 macrophages | M2 macrophages | FN1 | ITGAV_ITGB1 | 0.022649961790687 | 0 | FN1_ITGAV_ITGB1 | FN1 - (ITGAV+ITGB1) | FN1 | ECM-Receptor | KEGG: hsa04512 |
| M1 macrophages | M1 macrophages | FN1 | ITGA4_ITGB7 | 0.00476335348550537 | 0 | FN1_ITGA4_ITGB7 | FN1 - (ITGA4+ITGB7) | FN1 | ECM-Receptor | KEGG: hsa04512 |
| M1 macrophages | M1 macrophages | FN1 | CD44 | 0.0933110279228033 | 0 | FN1_CD44 | FN1 - CD44 | FN1 | ECM-Receptor | KEGG: hsa04512 |
| M2 macrophages | M1 macrophages | FN1 | CD44 | 0.0593498307032849 | 0 | FN1_CD44 | FN1 - CD44 | FN1 | ECM-Receptor | KEGG: hsa04512 |
| M1 macrophages | M1 macrophages | COL1A1 | CD44 | 0.0496479611470738 | 0 | COL1A1_CD44 | COL1A1 - CD44 | COLLAGEN | ECM-Receptor | KEGG: hsa04512 |
| M2 macrophages | M1 macrophages | COL1A1 | CD44 | 0.0315796816651822 | 0 | COL1A1_CD44 | COL1A1 - CD44 | COLLAGEN | ECM-Receptor | KEGG: hsa04512 |
| M1 macrophages | M2 macrophages | THBS1 | CD36 | 0.00181126477657553 | 0 | THBS1_CD36 | THBS1 - CD36 | THBS | ECM-Receptor | KEGG: hsa04512 |
| M2 macrophages | M2 macrophages | THBS1 | CD36 | 0.00191761256982311 | 0 | THBS1_CD36 | THBS1 - CD36 | THBS | ECM-Receptor | KEGG: hsa04512 |
| M1 macrophages | M1 macrophages | THBS1 | CD47 | 0.00236929894679482 | 0 | THBS1_CD47 | THBS1 - CD47 | THBS | ECM-Receptor | KEGG: hsa04512 |
| M2 macrophages | M1 macrophages | THBS1 | CD47 | 0.00250832919191552 | 0 | THBS1_CD47 | THBS1 - CD47 | THBS | ECM-Receptor | KEGG: hsa04512 |
| M1 macrophages | M1 macrophages | ALCAM | CD6 | 0.012779907217231 | 0 | ALCAM_CD6 | ALCAM - CD6 | ALCAM | Cell-Cell Contact | KEGG: hsa04514 |
| M2 macrophages | M1 macrophages | CD22 | PTPRC | 2.91967525074225e-05 | 0 | CD22_PTPRC | CD22 - PTPRC | CD22 | Cell-Cell Contact | KEGG: hsa04514 |
| M1 macrophages | M1 macrophages | FCER2 | ITGAM_ITGB2 | 2.63053783224284e-05 | 0 | FCER2A_ITGAM_ITGB2 | FCER2A - (ITGAM+ITGB2) | CD23 | Cell-Cell Contact | PMID: 7621072 |
| M2 macrophages | M1 macrophages | FCER2 | ITGAM_ITGB2 | 2.23153852080915e-05 | 0.01 | FCER2A_ITGAM_ITGB2 | FCER2A - (ITGAM+ITGB2) | CD23 | Cell-Cell Contact | PMID: 7621072 |
| M1 macrophages | M1 macrophages | FCER2 | ITGAX_ITGB2 | 2.24046175528919e-05 | 0 | FCER2A_ITGAX_ITGB2 | FCER2A - (ITGAX+ITGB2) | CD23 | Cell-Cell Contact | PMID: 7621072 |
| M2 macrophages | M1 macrophages | FCER2 | ITGAX_ITGB2 | 1.90062795973852e-05 | 0 | FCER2A_ITGAX_ITGB2 | FCER2A - (ITGAX+ITGB2) | CD23 | Cell-Cell Contact | PMID: 7621072 |
| M1 macrophages | M2 macrophages | PTPRC | CD22 | 2.91967525074225e-05 | 0 | PTPRC_CD22 | PTPRC - CD22 | CD45 | Cell-Cell Contact | PMID: 12115612 |
| M1 macrophages | M2 macrophages | PTPRC | MRC1 | 0.0475897301297507 | 0 | PTPRC_MRC1 | PTPRC - MRC1 | CD45 | Cell-Cell Contact | PMID: 27601670; PMID: 10575006 |
| M2 macrophages | M2 macrophages | PTPRC | MRC1 | 0.0424301561005225 | 0 | PTPRC_MRC1 | PTPRC - MRC1 | CD45 | Cell-Cell Contact | PMID: 27601670; PMID: 10575006 |
| M1 macrophages | M1 macrophages | CD46 | JAG1 | 2.81462546423278e-06 | 0 | CD46_JAG1 | CD46 - JAG1 | CD46 | Cell-Cell Contact | PMID: 23086448 |
| M2 macrophages | M1 macrophages | CD46 | JAG1 | 3.01482886558579e-06 | 0 | CD46_JAG1 | CD46 - JAG1 | CD46 | Cell-Cell Contact | PMID: 23086448 |
| M1 macrophages | M1 macrophages | CD6 | ALCAM | 0.012779907217231 | 0 | CD6_ALCAM | CD6 - ALCAM | CD6 | Cell-Cell Contact | PMID: 23602662 |
| M1 macrophages | M2 macrophages | CD80 | CD28 | 1.13800085498083e-06 | 0 | CD80_CD28 | CD80 - CD28 | CD80 | Cell-Cell Contact | PMID: 23954143 |
| M2 macrophages | M2 macrophages | CD80 | CD28 | 1.35027984009564e-06 | 0 | CD80_CD28 | CD80 - CD28 | CD80 | Cell-Cell Contact | PMID: 23954143 |
| M1 macrophages | M2 macrophages | CD86 | CD28 | 2.24130958044839e-05 | 0 | CD86_CD28 | CD86 - CD28 | CD86 | Cell-Cell Contact | PMID: 23954143 |
| M2 macrophages | M2 macrophages | CD86 | CD28 | 2.34560857893367e-05 | 0 | CD86_CD28 | CD86 - CD28 | CD86 | Cell-Cell Contact | PMID: 23954143 |
| M1 macrophages | M1 macrophages | CD99 | PILRA | 0.0417394136741173 | 0 | CD99_PILRA | CD99 - PILRA | CD99 | Cell-Cell Contact | PMID: 18234675 |
| M2 macrophages | M1 macrophages | CD99 | PILRA | 0.0543660828480785 | 0 | CD99_PILRA | CD99 - PILRA | CD99 | Cell-Cell Contact | PMID: 18234675 |
| M2 macrophages | M2 macrophages | CD99 | CD99 | 0.135919757960894 | 0 | CD99_CD99 | CD99 - CD99 | CD99 | Cell-Cell Contact | KEGG: hsa04514 |
| M2 macrophages | M2 macrophages | EFNA4 | EPHA5 | 1.42490078275651e-09 | 0 | EFNA4_EPHA5 | EFNA4 - EPHA5 | EPHA | Cell-Cell Contact | PMID: 15114347 |
| M2 macrophages | M2 macrophages | EFNA5 | EPHA5 | 1.26209675785861e-09 | 0 | EFNA5_EPHA5 | EFNA5 - EPHA5 | EPHA | Cell-Cell Contact | PMID: 15114347 |
| M2 macrophages | M1 macrophages | EFNA5 | EPHB2 | 1.7391042431245e-07 | 0 | EFNA5_EPHB2 | EFNA5 - EPHB2 | EPHA | Cell-Cell Contact | PMID:15107857; PMID: 15114347 |
| M1 macrophages | M1 macrophages | ICAM1 | ITGAX_ITGB2 | 0.0108140100075849 | 0 | ICAM1_ITGAX_ITGB2 | ICAM1 - (ITGAX+ITGB2) | ICAM | Cell-Cell Contact | PMID: 16252253 |
| M1 macrophages | M2 macrophages | ICAM1 | ITGAX_ITGB2 | 0.00777763113041886 | 0 | ICAM1_ITGAX_ITGB2 | ICAM1 - (ITGAX+ITGB2) | ICAM | Cell-Cell Contact | PMID: 16252253 |
| M1 macrophages | M1 macrophages | ICAM1 | ITGAM_ITGB2 | 0.0126729740865572 | 0 | ICAM1_ITGAM_ITGB2 | ICAM1 - (ITGAM+ITGB2) | ICAM | Cell-Cell Contact | KEGG: hsa04514 |
| M1 macrophages | M2 macrophages | ICAM1 | ITGAM_ITGB2 | 0.0102584551840116 | 0 | ICAM1_ITGAM_ITGB2 | ICAM1 - (ITGAM+ITGB2) | ICAM | Cell-Cell Contact | KEGG: hsa04514 |
| M1 macrophages | M1 macrophages | ICAM1 | SPN | 7.30187299263342e-07 | 0 | ICAM1_SPN | ICAM1 - SPN | ICAM | Cell-Cell Contact | PMID: 1683685 |
| M1 macrophages | M2 macrophages | ICAM1 | SPN | 6.71475873824988e-07 | 0 | ICAM1_SPN | ICAM1 - SPN | ICAM | Cell-Cell Contact | PMID: 1683685 |
| M1 macrophages | M2 macrophages | HLA-DPA1 | CD4 | 0.0744129820793202 | 0 | HLA-DPA1_CD4 | HLA-DPA1 - CD4 | MHC-II | Cell-Cell Contact | KEGG: hsa04514 |
| M2 macrophages | M2 macrophages | HLA-DPA1 | CD4 | 0.0717189155101521 | 0 | HLA-DPA1_CD4 | HLA-DPA1 - CD4 | MHC-II | Cell-Cell Contact | KEGG: hsa04514 |
| M1 macrophages | M2 macrophages | HLA-DPB1 | CD4 | 0.0707287035047738 | 0 | HLA-DPB1_CD4 | HLA-DPB1 - CD4 | MHC-II | Cell-Cell Contact | KEGG: hsa04514 |
| M1 macrophages | M2 macrophages | HLA-DQA1 | CD4 | 0.0464554159278664 | 0 | HLA-DQA1_CD4 | HLA-DQA1 - CD4 | MHC-II | Cell-Cell Contact | KEGG: hsa04514 |
[truncated: 3,587 more chars]
